# Characterization of Antennal Chemosensilla and Associated Chemosensory Genes in the Orange Spiny Whitefly, *Aleurocanthus spiniferus* (Quaintanca)

**DOI:** 10.3389/fphys.2022.847895

**Published:** 2022-02-28

**Authors:** Yu-Qing Gao, Zhen-Zhen Chen, Meng-Yuan Liu, Chang-Yuan Song, Zhi-Fei Jia, Fang-Hua Liu, Cheng Qu, Youssef Dewer, Hai-Peng Zhao, Yong-Yu Xu, Zhi-Wei Kang

**Affiliations:** ^1^College of Plant Protection, Shandong Agricultural University, Tai’an, China; ^2^Beijing Key Laboratory of Environment Friendly Management on Fruit Diseases and Pests in North China, Institute of Plant and Environment Protection, Beijing Academy of Agriculture and Forestry Sciences, Beijing, China; ^3^Phytotoxicity Research Department, Central Agricultural Pesticide Laboratory, Agricultural Research Center, Giza, Egypt

**Keywords:** *Aleurocanthus spiniferus*, transcriptome, antennal sensilla, chemosensory genes, expression patterns

## Abstract

The insect chemosensory system plays an important role in many aspects of insects’ behaviors necessary for their survival. Despite the complexity of this system, an increasing number of studies have begun to understand its structure and function in different insect species. Nonetheless, the chemosensory system in the orange spiny whitefly *Aleurocanthus spiniferus*, as one of the most destructive insect pests of citrus in tropical Asia, has not been investigated yet. In this study, the sensillum types, morphologies and distributions of the male and female antennae of *A. spiniferus* were characterized using scanning electron microscopy. In both sexes, six different sensilla types were observed: trichodea sensilla, chaetica sensilla, microtrichia sensilla, coeloconic sensilla, basiconic sensilla, and finger-like sensilla. Moreover, we identified a total of 48 chemosensory genes, including 5 odorant-binding proteins (OBPs), 12 chemosensory proteins (CSPs), 3 sensory neuron membrane proteins (SNMPs), 6 odorant receptors (ORs), 8 gustatory receptors (GRs), and 14 ionotropic receptors (IRs) using transcriptome data analysis. Tissue-specific transcriptome analysis of these genes showed predominantly expression in the head (including antennae), whereas CSPs were broadly expressed in both head (including the antennae) and body tissue of adult *A. spiniferus*. In addition, the expression profiling of selected chemosensory genes at different developmental stages was examined by quantitative real time-PCR which was mapped to the transcriptome. We found that the majority of these genes were highly expressed in adults, while *AspiORco*, *AspiGR1*, *AspiGR2*, and *AspiIR4* genes were only detected in the pupal stage. Together, this study provides a basis for future chemosensory and genomic studies in *A. spiniferus* and closely related species. Furthermore, this study not only provides insights for further research on the molecular mechanisms of *A. spiniferus*-plant interactions but also provides extensive potential targets for pest control.

## Introduction

In insects, the chemosensory system is extremely critical for detecting and discriminating specific chemical signals in the environment necessary for their survival and reproduction (Hallem et al., 2006; Knolhoff and Heckel, 2014; Kang et al., 2020). The insect peripheral chemosensory system comprises odorant receptors (ORs), gustatory receptors (GRs), ionotropic receptors (IRs), odorant binding proteins (OBPs), chemosensory proteins (CSPs), and sensory neuron membrane proteins (SNMPs) (Fleischer et al., 2018; Sun et al., 2020; Liu et al., 2021). These protein families have been identified from a large number of insect species, however, they still remain unidentified from several insect species.

Odorant binding proteins are small soluble olfactory proteins which are thought to be responsible for transporting hydrophobic odor molecules through the sensillum lymph to odorant receptors, which are housed on the dendritic membrane of olfactory sensory neurons (Wang et al., 2020; Tian et al., 2021). Previous studies have shown that OBPs are expressed selectively in different types of sensilla on the antenna, which are considered the minimum functional units for chemoreception. In general, OBPs show higher binding affinities with ligands *in vitro.* For instance, *ApisOBP3* and *SaveOBP7* showed a high binding affinity with aphid alarm pheromone, (E)-beta-farnesene, whereas, *ApisOBP1*, *ApisOBP3*, *ApisOBP8*, *ApisOBP7*, and *SaveOBP7* showed a high binding affinity with plant volatiles (Qiao et al., 2009; Zhong et al., 2012).

Chemosensory proteins represent another class of small soluble proteins abundant in the lymph of chemosensilla (Pelosi et al., 2006). They are also broadly expressed in various organs, such as palps, proboscis, legs, wings, eyes, and pheromone glands (Hua et al., 2012, 2013; Gu et al., 2013; Liu et al., 2014; Zhu et al., 2016; Li et al., 2020). CSPs are different from OBPs in amino acid sequence and structure, but appear to be similar in functions, although better evidence is needed to clarify their role in olfaction (Calvello et al., 2005; Tian et al., 2021). The first CSP protein was discovered in the regenerating legs of the American cockroach, *Periplaneta americana* (Nomura et al., 1992). OS-D, a related CSP, was later cloned from *Drosophila melanogaster* antennae and is thought to be involved in pheromone binding (Mckenna et al., 1994). More insect CSP genes have recently been identified and characterized as a result of the completion of diverse insect genome sequences (Pelosi et al., 2017). Various numbers of CSP genes have been identified in different insect species. For instance, 4 CSPs were reported in *D. melanogaster*, six in *Apis mellifera* (Forêt et al., 2007), and 20 in *Bombyx mori* (Gong et al., 2007), 70 in *Locusta migratoria* (Picimbon et al., 2000), 43 in *Aedes aegypti* (Mei et al., 2018), three in *Heliothis virescens* (Picimbon et al., 2001) and 27 in *Helicoverpa armigera* (Agnihotri et al., 2021).

Odorant receptors were the first insect chemosensory receptor family which were identified using a bioinformatics screen of the *D. melanogaster* genome (Gao and Chess, 1999). The typical ORs are seven-transmembrane receptors with a reversed membrane topology. In general, ORs have a wide variety of odor affinities, and a single odorant molecule may bind to a number of olfactory receptors with variable affinities, which are dependent on physio-chemical features of molecules such as their molecular weights (Buck, 2004). Once the odorant interacts with the odorant receptor, it undergoes structural modifications and binds and activates the olfactory-type G protein on the inside of the olfactory receptor neuron. Activated olfactory receptors trigger nerve impulses that transmit information about the odor to the brain (Fleischer et al., 2018).

In insect gustatory organs, gustatory receptors GRs are a large gene family, which are implicated in host-seeking (Hallem et al., 2006; Agnihotri et al., 2016). Most of these GR proteins have the typical structure of seven transmembrane domains, were initially identified in the *D. melanogaster* genome based on a bioinformatic approach (Clyne et al., 2000). Further studies discovered that *D. melanogaster* has 68 gustatory receptor proteins, which are encoded by 60 gustatory receptor genes by alternative splicing (Dunipace et al., 2001; Scott et al., 2001; Robertson et al., 2003). The amino acid sequences of most gustatory receptor proteins are quite diverse, with just 8–12% sequence similarity. Some of this variance might help to increase the diversity of GRs’ responses to ligands (Robertson et al., 2003). GRs were classified as sugar receptors, CO_2_ receptors, GR43a-like receptors, bitter receptors, sex pheromone receptors, and unknown receptors based on the ligands to which they respond (Jones et al., 2007; Sato et al., 2011). With the development of insect genome sequencing, insect GR genes have been discovered in an increasing number of species: *Anopheles gambiae* has 52 Gr genes that encode 76 GR proteins (Hill et al., 2002), and *A. aegypti* has 79 GR genes that encode 114 GR proteins (Kent et al., 2008). *Bombyx mori* and *Tribolium castaneum* have 65 and 220 GR genes, respectively (Richards et al., 2008; Wanner and Robertson, 2008). Among all insect species investigated, *H. armigera* had the second-highest number of GR genes (197) (Xu et al., 2016).

Compared to other chemosensory gene families, SNMPs are a small family where only one or two members have been reported (SNMP1 and SNMP2). SNMP1 is found to be co-expressed with pheromone receptors in pheromone responsive neurons and seems to be an indicator of pheromone-responsive neurons (Jiang et al., 2016; Fleischer et al., 2018; Zhang et al., 2018). In contrast, SNMP2 is expressed in cells surrounding the neuron clusters supporting cells (Jiang et al., 2016). Recently, a novel SNMP gene, SNMP3 was found specifically expressed in the larval midgut of (*B. mori*), which assumed to be involved in the immune response to virus and bacterial infections (Zhang et al., 2018).

The orange spiny whitefly *Aleurocanthus spiniferus* is a serious insect pest of citrus, grapes and tea plants (Tang et al., 2015; Nugnes et al., 2020; Radonjić and Hrnčić, 2021). It also causes significant damage to more than 90 plant species from 38 families widely distributed throughout the world (Tang et al., 2015; Radonjić and Hrnčić, 2021). Due to the serious damage caused by this pest, it has been reported as quarantine pest in many countries (EPPO A2 list^1^). To date, there are limited studies on *A. spiniferus* that are mainly focused on population dynamics, insecticide selections, biological control and color plates (Mokrane et al., 2020; Nugnes et al., 2020; Tian et al., 2020). In this study, we investigated the structure, distribution, and abundance of the antennal sensilla in the adult male and female *A. spiniferus* by scanning electron microscopy. Transcriptome sequencing of *A. spiniferus* was performed to identify the candidate chemosensory genes. Moreover, tissue expression patterns of the putative chemosensory genes were assessed by quantitative real-time PCR (qPCR). These findings provide a basis for future chemosensory and genomic studies in *A. spiniferus* and closely related species.

## Materials and Methods

### Insect Materials

In this study, *A. spiniferus* were collected form tea cultivar ‘Huangjinya’ (*Camellia sinensis*) that were maintained in the greenhouse in Jinan, Shandong, China. Due to the low sex ratio of male, we are unable to get high quality and quantity of RNAs from male head tissues. Thus, we conducted the transcriptome analysis with the mixture of male and female head and bodies tissues. Heads with antennae (200 heads per replicate) and bodies only with thoraxes, legs, wings and abdomens (50 bodies per replicate) were dissected, collected in liquid nitrogen and then subjected to RNA extractions using RNAiso (Takara Bio, Tokyo, Japan) according to the manufacturer’s instructions. The RNA integrity was verified by 1% agarose gel electrophoresis and the quantity was assessed with a Nanodrop ND-2000 spectrophotometer (Nanodrop Technologies, Wilmington, DE, United States).

### Scanning Electron Microscopy

Approximately 50 female and male adults were used for the identification of antennal sensilla using scanning electron microscopy (SEM). Experiments were conducted followed the method previously descried by Zhang et al. (2015). Whole bodies of *A. spiniferus* were putted into 1.5 ml clean Eppendorf tubes and washed twice using 0.1 M phosphate-buffered saline (PBS, pH 7.2) each for 5 min. After the preliminary cleaning, all of these samples were transferred into ultrasonic bath for deep cleaning (250 W, 30 s). Cleaned samples were fixed in 2.5% glutaraldehyde at 4°C overnight. After the fixation, all samples were washed five times in PBS (0.1 M, pH 7.2) for 20 min each, and then incubated in osmium tetroxide for 15 h. Dehydration of all samples was conducted in ethanol series (45%, 55%, 75%, 85%, 95% for 30 min each, and 100% for 14 h). Then, all samples were transferred into new Eppendorf tubes with 0.5 ml 100% ethanol for 7 h. Dehydrated samples were rinsed in isoamyl acetate for 1 h each. Finally, all samples were dried, mounted on aluminum stubs and gold coated. Antennal sensilla were observed and recorded using ZEISS Ultra-55 Scanning Electron Microscope (Carl Zeiss Meditec, Oberkochen, Baden-Württemberg, Germany). Student’s *t*-test was used for the comparison of the difference between male and female (*P* < 0.05).

### Transcriptome Sequences

Three biological replicates of high quality and quantity RNAs from heads and bodies of *A. spiniferus* were subjected to cDNA library construction and sequencing on the Illumina, Inc. (San Diego, CA, United States) by Novogene Bioinformatics Technology Co., Ltd. (Beijing, China). Clean data (clean reads) were obtained by removing adapter-containing reads, higher N rate reads (N rates > 10%), and low-quality reads (50% bases with *Q*-score ≤ 5) from the raw data (raw reads) using in-house Perl scripts. Clean read assembly was carried out with the short-read assembly program Trinity with min_kmer_cov set to 2 by default and all other parameters set default. The annotation of unigenes was performed by NCBI BLASTx search against the Nr protein database, with an *E-*value threshold of 1 × 10^––5^. The blast results were then imported into the Blast2GO pipeline for GO annotation. The longest open reading frame ORF for each unigene was determined by the NCBI ORF Finder tool^2^. Differential expression analysis was performed using the DESeq2 R package (1.20.0). DESeq2 provides statistical routines for determining differential expression in digital gene expression data using a model based on the negative binomial distribution. The resulting *P*-values were adjusted using the Benjamini and Hochberg’s approach for controlling the false discovery rate. Genes with an adjusted *P*-value < 0.05 found by DESeq2 were assigned as differentially expressed. Expression levels were expressed in terms of FPKM values (fragments per kilobase per million reads), which was calculated by RSEM (RNA-Seq by Expectation-Maximization) with default parameters (Kang et al., 2017b). The sequences reported in this paper have been deposited in the GenBank SRA database (BioProject ID: PRJNA792195).

### Verification of Candidate Chemosensory Genes in *Aleurocanthus spiniferus*

Genes annotated as chemosensory genes in *A. spiniferus* were further verified by BLASTp (*E*-value < 1 × 10^–5^ and Identity > 30%) in NCBI non-redundant protein sequences database with algorithm of PSI-BLAST. Furthermore, we also used the amino acid sequences of OBPs and CSPs of *B. tabaci* against our transcriptome database to avoid the omission of transcriptome annotation (Zeng et al., 2019). The signal peptide and conserved domains of OBPs and CSPs of *A. spiniferus* were predicted by SignalP 5.0 Server^3^ and SMART (simple modular architecture research tool^4^). Transmembrane domains in ORs, GRs and IRs were predicted by TMHMM - 2.0^5^.

### Sequence Alignment and Phylogenetic Analysis

Sequence alignment and phylogenetic analysis were conducted as described by Zeng et al. (2019). Amino acid sequences of candidate OBPs, CSPs, SNMPs, ORs, GRs, and IRs were aligned by ClustalW used gap opening penalty 10 and gap extension penalty 0.2. The alignments were further manually edited. Phylogenetic trees were subsequently constructed by the maximum likelihood method using MEGA X based on the model WAG and gamma distributed with bootstrap 1000 (Kumar et al., 2018). The trees were further edited using the ITOL tool (Letunic and Bork, 2019). All amino acid sequences used in this work are presented in Supplementary Table S1.

### Expression Pattern Analysis of Chemosensory Genes by Quantitative Real-Time Polymerase Chain Reaction

RNAs of *A. spiniferus* from different tissues (heads and bodies) and developmental stages (second nymphs, third nymphs, puparia/fourth nymphs, female adults and male adults) were extracted by RNAiso (Takara Bio., Tokyo, Japan). The cDNA was synthesized from total RNA using FastQuant RT Kit (With gDNase) (Tiangen, Beijing, China) according to the standard manufacturer’s protocol. Gene-specific primers were designed by Primer Premier 6 (PREMIER Biosoft International, Palo Alto, CA, United States), which are listed in Supplementary Table S2. qPCR reaction was conducted in a total volume of 20 μL containing: 10 μL of 50× SYBR Premix Ex Taq, 0.8 μL of primer (10 mM), 0.8 μL of sample cDNA, and 7.6 μL sterilized ultra-pure grade H_2_O. The cycling conditions were as follows: 95°C for 30 s, followed by 40 cycles of 95°C for 5 s and 55°C for 30 s. Three technical and three biological replicates were used for each sample. Relative quantification was performed using the Comparative 2^–ΔΔCT^ method (Livak and Schmittgen, 2001). Transcription levels of these chemosensory genes were normalized by reference gene RPS28 (Kang et al., 2017a; Kong et al., 2021). Heatmaps of chemosensory genes were constructed by pheatmap in R 4.0.4 as Liu et al. (2020) reported. Differences of selected chemosensory genes between male and female were subjected to Student’s *t*-test (*P* < 0.05), while one-way analysis of variance (ANOVA) followed by separation of means with the Fisher’s protected least significant difference (LSD) test (*P* < 0.05) was used for the difference among the different developmental stages.

## Results

### Morphology of Antennal Sensilla of *Aleurocanthus spiniferus*

**FIGURE 1:**
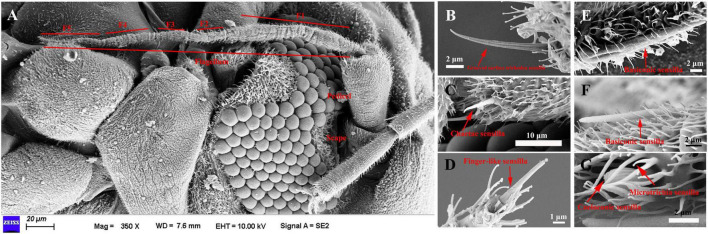
The types of sensilla present on *A. spiniferus* antennae. **(A)** Female antenna. **(B)** Grooved surface richodea sensilla **(C)** Chaetae sensilla. **(D)** Figure-like sensilla. **(E)** Basiconic sensilla. **(F)** Basiconic sensilla. **(G)** Coeloconic and microtrichia sensilla.

The length of female antennae was significantly longer than that of male (Table 1). Six different sensilla types were observed: trichodea sensilla, chaetica sensilla, microtrichia sensilla, coeloconic sensilla, basiconic sensilla, and finger-like sensilla. There was no difference of the distribution and structure of other sensilla between the two sexes (Figure 1A). Grooved surface trichodea sensilla were only found on the scape (Figure 1B and Table 1). Chaetae sensilla presented on the scape and pedicel female *A. spiniferus*, while it was found on the pedicel and flagellum (Figure 1C and Table 1). Finger-like sensilla was only found on the tips of the fifth flagellum of *A. spiniferus* (Figure 1D and Table 1). Basiconic sensilla looks like a sword and was found on the flagellar subsegment 5 (Figures 1E,F and Table 1). Coeloconic sensilla were surrounded by microtrichia sensilla, and microtrichia sensilla were the most abundant and widely distributed sensilla on the entire antennae of *A. spiniferus* (Figure 1G and Table 1).

**TABLE 1:** Antennal length and chemosensillar distribution on the antennae of *A. spiniferus.*

	Segment		Length (μm)	The number of antennal sensillar					
				Microtrichia sensilla	Grooved surface trichodea sensilla	Chaetae sensilla	Coeloconic sensilla	Basiconic sensilla	Finger-like sensilla
Female	Total		296 ± 11*a*	More	1	7	4	1	1
	Scape		16.54 ± 1.27*a*	More	1	1	0	0	0
	Pedicel		49.26 ± 4.07*a*	More	0	6	0	0	0
		F1	101.61 ± 3.24*a*						
		F2	21.60 ± 2.52*a*						
	Flagellum	F3	21.70 ± 2.15*a*	More	0	0	4	4	1
		F4	28.58 ± 3.17*a*						
		F5	57.88 ± 3.95*a*						
Male	Total		247 ± 7*b*	More	1	7	4	4	1
	Scape		15.14 ± 0.70*b*	More	1	0	0	0	0
	Pedicel		44.01 ± 3.36*b*	More	0	5	0	0	0
		F1	78.12 ± 2.20*b*						
		F2	15.83 ± 2.02*b*						
	Flagellum	F3	24.77 ± 2.13*b*	More	0	2	4	4	1
		F4	24.77 ± 2.13*b*						
		F5	45.22 ± 2.58*b*						

### Transcriptome Analysis Data of *Aleurocanthus spiniferus*

The transcriptome data was presented in Table 2. The total number of unigenes was 75298. Max, Min, and mean length were 38279, 301, and 782 bp, respectively (Table 2). GC percent of sequences from bodies showed a little bit higher than that from heads (Table 2). Homology analyses results showed that the most similar sequences of 75.1% sequences were from *B. tabaci* (Supplementary Figure S1). Functional annotation was performed using NR, NT, KO, Swissprot, PFAM, GO, and KOG databases (Supplementary Table S3). Based on the GO categorization, differential expressed genes were enriched in protein metabolic process, hydrolase activity, cellular protein metabolic process, intracellular non-membrane-bounded organelle, non-membrane-bounded organelle and translation (Supplementary Figure S2).

**TABLE 2:** Assembly summary of the *A. spiniferus* transcriptome.

Group name	Head			Body		
	1	2	3	1	2	3
Raw reads	29,663,967	28,041,038	31,218,486	29,511,378	27,409,600	30,642,126
Clean reads	29,080,717	27,293,657	30,829,257	28,968,202	26,996,849	30,291,895
GC percent	38.7%	38.8%	37.57%	40.24%	40.16%	39.3%
Total number of unigenes	75,298					
N50 length	2,355					
Max length	38,279					
Min length	301					
Mean length	782					

### Putative Chemosensory Genes in *Aleurocanthus spiniferus*

**FIGURE 2:**
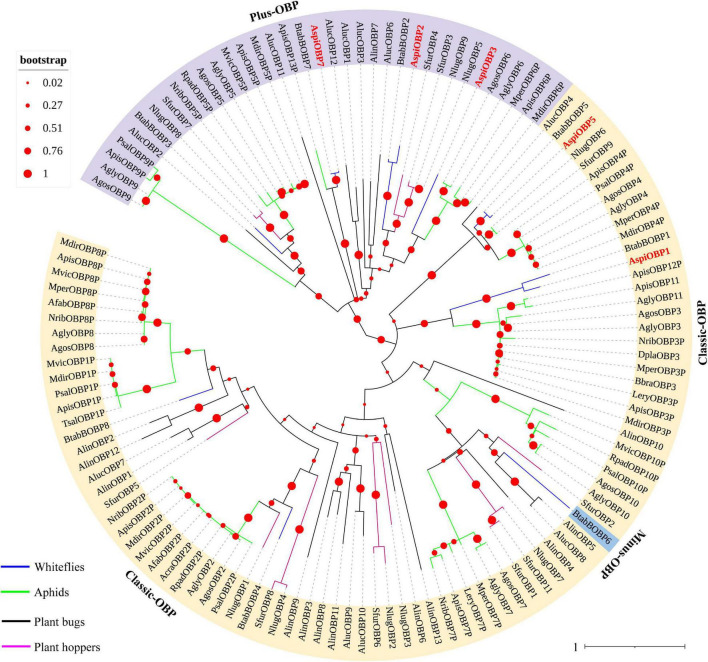
Phylogenetic analysis of putative odorant-binding proteins (OBPs) of *A. spiniferus*. The phylogenetic tree was built using OBP sequences from whitefly species (Btab, *Bemisia tabaci*; Aspi, *A. spiniferus*), aphid species (Apis, *Acyrthosiphon pisum*; Mper, *Myzus persicae*; Agos, *Aphis gossypii*; Psal, *Pterocomma salicis*; Agly, *Aphis glycines*; Mdir, *Metopolophium dirhodum*; Mvic, *Megoura viciae*; Bbra, *Brevicoryne brassicae*; Lery, *Lipaphis erysimi*; Afab, *Aphis fabae*; Acra, *Aphis craccivora*; Tsal, *Tuberolachnus salignus*; Dpla, *Drepanosiphum platanoidis*; Nrib, *Nasonovia ribisnigri*; Rpad, *Rhopalosiphum padi*), plant hoppers (Sfur, *Sogatella furcifera*; Nlug, *Nilaparvata lugens*), and plant bugs (Aluc, *Apolygus lucorum*; Alin, *Adelphocoris lineolatus*).

In this study, a total of five transcripts encoding candidate OBPs were identified in the transcriptome of *A. spiniferus* (Table 3). The number of putative OBPs was a little bit lower than that identified in the genome of *B. tabaci* (eight OBPs). All of the putative OBPs had full-length ORFs, and only *AspiOBP7* without signal peptide (Table 3). A phylogenetic tree was constructed using the identified OBPs from whiteflies (*A. spiniferus* and *B. tabaci*), aphids (*Acyrthosiphon pisum*, *Aphis glycines*, *Brevicoryne brassicae*, *Metopolophium dirhodum*, *Rhopalosiphum padi*, *Lipaphis erysimi*, *Aphis fabae*, *Aphis craccivora*, *Tuberolachnus salignus*, *Myzus persicae*, *Aphis gossypii, Drepanosiphum platanoidis*, and *Nasonovia ribisnigri*), plant bugs (*Apolygus lucorum* and *Adelphocoris lineolatus*) and plant hoppers (*Nilaparvata lugens* and *Sogatella furcifera*) (Figure 2 and Supplementary Figure S3). In the phylogenetic tree, *AspiOBP1*, *AspiOBP2*, *AspiOBP5*, and *AspiOBP7* were clustered with OBPs from *B. tabaci*, while *AspiOBP3* was clustered with OBPs from aphids (Figure 2).

**TABLE 3:** Candidate chemosensory genes in *A. spiniferus*.

Gene name	Unigene IDs	ORF (aa)	Signal peptide	Homology search with known proteins		
				Best blastp hit	E-value	Identity (%)
*AspiOBP1*	Cluster-17909.36062	143	1–23	AQS80474.1| odorant binding protein 1 [*Bemisia tabaci*]	1e-58	59.29
*AspiOBP2*	Cluster-17909.4418	248	1–22	XP_018902547.1| PREDICTED: uncharacterized protein LOC109034040 [*Bemisia tabaci*]	1e-89	64.29
*AspiOBP3*	Cluster-17909.4100	265	1–26	AQS80478.1| odorant binding protein 5 [*Bemisia tabaci*]	3e-114	83.51
*AspiOBP5*	Cluster-17909.46264	223	1–28	AMQ76484.1| odorant-binding protein 31 [*Apolygus lucorum*]	7e-15	33.70
*AspiOBP7*	Cluster-17909.17740	141	NF	XP_018909253.1| PREDICTED: uncharacterized protein LOC109038604 [*Bemisia tabaci*]	2e-68	84.56
*AspiCSP2*	Cluster-17909.27950	133	1–19	XP_018914249.1| PREDICTED: ejaculatory bulb-specific protein 3-like [*Bemisia tabaci*]	1e-72	83.46
*AspiCSP3*	Cluster-17909.9823	132	1–20	AIT38537.1| chemosensory protein 3 [*Bemisia tabaci*]	2e-50	61.54
*AspiCSP4*	Cluster-17909.11369	109	1–20	XP_018912154.1| PREDICTED: ejaculatory bulb-specific protein 3-like [*Bemisia tabaci*]	5e-45	68.81
*AspiCSP5*	Cluster-17909.27981	173	1–17	AQS80473.1| chemosensory protein 13 [*Bemisia tabaci*]	4e-59	56.82
*AspiCSP7*	Cluster-17909.18369	125	1–20	ANJ43349.1| chemosensory protein 4 [*Bemisia tabaci*]	2e-54	64.34
*AspiCSP8*	Cluster-17909.18168	140	1–27	XP_018914236.1| PREDICTED: ejaculatory bulb-specific protein 3-like [*Bemisia tabaci*]	5e-44	58.12
*AspiCSP9*	Cluster-17909.19859	124	1–20	XP_018898412.1| PREDICTED: ejaculatory bulb-specific protein 3-like [*Bemisia tabaci*]	7e-62	78.23
*AspiCSP10*	Cluster-17909.8133	136	1–22	XP_018914236.1| PREDICTED: ejaculatory bulb-specific protein 3-like [*Bemisia tabaci*]	3e-57	66.91
*AspiCSP12*	Cluster-17909.30984	132	NF	XP_018916537.1| PREDICTED: ejaculatory bulb-specific protein 3-like [*Bemisia tabaci*]	4e-46	57.03
*AspiCSP14*	Cluster-11558.0	142	1–22	XP_018912701.1| PREDICTED: ejaculatory bulb-specific protein 3-like [*Bemisia tabaci*]	9e-74	89.44
*AspiCSP15*	Cluster-17909.27059	109	NF	XP_018916603.1| PREDICTED: ejaculatory bulb-specific protein 3-like [*Bemisia tabaci*]	2e-69	92.59
*AspiCSP16*	Cluster-17909.35439	149	1–21	XP_018913601.1| PREDICTED: ejaculatory bulb-specific protein 3-like [*Bemisia tabaci*]	8e-66	76.12
*AspiSNMP1*	Cluster-17909.47564	494		XP_018916083.1| PREDICTED: sensory neuron membrane protein 1-like [*Bemisia tabaci*]	0.0	66.87
*AspiSNMP2.1*	Cluster-17909.2178	564		XP_018909770.1| PREDICTED: sensory neuron membrane protein 2-like [*Bemisia tabaci*]	0.0	59.96
*AspiSNMP2.2*	Cluster-17909.23140	457		XP_018914385.1| PREDICTED: sensory neuron membrane protein 2-like [*Bemisia tabaci*]	0.0	79.21
*AspiORco*	Cluster-17909.2187	472		XP_018916513.1| PREDICTED: odorant receptor coreceptor [*Bemisia tabaci*]	0.0	76.82
*AspiOR2*	Cluster-17909.26288	423		XP_018901087.1| PREDICTED: uncharacterized protein LOC109033105 [*Bemisia tabaci*]	1e-35	31.05
*AspiOR3*	Cluster-15455.0	418		XP_018901080.1| PREDICTED: uncharacterized protein LOC109033100 [*Bemisia tabaci*]	3e-24	41.50
*AspiOR4*	Cluster-17909.52227	179		XP_018901080.1| PREDICTED: uncharacterized protein LOC109033100 [*Bemisia tabaci*]	6e-15	32.65
*AspiOR5*	Cluster-17909.1519	272		XP_018901080.1| PREDICTED: uncharacterized protein LOC109033100 [*Bemisia tabaci*]	6e-18	36.62
*AspiOR6*	Cluster-17909.15899	138		XP_018901202.1| PREDICTED: uncharacterized protein LOC109033177 [*Bemisia tabaci*]	2e-19	39.69
*AspiGR1*	Cluster-17909.53621	239		XP_018917335.1| PREDICTED: uncharacterized protein LOC109044210 [*Bemisia tabaci*]	1e-49	65.32
*AspiGR2*	Cluster-17909.51990	136		XP_016657079.2| gustatory receptor for sugar taste 64a-like [*Acyrthosiphon pisum*]	4e-14	42.11
*AspiGR3*	Cluster-18904.0	176		XP_018903763.1| PREDICTED: gustatory receptor for sugar taste 64f-like [*Bemisia tabaci*]	2e-116	96.00
*AspiGR4*	Cluster-18974.0	184		XP_018910036.1| PREDICTED: uncharacterized protein LOC109039135 [*Bemisia tabaci*]	3e-110	97.09
*AspiGR5*	Cluster-17909.6070	108		XP_018910041.1| PREDICTED: gustatory receptor for sugar taste 43a-like [*Bemisia tabaci*]	3e-23	65.75
*AspiGR6*	Cluster-17909.19648	97		XP_025419807.1| gustatory receptor for sugar taste 61a-like [*Sipha flava*]	2e-17	49.44
*AspiGR7*	Cluster-14878.0	87		XP_018910041.1| PREDICTED: gustatory receptor for sugar taste 43a-like [*Bemisia tabaci*]	3e-40	89.74
*AspiGR8*	Cluster-17909.12848	73		XP_027845934.1| gustatory receptor for sugar taste 61a-like isoform X2 [*Aphis gossypii*]	1e-08	50.00
*AspiIR1*	Cluster-14132.0	416		XP_018902736.1| PREDICTED: uncharacterized protein LOC109034187 [*Bemisia tabaci*]	4e-131	55.85
*AspiIR2*	Cluster-8053.0	267		XP_018916090.1| PREDICTED: glutamate receptor ionotropic, delta-1 [*Bemisia tabaci*]	1e-162	88.35
*AspiIR3*	Cluster-17909.2243	605		XP_018911141.1| PREDICTED: ionotropic receptor 25a [*Bemisia tabaci*]	0.0	86.28
*AspiIR4*	Cluster-17909.4915	603		XP_018908639.1| PREDICTED: ionotropic receptor 21a [*Bemisia tabaci*]	0.0	72.12
*AspiIR5*	Cluster-17909.17580	286		XP_018909625.1| PREDICTED: glutamate receptor ionotropic, kainate 4-like [*Bemisia tabaci*]	1e-157	79.23%
*AspiIR6*	Cluster-17909.52928	909		XP_018900134.1| PREDICTED: glutamate receptor ionotropic, kainate 3-like [*Bemisia tabaci*]	0.0	89.99
*AspiIR7*	Cluster-3371.0	548		XP_018918104.1| PREDICTED: glutamate receptor ionotropic, delta-2 [*Bemisia tabaci*]	0.0	81.93
*AspiIR8*	Cluster-17909.14487	580		XP_018911078.1| PREDICTED: glutamate receptor ionotropic, kainate 2-like isoform X1 [*Bemisia tabaci*]	0.0	98.02
*AspiIR9*	Cluster-17909.54060	549		XP_018904379.1| PREDICTED: uncharacterized protein LOC109035262 [*Bemisia tabaci*]	0.0	74.50
*AspiIR10*	Cluster-17909.50436	912		XP_018907677.1| PREDICTED: glutamate receptor ionotropic, kainate 2-like isoform X2 [*Bemisia tabaci*]	0.0	91.28
*AspiIR11*	Cluster-17909.4133	919		XP_018914442.1| PREDICTED: glutamate receptor ionotropic, kainate 2 [*Bemisia tabaci*]	0.0	97.26
*AspiIR12*	Cluster-17909.605	893		XP_018906951.1| PREDICTED: glutamate receptor 1-like [*Bemisia tabaci*]	0.0	94.97
*AspiIR13*	Cluster-11154.0	1051		XP_018917922.1| PREDICTED: uncharacterized protein LOC109044571 isoform X1 [*Bemisia tabaci*]	0.0	85.46
*AspiNmdar1*	Cluster-17909.33013	981		XP_018899297.1| PREDICTED: glutamate [NMDA] receptor subunit 1 isoform X1 [*Bemisia tabaci*]	0.0	96.74

**FIGURE 3:**
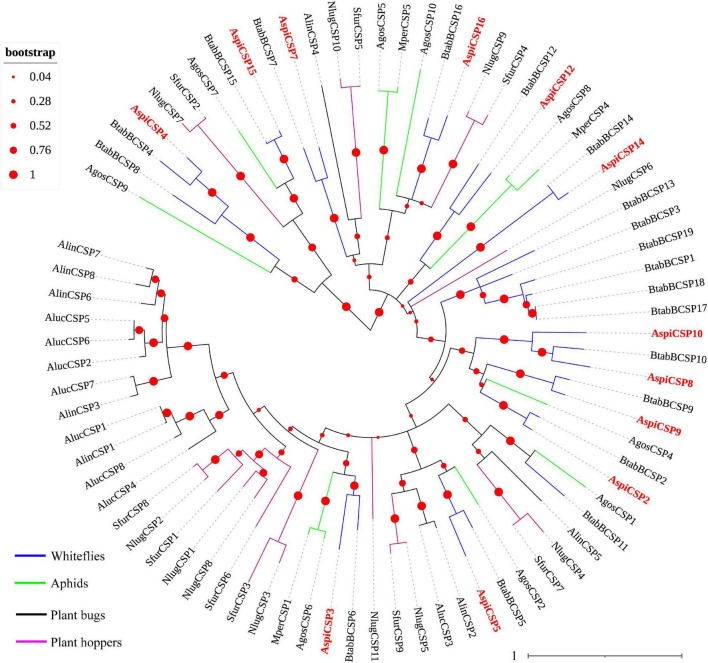
Phylogenetic analysis of putative chemosensory proteins (CSPs) of *A. spiniferus*. The phylogenetic tree was built using CSP sequences from whitefly species (Btab, *B. tabaci*; Aspi, *A. spiniferus*), aphid species (Apis, *A. pisum*; Agos, *A. gossypii*), plant hoppers (Sfur, *S. furcifera*; Nlug, *N. lugens*) and plant bugs (Aluc, *A. lucorum*; Alin, *A. lineolatus*).

We identified 12 candidate CSPs in *A. spiniferus* (Table 3) and the number of putative CSPs identified was also lower than that in *B. tabaci* (19 CSPs). Of the 12 putative CSPs, all of them had full-length ORFs, and only *AspiCSP12* and *AspiCSP15* without signal peptide. To analyze the relationship between the CSPs in the different species, a phylogenetic tree was constructed and is presented in Figure 3, which includes the identified CSPs from whiteflies (*A. spiniferus* and *B. tabaci*), aphids (*A. gossypii* and *M. persicae*), plant bugs (*A. lucorum* and *A. lineolatus*) and plant hoppers (*N. lugens* and *S. furcifera*). In the phylogenetic tree, all of the identified CSPs were clustered with CSPs in *B. tabaci* (Figure 3).

**FIGURE 4:**
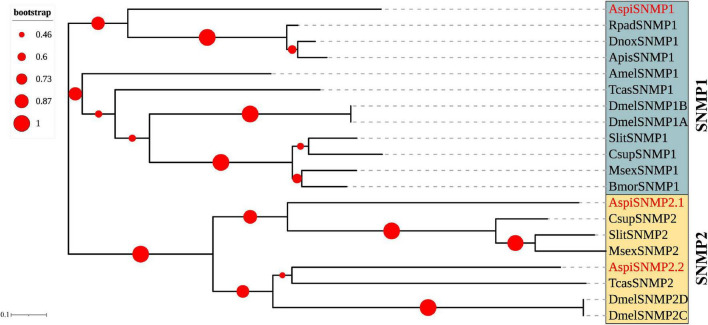
Phylogenetic analysis of putative sensory neuron membrane proteins of *A. spiniferus*.

Interestingly, there were three SNMPs identified in *A. spiniferus* that were significantly different from other Hemipteran insects (Table 3). The best hits by homology search in NCBI of these SNMPs were SNMPs from *B. tabaci* (Table 3). The phylogenetic tree showed that there were two distinct cluster SNMP1 (*AspiSNMP1*) and SNMP2 (*AspiSNMP2.1* and *AspiSNMP2.2*; Figure 4).

**FIGURE 5:**
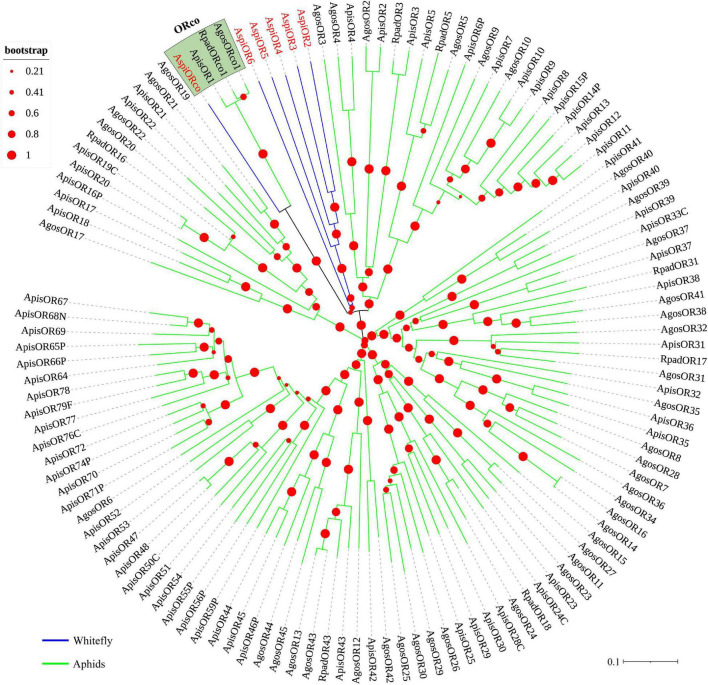
Phylogenetic analysis of putative odorant receptors (ORs) of *A. spiniferus*. The phylogenetic tree was built using OR sequences from whitefly specie (Aspi, *A. spiniferus*) and aphid species (Apis, *A. pisum*; Rpad, *R. padi*; Agos, *A. gossypii*).

We identified transcripts encoding six putative ORs (Table 3). Among these candidate ORs, *AspiORco*, *AspiOR2*, and *AspiOR3* likely represented full-length genes, encoding proteins made up of more than 400 amino acids (Table 3). In the phylogenetic tree, *AspiORco*, *AgosOrco1*, *RapdOrco1*, and *ApisOR1* were clustered in a specific subgroup called odorant co-receptor (Orco) with four transmembrane domains (Figure 5 and Supplementary Table S4). Rest of these identified ORs was clustered in a specific subgroup (Figure 5).

**FIGURE 6:**
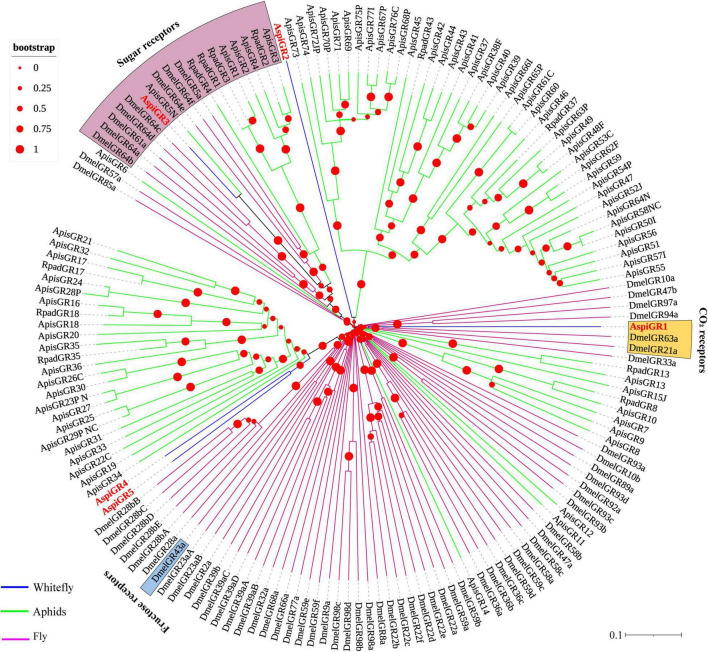
Phylogenetic analysis of putative gustatory receptors (GRs) of *A. spiniferus*. The phylogenetic tree was built using GR sequences from whitefly specie (Aspi, *A. spiniferus*), aphid species (Apis, *A. pisum*; Rpad, *R. padi*; Agos, *A. gossypii*) and fly (*Drosophila melanogaster*).

For GRs, in this study, we identified eight candidate GRs from the transcriptome of *A. spiniferus* (Table 3). A phylogenetic tree was constructed with sequences from whitefly (*A. spiniferus*), aphids (*A. pisum* and *R. padi*) and fly (*D. melanogaster*). *AspiGR14* was clustered with *DmelGR63a* and *DmelGR21a* as a CO_2_ receptor, while *AspiGR3* were found in a clade with sugar receptors, which included GRs identified from *D. melanogaster*, *A. pisum*, and *R. padi* (Figure 6).

**FIGURE 7:**
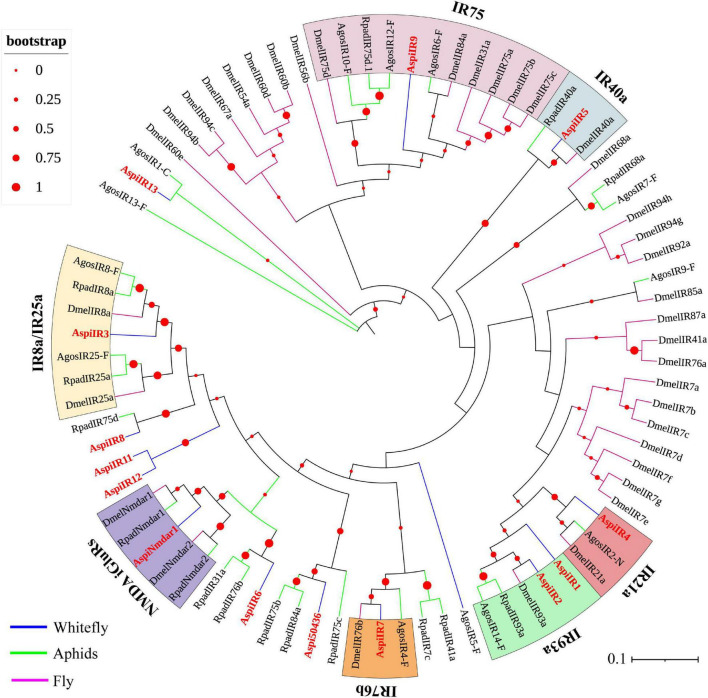
Phylogenetic analysis of putative ionotropic receptors (IRs) of *A. spiniferus*. The phylogenetic tree was built using IR sequences from whitefly specie (Aspi, *A. spiniferus*), aphid species (Apis, *A. pisum*; Rpad, *R. padi*; Agos, *A. gossypii*) and fly (*Drosophila melanogaster*).

Fourteen putative IRs were identified from the transcriptome of *A. spiniferus* (Table 3). Among them, only *AspiIR2*, *AspiIR5*, and *AspiIR8* were found to be a part of the full-length gene. The E-values for *AspiIR3*, *AspiIR4*, *AspiIR6*, *AspiIR7*, *AspiIR8*, *AspiIR9*, *AspiIR10*, *AspiIR11*, *AspiIR12*, *AspiIR13*, and *AspiNmdar1* were zero as compared to the amino acid sequences of these genes in *B. tabaci* (Table 3). In the phylogenetic tree, almost all of these IRs were clustered in a known group, such as IR8a/IR25a (*AspiIR3*), IR21a (*AspiIR4*), IR40a (*AspiIR5*), IR75 (*AspiIR9*), IR76b (*AspiIR7*), IR93a (*AspiIR1* and *AspiIR2*), and NMDA iGluRs (*AspiNmdar1*) (Figure 7).

### Expression Profiles of Chemosensory Genes

**FIGURE 8:**
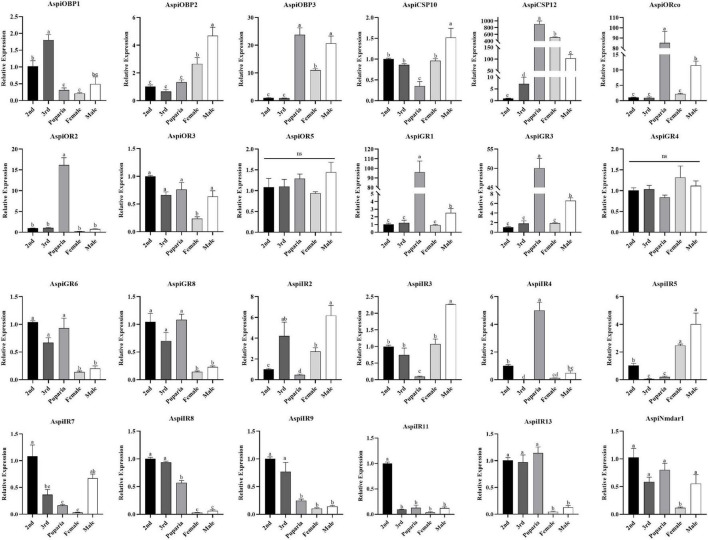
Quantitative real-time polymerase chain reaction (qPCR)-based expression profiling of selected chemosensory genes in different developmental stages of *A. spiniferus*.

Expression results of these selected chemosensory genes in different developmental stages showed that *AspiOBP1* and *AspiIR9* were more strongly expressed in nymphs than that in puparia and adults whereas expression of *AspiOBP3* and *AspiCSP12* in puparia and adults were significantly higher than that in nymphs (Figure 8). Surprisingly, *AspiORco*, *AspiOR2*, *AspiGR1*, *AspiGR3*, and *AspiIR4* showed highest expression profiles in puparia among the developmental stages (Figure 8). On the contrary, *AspiCSP10*, *AspiIR2*, and *AspiIR3* had the lowest expression in puparia. The expression of *AspiOBP2* and *AspiIR5* were significantly higher than that in nymphs and puparia (Figure 8). *AspiGR6*, *AspiGR8*, *AspiIR8*, and *AspiIR13* presented a higher expression in nymphs and puparia than that in adults (Figure 8). The expression of *AspiIR11* in second instar was significantly higher than other developmental stages (Figure 8).

**FIGURE 9:**
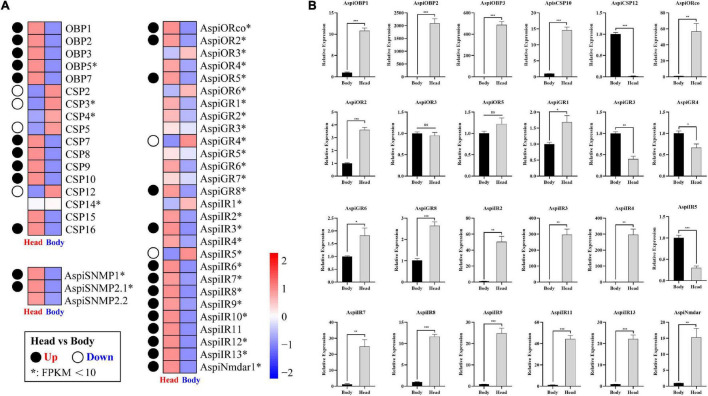
Expression profiles of chemosensory genes in different tissues. **(A)** Heatmap of chemosensory genes in the antennal transcriptome. Significance means an absolute value of log_2_Ratio ≥ 1 and FDR < 0.05. **(B)** Validation of selected chemosensory genes in different tissues by qPCR.

Based on the transcriptome results, we found that all of OBPs and SNMPs, and major of ORs and IRs were more considerably expressed in head than in bodies (Figure 9A). Meanwhile, only five of 12 CSPs were predominately expressed in heads, and four of 12 CSPs highly expressed in bodies (Figure 9A). In addition, there were only two of eight GRs showed significant tissue-specific expression patterns (Figure 9A). qPCR validation of selected chemosensory genes showed that expressions of *AspiOBP1*, *AspiOBP2*, *AspiOBP3*, *AspiCSP10*, *AspiORco*, *AspiOR2*, *AspiGR1*, *AspiGR6*, *AspiGR8*, *AspiNmdar1*, *AspiIR2*, *AspiIR3*, *AspiIR4*, *AspiIR7*, *AspiIR8*, *AspiIR9*, *AspiIR11*, and *AspiIR13* in heads were significantly higher than that in bodies, while *AspiCSP12*, *AspiGR3*, *AspiGR4*, and *AspiIR5* were predominantly expressed in bodies (Figure 9B). There was no difference of the expression of *ApisOR3* and *AspiOR5* between heads and bodies (Figure 9B).

## Discussion

Insects have a complex chemosensory system that accurately perceives external chemicals and plays a pivotal role in many insect life activities. Several studies have been conducted to understand the structure and function of the chemosensory system in different insect species, however, the chemosensory system in the orange spiny whitefly, *A. spiniferus* has not been investigated yet. The present study is the first report identifying the various types and distribution of the sensilla on the adult male and female antenna of *A. spiniferus.* Consistent with the results of two cryptic *B. tabaci* specie, length of male antenna was significantly longer than that of females, which was caused by the obviously smaller bodies of male *A. spiniferus* (Zhang et al., 2015). Furthermore, there was no differences in the composition and number of antennal sensilla between males and females. Contrary with that, in two cryptic *B. tabaci* specie, males had more chaetae sensilla (7) than females (5) (Zhang et al., 2015). Interestingly, in *A. spiniferus*, distribution of chaetae sensilla between males and females was different. In females, chaetae sensilla was observed in scape (1) and pedicel (6), while in males chaetae sensilla was found in pedicel (5) and flagellum (2). Differences of the distribution of chaetae sensilla might be involved in the different behaviors between males and females of *A. spiniferus*.

In this study, we systematically identified and chemosensory genes in *A. spiniferus* via transcriptomic analyses. A total of 48 candidate chemosensory genes including 5 OBPs, 12 CSPs, 3 SNMPs, 6 ORs, 8 GRs, and 14 IRs were predicted. The number of identified chemosensory receptors was close to *B. tabaci* that contains 9 OBPs, 18 CSPs, 7 ORs, and 17 GRs, but significantly lower than that in other hemipterans (*A. pisum*: 79 ORs, 77 GRs, 15 OBPs, and 1 SNMP; *A. gossypii*: 45 ORs, 14 IRs, 9 OBPs, 9 CSPs, and 1 SNMPs) (Chen et al., 2016; Wang et al., 2017; Xie et al., 2017; Zeng et al., 2019). However, the total number of OBPs and CSPs in whiteflies showed no contractions or expansion when compared with other hemipteran insects (Zeng et al., 2019). The reduction of numbers of ORs and GRs in whiteflies might result from their polyphagia and strong detoxification systems (Chen et al., 2016; Xie et al., 2017). Thereby, less ORs and GRs are enough for them to find their suitable host plants. In addition, as the number of IRs was similar with other insects, we speculated that IRs may work as ORs and GRs. Thus, the functional investigations about ORs, GRs and IRs are needed to figure out the reason of this phenomenon.

In this study, we found that all of these five OBPs were predominately expressed in the head. Expression of *AspiOBP1*, *AspiOBP2*, and *AspiOBP3* across developmental stages showed that *AspiOBP1* was more highly expressed in nymphs than that in pupae and adults whereas the expression of *AspiOBP3* in pupae and adults was significantly higher than that in nymphs. *AspiOBP2* was abundantly expressed in adults. Similarly, in *Sitophilus zeamais*, *SzeaOBP1* showed highest expression at larval stage, while the expression of *SzeaOBP28* at pupae and adult stage was significantly higher than that at larval stage (Zhang Y. et al., 2019). Furthermore, *SzeaOBP1* showed broader binding affinity for plant volatile compounds than *SzeaOBP28* (Zhang Y. et al., 2019). Silencing *SzeaOBP1* reduced the preference of *S. zeamais* to its preferred volatiles (Zhang Y. et al., 2019). In *B. tabaci*, *BtabOBP1*, *BtabOBP2*, *BtabOBP3*, *BtabOBP4*, *BtabOBP7*, and *BtabOBP8* were highly expressed in heads whereas *BtabOBP5* predominately expressed in legs and wings. *BtabOBP1*, *BtabOBP3*, and *BtabOBP4* have been demonstrated to bind oviposition repellent volatile, β-ionone and various volatiles to its specific chemosensory receptor (Li et al., 2019; Wang et al., 2020). Knockdown of *BtabOBP3* in *B. tabaci* by RNAi resulted in a reverse olfactory behavior to β-ionone (Wang et al., 2020). Furthermore, silencing *BtabOBP3* also reduced the preference of *B. tabaci* on ToCV-infected tomato plants and the ToCV transmission rate of *B. tabaci* (Shi et al., 2019). Besides the function of odorants perception, OBPs also have been found to been involved in other insect physiological process. For example, the adult *A. lineolatus* head predominately expressed *AlinOBP14* showed a pronounced binding affinity for insect juvenile hormone III (Sun et al., 2019). In *N. lugens*, knockdown of the gene for *NlugOBP3* not only reduced the response rate to seeding volatiles but also resulted in strikingly high nymph mortality (He et al., 2011).

Unlike the expression of OBPs is focused in the antennae or other olfactory sensilla in most insects, CSPs were found to be broadly expressed in various tissues including antennae, wings, legs and abdomen (Hua et al., 2013; Yang et al., 2014). For example, *AlucCSP2* and *AlucCSP3* of *A. lucorum* were specifically expressed in female wings, and showed high binding affinities with cotton secondary metabolites including gossypol, tannings, quercetin and rutin hydrate (Hua et al., 2013). In *N. lugens*, none of CSPs was predominately expressed in antennae (Yang et al., 2014). Leg highly expressed *CSP3* and *CSP8* of *N. lugens* strongly bound to plant volatiles (Waris et al., 2018). Injected with *dsNlugCSP8* significantly reduced the attractive responses of *N. lugens* to nerolidol and hexanal (Waris et al., 2018). Furthermore, CSPs are also known to be involved in insecticide resistance. Overexpressing *AgosCSP5* on *Drosophila* files showed higher resistance and survival in response to imidacloprid and cypermethrin than control flies (Li et al., 2021). In *B. tabaci* and *Ophraella communa*, *BtabCSP11* and *OcomCSP12* were strongly expressed in the female abdomen and ovary respectively, and both of them are involved in the reproduction (Ma et al., 2019; Zeng et al., 2020). Consistent with these results, in this work, we found *AspiCSP12* was specifically expressed in female bodies. Additionally, *AspiCSP12* also showed higher expression levels at puparia and adult stages than that in nymphs. However, AspiCSP10 showed lowest transcript abundance in puparia, and there was no difference of this gene between nymphs and adults. All of these results indicate that *AspiCSP12* might have other physiological functions rather than just being involved in odorant perception.

Recently, more research has been focused on the function of three chemosensory receptor types: ORs, GRs, and IRs (Zhang R.B. et al., 2017; Yang et al., 2020, 2021). In this study, we identified six ORs in the *A. spiniferus* transcriptome. Among of these ORs, *AspiORco*, *AspiOR2*, and *AspiOR5* were predominately expressed in head whereas other ORs showed broadly expressed in heads and bodies. Furthermore, the expression of *ORco* was significantly higher in male and puparia than that in other stages. Similar results were observed in aphids, and knockdown of *SaveORco* in *S. avenae* disrupted its response to plant volatiles and the aphid alarm pheromone, (E)-β-farnesene (Fan et al., 2015; Kang et al., 2018). Besides the *ORco*, in *A. pisum*, *ApisOR5* is known as an essential receptor for its alarm pheromone E-β-farnesene, and *ApisOR4* is involved in the recognition of plant volatiles (Zhang R.B. et al., 2017; Zhang R.B. et al., 2019). Silencing *CquiOR114/117* in female *Culex quinquefasciatus* significantly impaired the blood feeding behavior (Wu et al., 2020). All of these results indicated that ORs especially for *AspiORco*, *AspiOR2*, and *AspiOR5* in *A. spiniferus* might be involved in the plant volatiles perception.

As phloem-feeding insects, whiteflies can be affected and even killed by the phytochemicals in plant phloem sap, such as amino acids, sugars and other metabolites (Cui et al., 2017; Hasanuzzaman et al., 2018; Xia et al., 2021). To cope with this, whiteflies assess the suitability of a potential host plant and select the best plant as well as the best feeding region on the plant (Döring, 2014; Cui et al., 2017; Hu and Tsai, 2020). For example, the content of phenolic glycosides and amino acids in cottonwood leaves varies with their developmental stage and its host aphid *Chaitophorous populicola* was able to detect the difference and to track the preferred leaf stages to optimize its feeding (Gould et al., 2007). In *M. persicae*, the high glutamine concentration stimulated the feeding behavior (Cao et al., 2017). Overexpressing *PrapGR28* from *Pieris rapae* in *Drosophila* files resulted in a strong preference to the food with sinigrin whereas the wild-type (WT) files showed avoidance (Yang et al., 2021). In *B. mori*, *BmorGR66* mutant showed no significant feeding preference for both mulberry leaves and Mongolian oak leaves, while WT *B. mori* did not eat Mongolian oak leaves (Zhang Z.J. et al., 2019). Apart from the GRs, some IRs are also expressed in gustatory organs and are involved in gustation perception (Zhang et al., 2021). For example, IRs expressed in *D. melanogaster* leg sensilla also showed a response to food components such as sugar, salts, polyamines and bitter compounds (Ling et al., 2014; Hussain et al., 2016). In *H. armigera*, knockout of *IR8a* reduced the EAG responses and trend behavior to acetic acid (Zhang et al., 2021). Additionally, IR8a was found to be essential to detect human odors and water detection in *Aedes aegypti* (Raji et al., 2019a,b). Meanwhile, *IR40a*, *IR93a* and *IR25a* mediate the humidity preference in *D. melanogaster* (Enjin et al., 2016). Furthermore, IR25a and IR93a are also involved in the detection of temperature. Interestingly, *Nmdars* have been implicated in associative learning and memory in *D. melanogaster* and are essential factors for male offspring production in *Diploptera punctata* (Xia et al., 2005; Huang et al., 2015). Based on the *A. spiniferus* transcriptome, putative receptors for sugar in GRs, *IR8a*, *IR40a*, *IR93a*, and *Nmdars* were predicted according to the phylogenetic analyses. Almost all of GRs except *AspiGR4* were widely expressed in heads and bodies whilst major of IRs exhibited higher expression in heads than that in bodies. Expressions of selected GRs and IRs showed that the highest expression of *AspiGR1*, *AspiGR3*, and *AspiIR4* were at puparia. *AspiIR5*, which was clustered as *IR40a*, had higher expression in adults than that in nymphs and puparia. All of these results indicated that GRs and IRs in *A. spiniferus* might be involved in various biological processes and have critical roles in the survival.

Taken together, in this study, we systemically identified six types of sensilla on antennae of including grooved surface trichodea sensilla, chaetae sensilla, microtrichia sensilla, coeloconic sensilla, basiconic sensilla and finger-like sensilla via SEM and a total of 48 chemosensory genes in *A. spiniferus* including 5 OBPs, 12 CSPs, 3 SNMPs, 6 ORs, 8 GRs, and 14 IRs. Based on the transcriptome data, we developed a tissue-specific expression profile for each of the identified chemosensory genes in *A. spiniferus*, which might reveal an initial prediction of these genes’ function. Furthermore, we also analyzed the expression of 24 selected chemosensory genes across the developmental stages. In summary, this study not only provides strong background information and initial understanding on the chemosensory systems in host reception of this polyphagous insect but also provides extensive potential targets for pest control. In future, the further investigation about which gene is the key factor of plant perception and the suitable pest management target is needed to be done.

## Data Availability Statement

The datasets presented in the study are deposited in the Genbank SRA database, accession number PRJNA792195. Our data has now been released in NCBI (https://www.ncbi.nlm.nih.gov/bioproject/PRJNA792195).

## Author Contributions

Z-WK designed the research, analyzed transcriptome data, constructed phylogenetic trees, and wrote the manuscript. Z-WK, Y-QG, and M-YL collected sample. Y-QG, Z-ZC, and C-YS performed SEM experiment. Y-QG, Z-ZC, and F-HL conducted qPCR and analyzed qPCR results. YD, H-PZ, Y-YX, and CQ edited the manuscript. Y-YX and Z-WK revised the manuscript. All authors contributed to the article and approved the submitted version.

## Conflict of Interest

The authors declare that the research was conducted in the absence of any commercial or financial relationships that could be construed as a potential conflict of interest.

## Publisher’s Note

All claims expressed in this article are solely those of the authors and do not necessarily represent those of their affiliated organizations, or those of the publisher, the editors and the reviewers. Any product that may be evaluated in this article, or claim that may be made by its manufacturer, is not guaranteed or endorsed by the publisher.

## Footnotes

1
https://www.eppo.int/ACTIVITIES/plant_quarantine/A2_list

2
http://www.ncbi.nlm.nih.gov/gorf/gorf.html

3
http://www.cbs.dtu.dk/services/SignalP/

4
http://smart.emblheidelberg.de/

5
https://services.healthtech.dtu.dk/service.php?TMHMM-2.0

## Supplementary Material

Supplementary Figure S1 Homology analyses results. The BLASTx annotations of *A. spiniferus* antenna transcripts. **(A)** E-value distribution. **(B)** Similarity distribution. **(C)** Species distribution.

Supplementary Figure S2 Functional annotation of *A. spiniferus* transcripts based on gene ontology (GO) categorization.

Supplementary Figure S3 Alignment of OBPs **(A)** and CSPs **(B)** of *A. spiniferus*.

Supplementary Table S1 Amino acid sequences of chemosensory genes from *A. spiniferus* and other insects that used for phylogenetic analyses.

Supplementary Table S2 Primers used in this work.

Supplementary Table S3 Summary of *A. spiniferus* transcriptome annotation.

Supplementary Table S4 Transmembrane domains in ORs, GRs and IRs.
