# Supplementary material for: Characterization of Antennal Chemosensilla and Associated Chemosensory Genes in the Orange Spiny Whitefly, Aleurocanthus spiniferus (Quaintanca)
Source: Front Physiol. 2022 Feb 28;13:847895. doi: 10.3389/fphys.2022.847895 (PMC8920487; doi:10.3389/fphys.2022.847895)
Supplement: Supplementary Figure S1 — Homology analyses results. The BLASTx annotations of A. spiniferus antenna transcripts. (A) E-value distribution. (B) Similarity distribution. (C) Species distribution. [file Data_Sheet_1.docx]

Supplementary materials

Figure S1


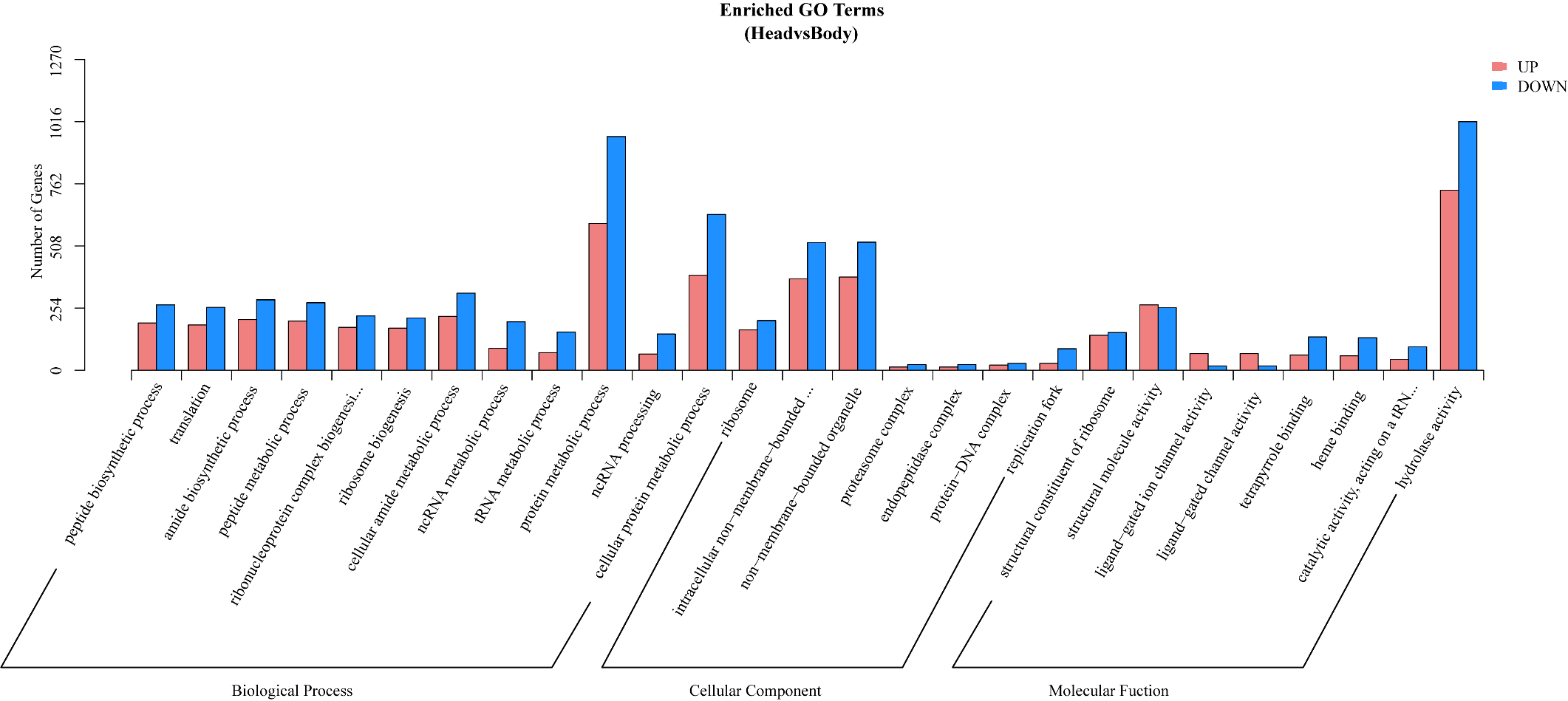


Figure S2


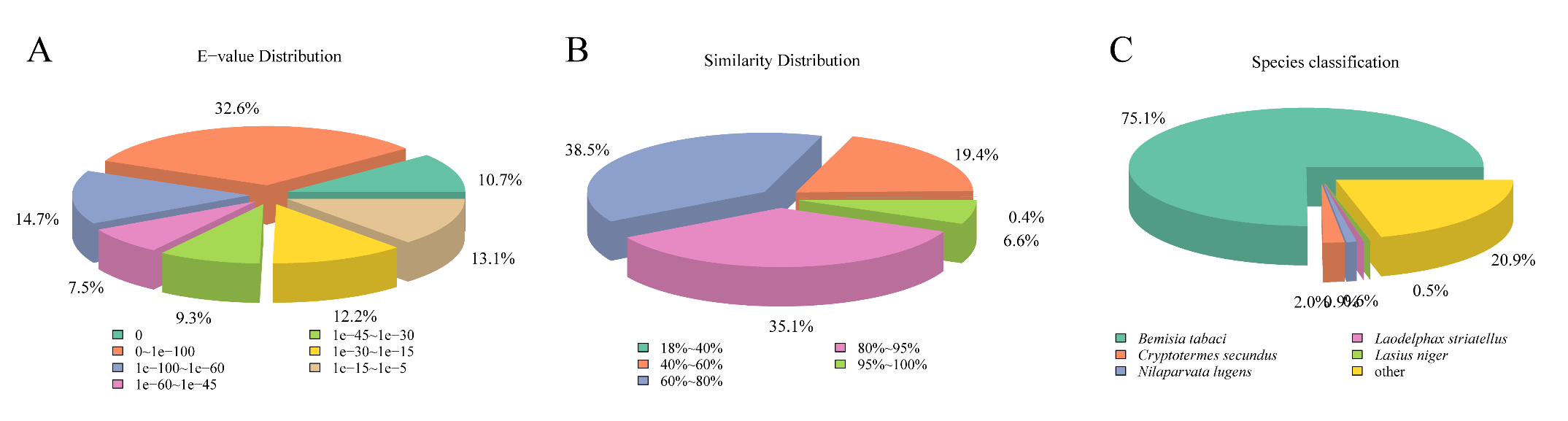


**TABLE S1 |** Amino acid sequences of chemosensory genes from *A. spiniferus* and other insects that used for phylogenetic analyses.

| **Amino acid sequences** |
| --- |
| **OBPs** |
| >AspiOBP1  MDLTAVLVFVSFVAQSTIHFTHAVTHEQIDQLAVACSGKKEDIDVMKQYLVPSTDSGKCLMKCLTSYYKMFDENGQYNRENTEKSMKASWPNKPAHDVTVIAKHCESKMSDAPKSHYNTCEMSYNTMKCVNDKAWELGWFTKS  >AspiOBP2  MATRVYFSFLLVIFFVSQGCRAETSTVDSVEEDDEPLKFHPRFARAAEEKSKEESGECPPPHHHGGGPGPRSQKPPPECCKGLPRTCNPAEDQKAWETCLAKLPRKPPTTSEPTTVANGGKPTGGPMFGIKKPCLIECVYNETGLLTPEMKLQEAKVSQKLTDKLKDTKFGSMAPSVITKCFTTAKANVPPNGECKSGAAEFAKCVNRELFMNCPTENWDNNEECQKIKEKLTKCPNMPVPMGPPPKP  >AspiOBP5  MAPSLHAFGFVSAASLGVIALLAAFVAAAPNLQIQGSFANCQAPATAPQKLERVVEVCQNEIKFALLQEALTLLGLGGSDTDDEEEETKISSPRNRTTTAATTAKSIIAPTKSRPKRDSSFSQEERRVAGCLLQCVYRKVKAVDDTGFPQSEGMVRLYSEGVQDNNYYSATVQAVERCVSYAQQLKQKQPHQKFASGQTCDLAYDMFECISEKIEKACGGAAP  >AspiOBP3  MGFYALGFAVLGLAALQVAGFDGAEADGHYVGRRGWLRMKREDDESDAAPKAERPDPKCREYDMMIITSATDTMEECCKCSDCKLSGLKDNVIEQCEVDLQKEAEVQMGVSGNSTKGAPMPMALKSESGSSNSSSANETKTSSEEDAASVKVYGECLTECVFRASGYLDSNLQLNESAINKEMDAGIKDPNWAPIAQKTFKKCMEHVKQVSPTGKCKSKSDALVVCLARERYLSCPEKAWNQNNAECQKIKEKLVKCPTLAPPPF  >AspiOBP7  MDACVVQCIFRQMEMLTEDARPDKGSVISVMTQRIRDPELKEFIQESIEECFDIIESDAEGGKCEYSKNFAMCLEEKGRRNCEDWEEAAQINRFKGIQNAMGPNYNYNAPNYNNNNFDSKIPGNRPNSFTNYNGK  >BtabBOBP1  MMDLKAILLSLFVVTLSLFGITHAVTHAEMDQLAVACNGQKADIDVMKQWRVPSSDSGKCLMKCLTDYYKMFDSEGKYSLKNTEDSLYKAWSEKPKEHMPIIAQHCDNMMRSAPKENLGSCQMSYDTMKCINEKAWELGWFN  >BtabBOBP2  MAMRALIGLFLVAAFFVQRCKAESTTPIPMPEEEDELPARFRSRVVRSASEEKSKEDSSEECGPPQHHHPHPHKPPPECCKGLPRPCNPTEDKKAWDTCVNKLSKKNPTTPQPTTQANGGKPGHGPMHGIDKQCMVECVYNETGLLTPDMKLQEAKISQKLTDKLKDTKFASMAPSVISKCFEKAKNAKDPKECKSGAGEFSKCVNRELFMNCPTESWTNSEECQKMKEKLTKCPNMPVPMSPPPKP  >BtabBOBP3  MSASTARCVGVVAAVFVLTIIAAVAAYDFEDAAFNARLIGDLDEYAVSSARNRRDADLDVDEFFQCKQRNQKVCCGKSSLFKNFGDKDKTAGKACYREIAEKLKRKDDLDEDLMDIFSCEQIKQMKKKHFCIHECIGKRKNLLNADGSINVLNMRTYLKKELFTEEWQQKVGDKAMEKCFNEKYVSSWPDSDSSDVKCNPISVQFQHCLWKQIELNCPDSEWKDTRRCNKIKEYLRKQELSQKNKISKN  >BtabBOBP4  MKSSIAVLAVLSVFACVQAQDAKQKVAAVAEKCKTEVRGGHDVLKIVGTSELPKTEEQRCFLECVYKNLQLIVDDKFAEPGAKRLMMMKYGSNPDQLKIAQTEIETCAKEVKPTAGMKCSLAHNIRQCFAKEGQKHNFYIKA  >BtabBOBP5  MSSLRPVQVTVLLGAALLVGLAAAAPNIQLQGSFANCKAPSTAPQKLERVVEVCQNEIKFALLQEALNLLGLGIADDDSSEEEEPVKESKNRTVTIAPAKSRPKRDSSFSQEERRVAGCLLQCVYRKVKAVDDTGFPQAEGMVRLYSEGVQDNNYYSATVQAVERCVSFAQALKLKQPNQKFASGQTCDLAYDMFECISEKIERACGGAAP  >BtabBOBP6  MQGEVCFALLVVCLLHVTFIPDVETAVSEQMIKQMKDMHKKTCMARSGVKKEGLAKFMEEGVNDDPAFKKYTLCMLKNMQGFKNGKIAIKDIENQAKAMLPPPLRDAILDATSKCSNTGGDTPEDITYNFSKCSHKANVKSVMII  >BtabBOBP7  MGGGGRPWSQNTQDSGRGGGNGGGRHQSNQYGNQNTQDSGRGGGNGGGRHQSNQYGNQNNDRYCSSGYQNSDRDRPRFSSSGSGGGGGGGNDDDFYTKTQSEQRFRGDGGGGGRTRPGQYRVKVQYGSGQGGGPDSRAYPLGRKRRATKIISNNAKNATGRNPTSSETGKTSLLEELDACVVQCIFRQMEMLTEDARPDKNSVISVMTQRIRDPELKEFIQESIEECFDIIESDAEGGKCEYSKNFAMCLEEKGRRNCEDWEEAAQINRFKGIQSGMSPPDTTNNYNPPNFNNFNSNNQGNRQNAFYNYNGK  >BtabBOBP8  MNNLMVFSILLYLQYLLAVEADQGNQGHQGQQGNQSEDQEKVKAIYQKCQQESMAEEKDLDNFKKMEMPSSEKGKCMMACLMREAKIIVNKRFSKDGAMALAQRYYSTQPQNMDKAKQVIVACDKQVEHERNECNIAGKLAECVVTEAQRVGLTSVPKG  >AglyOBP2  MKVSAATAVLVALVATVQSSDPCNISTCYKSGTTKPPTTVTPTRLPVQSSSTPTSHQQTTYAKDHVHSSTATKSGVNTTATTTSGASVNGTERTTVVKSSSGVAGNVTTPKPTMTDGHLALKQKLNTIAVKCKDELHAPQEIMALVSNTVVPQNEQQRCYLECVYKNLNLIKNNKFSVDDGKAMAKIRFANQPEEHKKAVTIIETCEKEAIIDPKTTEKCAAGRVIRNCFVKNGEKINFFPKA  >AglyOBP3  MISSTFYTSLMFGIVMLISCSFGRFTTEQIDHYGKACNATEDDLVVVKSYKVPTSDTGKCLMKCMISKLGLLNDDGSYNKTGMEAGLKKYWSEWSTDTIESINNKCYEEALLVSKDIIATCNYAYVVMACLNKQLDLDKST  >AglyOBP4  MRGNYSLVVFLLFGFGLLEIYCQKQETSGKCRAPDKAPLNLEIIINICQEEIKSALLQEALDILNDGTLEQNTPSYSRSKRDADEDLSNEERRVAGCLLQCVYKKVKAVDETGFPVVDGLMKLYNEGVQDRNYYMATLSAVRHCISIAQQLKQQQPSKSFDDGQTCDLAYEMFECVSEKIEENCGVENKSNN  >AglyOBP5  MKRFGDKDKVAADECYAQVAEKFATVTATTPKQDLFSGEAVKITKKKQFCLHECIGKKNKLLTEDGSLNKTFIADYAMKSVFKEQWQKQIGQKALDKCLEETYIPWPAEETENKCNPVYVQFQHCLWLEYESNCPDNKIKLTKKCEKTRNRYRMQKSTSN  >AglyOBP6  MQKVVFLCIFAIICQTVFTVGFERTWILRQKRVTNDDECRTLIPSSEKKLPTCCQMPNILPGLDNAWEVCFEKFKQFKDKHATKEYKEMAHGNEPPCLFQCVFMQSGLTTSDGKVNEDAVIKKMAEGMDNDEKWKSIWRNTFNKCLNDVKQEDKEQIKMTNTPTGRLMKCFLRDLYMNRPKNVWVESSECSNLKDLVEKCPKMPPPVFKSPPKLI  >AglyOBP7  MVARKRMYMLPATVLLAVVAATILKDSDAYLSEEAIKKTQKMLKNVCSKKHSVEEEVFTDIKKGIFPENNNNIKCYFACNFRTMQMVNQKGILDKKMFKDKMTMLAPPNVLAILLPPIEQCIGNDKDTEICRSSYNFIKCAHRVDPKSLEFLPL  >AglyOBP8  MFAFKVACLCLSVAVVFGENNQQNSNDRSASIFQSCISETKLSGDALKGFRSMSIPKTQAEKCMMGCLMRKVNVINNGKFSVEEATKVAQKYYGTNETMMKKAKDLIDVCAKKAQSTTEECALAGIVTTCIVEEAQKAGLTGGPGSRSKRTVSPKFRHSIV  >AglyOBP9  MIIKKTLLVSGFVLFGCMFSINKAADDADAKDKELMSKLITVAFKCFKDADWGTCGEMITTKYDITQAKYKQCTCHMACAGEDLGLINSNGQPEPAKFLEYVKRINNSVIKSQLQHIYDKCQNVKGTEKCDLAEQFAICAFKESPEMKERVTKLIEMLVKMKPKSK  >AglyOBP10  MEHLRGTNVVFAIVMALLVVQSSTRPQPDELDDIKKTLYNACSEKFPLTEEIKNNVKNSIVIDDQNFKCFLRCCFDEMSLIDEDGIIDGESLAAMAVDKIKPVAEKIVHDCLPAGKQEKQDGCEASFKFFSCGIKLNPLTIELLPLQ  >AglyOBP11  MISSTFYTSLLFGIAMLISCSFGRFTTEQIDHYGKACNATEDDLVIVKSYKVPTSDTGKCLMKCMISKLGLLNDDGSYNKTGMEAGLKKYWSEWSTDTIESINNKCYEEGGTICFILNRFVT  >AfabOBP2P  SDPCNISTCYQSGTTKPPTTVTPTRLPVQSSSTPTSHQQTTYAKDHVHSSTATKSGVNTTATTTSGASVNGTERTTVVKSSSGVAGNLTTPKPTMTEGHVALKQKLNTIAVKCKDELHAPQEIMALVSNTVVPQNEQQRCYLECVYKNLNLNKNNKFSVDDGKAMAKIRFANQPEEHKKAVTIIETCEKEAIIDPKTTEKCAAGRVIRNCFVKNGEKINFFPKA  >AfabOBP8P  ENNQQNSNDRSATIFQSCISETKLSGDALKGFRSMSIPKTQAEKCMMGCLMRKVNVINKGKFSVEEATKVAQKYYGTNETMMKKAKDLIDVCAKKAQSTTEECALAGIVTTCIVEEGQKVGLTGGPGGRSRRTVSPKFRRNSM  >AcraOBP2P  SDPCNISTCYKSGTTKPPTTVTPTRLPVQSSSTPTSHQQTTYAKDHVHSSTATKSGVNTTATTTSGASVNGTERTTVVKSSSGVAGNATTPKPTMTEGHVALKQKLNTIAVKCKDELHAPQEIMALVSNTVVPQNEQQRCYLECVYKNLNLIKNNKFSVDDGKAMAKIRFANQPEEHKKAVTIIETCEKEAIIDPKTTEKCAAGRVIRNCFVKNGEKINFFPKA  >BbraOBP3  MISSTFYITLLFGIAMIISCSYGRFTTDQIDYYGKACNASEDDLVVVKSYKVPSTETGKCLMKCMITKLGLLNDDGSYNKTGMEIGLKKYWSEWSTEKIEAINNKCYEEALLVSKEVIATCNYSYTVMACLNKQLDLDKST  >TsalOBP1P  ESDQVPMNSSAAVENCLLETNMTRDEFEDMLTSPNARELTILKSHAHKCMLGCVMRKNHIVNDGVVSKEVLSKYVLNFYGRPDYKRRLIIKDVEHIVDVCAKKVADESETDECELAATLVTCIVLEANKAGLVDDPARQI  >DplaOBP3  MISSTFYITSVFGIAMLISCGYGRFTTDQIDYYGKACNASEDDLVVVKSYKVPSTETGKCLMKCMITKLGLLNDDGSYNKTGMEAGLKKHWSEWSTEKIENINNKCYEEALLVSKEVVATCNYSYTVMACLNKQLDLDKST  >PsalOBP1P  ESDQVPMNSSAAVENCLLETNMTRDEFEDMLTSPNARELTILKSHAHKCMFGCVMRKNHIVNDGVVSKEVLSKYVLNFYGRPDYKRRLIIKDVEHIVDVCAKKVADESETDECELAATLVTCIVLEANKAGLVDDPARQI  >PsalOBP2P  SDPCNISTCYKSGTTKPPTTVTPTRLPVQSSSTPTSHPQTTYAKDHAHGSTTVKSGANVTATKDNKATVNGTTERPAVKSTTGAAGNATTLNSTMTEVHVALKQKLNTIAVKCKDELHAPQEIMALASNTVVPQNEQQRCYLECVYKNLNLIKNDKFSVDDGKTMAKIRFAKQPEEYKKAVTIIETCEKEAVIDPKTTEKCAAGRVIRNCFVKNGEKINFFPKA  >PsalOBP4P  QKQETSGKCRAPDKAPLNLEIIINTCQEEIKSALLQEALDILNDGNTEQNTQNQSNRSKRETEEDLTNEERRVAGCLLQCVYKKVKAVDETGFPVVDGLMKLYNEGVQDRNYYIATLSAVRHCISIAQQLKQQQPSKTFDDGQTCDLAYEMFECVSEKIEENCGVENKSNN  >PsalOBP9P  DDADAKDKELMAKLFGVALKCFKDADWGACGEMITTKYDITEPKYKQCTCQMACVGEDLGMINTKGEPEPAKFLEYVKRINNQSIKSQLQHIYDKCQNVKGADKCDLSEQFAICAFKESPALKERVSTLMEMLVKMKPKSK  >PsalOBP10P  STRPQPDELEEVKKSLYNACSSKFPLTEEIRNQAKNGILTEDPNLKCFLRCCFDEMSLIDEDGIIDGETLVAMSMDRIKLITEQAVHNCLKTTKQDGCEASFQFLSCGIKLNPLI  >LeryOBP3P  RFTTEQIDYYGKACNASEDDLAVVKSYKVPSTETGKCLMKCMITKLGLLNDDGSYNKTGMEIGLKKYWSEWSTEKIEAINNKCYEEALLVSKEVVATCNYSYTVMACLNKQLDLDKST  >LeryOBP7P  MVARKRMYNMLPTNVLLTIIAATVLNDCDAYLSEAAIKKTQQMLKSVCSKKYTVEEDVFTNIKKGIFPEDNNNIKCYFSCVFKTMQMINQKGSLDKKIFKEKMSMMAPPSVYNILLPAIEQCIGKDNGEELCQASYNFIKCAHHIDPKSLEFLPL  >MvicOBP1P  EIDQVPINSSAAVENCLLETNMTRDEFEDMLTSPNARELTILKSHAHKCMFGCVMRKNHIVNDGVVSKEVLSKYVLNFYGRPDYKRRLIIKDVEHIVDVCAKKVADESETDECELAATLVTCIVLEANKAGLVDDPARQI  >MvicOBP2P  SDPCNISTCYKSGTTKPPMSVTPTRLPVQSSSTPTSHPQTTYAKDHSHGSTTTKSGANATATTASGASVNGTERPAVVKSSAGVTGNLTTPKPTMTEGHVALKQKLNTIAVKCKDELHAPQEIMALVSNTVVPQNEQQRCYLECVYKNLNLIKNNKFSVEDGKAMAKIRFANQPDEHKKAVTIIETCEKEAVIDPKTTEKCAAGRVIRNCFVKNGEKINFFPKA  >MvicOBP5P  DAGHHRRGKELLDTEDSDFFRCKQASRKSCCGPENAMKRFGDKDKVAADECYAQVAEKFATVTATTPKQDLFSADAVKITKKKQFCLHECIGKKNHLLTEDGSLNKTFIADYAMKSVFKEQWQKPVGLKALEKCLEETYIPWPAEDKENVCNPVYVQFQHCLWLQYESNCPANKIKITKKCEKTRNRYRMQKSTSN  >MvicOBP8P  ENNQQNSNDRSATIFQSCISETKLSGDALKGFRSMSIPKTQAEKCMMGCLMRKVNVINKGKFSVEEATKVAQKYYGTNETMMKKAKDLIDVCAKKAQSTTEECALAGIVTTCIVEEAQKAGLSGGPGSRSRRTVSPKFRRNSM  >MvicOBP10P  STRPQPDELEEIKKTLYNACAGKFPITEEVKNNAKNSIFLDDQNFKCFLKCCLDEMSLIDDDGIIDGDSLKAMASDKIKPMXEQVVPNCLKNVQQDGLEAAFVFLRXGRG  >MdirOBP1P  EIDQVPINSSAAVENCLLETNMTRDEFEDMLTSPNARELTILKSHAHKCMFGCVMRKNHIVNDGVVSKEVLSKYVLNFYGRPDYKRRLIIKDVEHIVDVCAKKVADESETDECELAATLVTCIVLEANKAGLVDDPARQI  >MdirOBP2P  SDPCNISTCYKSGTTKPPMAVTPTRLPVQSSSTPTSHPQTTYAKDHVHGSTTIKSGANATATTASGASVNGTERPTVVKSSAGVIGNSTTPKPTMTEGHVALKQKLNTIAVKCKDELHAPQEIMALVSNTVVPQNEQQRCYLECVYKNLNLIKNNKFSVEDGKAMARIRFANQPEEHKKAVTIIETCEKEAIIDPKTTEKCAAGRVIRNCFVKNGEKINFFPKA  >MdirOBP3P  RFTTEQIDYYGKACNASEDDLVVVKSYKVPSTETGKCLMKCMITKLGLLNDDGSYNKTGMEAGLKKYWSEWSTEKIENINNKCYEEALLVSKEVVATCNYSYTVMACLNKQLDLDKST  >MdirOBP4P  QKQETSGKCRAPDKAPLNLEIIINICQEEIKSALLQEALDILNDGNLEQNTPASYSSRSKREADEDLTNEERRVAGCLLQCVYKKVKAVDETGFPVVDGLMKLYNEGVQDRNYYMATLSAVRHCISIAQQLKQQQPSKSFDDGQTCDLAYEMFECVSEKIEENCGVENKSNN  >MdirOBP5P  DAGHHRRGKELLDTEDSDFFRCKQASRKSCCGPENAMKRFGDKDKVAADECYAQVAEKFATVTATTPKQDLFSAEAVKITKKKQFCLHECIGKKNNLLTEDGSLNKTFIADYAMKSVFKEQWQKQVGQKALDKCLEETYIPWPAKNKENVCNPVYVQFQHCLWLQYESNCPANKIKITKKCEKTRNRYRMQKSTSN  >MdirOBP6P  PNILPNSNSTWAKCFETFKQFKDKPETKEYKEMAHGKEPPCLFQCIFVQSGLTTSDGKLNEDAITKKMSEGINNDEKWKSTWQNSLNKCFDDVKQEDKKQILIMNTPAGRLMKCFLRDMYMSCPKSVWVESSECLNMKDLVQKCPEMPPPVFKSPPKLI  >MdirOBP8P  ENNQQNSNDRSATIFQSCISETKLSGDALKGFRSMSIPKTQAEKCMMGCLMRKVNVINKGKFSVEEATKVAQKYYGTNGTMMKKAKDLIDVCAKKAQSTTEECALAGIVTTCIVEEAQKAGLSGGPGSRSRRTVSPKFRRNSM  >MperOBP3P  RFSTEQIDYYGKACNASEDDLVVVKSYKVPTTETGKCLMKCMITKLGLLNDDGSYNKTGMEAGLKKYWSEWSTEKIEAINNKCYEEALLVSKEVIATCNYSYTVMACLNKQLDLDKST  >MperOBP4P  QKQEPSGKCRAPDKAPLNLEIIINICQEEIKSALLQEALDILNDGNLEQNTPSYSSRSKREADEDLTNEERRVAGCLLQCVYKKVKAVDETGFPVVDGLMKLYNEGVQDRNYYMATLSAVRHCISIAQQLKQLQPSKSFDDGQTCDLAYEMFECVSEKIEENCGVENKSNN  >MperOBP6P  PNILPGLDSTWEKCYEKFIQFKDKPETKEYKEMSHGKEPPCLFQCIFMESGLTTNDGKLNEDAITKKMTEGINNDEKWKSTWKKSLDKCFDDVKQEDKKQILIMNTPAGRLMKCFLRDIYMNCPENVWVESSECLNVKNLVQKCPEMPPPVFQSAPKLI  >MperOBP7P  YLSEAAIKKTQQMLKTVCSKKHSVEEDVFTDIKKGIFPENNNNIKCYFACNFKTMQMINQKGTLDKKLFKDKMSMMAPPNIYNILLPAIEQCIGIDKGEELCQSSYNFIKCAHRVDPKSLEYLPL  >MperOBP8P  ENNQQNSSDRSATIFQSCIAETKLSGDALKGFRSMSIPKTQAEKCMMGCLMRKVNVINKGKFSVEEATKVAQKYYGTNETMMKKAKDLIDVCAKKAQSTTEECALAGIVTTCIVEEAQKAGLAGGPGSRSRRTVSPKFRRNSM  >NribOBP2P  SDPCNISTCYKSGTTKPPMTVTPTHLPVQSSSTPTSHPQTTYAKDHVHGSTTTKSGANATATTASGASVNGTERPAVVKSSAGVTGNFTTPKPTMTEGHVALKQKLNTIAVKCKDELHAPQEIMALVSNTVVPQNEQQRCYLECVYKNLNLIKNNKFSVEDGKAMARIRFANQPEEHKKAVTIIETCEKEAVIDPKTTEKCAAGRVIRNCFVKNGEKINFFPKA  >NribOBP3P  RFTTEQIDYYGKACNASEDDLVVVKSYKVPSTETGKCLMKCMITKLGLLNDDGSYNKTGMEAGLKKYWSEWSTEKIETINNKCYEEALLVSKEVVATCSKSHDRKACLNQDPDLDKST  >NribOBP5P  DAGHHRRGKELLDTEDSDFFRCKQASRKSCCARKNAMKRFGDKNKVAADECYAQVAEKFATVPATTHKQDLFSAEAVKITKKKQFCLHECIGKKNNLLTEDGSLNKTFIADYAMKSVFKEQWQKEVGQKALDKCLEETYIPWPAEDKENVCNPRYVQIQHCLWLLSRRNIPAHKSKITKKCEKTRNRYRMQKSTSN  >NribOBP7P  YLSEAAIKKTQHMLKTVCSKKHSVDEDVFTEIKKGIFPEDNNDIKCYFACNFKTMQLVNQKGYIDKKLFKDKMSIMAPPNVYNILLPVIEQCAGIDKSEELCQSSYNLIKCAHRVNPKSLEFLPL  >NribOBP8P  ENNQQNSNDRSATIFQSCISETKLSGDALKGFRSMSIPKTQAEKCMMGCLMRKVNVINKGKFSVEEATKVAQKYYGTNETMMKKAKDLIDVCAKKAQSTTEECALAGIVTTCIVEEGQKAGLTGGPGGRSRRTVSPKFRRNSM  >RpadOBP2P  SDPCNISTCYKSGTTKPPTTVTPTRLPVQSSSTPTSHQQTTYAKDHAHSSIAAKSGANVTATTASGATVNGTERPTVVKSSPGVAGNATTPKPTMTVEHVALKQKLNTIAVKCKDELHAPQEIMALVSNTVVPQNEQQRCYLECVYKNLNLIXNNKFSVDDGKAMARIRFANQPEEHEKAVTIIETCEKEAIIDPKTTEKCAAGRVIRNCFVKNGEKINFFPKA  >RpadOBP5P  DAGHHRRGKELLDTEDSDFFRCKQASRKSCCGPDNAMKRFGDKDKVAADECYAQVAEKFATTKATTPKQDLFSSEAVKVTKKKQFCLHECIGKKNKLLTEDGSLNKTFIADYAMKSIFKEQWQKQIGQKALDKCLEETYIPWPAEETENKCNPVYVQFQHCMWFEYESNCPSNKIKLTKKCEKTRNRYRMQKSTSN  >RpadOBP10P  STRPQPDEMEEIKKTLYNACSAKFPLTDEIRNNAKNSIVADDQNLKCFLRCCFDEMSMIDEDGIIDGESLVSMTSDKLKIVAKKAVDSCLTADKQDGCEAAFKFISCGIKLNPLIGSATL  >AgosOBP2  MKVSAATAVLVALVATVQSSDPCNISTCYKSGTTKPPTTVTPTRLPVQSSSTPTSHQQTTYAKDHVHSSTATKSGVNTTATTTSGASVNGTERTTVVKSSSGVAGNVTTPKPTMTDGHVALKQKLNTIAVKCKDELHAPQEIMALVSNTVVPQNEQQRCYLECVYKNLNLIKNNKFSVDDGKAMAKIRFANQPEEHKKAVTIIETCEKEAIIDPKTTEKCAAGRVIRNCFVKNGEKINFFPKA  >AgosOBP3  MISSTFYTSLMFGIAMLISCSFGRFTTEQIDHYGKACNATEDDLVIVKSYKVPTSDTGKCLMKCMISKLGLLNDDGSYNKTGMEAGLKKYWSEWSTDTIESINNKCYEEALLVSKDIIATCNYAYVVMACLNKQLKLDNST  >AgosOBP4  MRGNYSLVVFLLFGFGLLEIYCQKQELSGKCRAPDKAPLNLEIIINICQEEIKSALLQEALDILNDGTLEQNTPSYSRSKRDADEDLSNEERRVAGCLLQCVYKKVKAVDETGFPVVDGLMKLYNEGVQDRNYYMATLSAVRHCISIAQQLKQQQPSKSFDDGQTCDLAYEMFECVSEKIEENCGVENKLNNLSQRQV  >AgosOBP5  MKMSANGATMKCVAVAVVLFQMSVIFAEAGHQRRGKELLDTEDSDFFRCKQASRKSCCGPENAMKRFGDKDKVAADECYAQVAEKFATVTATTPKQDLFSGEAVKITKKKQFCLHECIGKKNKLLTEDGSLNKTFIADYAMKSVFKEQWQKQIGQKALDKCLEETYIPWPAEETENKCNPVYVQFQHCLWLEYESNCPDNKIKLTKKCEKTRNRYRMQKSPSNQ  >AgosOBP6  MQKVVFLCIFAIICQTVFTVGFERTWILRQKRMTNDNECRALFPSPEKKLPTCCQMPNILPGLDNAWEVCFEKFKQFKDKHATKEYKEMVHENEPPCLFQCVFMQSGLTTSDGKVNEDAVIKKMAEGMDNDEKWKSIWRNTFNKCLNDVKQEDKEQIKVMNTPTGRLMKCFLRDLYMNCPKNVWVENSECSNLKDLVEKCPKLPPPVFQSPPKLI  >AgosOBP7  MNMLPATVLLAVVAATILKDSDAYLSEEAIKKTQKMLKNVCSKKHSVEEEVFTDIKKGIFPENNNNIKCYFACNFKTMQMVNQKGILDKKMFKDKMTMLAPPNVLAILLPPIEQCIGNDKDTEICQSSYNFIKCAHRVDPKSLEFLPL  >AgosOBP8  MFAFKVACLCLSVAVVFGENNQQNSNDRSASIFQSCISETKLSGDALKGFRSMSIPKTQAEKCMMGCLMRKVNVINKGKFSVEEATKVAQKYYGTNESMMKKAKDLIDVCAKKAQSTTEECALAGIVTTCIVEEAQKAGLTGGPGSRSKRTVSPKFRHSIV  >AgosOBP9  MIIKKTLLVSGFVLFGCMFSINKAADDADTADKELMSKLITVAFKCFKDADWGTCGEMITTKYDITQAKYKQCTCHMACAGEDLGLINSNGQPEPAKFLEYVKRINNSVIKSQLQHIYDKCQNVKGTEKCDLAEQFAICAFKESPEMKERVTKLIEMLVKMKPKSK  >AgosOBP10  MEHLRGTNVMFAIVMALLVVQSSTRPQPDEPDDIKKTLYNACSEKFPLTEEIKNNVKNSMVIDDQNFKCFLRCCFDEMSLIDEDGIIDGESLAAMAVDKIKPVAEKIVHDCLPAGKQEKQDGCEAAFKFFSCGMKLNPLTIELLPLQ  >ApisOBP1P  ESDQVPINSSAAVESCLLETNMTRDEFEDMLTSPNARELTILKSHAHKCMFGCVMRKNHIVNDGVVSKEVLSKYVLNFYGRPDYKRRLIIKDVEHIVDVCAKKVADESETDECELAATLVTCIVLEANKAGLVDDPARQI  >ApisOBP2P  SDPCNISTCYKSGTTKPPMAVTPTHLPVQSSSTQTSHPQTTYAKDHVHGSTTTKSGVNATVTTASGASVNGTEPPAVVKSSAGVTGNSTTPKPTMTEGHVALKQKLNTIAVKCKDELHAPQEIMALVSNTVVPQNEQQRCYLECVYKNLNLIKNNKFSVEDGKAMARIRFANQPEEHKKAVTIIETCEKEAVIDPKTTEKCAAGRVIRNCFVKNGEKINFFPKA  >ApisOBP3P  RFTTEQIDYYGKACNASEDDLVVVKSYKVPTTETGKCLMKCMITKLGLLNDDGSYNKTGMEAGLKKYWSEWSTEKIESINNKCYEEALLVSKEVVATCNYSYTVMACLNKQLDLDKST  >ApisOBP4P  QKQETSGKCRAPDKAPLNLEIIINTCQEEIKSALLQEALDILNDGNVEQNTPNYSSRSKREAEEDLTNEERRVAGCLLQCVYKKVKAVDETGFPVVDGLMKLYNEGVQDRNYYIATLSAVRHCISIAQQLKQQQPSKSFDDGQTCDLAYEMFECVSEKIEENCGVENKSNN  >ApisOBP5P  DAGHHRRGKELLDTEDSDFFRCKQASRKSCCGPENAMKRFGDKDKVAADECYAQVAEKFATVTATTPKQDLFSAEAVKITKKKQFCLHECIGKKNNLLTEDGSLNKTFIADYAMKSVFKEQWQKQVGQKALDKCLEETYIPWPAEDKENVCNPVYVQFQHCLWLQYESNCPANKIKITKKCEKTRNRYRMQKSTSN  >ApisOBP6P  PNILPNLDSTWEKCFETFKQFKDKPETKEYKEMAHGKEPPCLFQCIFMQSGLTTSDGKLNEDAITKKMSEGINNDEKWKSIWQNSLNKCFDDVKQEDKKQILIMNTPAGRLMKCFLRDMYMSCPKNVWVESSECLNMKDLVQKCPEMPPPVFKSPPKLI  >ApisOBP7P  YLSEAAIKKTQQMLKTVCSKKHSVEEDVFTNIKKGIFPEDNNNIKCYFACNFKTMQLINQKGVIDKKMFKDKMSMMAPPNVYKILLPVIEQCTGKDKGEELCQSSYNVIKCAHSVDPKSLEFLPL  >ApisOBP8P  ENNQQNGPSDRSATIFQSCIAETKLSGDALKGFRSMSIPKTQAEKCMMGCLMRKVNVINKGKFSVEEATKVAQKYYGTNEAMMKKAKDLIDVCAKKAQSTTEECALAGIVTTCIVEEAQKAGLSGGPGSRSRRTVSPKFRRDAM  >ApisOBP9P  DDADAKDKELMSKLFTVVFKCFKDADWGTCGEMITTKYDITQAKYKQCTCHMACAGEELGMINASGQPEPAKFLEYVNKINNPDIKSQLQLIYDKCQNVKGSEKCDLAEQFAICAFKESPALKERVSTLMEMLVKMKPKSK  >ApisOBP11  MSSSTFYITLLFGIAMLISCGYGIFTTEQIDYYGKACNASEDDLIVLKSYKVPSTETGKCLMKCMITKLGLLNDDGSYNKTGMEAGLKKYWSEWATEKIETINEKCYEEGNTATLLYHVAIYFTCVSGDYSDVQLLIHCDGMFEQEVGSRQVNLKLLIMLKIGLSEPKR  >ApisOBP12P  DDLVVVKSYKVPTTETGKCLMKCMITKLGLLNDDGSYNKTGMEAGLKKYWSEWSTEKIESINNKCYEEGDTSTLLYHVVIYFTCVKGGSSDVQLLVHCDGMFEQAVGSRQVNL  >ApisOBP13P  CTIHCVFNQLEMLNSNSRPDKYSIVNIMTNQIKDVELKEFIQDSIDECFDTLELDSHNNKCEFSKNFAVCMENKAQRNCDDWDENLSANKINSAGLQDGTNQQDKRKGY  >AlucOBP1  MCSKYFVMLIGLTVYTSAEVINEECKDRNQSSTEYETFYNCCDLESSFNETKSKEKEEAREFCENEFEKANNVSEDEAEPSPSSVRQDCYVDCILKKLGAMSEDYKMDKEKVTKWFMEGTHKDFEEVGKQAMEKCYDKTYSKKHCASRVMGLLWCYSEELVMNCPAKYWDQSEKCTAAKAYMKKCSTNPWRSED  >AlucOBP2  MRSTGSECFEEIDAKLGNKTSLESDMDPYNCEKVKRMKKRHYCMHECKAKKLGVATEEGNLEFPKVKELLLSRVNETWQKDILGQAADTCATSKFDQTWKDDTEEYKCNPQALQFKHCVWKQVEMKCPEEHQNTGRHCKKLRSKISSETSKDIAKETSV  >AlucOBP3  MFSSATLVCLFAVALTQGQLDEDPECRPPHPPGKDDKCCTIPELIVGENMQAMMKQCFEESGMERRPPGPPGSGTPPTPEEIEAHRSAHECVDECFFKAAKFMNSDGEFDLEAMKTAAASVFTGDWAPLGSETIDKCFASAKSQVSASAKCTSGAHRAKKCILRNFIINCPPSAWNDSTDCAALKARLTKCSNAMPPFPHHKH  >AlucOBP4  MEVAACLVLLAALAALTAAVEEGRPLCKAPTTAPRKLEKVINQCQEEIKYALLQEAPSVLGETVGLKTALTRNRSKRETFTGEERRIAGCLLQCVYRKMKALDETGFPTATGLVKIYSEGVEDRNYYLATIQGVQRCLSRELQSRNTNPSIVKAEGYSCDVAYDMFNCVSEQIEQLCGTSP  >AlucOBP6  MYDRFKLFALLALVVSCKSAPPEEPAECKLPESDSAELVKCCKLNVVLDEMADSVGECMKLVKGKPEKGPPVPEGFDCMDTCVFSKLGFAANNKLDAEKLTKKFSELFKGDWSALSDSTLKKCLPMAEGAKGSCASGADVFKFCIVRELYMNCPASSWTKSDLCKANVERLEKCPHSMPFLPGTGIKKN  >AlucOBP7  MNPLILILLVVFAAATRGEEQANALVAKAFNKCFGEFPLGDDEMKEVKDKSTVPSSHNAKCLMACMLKEGRILRGGKYELENAILMADVLNKNDHAATDKAKQLIETCAAQVGTDASADECEFAYKMALCASDEAKKLGVRPPDF  >AlucOBP8  MVLKMKQILVVFVALQVLISTTEAVMTQAQMKQAMKTVRNMCIPKSGVDKEALAKMVEGEFDESDQKLKCYLGCVLGMMQAVKNNKINLTMVKNQISKMLAPEQGQRILAAFEGCATVTGDDNCDLAFKFAKCIYDTDKELLFQAFIVP  >AlucOBP9  MKSFVGLIFAVALVEFASAITKEYHDRAVAAKDACLKKHPSIKESDVQEFLKKHKLPETDDGKCMIACYMEEMNLMADGKINVEEAKKTNSDKYDGEPDNKELADKLIDHCSSQVSPDGMSKCEYAYQISKCGLEYGMKNGLTPPKMYEEQRR  >AlucOBP10  MTYHVFFRKFDLPRISRRVRQCYYHSVPRSLSGSSRRMLEETSQHHPKRRSRVSEKHKLPETDDGECMIACYMEEKNLMADGKINVKEANQTNSDKYDGEPDNKQLAEKLIDHCSSQVSPDGMSKCEYAYQFSKCGLEYGMKNGLTPPKMYEEQRR  >AlucOBP11  MGSQYERTLVGVRYLPIMKRVKFILVLSLLSRCSSAPTDDMAACMQITNEDSASMATCCDYVIPFSNKTMTTCDKKETSGEMSKEFECVQDCLFSSDNVLGADKKFDPVAWRKHATNTISGDWKGVIANSGSNCEGFKKVLAQSMEKKCPTSESDVSFNCMTLQWYMNCPKSAWTSSESCEASKKKLMSCFGPIFENTS  >AlucOBP12  MTCSHFIALLSVVALSLSSGEINEECKDIENLKTQLENFYGCCDFESMIERVVRTEEEVETDRFCREERKKINSTDGKVPLASEGHDCFMECVLKRMGAMGQDFKFIREKLDDFFLRGYPEEVKQAGKLAFDKCLSKNFSKKYCASGINGLMMCLPEELVMNCPANIWSSHESCPIAKEAIKKCPSYRVMIEQE  >AlinOBP1  MNSLIPVLLVVCAAATRADEQTNAMVAKAFNKCREEFPISDDEIGGVREKTTIPESHNAKCLMACMLREGKMLRDGKYEKENALIMADVLNKDDPASADKAKQLVETCAGKVGTDAGGDECEFAYKMAVCAAEEAKKLGVRPPDF  >AlinOBP2  MSLKIQFFVFAAICAACVCAYQEQLKQTIRDCQDGKEVTDDELEEFTKPLIPRNREEKCIMACVMRTYNIISNGHYDPKIAFGILKGILKDHPEKLNKIKEVMDHCGEDVPSHMDDECDLAGEIMQCEVKYQKAMGMA  >AlinOBP3  MDIRFGFIIACLAILSVANAISKEYSARMIAAKEKCQKEFNVTDSVVEDFMKRNIKPESKSGKCMVHCIMEEMGMIDDHKINTEQVKLGNKEKWDDPALVELANQVADTCDQEVFTEGRCKCLVAVEYMMCLATHGDEVGLPHVDFEDSQDS  >AlinOBP4  MRIFVIFTAALTCVMAGELPEEMKEMAQGLHDSCVEETGVDNGLIAPCAKGNFADDAKLRCYFKCVFGNLGVISDEGELDAEAFGSILPDSMQELLPTIKSCGGTTGSDPCDLAMNFNKCLQKADPVNFLVI  >AlinOBP5  MVLKMNLLLVVLVMSQVFFSVTEAAMSQAQMKQAMKTVRNMCIPKSGVDKEALAKMVNGEFDESDQKLKCYLGCVLGMMQAVKNNKINLTMVRNQITKMLAPERGQRILAAFESCATVTGDDNCGLAFRFAKCIYDTDKEAFIVP  >AlinOBP6  MGFKFVKYRSYFFVLVIRIILCIQIKAKELTDEQKEQIFAEIKNCMESTKLTDEEFESIMAKKELPTSIEGKCFTKCLMEKMEYLEEGGKINVIAVQAGMEENMEKESEITKAKEVIQQCADSVPPEDSCEYAYGISQCMYNKMKEAGISGS  >AlinOBP7  MNRPLLLLTAVLTVGSGQQEDCKTAPAGWPRRPPQCCDLPFPLEGMKKEFGSCIRQIGNRQSSAVPTAQAVRDARLCIEECVYKGLGFMDEHKLNKDQLLEQLKKGIADKKDWTKPMEGAVKRCHETITKRETPQEAACQDSAHEFTHCAMRELFLNCPASEWNNNDECNLVKSRMQACPNIPPPPPPPPQGFRGQGPPPQ  >AlinOBP8  MDTHFGLLIASLAILHTANAVINKDYLEKVVTAKDKCLKEFNVDDSVVEDFIVKYNKPQSESGKCMVACFMEERGMMKDGKTITEQVMLDNQEKWIAATHVNMGKEVIDTCDKEVPNEENDKCDLAVDYMMCLVKRGDEAGLPKMDVAQLKH  >AlinOBP9  MMELWKWRLALIIFGLVSCIQQTEGSQRTKQQPKSKTKESVVGATRPRDAKATECVNKVNANEEESASFFRKEIPETEAGKCLLACYLEGKGLIVGGKISSSGAARVAARAYPNNRVKTGNVKHILSHCGTIAGRESNNCEMAYKLADCTTTLSDKFRL  >AlinOBP10  MFFNSVFLLVVCVSSYVTKGQELPPPGDVKNKTVVFKNSFLRSAKYCSSIYETSTLAIMALLMSEKSDDQNGKCFLNCMLQRYRLMSQDGSYNKDKFKPFLEYIPDSKFLQSIRGNLKNCISEKDPDPCEKASKFIKCFYTRARNKGEIGASKEVIPADGF  >AlinOBP11  MKTFVGLIFAVALVEFASAISKEYHDKAIEAKNTCAKLHNVDDETIMTYWKNHQLPEKEPETCIVICYLKEMKLVVDGKVDADAWKASNKEKWDDEKHVAAADEIVDKCSAEVPPTENECEWGLALTKCALKHGKEAGIPPPDMEHPKRR  >AlinOBP12  MTTKLRSIGLVFIVSISYAFAYQELLKETIKKCQNGRDVTDDEVEEFTKPLVPKNEEERCLVACVFKEYKVIIDGHFDPVNALNVAKVVYKDYPDKVERIKDVLDHCGEDIPTHNDNECDLAGDIMKCEVKYLNSVPKMTSLEFLAGSMAATAEP  >AlinOBP13  MNISTRMISLTMAYLAAALVSGHRALDGILPQANQDECREESNFRGELNDDVGRNVTQELKCFAACSLMKLGIMNEKDGTVNMTRLDELIASHTPGKDAADVFKTTVVEPCMKEVKKSTDYCEYSYQLIACGMSKVP  >SfurOBP1  MLLEVCRFSVFLLAFSATVYGRFSEEEKQLMNQVHTQCVTETGTSEDLVNKATNGDFAEDENLKCYVKCIWSTLTVMDDDGNFDVGVLEVMLPADMKDIVMKAMSACIGAGGGSPCEKAFAVTKCLYKEAPADFFLP  >SfurOBP2  MSTLLNFVFVFLVCLCSYSEASPALTEAQIEQVGKAMANMCISSSGVQRSLITKAMTGEIEDDRKLKCFFGCIMEAVQVTKNGKMQPEVLKRRANAMLPKTMREMILPTIDSCSHIENEDKCELAYSIVKCHFSVNGKNPFFFNF  >SfurOBP3  MPPVFRTAHDKFESCLEELSSIFPPPPPPNGHHGPPPPGGPGGHGPPPPPPPGGRRGPPPGFGGPPGHEPPIFACAHECLFNKTGLLENGKLNVEALKKKLEGELGDDEVWKNLLQSIVDKCMESKDPPSNDMCTSGSHELARCVLRDMFMNCPQEKWKESDDCSNMKMKLEKCPELVPPMAMRLSQPPMP  >SfurOBP4  MCCDLPLVYRGTPELFKACREELGFPDHKPPPPPPSSDGHGPHGHPQRGMCVAECLFNRTGLLENGKINKEALKKALDEYLKTDGAWKDVATTTLEICYDAQTRGDFKPDNEKFTSGSSEFLKCFTRNLFMDCIPEKWTDSEECKKMKEKIDKCPKMLPPALFNKRPH  >SfurOBP5  MLYVSYFVIVTAASSAVITQIMAADSNNPDMQTVFNNCREEASATEDDIKTFRAQQIPSTTTGKCMLACMFNHSGLMKEGKYDSEGALKLVGQVFAADPVKLGKAKTLINTCSDEVKNENDKCEIASKIADCTVKMTSQVGLS  >SfurOBP6  MKCQVFLASFVLVAVFELGYAGLTPEKLKEIKPLIDTCIKESKVEEETLGKLHNGHEIPSSQSGKCFIACMAEHMKLMKDGKFEPAMTMEFIDKMVQDKVKADEIKKAVDDCFKSVPDGDKCEMAASLATCMKEHHAELAGMN  >SfurOBP7  MEEEMLSIGRVQRDADQTQEVADEYFKCKHRNLKTCCGKINLMKNYGDKGKIYGKQCYEEVVSAFKTNSSSTADDDDSMMDMFSCEKVKMIKLKHICVHECIGKKTKILKEDGSLNAEEIKQYAREYMFNEEWSKELGERALDKCLTQSYNSVTKMLDEYEIKCNPTSVQFHHCLWKEIEMTCPESKVDLKAKCVRLRERLRKQQAAGM  >SfurOBP8  MERTHVLIIAFAFIPFLSSAMQADFAMMQFPMQGTGTPMIQSIAGELKYCMDVNAEQNSDGLEDYLPLLFNEELPTSLGQKCFLTCLFNRFGLLKDGFLDAQTAKTLVETFYKDKHDEKTMANIAINVCRVSAVPDILNPCEIGFSLKSCFVDSNKKGKELRGKN  >SfurOBP9  MNTFQKFILSGMVVLAGAMLITAEDTTIKIKNQQSPHKQQQVYCQAPPTAPERLERIIEQCQDDIKTALLQEALNVLTDTSPRDLVKKTRSKREVFSGEEKRIAGCLLQCVYRKVKAVNDQGMPTVPGLVRLYSEGVQDRNYYVATVQAVQQCVSASQHFRYYNPQVLKEDGYTCDLAYDMFNCVSDKIEAFCGRTP  >SfurOBP11  MLLEVCRFSVFLLALSATVYGRFSEEEKQLMNQVHTQCVTETGTSEDLVNKATNGDFAEDENLKCYVKCIWSTLTVMDDDGNFDVGVLEVMLPADMKDIVMKAMSACIGAGGGSPCEKAFAVTKCLYKEAPADFFLP  >NlugOBP1  MKSFIVCIAVSYLLVANIKADEATSSSDAESLITSTTLSPASNESDAARSAIKEQLAKLTESCKTSSQANSDDAKIIGTESVPKTEGEKCFLQCVYTGFGIVKNDQFSVEGARLLAQKRFGAFPEELEKANQLIETCSKEAVKKDSKDKCPMGFLIRQCFVKNGQKINFFPKA  >NlugOBP2  MKCQIVLAALALATICEVSYAGLTPDKLKELKPLIDTCIKQSKVEEDTLGKLHNGHEIPSSQSGKCFIACMAEHMKLMKDGKFEPEMTMEFIDKMVQDKDKAAEIKKSLGECIKSVPEGDKCEMAAGLATCMKDHHAELAGMN  >NlugOBP3  MKASAAITLVFLSLAVFHCSEAKLDKAKKEAAIKKCQAETQATDEDVMKVRKEHIVPDSEEGKCFIACGFNSYDMLKDNRINLEGVNAFFEKLYDEQDKRDIAIKAAASCAATETVSGLNECHYAAKYFACMQRHPDFAKMKDDFDI  >NlugOBP4  MERTSVLIVFTFIPFLSSVLGANFLMMQQSMQGTQMPMIQSIASELKFCMDVNAEQNSDGLNDYLPLLFNEELPSTLGQKCFLTCLFNRFGLLKDGFLDTKTAKNLVETFYADKHDEKTMANIAINVCHVAAVPDALNPCEIGFSLKSCFVDSNKKGKELRGKN  >NlugOBP5  MGIYTTNLIFTLLGSAVVSGVFIDGRNEYRLTRQAPPDDECRPPRPGPNEDGVCCDMPPVFRTAHDKFESCLEELSSIFPPPPPPPHGHHGPPPPPPGARGPPPPPPPGGRRGPPPGFGGPPGHEPPIFACAHECLFNKTGMLENGKLNVEALKKKLEDELGENEVWKNLVQSIVDKCMESKDAPSNEMCTSGSHELARCVLRDMFMNCPQEKWKESDDCSNMKMKLEKCPELVPPMAMRLPHPPMP  >NlugOBP6  MSTFHKFVISGMVVLAGALFVTAEDTTIKIKTPSPHKHQQVYCQAPPTAPERLERIIEQCQDDIKTALQEALNVLTDTSPRDLVKKTRSKREVFSGEEKRIAGCLLQCVYRKVKAVDDQGMPTVPGLVRLYSEGVQDRNYYVATVQAVQQCVSASQHFRYYNPQVLKEDGYTCDLAYDMFNCVSDKIEAFCGRTP  >NlugOBP7  MLLEVCRFSVFLIALFATVNGRFTEEEKQLMNQVHSQCISETGTSEDLVTKATTGDFADDDNLKCYVKCIWSTLTVMDDEGNFDVGVLEVMLPADMKDTVMKAMNACTGVGGATPCEKAFAMTKCLYKEAPSDFFLP  >NlugOBP8  MVTSALMQTATAACLLLVTAYAYDFSDPYFNEHLQSAMEEIMEEEMLSIGRVQRDADQGQEVADEYFKCKHRNLKTCCGKINLMKNYGDKGKIYGKQCYEEVVSAFKTNSSSTADDDDSMMDMFSCEKVKMIKLKHICVHECIGKKTKILKEDGTLNPEEIKQYAREYMFNEEWSKELGEKALDKCLSQTYNSVTKMLDEYEIKCNPSSVQFHHCLWKEIELTCPESKVDLKAKCVRLRERLRKQQAAGM  >NlugOBP9  MPPVFRTAHDKFESCLEELSSIFPPPPPPPHGHHGPPPPPPGARGPPPPPPPGGRRGPPPGFGGPPGHEPPIFACAHECLFNKTGMLENGKLNVEALKKKLEDELGENEVWKNLVQSIVDKCMESKDAPSNEMCTSGSHELARCVLRDMFMNCPQEKWKESDDCSNMKMKLEKCPELVPPMAMRLPHPPMP |
| **CSPs** |
| >AspiCSP2  MLKLLAVVCVLSCAVYVHSAPRAEEKYTDKYDNINVDDILGSKRLLKSYLTCLLDKGACTPEGGELKRLLPDALKTACAKCTEKQKEGAARIVERVTSEYPGEWKELSAKWDPTGEYWAKYKPLIDDYLKATA  >AspiCSP3  MNQIILALFALCALIGFSSAAAPSPRYTDKYDGIDYKNIIKNPRLLDAYFKCLMDQGNCTPDALELKKNLADALTNKCSKCSDKQKTASEEIIRFLIKEKPEMWEKLEGKYDPKGIYRATYKDELVMLTKKE  >AspiCSP4  MKCTYGLIVLSVWCVSSTFGQSRIGESDVSGQLSDRNYVNKQINCVLDKGSCDNVGTQLKKAIPEVLGRQCRSCTPKQSENARKVIGFIRSNYPAAWSQIEAKYGRAPF  >AspiCSP5  MKSAVFVFILFIGVCYAAPLNDDLLKKYDNFDLDKVLNNERILTNYVKCFMDEGPCTNEARDFKQNIPDALAGGCDNCNEKQKNMTEKVIRHLVTKRPKDWERLSKKYDPQGLYRHKYDYLLEKVKKGESIELKDLKSNPKEQTPVKPKETPKKDAKIETKKEVKEPAKATKT  >AspiCSP7  MGRVVFLCALAAVFVVFVSGLDKSDLEKFESMDISSILSNNRLRSSYVNCMIDKGPCTADGAEFKKIIPSLMESNCAECSPKFKEMIKKSIGTFKKDFPEDYKMLMDHYDPEGKRTAAMEEFMSS  >AspiCSP8  MGTLKVILPLVIGICFFYTHYATPIPGEEKYSTKYDNIDYKSILNSKRVLNSYVKCLLDEGPCSPEGKILRDELPTIIKTQCKKCTDKQKRTSLDVLEIIQKDHPDEWAVLHKRWDPEDKLAKPLIESLRKDIGQA  >AspiCSP9  MNKYVIALFCVVATLVAVHAADDMYTDKYDNIDLDSILASKRLIRNYMNCFQGKGACTPEGTYLNQVLPDALKNECAKCSEKQKQGAVKAIKKLSGDYPEEWKEITDKLDPTGELYQKFKAKYP  >AspiCSP10  MVLSMRALRLLLPIVCIGIYALQRVQTSPVPEEGYPTKYDGIDYKSILKSKRLLTNYTKCLTSEGPCTAEGKMLLELLPDAIATKCRKCNQNQKSGTLDVIEILQKEHPDSWKILQGKWDPDNNLTEPLLEQIRKELGRS  >AspiCSP12  MNYKFGFLTLLVNFNSIVSIDEEVKLEDLCSSPVFDRFDINVFLRNDRLVTNYFGCFMDEGTCTSEGRAIKRLIPEIVRTQCKDCKPPLQRIVRTVMKFMFEKRPEDVERFFEKYDPQKTYRVDLVEFMDQE  >AspiCSP14  MIKYYSFSAVLACVFVAAVARAQDNQKQVQVPVNEMLNNTKMREAYFKCMSDKGPCTPDAAELKKVLPEAMTKKCAACTDTQKKILSRILDYMMEKDKATYKDIQEKYDPKNEYSKMREEELKKEKEEAKKKPAEKDAAKKS  >AspiCSP15  NQILTLLCVTVLLAQGMPAPQTTRSTISDEALESALNDKRYLMRQLKCALGEGVCDPVGRRLKTFAPLVLRGACPQCTPTETRQIQKVLSHIQRHHPKEWSKIVKQFTS  >AspiCSP16  MLKLLVFSSLVLLAACLPAQPQPNPKKSVKEAIGDKPEELPKTMKEALKRMEAVDVEKILSNDRILTNYLKCFLNKGPCTSEAKSVKTFIKMLVDTRCAECEPRQRKIIKKAMLAVKNKKPKEYQELVKLYDPKGTQAAELEKFILASR  >BtabBCSP1  MHLFSVVVLVCCLLVAVLSAPAEFYTSQFDNIDIESILKNEKLLDNYFNCLMDEGPCTLEGRTLKSLLPDALNTSCAKCTEKQKKIARRVMTFYLDKYPANSARIIKKYDPENKFKDGIEKALLGSR  >BtabBCSP2  MFKVLVVLCVLGAAFVYAAPAEDKYTDKYDNINVDDILGSKRLLKSYLTCLLDKSPCTPEGSELKRLLPDALKTACSKCTEKQKEGAARIVERVTAEYPTEWKELSAKWDPTGEYWAKYKPLVQEYLKASA  >BtabBCSP3  MQVLTLVVLVGCAATAVLSADTYTTQFDNIDLEAILKNEKLVDNYTKCLMDEGPCTNEGRTLKKLLPDALKTACAKCTEKQKTGARKVIKFYQTQHPEDFKKLQQKYDPEGKFKAEFEKALFGQTL  >BtabBCSP4  MRCACVLLVLVICVWGTSGQRVGEGDVSRLLTNRDYVNRQINCVLDKGSCDNIGRQLKQAIPEVLGRQCKSCSARQLDNARKVVNYIRSNYPGPWSQIEAKYGRAAF  >BtabBCSP5  MKSVCVFAALVVACYAAPPAGVDEKLLSKYDNFDVDRVLNNDRVLANYIKCLMDEGSCTNEGRDLKKSIPDVLAGGCDKCTEKQKMVTEKVIKHLINKRPKDWDRLSKKYDPQGQYKNKYADLYEKVQKEAAKESKEPSKPTKDTTKVTKDTKESAKAPKA  >BtabBCSP6  MNKIVLALFALCALFGFSSAAAATKESTKESTYTNKYDNIDLGKILTNDRLFLNYFKCLMDEHTCSPDGAELKKVLPDALSNKCAKCTERQRSGSEKVIRHLIDNKPEMWAKLEAKYDPKGTYRKTYKNEAEKLGIKV  >BtabBCSP7  MIRVTLLLVAVALVGFVAGAPAPLEQSDLEKFENMDLSSILSNKRLRTAYVNCMVDKGPCTADAAEFKKILPDLTETQCADCSAKFKELIKKSVSTFQKDYPEDWKTLMAHFDPDNKRAADLEKFMSS  >BtabBCSP8  MKCVVLLAIFSVLAVYFVESAHLGPNFGAGDISGHLKNKNYILKQLNCVLGKGACDNVGKQLKVAIPEVLNKNCKGCTSQQAANAKRLITFMKSNYPAEWSKIAAKYKK  >BtabBCSP9  MSKYVFVLCVVAALAAVVSAADDFYSDKYDNIDLDSILASKRLIRNYMNCFQGKSPCTPEGTYLNQVLPEALKTECAKCTEKQREGAVKAIKKLSAEYPEEWKEITDKLDPTGEQYAKFKARFP  >BtabBCSP10  MGTFRALLLVVVSVCVFNVLRATPVPDEEKYSDKYDDVDYKSILNSKRLLNNYVKCLLDEGPCTAEGKALRDQLPDILATECKKCTDKQKKGSLDILEILQTEHQDAWTVLAKRWDPEDKLTKPLMEKLKKETGQA  >BtabBCSP11  MVCSESIVRCLTYFQVISLVYRLASAQSNNRATYTTKYDYINVDAVMKNERILKMLVECMLERGRCTREGLELKAAVPDALATDCAKCSQMQRKHASRVIAYLITYKKEYWNALATKYDPDGSYRRKYGIPPQPQELSAGAAIQPSPNLIKPTKTVKKTVTTVNNTNNLFKAKKRMTREEKKEMVRRRFPQLHFMPNMWASNIQKIEKKSERAPSIARRNVRRKAHTPTRRHSTQRTAPKRGQETG  >BtabBCSP12  MNPTVFLVILGQLSFVFSAISDDEYRLETLCSSPALEHFDITPILKNDRLVSSYFKCFMDEGPCTNEGKMVKRIIPEIMRTQCRNCNPTMRRIVRTVMKHMFQTRPRDVDDFFLKYDPHEMYYDDLIEFMDEDNDY  >BtabBCSP13  MNSLVLCAFVGSFIVGTLAAPAETYTTEFDGIDIDSVLKNEKLLDAYAKCLLDEGPCTREGRTLKTLLPDALETTCAKCSPTQKEKAKKVITFYMEKYPENAQKIMKKYDPTGKYRKALEEAFLGSL  >BtabBCSP14  MMKYCALSAVLACVFVVAVGRAQENQKQVQVPVNEMLNNTRMREAYFKCMSDKGPCTPDAAELKKVLPEAMTKKCAACTDTQKKILSKILDYMMEKDKATYKEIQEKYDPKNEYTKMREEEIKKEKAEEKKKPAEKDAPKKS  >BtabBCSP15  MIYVQILSFLCLSVLLAEAMPAPQTTRATISDEALESALNDKRYLMRQLKCALGEGVCDPVGRRLKTFAPLVLRGACPQCSPTETRQIQKVLSHIQRHHPKEWSKIVKQFTS  >BtabBCSP16  MFRLLLVTSLVLLVTGLPQKGPASTPRKQSVEEALGKKPEELPKTMKEALKRMEAVDVEKVLNNDRILTNYLKCFLNKGPCTSEAKNVKKFIALLVESRCVECDPKQRKIIKKSMQVVKTKKPREYQELIKLYDPKGTQIAELEKFFASSK  >BtabBCSP17  MNFLSVVVLVCCLFAAVLSAPAEFYTSQFDNIDIESILNNEKLLDNYFKCLMDEGPCTLEGRTLKSLVPDALNTSCAKCTDKQKQIARRVITFYLDKYPANSARIIKKYDPENKFKDGIEKALLGSR  >BtabBCSP18  MNLLSVVVLVCCLFAAVLSAPAEFYTSQFDNIDIESILNNEKLLDNYFKCLMDEGPCTLEGRTLKSLVPDALNTSCAKCTDKQKQIARRVITFYLDKYPANSARIIKKYDPENKFKDGIEKALLGSR  >BtabBCSP19  MHLLSVVVMVCCLVAAVLAAPAEFYTSQFDNIDIESILRNEKLLDNYFKCLMDEGPCTSEGRFLKNLLPDALNTKCAKCTDKQKKIARRVMTFYFDKYPANAARAIKKYDPENKLKDGLEKALLGAR  >MperCSP1  MNTLLLAVALCIAITMTVVQTAPAKYTTKYDNVNIDDILNNDRLVASYFKCLMETGKCTPEGEEIKRWLPEAIENKCENCSEKQKIGSEKIIKFLIEKKNDMWKQLEQKYDPQGLYKQRYSEEAKKLNLDV  >MperCSP4  MTNNNMNSPRCRPEIFSLLAVAAIATVLVHQPSTVHCADAGVYPPQQQQQEATMFTAPSGYYVSTYDHMDVGRLLRNNKVVAGFVKCFTNEGPCTPEGRLAKAYLLPEIIRTVCGKCTPRQKDMARLVIRHIYTYRRGDFDKIMQIYDTDGKKNEIIDFMNQK  >MperCSP5  MNCKVLIALCCVAVYAAHASPAGAATAAAASADEEIKDFPAYMKRFDKLNVEQVLNNDRVLASHLKCFLNEGPCVQQSRDLKRVIPVIANNGCNGCTERQMTTIKKSLNFLRTKKPVEWARLVKIYDPSGTKLNKFLDA  >AgosCSP1  MNILTIFCYVTVMCDTQVKPAVSAQRLQSVNQNVTPTNDGRKTIRETSSYPTRYDYIDIEAVMNNERIIKILFNCVMSRGPCTREGLELKRIVPDAIQTECAKCNERQRKQAGKVLAHLLQYKPEYWKMLVQKFDPNNVYLRKYMADNDDDEKLSLQKLSNDTTKKKRNI  >AgosCSP2  MAHLNLFVVLIASLIYFTSAAEEKYTTKFDNFDVDKVLNNNRILTSYIKCLLDEGNCTNEGRELKRVLPDALKTDCSKCTDVQKDRSEKVIKFLIKNRSTDFDRLTAKYDPTGEYKKNLEKFEKERASAKPLKA  >AgosCSP4  MDSRIAVVCVVLAAFAVDQTVGAPQKDAVAASGPAYTTKYDHIDVDQVLASKRLVNSYVQCLLDKKPCTPEGAELRKILPDALKTQCAKCNATQKNAALKVVDRLQKDYDAEWKQLLDKWDPKREHFQKFQQFLAEEKKKGFTKF  >AgosCSP5  MHCKVLIALCCVAVYAVQASPAGTATAAAVSADDEIKDFPAYMKRFDKLNVEQVLNNDRVLASHLKCFLNEGPCVQQSRDLKRVIPVIANNGCNGCTERQMTTIKKSLNFLRTKKPTEWARLVKIYDPSGTKLNKFLDA  >AgosCSP6  MIKLILAIAFCVSITMTVVQTAPAKYTTKYDNVNIDEILNNDRLVASYFKCLMETGKCTPEGEEIKRWLPEAIENKCEDCSEKQKLGSEKIIKFLFEKKNDMWKQLEAKYDPQGTYRQRYAEEAKKLNINV  >AgosCSP7  MSRSSSSVTMKVFVIAICVCAALARPEDVKVENKPAVIKSETLAAPLPTNIVKRATDTIQLDSSLPNVSEDVLDKALSDRRFVQRQLKCATGEGPCDPIGRKIKAHAPLVLRGMCVKCSQSEIKQIQRVMSHIQKNYPKEYTKMLKQYQSGF  >AgosCSP8  MNNIIMNNSRGRYGIFSLLAVTIAAIMLVHQPATVRCADGGIITPQQQQQQTMMFTAPTGYYVSTYDHIDVGRLLRNNKVVSGYVKCFVNEGPCTPDGKLVKAYLLPEIIRTVCGKCTPRQKDMARMVLKHIYTYRQADFEKIMQIYDTDGKRNEILAFMNH  >AgosCSP9  MSAFCLNSFILMTMITVIVTHATFTRSTKFDDRTGIDIHLVKRDTDDVNDDENSVESDEGFFYRFTHFFQDSSDKEDDDDDEKKPDFITTFDIFKLLDEEYAMQQFYCVINEDPCDEVGMRLKATIPEEINRNCERCTSTERNNIRRILNYVKKHYPQFWKRVEPIYKKKI  >AgosCSP10  MINTRPRKLVRCIRGVSISVAKGDDAVNAENKDDDSHLVNREEIQRYMSMMEKINIDQMLNNTRLMSNNVKCFLNEGPCTAHLREMKKMVPMLVKDSCSSCTKEQKIMMKKAMDAVKARRPNDYEKLSKFFDPEGKYEKKFLENLNESK  >AlucCSP1  MLKVLVLLAAVVCCVSAAATYTSKYDNIDLDEILSNTRLYKKYFDCLANKGKCTPDGKELKESLPDALKTNCAKCTKKQQEGTDKVLRHVLKNKPNDYKVLESIYDPTGIYRKKYEIEAEKRGIKLPGSH  >AlucCSP2  MASKLSVVLLIGAVGMVLAADKYTDKYDNIDLDEILGNQRLYQKYFDCIQGKGKCTPDGAELKETIPEALKTECAKCSDKQKAGVEKVLRHLIREKPDDYKVLEDQFDPEGVYRKKYEDLKKKVEEGKPIEY  >AlucCSP3  MKVAVLVLLCVGAALSAEVYTSKYDNIDVDKILSNDRILTQYIKCLMEEGNCTNEGKELKKTLPDALASGCTKCSEKQKAQTEKVLRHLSKNRPRDWNRLKNKYDPKGEYSKKYEKEAKAISA  >AlucCSP4  MKFVAALFVASVAVLAVEAADQYTTKYDNIDLDDILKNQRLYKKYFECLTNKGKCTPDGKELKEHLPDALKTGCSKCSEKQRAGSEKVIKHLLKNKPSDYAILEKIYDPQGSYKKKYEAEAKKLGINV  >AlucCSP5  MVGKLSVVLLIGAVGMVLAAELYTDKYDNIDVDEILGNQRLYQKYFDCIQGKGKCTPDGAELKKNIPEALQTDCAKCSEKQKAGVEKVLRHLINEKPEDYKVLEEQFDPEGVYRKKYEHLKKKVEEGKPV  >AlucCSP6  MVSKLSIVLLIGALADVWASELYTDKYDNIDVDEILGNQRLYQKYFDCIQGKGKCTPDGAELKKNIPEALQTDCAKCSEKQKAGVEKVLRHLINEKPEDYKVLEEQFDPEGVYRKKYEHLKKKVEEGKPIEY  >AlucCSP7  MVSKLSIVLLLGALADVWAAELYTDKYDNIDIDEILNNDRMYKNYFNCVMGNGKCTPDGLELKAKIPEALQTECAKCSDKQKKGAEKVLRFIINQKKDDYKLLEEKFDPEGVYRKKYEAQKKLAEEGKPIEY  >AlucCSP8  MLKVLVLLANAASTYTTKYDNIDLDEILSNQRLYKKYYDCLANKGKCTPDGKELKEALPDALKTNCSKCSKKQQEGTDKVLRYVLKNKPNDYKVLENIYDPSGNYRKRYEDEASKRGIKLPGSH  >AlinCSP1  MLKVLVLLAAVVCCVSAAATYTSKYDNIDLDEILSNTRLYKKYFDCLANKGKCTPDGKELKESLPDALKTNCAKCTKKQQEGTDKVFRHVLKNKPNDYKVLESIYDPPGIYRKKYEAEAEKRGIKLPGSH  >AlinCSP3  MISKLSMVLLIGAFADVWAAEQYTDKYDNIDIDEILNNDRMYKNYFHCVMGNGKCTPDGLELKAKIPEALQTECAKCTDKQKKEVEKVLRFIINQKKDDYKLLEEKFDPEGVYRKKYEAQKKLVEEGKPIEY  >AlinCSP2  MKVAVLVLLCVGAALSAEVYTSKYDNIDVDKILSNDRILTRYIKCLMEEGNCTNEGKELKKTLPDALASGCTKCSEKQKAQTEKVLRHLSKNRPRDWALLKTKYDPKGEYSKKYEKEAKALTA  >AlinCSP4  MRIILSAFLVAMACSLATCEMTEEEFYTKVFEEVDPDFILDNERILTSYLKCFYNEIECNAHAEVVKKSIPDVLATVCGRCSDKQKSIFKYSLNKFIPAHPKDWEKILSIYDPSGEAWPKVKAFIES  >AlinCSP5  MGHLTIVLLAAAFEVLTGSRAYTTHYDYIDVDQVLNNTRLYTKYVECLLGQGKCTPEARELRDKLPEALQTNCARCSERQASESHRVIRFLIQNRQEDFKLLEAKYDPSGLYFKRFEEETKRNVSLS  >AlinCSP6  MFYKLSVVVLMGILAGVWAADKYTDKYDNIDIDEILTNERLYKKYFDCIQGTGKCTPDGIELKEKIPEALKTECAKCNEKQKAGVEKVMRYLITKKPEDFKILEDKFDPEGVYRKKYEAQRKLVEEGKPVEY  >AlinCSP7  MNYKLSVILLIGVLASVWAASTYTDKYDNIDLDEILTNERLYKKYFDCIQGKGKCTPDGTELKEAIPDALKTECAKCNAKQKAGVEKVLRHLLTKKAEDYKILEDKFDPEGVYRKKYEAQKKLADEGKPIVL  >AlinCSP8  MDYKLSVMLLMGVLACAWAADKYTDKYDNIDIDEILNNERLYKKYFDCILGNGKCTPDGTELKETIPDALKTACAKCNDKQKAGVEKVLRHLLTKKAEDYKILEAKFDPEGVYRKKYEAQKKLAEEGKPIAL  >SfurCSP1  MFNLLTLVVCLSTIAVQIQAAPEEAQYTTKYDKINLDEILNNDRLFKSYFGCLMGGKCTPDGQTLRDILPDALETACSKCSDTQKAGTEKVFKFMIEKKPSEFADLEKKYDPNGKYRARYEADAEKFGIKV  >SfurCSP2  MVLADTPTTSPKVETKAVESGKSSSKDEIPDQTFDRYINNERYMLMQYECLMGNKPCDHVGRKLKAAVPLVVRGLGCPKCSQREEDQMKRIVSHVQRSYPDKWQKLIKKYGN  >SfurCSP3  MQLLYALVFGCTLVMVSSDMPQSTYPTKYDDYNPDDILKNDRLFNQYFICLTKKKGCTTAGELLSAIIPDALATSCAKCSAKQKAIGEKVIRFLYFNKPDEFAEMSKIYDPEGKYLEMYIASGGLI  >SfurCSP4  MKCPLLSVSCLWISLLALSSSASAATKEKDPERKALYRLEYIDIEKVLDNNRMLTNFIRCFLRKGPCSPEARDFRKLLPKLAKTMCSDCSPRQRFIIKKVFKHLMEERPKEWELLMDRFDPQRKYAERLDTFMVDMTTPSTTTTTTSTTPSTPMSSTTQRIIEILRTSTEMSNESSP  >SfurCSP5  MSEILVTSLIFMLLAASELGLGQQQQTQKPQQQNVDNIEMSIYDKMFENMDVNSLLKNHRLVDSYLKCFLNEGSCTHIGHEVKMMIPEVIRSRCATCGENQMRALKAGLRLFIVRRPDDWKRFLDVYDPDRTEWPHIKAFMESDD  >SfurCSP6  MKLALFCCLLGLVAAVSAQSEKSEKPEKYTTKYDYINVDEILSNDRLFNSYYKCLMGGKCTPGGPELRTHLPDALQTNCSKCSEKQKEFSDKVIKHLMDNKPEEFSALVKKYDPEGIYKDAFKPKHNQ  >SfurCSP7  MRASKASSLVAVLLIAVWGFTGVQAQQKSKDTRYTTRFDSIDVEVILKNERIFKRYMDCLLDKGRCTPEARELKRLLPEALKTECLKCSEVQRRQGAKVMGFIIKNKRPYWDLLLAKYDPQGIFRAKYNYNENNIEGVLKQLEREQQGLYGTYSNTTNTTNTVNSTSTRK  >SfurCSP8  MLKFTLTLLVLAVVSVNCGKLYKDRYTTKFDKIDLDEALNNQRLFESYLKCLMGDKCSPDGYELREALPDALATACAKCSDAQKAGTEKVIRFLIEKRPKEYALLEKEYDPEGIYRDKYKPIAQEKGIKI  >SfurCSP9  MRCLLLVAVVFAAFIAAARADEANKYTSKYDNIDIDKILKNDRVLSQYIKCLMGEGSCTQEGRELKRLLPDAIQSNCSKCSEKQRQASVKVMRHLRQSKERDWNRLLDKYDPQGDKRKNLKLD  >NlugCSP1  MFKNVLLVCLLVAVVSAKPKPAEKKQYTTKYDNIDLDEILNNQRLFDNYYKCLLGGKCTPDGQELREALPDALATACSKCTEKQRVGTEKVIKYLIEKKPTEYSELEKKYDPQGNYKRKYQAEAAKRGIKV  >NlugCSP2  MSKLPVTLVLMLAVFSVDCGKLYKDRYTTKFDKIDLDEALNNQRLFESYLKCLMGDKCSPDGYELREALPDALATACAKCSEAQKAGTEKVIRFLIEKRPKEYALLEKKYDPEGIYRDKYKPIAEMKGIKLD  >NlugCSP3  MKFLCVTIFECALIVVAFGMPQDTTYPTTYDDVNVDDILHNDRLFNRYFTCLTKKEGCTPEGKLLAATIPDALATTCAKCSAKQKTAAEKVIKYLYFNKRDKFDELAKIYDPESNYLNKYLVDGFPAKV  >NlugCSP4  MFLIAVWALSPRRLPWGLPWGGLAGVAAQQQAKNTRYTTRFDSIDVEVILKNERIFRRYMDCLLDKGRCTPEARELKRLLPEALKTECLKCSEVQRRQGAKVMAFIIKNKRPSWELLLAKYDPQGIFRAKYMYNENNIEAVLKQLEREQQGIYGTYSSTNSTTSSNSTSIR  >NlugCSP5  MRCLLLVAVVCAALVAVCHAQDSKYTSKYDNIDIDKILKNDRVLSQYIKCLMGEGSCTQEGRELKRLLPDAIQSNCSKCSEKQRSASVKVMRHLRQSRERDWNRLLDKYDPQGDKRKNLKLD  >NlugCSP6  MLWAARFIVLPLLFCVLQVWSAPADEKYTDIDFDSILANRRVLSSYVKCLTDKGPCTPQGKELKKIVPEVIQTSCTKCSPQQKKVVRNVITTMQSKYKDQWDLVVNKYDPKKQRSGELKAFLSGTD  >NlugCSP7  MASASSGTTSTTSAPKTAESASAKSSSKDEIPDQTFDRYINNERYMLMQYECLMGNKPCDHVGRKLKAAVPLVVRGLGCPKCSQREEDQMKRIVSHVQRSYPDKWQKLIKKYGN  >NlugCSP8  MSSTMLVFVAVLCFSAVLAKPADKYTTKYDNIDLDEVLSNQRLFDSYFKCLMGGKCTPDGQELRDALPDALATACEKCSEKQKEGTEKVMKFLIEKKPTEFAELEKKYDPQGTYRQKYKAEADKRGYSV  >NlugCSP9  MKSQQLLVSCLFICTWLVVLMAPSANAAPKEKDPERKALYRLEYIDIEKVLDNNRMLTNFIRCFLRQGPCTPEARDFRKLLPKLAKTMCSDCTARQRYIIKKVFKHLMEERPKEWELLMDRFDPQRKYAERLDTFMVDMTTRAPVTSSPMPSSPVTLTSSSVTMSSTTQRVIEILRTSTDMSNESRPAS  >NlugCSP10  MFMLLACSELGSGQQQQNVDNIEMSIYDKMFENMDVNSLLKNHRLVDSYLKCFLNEGSCTHIGHEVKMMIPEVIKSRCGTCGENQMRALKAGLRLFIVLRPDDWQRFLDVYDPDRKEWPHIKAFMDSDD  >NlugCSP11  MKSIILLVFVSMSAMVYRCRADEPSYPTSWDNVNIDEVLGNERLVQNYAKCLLEKGSCSPEGTELKKAIPDALKTGCTKCSDKQKAGAQKVIKWLVQKKPELWKEVVDKYDPSGEYTKKYEKEYQI |
| **SNMPs** |
| >ApisSNMP1  MLNNKILESKSLNPRSNMRQMWSHPPLSADFKIYLFNVTNPIEAQKGEKVIIKEIGPYVYHEWKEKENLIDDIDADTVEFSFKNTFVFDEMSTLPLTGDEIIVMPHLAMIGMVTMTKMMKPAALGLVNKAIPYLYPDQTSAFMMGTANDIMWNGLDINCTSEEFASVAICSQIRQNSESLHKISKDHFKFSLFGVKNGTIESNRYTVKRGYTSPATEVGQVIRFNDKHKMDVGPATSATRYTGQTPQYFNRSSRRTPTWPSFSGDICRSLTPDYVQETKYNGLNVFEYSAMLVKPEEKCYCLNKKKCLKPGALDLTNCSGAPIIATLPHFYKSEDYLNNVDGLSPQVEKHKIQMYFEPMTGTPLLGYKRLQFNIFLKRESKINVMKTLNEDEKLIPLFWVEEGIALNKTWTNQIKNKLYLPITIMKYVKYIFVVFGIVFIILAVVVNYNSVKTMEITPKY  >CsupSNMP1  MQLPKHLKIGAGTAAAGVFGIIFGWVLFPAILKSQLKKEMALSKKTDVRGMWEKIPFALSFKVFLFNYTNVEEIQKGGVPIVKEIGPYHFDEWKEKLEVEDHEEDDTITYKKRDVFYFRPELSGPGLTGEEIITMPHILMVSIATVVNKEKPAMLNMIGKAFNGIFDGPQHVFMNVKALDIMFRGTIINCARTEFAPKAVCTAIKKEASGLIIEPNNQFRFSLFGMRNDTIDPHVITVKRGIKNVMDVGQVVAVDGNPEQSIWRDSCNMYEGTDGTVFPPFLTENDRLESFSTDMCRSFKALYQKKTSYKGIKTNRYVVTIGDLANDPDLQCFCEAPEKCPPKGTMDLMKCMNAPMYASLPHYLDCDPEVQKKVKGLNPDVNVHGIDIDFEPISGTPMVANQRMMFSLVLQQIDKLDLFKDLPGTMTPLFWIEEGIALNKTFVKMLKNQLFVPKRIVGALRWLLVAVGVCGVIVTGIIHYKGSILGFTLPRGSATVAKVNPETNQPKDISVIGNAQSPPKVDM  >CsupSNMP2  MLAKHMKVFFLASLAALVLAVILAAWGFPRIVSKQIQKNVQLENSSVMFEKWRKLPMPLTFKIYVFNVTNAEDINSGAKPMLTEIGPYVYKEYRERTILGYGENDTVRYTLKKTFIFDAEESGPLTENDEVVVINFSYMAAILAVQEMMPSLTTVVNQALEEFFTDLKDPFMRIKVRDLFFDGIHVNCVGNHSALGLVCGQLKSDTPPTMRPTEDGTGYYFSMFSHMNRTESGPYDMVRGTEDIRELGHVVAYKGERSMSQWGDPYCGQLNGSDSSIFPPIDGGNVPQRLYIFEPEICRSMFATLVGKTTVFNMSAFHYSISSDVLAARSANPNNKCYCRKNWSANHDGCLLMGVMNLAPCQGAPAIASLPHFYLASEELLQYFASGINPDKEKHDTYLYLEPVTGVVLKGLRRFQFNIELRNIPEVPQLAKVPTGLFPLLWIEEGATLPDSVVKELQSSHKLLSYVEAARWILLVVAVIATVVSAVTLARSGVLPVCPRNSNSVSFILNPHPTVIDVNKVH  >MsexSNMP1  MRLARGIKYAVIGAGVALFGVLFGWVMFPAILKSQLKKEMALSKKTDVRKMWEKIPFALDFKIYLFNYTNPEEVQKGAAPIVKEVGPYYFEEWKEKVEIEDHEEDDTITYRKMDTFYFRPELSGPGLTGEETIIMPHVFMMSMAITVYRDKPSMMNMLGKAINGIFDNPSDVFMRVNAMDILFRGVIINCDRTEFAPKAACTAIKKEGAKSLIIEPNNQLRFSLFGLKNHTVDSRVVTVKRGIKNVMDVGQVVAMDGAPQLEIWNDHCNEYQGTDGTIFPPFLTQKDRLQSYSADLCRSFKPWFQKTTYYRGIKTNHYIANMGDFANDPELNCFCETPEKCPPKGLMDLTKCVKAPMYASMPHFLDADPQMLENVKGLNPDMNEHGIQIDFEPISGTPMMAKQRVQFNMELLRVEKIEIMKELPGYIVPLLWIEGGLALNKTFVKMLKNQLFIPKRIVSVIRWWLLSFGMLAALGGVIFHFKDDIMRIAIKGDSSVTKVNPEDGEQKDVSVIGQSHEPPKINM  >MsexSNMP2  MLAKHSKLFFTGSVVFLIVAIVLASWGFPKIISTRIQKSIQLENSSMMYDKWVKLPIPLIFKVYFFNVTNAEGINEGERPILQEIGPYVYKQYRERTVLGYGPNDTIKYMLKKNFVFDPEASNGLTEDDDVTVINFPYMAALLTIQQMMPSAVAMVNRALEQFFSNLTDPFMRVKVKDLLFDGVFLNCDGDSPALSLVCAKLKADSPPTMRPAEDGVNGYYFSMFSHLNRTETGPYEMVRGTEDVFALGNIVSYKEKKSVSAWGDEYCNRINGSDASIFPPIDENNVPERLYTFEPEICRSLYASLAGKATLFNISTYYYEISSSALASKSANPDNKCYCKKDWSASHDGCLLMGVFNLMPCQGAPAIASLPHFYLASEELLEYFEDGVKPDKEKHNTYVYIDPVTGVVLKGVKRLQFNIELRNMPRVPQLQAVPTGLFPMLWIEEGAVMTPDLQQELRDAHALLSYAQLARWIILAAAIILAIIATITVARSTSLISWPRNSNSVNFIIGPMVNDKMR  >TcasSNMP1  MIKGKVKSMINLNKGSEIRQMFVKVPFALDFKIYMFNVTNPMDVQKGALPVLKEVGPFCFEEWKEKVDLDDNDDEDVMFYNPKDTFYKANGPGCLDGSQMITMAHPLILGMVNTVVRTKPGAISLISKAINSIYGNPDSIFMTASAMDILFDGVVIKCGVKDFAGKAVCSQLKEAPDLRHVDENDLAFSFIGPKNATPGKRFKVLRGVKESHDVGRILEYDNKKEMEVWPTKECNQYKGTDGTVFPPYLTKEEGLASYAPDLCRSLVAVYSGDTKYDGIPVRIYTATLGDMSKNADEKCYCPTPDTCLKKGMMDLFKCAGVPVYVSLPHFYESDESYVKGVVGLNPNKKDHGIQILFESTTGGPVKAAKRLQFNMPLEPNPKLPIFANLPNTVLPLFWVEEGVALNNTFTKPLKDLFKIMKIVKIAKWLIMLGCLGGLGAAGYLYFSKKGEANITPVHKVKPAENGVSTLGGEVNHAMSDNEIEKY  >TcasSNMP2  MGCSCCTIKVLLVCVVISVALLIVSLALAFKVFPDLLESEVNKAVRLEDGTKQYDRFVELPFPVDFKVYLFNVSNPQQVLDGTEKPKLEEIGPFVYKQYRKKTILGKNEEEDTISYTQKETFEFDAEASKPLTEESVVTVLNPALMSIYQLAEDLHLAGAADTCIKQTFENNQGKVFIEANVRKLLFDGFSFCKNTSPGICGLVNDLICAIAATKRNSDLVLPDYSLIFSYLNYKRKPDDGKYTVKRGLTNIEKLGHIVAWNDSLYTKFWGEGTTCSEVKGTDSTLYPPRVTTDSAFYIYSTDICRFVKINYKGEESYKGIDGYLFETSEDTLRSSAPEEDCYCSKLSRDMEGKKSCFLDGVIDMQTCFGVPVLFSFPHFLWADNKYLSAVEGLNPVEEKHKTYLVVEPNTGTPLKGMKRIQLNGVIRPIVGIKSMLQTKRALLPLLWIEEGVSLPQKYVDELKSSYFDKVQIVDGVRYALIVISAILVGAFGIIILRKRSHAKHHV  >DmelSNMP1A  MQVPRVKLLMGSGAMFVFAIIYGWVIFPKILKFMISKQVTLKPGSDVRELWSNTPFPLHFYIYVFNVTNPDEVSEGAKPRLQEVGPFVFDEWKDKYDLEDDVVEDTVSFTMRNTFIFNPKESLPLTGEEEIILPHPIMLPGGISVQREKAAMMELVSKGLSIVFPDAKAFLKAKFMDLFFRGINVDCSSEEFSAKALCTVFYTGEIKQAKQVNQTHFLFSFMGQANHSDSGRFTVCRGVKNNKKLGKVVKFADEPEQDIWPDGECNTFVGTDSTVFAPGLKKEDGLWAFTPDLCRSLGAYYQHKSSYHGMPSMRYTLDLGDIRADEKLHCFCEDPEDLDTCPPKGTMNLAACVGGPLMASMPHFYLGDPKLVADVDGLNPNEKDHAVYIDFELMSGTPFQAAKRLQFNLDMEPVEGIEPMKNLPKLILPMFWVEEGVQLNKTYTNLVKYTLFLGLKINSVLRWSLITFSLVGLMFSAYLFYHKSDSLDINSILKDNNKVDDVASTKEPLPSANPKQSSTVHPVQLPNTLIPGTNPATNPATHHKMEHRERY  >DmelSNMP1B  MQVPRVKLLMGSGAMFVFAIIYGWVIFPKILKFMISKQVTLKPGSDVRELWSNTPFPLHFYIYVFNVTNPDEVSEGAKPRLQEVGPFVFDEWKDKYDLEDDVVEDTVSFTMRNTFIFNPKESLPLTGEEEIILPHPIMLPGGISVQREKAAMMELVSKGLSIVFPDAKAFLKAKFMDLFFRGINVDCSSEEFSAKALCTVFYTGEIKQAKQVNQTHFLFSFMGQANHSDSGRFTVCRGVKNNKKLGKVVKFADEPEQDIWPDGECNTFVGTDSTVFAPGLKKEDGLWAFTPDLCRSLGAYYQHKSSYHGMPSMRYTLDLGDIRADEKLHCFCEDPEDLDTCPPKGTMNLAACVGGPLMASMPHFYLGDPKLVADVDGLNPNEKDHAVYIDFELMSGTPFQAAKRLQFNLDMEPVEGIEPMKNLPKLILPMFWVEEGVQLNKTYTNLVKYTLFLGLKINSVLRWSLITFSLVGLMFSAYLFYHKSDSLDINSILKDNNKVDDVASTKEPLPSANPKQSSTVHPVQLPNTLIPGTNPATNPATHHKMEHRERY  >BmorSNMP1  MQLAKPLKYAAISGIVAFVGLMFGWVIFPAILKSQLKKEMALSKKTDVRKMWEKIPFALDFKIYLFNYTNAEDVQKGAVPIVKEVGPFYFEEWKEKVEVEENEGNDTINYKKIDVFLFKPELSGPGLTGEEVIVMPNIFMMAMALTVYREKPAMLNVAAKAINGIFDSPSDVFMRVKALDILFRGIIINCDRTEFAPKAACTTIKKEAPNGIVFEPNNQLRFSLFGVRNNSVDPHVVTVKRGVQNVMDVGRVVAIDGKTKMNVWRDSCNEYQGTDGTVFPPFLTHKDRLQSFSGDLCRSFKPWFQKKTSYNGIKTNRYVANIGDFANDPELQCYCDSPDKCPPKGLMDLYKCIKAPMFVSMPHYLEGDPELLKNVKGLNPNAKEHGIEIDFEPISGTPMVAKQRIQFNIQLLKSEKMDLLKDLPGTIVPLFWIEEGLSLNKTFVKMLKSQLFIPKRVVSVVCWCMISFGSLGVIAAVIFHFKGDIMHLAVAGDNSVSKIKPENDENKEVGVMGQNQEPAKVM  >DnoxSNMP1  MGAPTTLSVIGIIFLLFGVFVGWFAFPKMIHKKILESKSINPRSAMRQMWSHPPIYADFKIYLFNVTNPEEAQKGEKIIIKEVGPYVYHEWKEKENLIDDMDADTVEFSFKNTFIFDEMSTLPLTGDEIIVMPHLAMIGMVTMTKMMKPAALGLVNKAIPFLYPDQTSVFMMGTANDIMWNGLDINCTSREFAAVAICTQIRQNSASLHKISNEHFKFSLFGVKNGTIESNRYTVKRAYTSPATEVGQVIRFNDKHKMDVWPGDECNKIYGTDTTIFQPFITKDTNLASFSGDICRSLVPDYVRETKYNGLNVFEYSAILVKPEEKCFCLNQKKCLKPGALDLTNCSGSPIIATLPHFYKSEDYLNNVDGLSPNVEKHKIQMYFEPMTGSPLLGYKRLQFNMFLKKESKINVMKTLNEDEKLIPLFWVEEGIALNKTWTNQIKNKLYLPITIIKYVKYIFVIFGIVFIILAVVINYNSVKTMEVTPKY  >DmelSNMP2C  MIHWSLIVSALGVCVAVLGGYCGWILFPNMVHKKVEQSVVIQDGSEQFKRFVNLPQPLNFKVYIFNVTNSDRIQQGAIPIVEEIGPYVYKQFRQKKVKHFSRDGSKISYVQNVHFDFDAVASAPYTQDDRIVALNMHMNAFLQVFEREITDIFQGFANRLNSRLNQTPGVRVLKRLMERIRGKRKSVLQISENDPGLALLLVHLNANLKAVFNDPRSMFVSTSVREYLFDGVRFCINPQGIAKAICNQIKESGSKTIREKSDGSLAFSFFGHKNGSGHEVYEVHTGKGDPMRVLEIQKLDDSHNLQVWLNASSEGETSVCNQINGTDASAYPPFRQRGDSMYIFSADICRSVQLFYQTDIQYQGIPGYRYSIGENFINDIGPEHDNECFCVDKLANVIKRKNGCLYAGALDLTTCLDAPVILTLPHMLGASNEYRKMIRGLKPDAKKHQTFVDVQSLTGTPLQGGKRVQFNMFLKSINRIGITENLPTVLMPAIWVEEGIQLNGEMVAFFKKKLINTLKTLNIVHWATLCGGIGVAVACLIYYIYQRGRVVEPPVK  >DmelSNMP2D  MIHWSLIVSALGVCVAVLGGYCGWILFPNMVHKKVEQSVVIQDGSEQFKRFVNLPQPLNFKVYIFNVTNSDRIQQGAIPIVEEIGPYVYKQFRQKKVKHFSRDGSKISYVQNVHFDFDAVASAPYTQDDRIVALNMHMNAFLQVFEREITDIFQGFANRLNSRLNQTPGVRVLKRLMERIRGKRKSVLQISENDPGLALLLVHLNANLKAVFNDPRSMFVSTSVREYLFDGVRFCINPQGIAKAICNQIKESGSKTIREKSDGSLAFSFFGHKNGSGHEVYEVHTGKGDPMRVLEIQKLDDSHNLQVWLNASSEGETSVCNQINGTDASAYPPFRQRGDSMYIFSADICRSVQLFYQTDIQYQGIPGYRYSIGENFINDIGPEHDNECFCVDKLANVIKRKNGCLYAGALDLTTCLDAPVILTLPHMLGASNEYRKMIRGLKPDAKKHQTFVDVQSLTGTPLQGGKRVQFNMFLKSINRIGITENLPTVLMPAIWVEEGIQLNGEMVAFFKKKLINTLKTLNIVHWATLCGGIGVAVACLIYYIYQRGRVVEPPVK  >SlitSNMP1  MLLPKELKYAAIAGGVAIFGLIFGWVLFPTILKSQLKKEMALSKKTDVRQMWEKIPFPLDFKVYIFNYTNAEEVAKGAVPILKEIGPYHFDEWKEKVDVEDHEEDDTITYKRRDVFYLNPELTAPGLTGEEIVVIPHVFMLGMALTVQREKPAMLNMVGKAMNGIFDDPPDIFLRVKAMDILFRGMIINCARTEFASKATCTALKKEAVSGLVLEPNNQFRFSIFGTRNNTIDPHVITVKRGIKNVMDVGQVVAVDGQTEQTIWKDTCNEYQGTDGTVFPPFLTENDRLQSFSTDLCRSFKPWYQKKSSYRGIKTNRYVANIGNLAEDPELQCFCPQPDKCPPKGLMDLAPCIKAPMYASMPHFLDCDPALLSKVKGLNPDVNAHGIEIDFEPISGTPLVARQRIQFNIQLLKTDKLDLCKDLSGDIVPLFWIEEGLALNKTFVNMLKHQLFIPKRVVGVLRWWMVSFGSLGAVIGIVFHFRDHIMRLAVSGDSKVSKVTPEEVEEQKDISVIGPAQEPAKINI  >SlitSNMP2  MLGKHSKLIFAVSMGFLVVAVIMAAWGFQKIVDKQIQKNVQLENNSMMFDKWLKLPMPLEFKVYIFNVTNVEDVNQGEKPILNEIGPYVYKQYRERTILGYGPNDTIKYMLRKRFEFDPEASGVLTEDDEVTVINFSYLAAVLTVHDMMPSFVGMVNKALEQFFPSLEDAFLRVKVRDLFFDGIYLNCDGDNAALGLVCGKIKSDTPPTMRPAEGANGFYFSMFSHMNRTETGPYHMIRGRENVYELGNIVSYKEQKVMPMWGDKYCGQINGSDSSIFPPIKEGNVPKKLYTFEPDICRSVYVDLVGKKEIFNISAYYYEISESAFAAKSANPNNKCFCRKNWSANHDGCLLMGLLNLMPCQGAPAIASLPHFFLGSEELLEYFGSGIMPDKEKHNTYVYIDPTSGVVLSGLKRLQFNIELRQIDTVPQLKRVPTGLFPMLWLEEGATIPASIQQELRDSHKLIGYVEVARWFLLTAAIIAVVTSAVAVARANALLSWPRNSNSVSFILGPSVTQVNKGN  >AmelSNMP1  MRFKKLIHDITTRFFHRGVHVILHSSFVKLQSFVTTGRKLHETLRVPVGRKCVTMKPKKLGIIGGSLLAFGILICAIAFPPFLRSQVKKQIALKDGSEMRELWSNFPVPLDFKIYLFNVTNPMEITAGEKPILEEVGPFFYDEYKQKVDLVDREEDDSLEYNLKATWFFNPSRSEGLTGEEELIVPHVLILSMIKLTLEQQPAAMGILNKAVDNIFKKPESVFVRAKAREILFDGLPVDCTGKDFASSAICSVLKEKDDALIADGPGRYLFSLFGPKNGTVLPERIRVLRGIKNYKDVGKVTEVNGKTKLDIWGEGDCNEFNGTDSTIFAPLLTEQDDIVSFAPDICRSMGARFDSYTKVKGINTYHYKADLGDMSSHPEEKCFCPSPDSCLTKNLMDLTKCVGAPLIASLPHLLGAEEKYLKMVDGLHPNEEEHGIAMDFEPMTATPLSAHKRLQFNLYLHKVAKFKLMKNFPECLFPIFWVEEGILLGDEFVKKLKTVFKTISIVGFMKWFTIVSGTCVSGAAAALFFKNKDKNKLDITKVTPQKGEEKKWPNQMTISTIQSAAVPPNLDAD  >RpadSNMP1  MGAPTTLTVIGIIFLLFGVFVGWFAFPKMLHKKILESKSLNPRSNMRQMWSHPPISADFKIYLFNVTNPEEAQKGEKIIIKEVGPYVYHEWKEKENLIDDINEDTVEFSFKNTFVFDEMSTLPLTGDEILVMPHLAMLGMVTMTKIMKPAALGLVNKAIPYLYPDQTSAFMTATANDIMWNGLDINCTSGEFAAVAICSQIRQNSASLHKISKDHFKFSLFGVKNGTVESNRYTVKRGYKMSPLDVGQVVRFNDKHKMEVWPGDECNRIYGTDTTIFQPFITQDTSLASFSGDICRSLVPDFLQETKYNGLNVFEYSAILVKPEEKCFCLNKKKCLKHGALDLTNCSGAPIIATLPHFYKSEEYLNNIDGLNPEVEKHRIQMYFEPMTGTPLLGYKRLQFNIFLKKESKISVMKTLNEDEKLIPLFWVEEGVALNKTWTNQIKNKLFLPITIMKYVKYIFVAFGVVFIILAVVVNYNSVKTMEVTPKY  >AspiSNMP1  MQTPAKLVFTATILAGTTVYFHNVFFPEFLAKTIKEGMLQAKTLKRDGEIRKFWEKAPISVDFKIWVFNVTNPEEAAKGAKIKLQQVGPYVYDEWKEKANVTDFPKEDAVSFHMLNTFVFNANKTETLTGDEIVYIPHPLIVSMASAVLRLKPEAISYVEMGIPWLFDDPKHIFIPVAVRDFLFDGIPIICNETHFAQAIFCSEFKRNPSGLVEKDEETFLFSIFGARNGTPDAMMLKVNRGLREPHLVGDVIEYNNSRVQHVWPGKKCNEIHGSDTTIIPSFVEKGDFDAFAADFCRVITSTFVKELDYKGIHAYEFTSEFGDPAESEDKCYCTTPDTCLKKNTIDLTLCTGAPFVVSLPHFYLSDPEYINGVGGLNPKKEDHAIYVLLEPMTGSPMVAYRRVQFNIMMVKTPEITLLQELPEQVTIVPLFWVEEGIALEEQYLSQLRLVSGIPSKINIFAIVLEITSGLLVIIALYLRFGRARKSKEISVAS*  >AspiSNMP2.1  MPLKLWSDVTIDRRFATKLSIIVGAIGLVASVAIRFIGLPLLIKFIVLHKTLLVEGTERFEKWVTIPFPLHFKVYIFNVTNPEEVELGAAKPILTETGPYVYDQFFKKRNITISDEYISYYLERTFQFNQEKSGADRKESDIVTTLNLAFMGTALHLEDKMPMSLPLLNDAAPFLFPPNQILFRTSTVKEFLFDGVLVNCTNATGAAFMICQGLKAVKPSTIREIKNSTNLLFSFFHHKNGTISKQYTVDSGTKDYRNLGQILAYDGKENNAIWLTDGCNKLRGTDTNIYHPFVTKEEKLEVYDPNMCRSMFVEFETETEIKGLPAYKYVLSLDNVADTNSVPANKCYCMRSKSVKLPPSCFKRGVFDPEFCTNQPAIISFPHFYGGHCDYVSFVEGLNPNKELHETFVNLEPNTGVPLSGAKRLQFNLRYGPIQKISVIQRNTTRGLFPILWIDEGAEPNDIVLTPLHTLQFTINMFGFLNWTLLVMSVICILFVPGALAVDERSWSALTRFSAGSRDTANADDSDMQTTKFSVYRRQGDANLNASYFGEPVAVDPISYRTVR*  >AspiSNMP2.2  MGTDWRWLYLNVLGFSLLISGAFIFWIAVPKIVVSEIEKQQALVNGSEAWERWVNTPLPIQFKIYLFNVTNPDQVMLGEKPRVQEVGPYVYKEYKYKTDLKIDSERDTVFYRQRSKFEFDQEATMPLSQEDQVTVLNPALMGMVETMNRIPYLRNITMPRLDRIIPDLYGKPQSVFATISVRTLLFEGLLIKCNHSMFIDDVMLVCSGTRGMLPMRAISETKDNNFVISIFGYKNNSDEGLMEVHSGITRPDLMGTLVSWNGRDRVSAWPEPCNKVTGADVTLPPSRPARHHQILIFSADICGAVGLEYVEDITFAGINGFRFSPVESFLESVDRFPENKCHCPGQIKKLTESQQCMEKGVLDVSSCLDAPIILSRPHFYKTSRVYSRTVEGLKPDPKKHDTFMDLEQYTGTPLRGYRRFQINMFLRPNDHVRLLKKVPFALVPLLWLDEVSKLNYL* |
| **ORs** |
| >AspiOrco  MEKKTIKRGLIADLYPNIRLIQLSGFFLSGYHEDNSPTLETVRGLYSWCTFTLCMFGYILILIYAVTMSYDTDQLTAATITCLFFAHSMVKFIYFSLKEKSVCRVLNAWNQENSHPLFVESNTRHKMRASERMRKLSYTVAIGSVLTYFGWIFVTLIDDPYKAMPDVENQNNTVMVKVPNLLVGSTFPWNYANGIGYYVAIGYQLYWLLLMIAHENLPDTLFCCTVIYACEQLKHLKEILKPLIEMSSDFDHGIPVKSSMLRVRSGDSDIPLIDDEKVPDETYQPVYNLIQEFGPMYQRKNIQNLQNSFDPLSKQEGAYVPSAIKYWVERHKHVVRFVNDIGSMYGLALLIHMLISTVTLTLLAYQATQITGVNVFGLSVIGYLLYTFTQVYAFCIYGNELIEESSSVMEAAYDCRWYDGSEEAKTFVQIICQQCQKSLHISGAKFFTVSLDLFASVLGAVVTYFMVLVQLN  >Aspi8804.0  VGYVLTAKMVGLGLVGFLSPFVRFVVKKEDPTFLLRRVWYPSWVYTSPILCWCAAVYEWILVFDTSLALMIPDLLYIYFTLTTVVKLQALGQFFATNFGGTSNYTKVDNFSNCKLKLFKERILVWHVLWRESNLINHTYKTAATWAVSVGVVNNTLMLYIAVTELRTFGFSLQVLIQSIIYFYHVLTGTFMMYATSYSSTMLQRAHDDLRQSLWRCPWFAESLKFKKLFLSFNVPLLRF  >Aspi15899  KINGIFVSVDYCTLLVVNALGLSAGAYEMRNSQTVSLIEMMFFSYSADCILSSYILFYSAARVEEAYEELRWALWDSLWYTEGSHFKKLFNLSQIVLSRPPKLKIFNFYNMTMHNFTLTLSNSWNFFLFLYKVTAKN*  >Aspi52227  ATGSGIEEIYSVGESTDAICERLTDGLKDLNILQWQKRNINDAFSFYFSWIHCGVLLANALLPTAAANEIRAHGRVTETAALFLTNSMSCMWTTFFISFMITKLQASFDDLLPALWECEWYKKDRQFKKLVQQADGMLRQSFELQAYSFYPLNLATFTEGVFNSYNFFTFFYELTGKK*  >Aspi1519  GTDMEKTPQDVQSFSCDLRDNTLRMRPSIHYDFARREYYIGDELLFYNFPGLVPCRRVCVSRMVLDHVCQRSRLLSVPFFVLLQYLYPVPVHNHDNSCTTRHIGGEFEEDYSYNEWNGIEKGALNDRRKTLVNRAFTPFLTALHAATLFANGILPFAVTSEALNAREVNTNVFMLSALSMTCFWVAFFISIFTSKLQKSYDNLLHSLWECEWFTEDREFKRLFIFSQGALKPSFTLKAYSFYSINLYNFTSCMLHSWRYFLFLYKTSRESK*  >Aspi26288  MYGKSGSSWDRKIINYHIIFAGLHENGKFSKHVIRFHYGMALFTAVYICLETIGISAKLASNQFTLIEVLLPFQYHAILCTNLVGLYSFIYKQPRVAACMRNLTEILYCSSLEEEAQMRKKHLKTLRVFLVSFMMMFYAFSLLPIVLSPIARFGLEVHSWYVLFPVWYPVDVLSSLKWFWTAYVCESVLLLYGCSCYASFCILFLHTTITTIVRLDVLSEYMRKTEPQVRAESWNRLRVQAGHQSEVENRSNEMTYASDKISKISYVNAERLNERLIDLNILQRQKVFINRAFTLYLSALHSMTLVANAIIPFAITFEVRNAGKLNANAVMISGHSMSCLWIAFFSSYLTTILQESLDNLQHSLWECEWYTGNMQFKKIFLRSQIALRQPLALKAYSFYRINLYTFTSVSMNFQNTSQNKNKE  >Aspi15455.0  MEESWELKIIGLHMVFTGLRENCNFHKYIVRFHYIMAFHTLIYVCLETMGIYAKLSSDQFQLAEFLLLLQYHLILSTNLVGIYTFVYKRHRVVSAIKDITDNLRCSFRTENAQIWGKHLKIFRVLLIVCLLAENVFNPIPILVGPIARFGLKVNSWYVIFPAWYPMDVLTSSWYWLAFVSEILMSWYTSVCYGNFCILFLYATMTTIVRFDVLGESMKKMTVPQTKTGLRNQLIVETGNESNLESKYLSYKIGKSADTQSKRLTGILEDLHILQRQKKLYSRAFSPYLSALHAMTFIADAIIPFGLTSEARNGGLLNMNTLTFSGHFVSCFWMAFFASYLLSKLHESLENLRHSLWQCEWFKAEIEFKNIFLHAQGALKKSFTLKAYSFYPINLHTFTQCAMQSWSYFLFLYQFSSRK  >ApisOr1  MGYKKDGLIKDLWPNIRLIQLSGLFISEYYDDYSGLAVLFRKIYSWITAIIIYSQFIFIVIFMVTKSNDSDQLAAGVVTTLFFTHSMIKFVYFSTGTKSFYRTLSCWNNTSPHPLFAESHSRFHAKSLSRMRQLLIIVSIVTIFTTISWTTITFFGESVWKVPDPETFNQTMYVPVPRLMLHSWYPWDSSHGLGYIVAFVLQFYWIFITLSHSNLMELLFSSFLVHACEQLQHLKEILNPLIELSATLDSSVHNPAEIFRANSAKNQSINGIDHDYNGSYVNEITEYGTKGENEPNRKGPNNLTSNQEVLVRSAIKYWVERHKHVVKYVSLITECYGSALLFHMLVSTVILTILAYQATKINGVNVFAFSTIGYLMYSFAQIFMFCIHGNELIEESSSVMEAAYGCHWYDGSEEAKTFVQIVCQQCQKPLIVSGAKFFNVSLDLFASVLGAVVTYFMVLVQLKZ  >ApisOr2  MDVMQKPERFILTPFQKFCIRWSVFFDSSSDRLSRIETVLRTIQFSTIMITSGMTMTSVLIADNKKALESFTYFVICVFMLAIITFAIRTKRFNRAMLLMVVDEFPGYNRPMPDVLKRKMAAIRTSYGDFTMKVIVSYLTLVLFEIPATAMVPLAAASLTDVKLGSQSTQMVVLWFPADTSQVGMYAVSYVIQFLIVVTVKFIITGIMCSFSFFVSQMISEFQILSAYVEHAVEIVEYDQSADKTTEQKLLDHVKNCVMLHDRLIYFKDQLNESYGYIILLELMFSTLYFCLSAFNMIFVGNRFVMIKGLLTLSNYLAELFIFCMYGSMVEDAHMGLLRASYSVAWYAQPVRFRQSLTMVMSRTQTPLQLTVGKVFIANLPLFLSVLKVSYSGVNALRAANAKZ  >ApisOr3  MKTSENITARKMSVYWLTLLLLNGINVYPGHGNRVVRIAAAAYPWMICAFWAFITTSVTTSLALRATSYQEAVEMLTYITGSSSTLALFAIGVHNRPGLHRMLDAVRRDFWDDGRRPAADVLFSRFVRTYGAILPIANVMMCMTPVIWAARNGDIESPAALIFRMWTPWTRLTTARYAVVYAAQFVVSLSVLTSISGMVFAMVLFVTEMQVQVDTLVDAVQDLHVDMWYGDGDGRPDAHPDRRRRAAFDGLVKCIKHHQALITYFNRFKSYFNLLFIVDILYIMVMTCLCASSVLMANGFSAFHIKMMSLLIIVVSQFFFYCLIGEQFSTMNQQIGDCVYFKLVKCKDPMLSRAGLLVILRTQKPLQLTAMGITTYTASLFTFTVTMRSAYAGLNVLYNSSZ  >ApisOr4  MTSIGKKNQRKFYQTLMTLAFFLDTSQYRYISRFVKQFYIFDWMVLVSVAAAFTILEGNYRMPFVMELIQYMIVGFYFTSIFVVFIIKKEAIMSNYNCIQTKFIQWSNKRALHSNAAYKRNIKTVKSLSIPLAILSLSIALGPLISTINDIGKLPLDNRAHFVLFWPTIVDTNKLSMYGIIYTLQVIFTIILYISVLSFNLGYMVFLNELITQFEMLLNGINDAFKYKMDKQFQTLFIDCIRHHQIIIKFLDDLKSYFKWMILIEIIVVQVILAILIYNLTKVNASLGYKVKIAGSILFNLLPICFHCHIGEVVLSLHTRLSNHIYNMPWYDMPNKNKQLIVIMLQRTQRDLTLSSALFSSERASRSLISKVIKQVYTILNVLLKT  >ApisOr5  MQRIDTINMFLQMTGCTDSKAMLYLTYFEFLITFYYLIATYASIVHFEQSVTIQLFALLCMLIECVILLNITFRLYHKNHIREMHQYSRRLGIPDSYRSVINVITKYHLIASNIFVVFPVTYAIFCDSVRVGDPFTFPFLDVLPMHTDNLAIYACKYLVYAISVYIAHVELCFINTTFIYYVGVLKHRLETIVQTIGEAFADNDEQKFKYAIIQHQKLLSYFNTMKKVFSKPILLSMSFNAIYFGLTTSFVIQAIRGYINQAIISICIASSAAAVINITIYTFYGSELMDLHDKILHVLFDNAFFYVSKSFKSSILIMITRVTIPLKFTVGYIFTINLNLLLKILKMSYTVLNVLLSSETIKPHKLSZ  >ApisOr6P  MADIVEIFIQKMICSDDGAGHGAKHISVSLFLAILTCLSLVYSTEDLSIRIYGLLFIIIEIVVCSLIAIRVYHQSQHRDMYQRSQKVGIPENYRWKIAMVIKYHLVMSNVLSVIPLLYTISLDWVRMGDPFTFASVDVLPIKTTNVTVYVCKYIVYALPAYFAHLEICFLNVTFMYNINTINYFIFRFFEDMKTLYEKPLLISIEISTLYIGLSGCTMIQVIQGFVDPIILGFCIVSGVAGFVTISVYCICASNMYDLVLQGMYRTTFFYPGMILWRHSFPGGGQCPGPTRPLPSVAYLGGWSPRPGPIVKLQRTSLTKCVKNHFMMVHDSRCVSTLDVZ  >ApisOr7  MAHAVDNFFQKTGRSDVRRYATIMTCVEFFTYCDLVVTLFFAISAFLSIVYSEEDQLSNRVYGFLWLFVEIHVFGLIVVRLYHQSQCRDMYDRSLLIEQGIPKNYRRTIAMVIAYYCIMSTVHVTVPMLYTISSDSAQVGDPFAFPFADVLPIKTTNPTAYVCKYIVYAFPVYLTHLECCFMNVTFMYFTGVVKRHFQILDQQVQEAVANEDEQKLKIAIEYHQESLKFFKEMETVYEKPLIMTIEFCGLYIGLTGYIMIQCIQGIIHPIILGLCIASSTASLITISIYCICGSNMYDLHDGILNSLFEHQSCYSRNKSLKHLILMMMKRATIPLELKAGSIFKINSNLLVKILKFTYTVFNLLLTSVNRQIKETAIZ  >ApisOr8  MTDIVEIFLQRLGCSDDGDGRGTRRAVFFTYCESAITLFFAVLTYLSLVYSSTGDNLSIRIYGLLCFVIEIYIFAYTAVRVYHQSEHHDMYQRSRQMGIPENYRRKIATVIKYHLVMSNVVLAIPMLCTILLDELRMGDPFTFPFADVMPIKTANVTVYVCKYILYALHTYFAHLELCFLNVTFMYSTGVVKRHFQVLDEQVEEAMVTEDEQKLKIAIIHHQQVLKFFKDMKTVYEKPLLLTIEGFGLYIGLCCCAIIQVIQGFVDQIILGLCMASCVAGFMTISLYCICASNMYDLQNGILNSLYEHRACYSRNKSFKRLNLIMMTRATIPLEFNVYSLFIVNLNLLVKILKLTYSVLNVLLTTINLKFKETATZ  >ApisOr9  MAHIVDIFLQKMVCSHDRGYGMVFFNYCELAITLFFTVSTYPTIADPTENLSIRLYGVLCLLIEVHIFAFIAVRIYHQSQHRDMYQRSQGVEIPGNYRRKIATVIKHYFIISNVFVAVSVLYTILLDWVRIGDPFTFPFIDVLPIKTTNVTVYVCKYIVYALPVYFAHLETCFLNVTFMYSAGVVKRHFQILDEQVEEAVVNEDEQKLKIAIKHHQQVLKYFEDMKTVNEKSILVTIEFCGLYVGLTSCFVIQVMQGFIHQIILGLCIVSSMACLMTIIIYCIYASNMYDLHNGILNALFEHRSCYSRNKSFKRLILIMMTRATIPLEFKAGSVFTINLNLLVKILKFAYTVFNVLLSSINRQFKETRMZ  >ApisOr10  MAHIVDIFFQNMGCSHDHGYGMVFFNCCELAITLFFTVSTYPTIADPTQNLSIRLYGVLCLLIEAHIFAFIAVRIYHQSQHRDMYQHLHGVEIPENYRRKIATVIKHHFIISNVFVAVSVLYTISLDWVRIGDPFTFPFIDVLPIKTTNVTVYVCKYIVYALPVYFAHLETCFLNVTFMFSVGIVKRHFQILNDQVEEAIVNEDEQKLKIAIKHHQQVLKYFEDMKTVYEKPILMTIEFCGLYVGLTSCFVIQVIQGFIHQIILGLCIVSSIACLMTIIIYCIYASNMYALHNGILNALFEHRSCYSRNKSFKRIILIMMTRATIPLEIKAGSVFTINLNLLVKILKFAYTVFNVLLSSINRQFKETAIZ  >ApisOr11  MADIVEIFLQRLGCSDDGAGHDTMRVVFFSCCEFTVSLFLAILTYLSLVYSKEDLSMRFYDLLLLIVEIVVCSLIVIRVYHQSQHRDMYQRSQKVGIPENYRRKITTVIKYHLVMSNVIVVIPVLCTISLDWVRMGDPFTFPSIDVLPIKTTNVTVYVCKYILYALPTYFAHLEMCFMNVTFMYSTGAVKGHFQILEERVEEAMATQDEEKLKIAIKYHQQTLKFFKDMKTVYEKSLLIAIEVSMLYIGLSGCTMIQVMQGFVDPIILGLCMGSCVSTFMTISIYCICASNMYDLHDGILNAIFEQQSCFSRNKSFKQLVLMMMTRATVSLEFRVYSIFTINLNLLVKILKFTYTLLNVLLTSVNRQFKETAKZ  >ApisOr12  MADIVEIFLQLLGCSDDGAGHGTMRVVFFSYCEFAVTQFLAISTYLSLVYSTEDPSIRIYGLLFFIVEIVVCSLIVVRVYHHSQHRDMYQRSQKVGIPENYRQKITMVIKYHLVMSNVLVVIPLLCTISLDWVRMGDPFTFPSIDVLPIKTTNVNVYICKYILYALPTYFAHLEMCFMNVTFMYSTGAVKGHFQILEERVVEAMATQDEEKLKIAIKYHQQTLKFFKDMKTVYEKPLLIAIEVSILYIGLSGCTMIQVVQGFVDPIILGLCIGSCVSTFMTISIYCICASNMYDLHDGILNAIFEQRSCFSRNKSFKRLVLMMMTRATVSLEFRVYSIFTINLNLLVKILKLTYTLLNVLLTSVNRQFKETAKZ  >ApisOr13  MADIVEIFLQKMVCSDDGAGHGTMRLVFFAYFELAVTFFFAISTYLSLVYSTEDLSIRIYGLLFFIVEIVVCSLIVVRVYHQSQHRDMYQRSQKVGIPENYRRKIATVIKYHLVMPNVLLAIPVLYTISLDWAQMGDPFTFPFADVLPIKTTNVTVYVCKYIVYALPTYFGHLEICFLNVTFMYSTGVVKGHFQTLEEMVEEAMVTEDEEKLKIAIKYHQQALKFFKDMKTVYETPILITIEVSILYIGLSGCTMIQVIQGFVNPIILGLCIVSCVSTFITISIYCICASNMYDLHDGILNAVFEHRSCYSRNKSFKRLILMMMTRATVSLEFRVYSMFTINLNLLVKILKLTYTLLNVLLTSVNRQFKETAKZ  >ApisOr14P  MADIVEIFLQKMVRSDDGAGHGTKHVVVFTYCEFAVSLFLAISTCLSLVYSTEDLSIRFYGLLFIIIEIVVCFLIVIRVYHQSQHRDMYERSQKVGIPDNYRRKIAMVIKYHFVMSSVYMTIPLLYTISLDWVQMGDSFTFPSADVLPVKTTNVIIYVCKYIVXSLPANFANLEICFLNATFMYSTGVVKGHFQILEEQVEEAMTAQDEEKLKIAIKHHQQVLKFFKDMITVYEKSLLTAIEVSILYIGLSGCTTIQVIQGFVDPIILGLSMASCVSTFMTISIYCICASNMCDLHDGILNAIFEHRSCFSRNKSFKQLVLIMMTRATVSLEFRVYSIYIIDLNLLVKILKLTYTLLNVLLTSVNRIQLKGTEKZ  >ApisOr15P_NC  NIFTKVRTQRRCKSWHDACGIFTCCEFAVTLFLAISTCLSLVYSKEDLSILFYDFLLFIVETVVCSLIVVRIYHQSQHRDMYQRSQKVGIPENYGRKIVKVIKYHLVMANVLSAIPVLYTISLDWVRMGDPFTFPFADVMLIKTANVTVYVCKYILYALHTYFVHLEICFLNATFMYSTGVVKGHFQIFEEQAEEAMATQGEEKLNIAIKHHQQALKFFKDMKTLYEKSLLIAIRIYTLYAGLSGCTMIQVIQVCGNNNVFDILKLTYTLLNVLTSVNRQFKETA  >ApisOr16P  MKDVDIHASDAMTTTALPGPMPVNANECGEDETAVDLTLFKTIGLHRMLDPGPAGRRLRATYKWIACLIVTIQLMQMVGLYASVNDLQRFASLAVVAFNELTCSFKGILMVTNADRMRAVLDVTLYRYTTCGHRQPANMRLTSATVSTLLRTFTMIGYCMLVVWIIAPLFTGVGYVQVQHSDGTTGAYRKTIDNMWVPGMSETVYNWPPVWATIYVTEVVMMTVDMVIWIMFDCYLITVCFVLNAQFRTLAAGYETIGSQRLILQRGVDFEKSMGGKSDDGDIDSLDYYEELIVHIKDNQNIIEKHDEFLEIVRPVVITQXVSSSISIVGLVFLIEILYFMGEPFTFGPVLRLIFGVITIIIQFYIYCYSFNYIEIAKCTLNFGLYSSNWTEMDLKFKKTLFLGMSMNSTHTKVMKLSPKSIINLEMFAAVMKMSYSVVSVILNSIKKZ  >ApisOr17  MTTTPRVTELTAPASEDLTIVDNRLFKAICLHQILDPTKGGNRYYRLAFMVVMWVSLSVQIIQLVGLYFAVNDLQRFAFTTTVIFNALLCLSKGYVLVVNADRLRASLEVARYEFTSCGARNQRLVRRSRAVLSTILRTFAVLSWVTCFIWALTPLFAMDEYLQVTNADGTVSRYRVTIYNVWLPVPATVYNETTVWSLVYAVEVIACFVNVFSWLLFDSYVVTMCFTFNAQFRTVSASCTTIGHHSDSFRSPPPHAPEGTSDDNNTFNCYDELINRIKDNQSIIKIYDDFFEILQPAILFQIIGGSYSVITLIFLTSLTYLMGFSIISIPVLKVFFGFLSVTFELFLYCYVFNHIETEKCNMNFGLYSSNWTAMDLKFKKTLLFAMNTNSSHRRVMKVTPMSIINLEMFANVMNMSYSIVSVLLNSRVQKZ  >ApisOr18  MTTPRVTAFTVPASSEDLTIVDNKLFKAICLHQILDPTNGGNRFCKLVLMAFMSVSLSVQIMQLVGLYFAVNDLQRFAFTTTTLSYAFLCMTKDYVLLAHADRLRDSLEVARFEFTSCGARDQRVVRRSRAVLSMVLRTFAMLSWSTCVIWALVPLFMMDEYLQVTNADDTVSRYRVTIFNMWLPVPVAVYNATPIWSLIYMVEVIACLFTSFSWLLFDSYVVTMCVTFNAQLRTVSASCATIGHRDCFASLSPHVCTGTHIIKIDDNSILSNCYDELIIHIKDNQNIIKKYDDFFEIIQPVVLFQIIAGSYSVITLIFLTALSYLMGWSIISGPVLKVFFGFLSLTFELFLYCYVFNHIETEMNFGLYSSNWTAMDLKFKKTLLFAMNVNSAHRRVMKVTPTSIINLEMFANVMNMAYSIVSVLLNSRVQKZ  >ApisOr19C  MRSSSATVVDVMLFKAIGLYQLLCPADRGGYSVRSRRALMTALGLSFALHSFQVPYLYYALNDLQRFAYMAAVIIYGMMCSFKGYVLVTNADRLWLVLNAADYGYTGCGHRDPSRLRRCRATLSALLRTFVALSYGTLIVWIVLPFFVDEYTGITNSDGTVTRYRTTIHNMQYPISLAVYNSRPVWALIYVTELYVCIVNVFIWSLFDCYLVTMCFVLNAQFHTMSAGYGTLGIRRTGSSPPDTTFAGVRRIKFDEIESNHYSDLISHIQDNQNLIKMFDVFFEVVRPVVLVQIANGSYSVISLIFLTALMYLMGVPVLSAAFLKFICGLISLTIELFIFCYGFNHIETA  >ApisOr20  MRSSSATVVDVMLFKAIGLYQLLCPADRGGYSVRSRRALMTALGLSFALHSFQVPYLYYALNDLQRFAYMAAVIIYGMMCSFKGYVLVTNADRLWLVLNAADYGYTGCGHRDPSRLRRCRATLSALLRTFVALSYGTLIVWIVLPFFVDEYTGITNSDGTVTRYRTTIHNMQYPIPLAVYNSRPVWALIYVTELYVCIVNVFIWSLFDCYLVTMCFVLNAQFHTMSAGYGTLGIRRTGSSPPDTTFAGVRRIKFDEIESNHYSDLISHIQDNQNLIKMFDVFFEVVRPVVLVQIANGSYSVISLIFLTALMYLMGVPVLSAAFLKFICGLISLTIELFIFCYGFNHIETAKSVLNFGIYSSNWTEMDLTFKKTMLLTMKMNSSHKRAMKVSPNSAVGLEMFARVMNMSYSTVSVLLNSRSZ  >ApisOr21  MIGVHQLLRPDEYGQDNDLYRTAAKVIVGLTLVLQSMQVCRLYLARHDITMFAYIGVMIINGLMCLLKGYMVAAKADQMSATLTAANYAFTKCGGRDPSKLRLCRARLSAILRTFVGLSFGTLIVWLTMPWFMASDYDDQPFIWGVVYVIESIILTVNVFCWTSFDCYLVTMCFVFEAQFCTMSTGYETLGRRRTGAKSSAPQKLGNASTINNVKISDVNYDDLTSHIRDNQNIIKQYDAFFDVVRPMVLIQIANGMYSIIMLIFLTLVTHLSGYSIFSAPILKFVCGLASLTIELYIYCYGFNHIEDGKSTVNFGLYSSNWTEMDLKFKKTLLLAMTLNSAHKRVMKVSPNSIVNLEMFTGVMNMSYSIVSVLLKZ  >ApisOr22  MDVKLFKAIGMYQLLHPVECGLNSDLCRKTAMMVVGLTVGLQLMQVFRLYLARHDIPMFANMAMLVVYGFMCLLKGYTLANHADRICITLEVARYAFTDCGRRDPSLMRRCRARLSTILRTFVGLSFGTLVVWLVMPWFLASEYDGKPLIWAVVYVVESIILTVNVFCWTSFDCYLVTMCFVFEAIFRTMSSGYEKVGQVKANVTLTFPSHYDDLISHIKDNQKIVEKYKTFFEIVTPTVLLQIADGSYTIITMIFLISIAYLNGNSILSPMILKYVCGLVSLTIELYIFCYAFNYIEDGRSTVNFGLYSCDWTDKDLKFKKTVLLAMSMNSANKQVMKLSPNSIVNLAMFSRVMNMSYTIVSTLLSZ  >ApisOr23  MNLNDEQNYIVNLKLMKITGFYHLISPRAPKYFGFNVYKVTAAIEVMTGIFSIIMLFLSSYYYLDNTNELMSHFMLVVAIFFSTLKIFWVSRNSETIWNNMDMTCINFLLYTGHKKEILKKARAKSISTTILFVILWSSVTVAWSISPFFVKDVYLNIKFKDETRRFRYNSLNYVYPISEEFYNEHFLYFYVVEMLSVVFWGHGTVAYDTFVISICITIAFQLKTIAVSYISLNDKKGDIKNLKDNDLEAMFNLKLLIQDQQNMFKKIKEIYKIFEPVTFVQLAAQSMLIILQAYMIFINHYNGFSLLSVPIIKLIVTVAPNIIHLFITCYLYTNINHQQDSMNFALYSSDWTAMSINYKKMLLFTMRMNDAEKLKLKISLRKIVNLEMFASVMHLTYSIISVLAKSYGNTNTKZ  >ApisOr24C  MELQDENSIINLKFMKITGFYQLIRPSDCSKLFNMNIFKMLFIAQILILSITTIMCLYSIYSCVNDVNQVFNYSIMIFATNFAIYKYYFIIKNAKTIWNFAHNMMSTNLLCYKDHTKEVYKVARTRCSTITLISLALWSSILLYWSLLPFSNNNTYLKVKFEDGVHYYRLNALGVVYPVTDIFYNKYFHVFYIIEIVLLILWCKMMWVFDILMISVCISIEYQLKTIAESYRSLGLNHNELMSNKKSTMSVEAISDLEVLIQDQQNIYEKMKNMYQILKPFTFIQVAAESFQIILQSCMILKFYLDGSLSLILFLKFLLPGITYSCHLFLTCYLFSFVNEQKESMNFALYSSNWTDMSIKFKKLLLLTMRMNNAENLKMKISMKRMVNMEMFAD  >ApisOr25  MATGIKTVSKNEDNFMINMRLMKKTGFYQLLDSRSLKVFGHNVFKCMSVVQMSILSSVAFIFVANIYYFSDDINTVMMYSMLITSDVLSILKLYYILQNSDTIWNCIQMTSIDDLSYKYHDRRILEEGRSKSTSYSILIMFMWLNLIVSWSLGPLFVTNYFLIVEQNDEIYRYRFNIMNFAFPATDRFYNDNFMIYYGIEFITLVLWCHCTMNFDVLLLSMNITFKYQLKTISNSFKSMFDFKSLIYDQQRVIENMKNIYRVFRPVVLTQLASESLIIMLLSCIIMLNYFNGISLLSALNLRIFAAISTFLFHIYVICYLFDDVNEQKDSMNLALYSSDWTTSDLQHKILLLHAMRMNNAENLRLQVTRNKIVNFQMFTYIQSYNYSLVVRIAIYIVIKSRVZ  >ApisOr26C  MKLKNEHKFMMDTRLLKIIGFYQLFNPRSPKLFGYNIFKCIAAIQIFIFTMTLFGLIVSIYCCLSDIIEGMRCFWLCVIGMVTIYKHSYIIYYSDIIWKCIQLTFAEGLSYKYYSRRIHENGRKKLELVLEFFIVLWFVNVLFWILTPFAVKNSYTIVKGRNEIYHYHLNIINVIYPVTDKFYNDNFTMFYTMECTIMFVWAHVTLIFDVFIIVICIATAYQLKAIEYSFNTLGN  >ApisOr27C  MVLKNEDKLMANTKLLKTIGLYQILNSSSPKVFGYNIFKCITIIEAFIAAAVVLGCILNAFFCLSDLPETTRYFTIGVMCTITFFKLCCIIGYSDTIWNCLHIITSVKYLTYKYHSKRMLKVGRKKSKSILILFLVVWITVYVNWMFMPIVIQNSYLKVEAGNLIYHFRTNIISLVYPATDKFYNDNFLTFYTMEFIIMLVGIHCTFMFDMLLILMCTTIACYLKTIANSFSTLGNGEHHFMIRYDETKLINAFKIIIQDQQKVIK  >ApisOr28C  MVLKNEDKLMANTKLLKTIGLYQILNSSCPKVFGYNIFKCITIIEAFSAAAVILGCILNAFFCLSDLYVTTRYFTIGVMCAINTFKLCCIIGYSDTIWNCLHIITSVKYLSYKYHSKRMLKGGQKKSKLILIFYLVVWIPVYVNWIFMPIVLQNSYLKVEAGNLIYHYRTNILSLVYPATDKFYNDHFLTFYTMEFIIMLVGIHGAFMFDMFLILMCTTIACYLKTVANSFSTLGNVEDHFLIRYNETKIINAFKIIIRDQQKVIENMNNFCKVIRPVILFQLAAVSSVIILLSIIIIMDYFNGFPLASLKSFTLIATLITYTLELYLICYLLNDVNEQ  >ApisOr29  MVFKNEPKLMENIQLLKTIGLYQILDSHSPKVFGYNVFKCVAVIEAFILTATVYASILNIFYCLSDINEATRYFTLCLIASVPTFKLSYIIGYSDTIWNCLHITSAEYLSYKYHSRCILEVGRKKLKQFLILFVILWIVVFIAWILTPFIVQNSYLRVEARNMTYHYRTNILNLVYPAPDKFYNANFIMYYNIELTISIVWAHSTIIFDILLISMCITIEYYLKTIANTFSTLGNVENQFMIRLDDTKIINDFKIIIQDQQKVIENMKNIYKVIRPVILLQIVAESSIIILLSSITIMNYFNGFSLVSPSNFRFITSIFIYILHIYFICYFLNDVNEQKDSMNFALYSGDWSGKSLKYKKMILYAMQMNSTDQMKLQVTKTRVVNLELFTSVMRTTYTVISVLSEQYAKKTZ  >ApisOr30  MVLKNEPKLMSNIPLLKMIGFYQILNSRSQKVFGCNIFKCIATIEACILIAGVFALILNAFYFLSDINEATSYFTMGVMVSVATCKLFYIIGYSDTIWNCLHITSVEYLSYEYHSRCMIDVGRQKLKSILIISMVVWITTSIGWLLTPLIIQNSYLRVEVKNEIYHYRTNIMNLVYPATDTFYNDHFIMFYIIEFIVPIGFIHCTLIFDILLISMCSTIACYLKTIANSFSTLGNVENHCMGHDEMKTLNNFKIIIRDQQKVIQNMKNIYKVIRPVILLQISAATSIIILLSSMTIMNYFDGLSLISPLNFKFMSTMLTYAMHMYLVCYLLNDINEQIDSLNFALYSGDWTSKSLKYKKMILLAMCMNSAIKLKMQVTMTRIVNLELFAGVMRTTYSIISVFSDQYAKNKIZ  >ApisOr31  METCNDHTCTINLNILKQCGFYQIFDPNSKKIFGWNVYRISFIALTVITQCLIGFGNCGFLFELEDTTDNIDLFLIIFSNSYFCLTEWKVVILIINRKKFLELLDVTDLIFLKSKQCRNNIKILCKHRIRTLQLTNLYFMFCIFVIIEWIIFPIMINSFIAHKTENRRLENVVNRRYPVDVNTYNKYYILFYVFEIIIGVKTVYLVLMVDILLLSIGWAIIIQYEVLAEAFKNIGYNENLQKDHDHDVDDYKYFKSILFDQQQLDSKVKLYFPIVKPIVLMHVAINSVLFIMLSNSFLMVFLSTESFTYKIVNLFKIGTGILYICLQLFLYCHLFDNINLKRKSVNLGIYSCNWTKMDLKFKKLLLLTMQINDANYITIKASTKTIVNLPIFANVLMTSYNIVSVMVKTMSKYRKTZ  >ApisOr32  MNTFKDQDVAINLKLLKQCHFYQIFDSSNRKVFGWNVHQISFVMFAVVVQCFVCYGNAGSVFEKDDVVTNIDYFLIFYTNIHTYLSLWKLIVYLYNAKKILKVFNVTRINFLTSETCCNYSEILHKYRDKTIRFTNWYIIFSIVVIIQWLIFPLVLNMFMISGNSNVRYKNIMNLRYDVSTHTYNQYIIIFVLMETTTLSFAMYFMVMTDLILISFCSAIITQQEVLIHAFKNIGHEDKSQIKYYEQLKSILRDQQHLNLKTQSFYSAMKPIVLLAVAINSTFIIILTYLFILVCLTTESDTSDTALILIKLGSSAVYISLELFLYCYLFENMNIQRERVNLSLYSCDWTKMNLKFKKLLLLSMQMNNANQMLIRASPKKIINLQLFASIISMSYNVVSVMLKTTTPKSSRZ  >ApisOr33C  MSSFKVHDVAINIELYKLLRFYHVFDPSNSKIFGFNFYRFTGILITVFIQSCVLFGLLGCFMEMEDSLDYIELFVFIFVNSSNFLSVMKICVFIYKANDTWDLFEVTRIQFLKSERCRKYRDEILEKVRDKSIKLTNFIFGFAFMTCIIWVIYPLVVNFFMLATDQINNQRYQNVFNMRYPVTINTYNQYYFVFYTIEVILAAFILYNSILIDTFLVSFCWVIIAQYEILTEAFGNIGYEDKFQDQDKDCSIKAYNDLKSLLSEQRRLNLKLKLYYSIVWYIVLTYVVLTSCSIITLTYSFIMV  >ApisOr34C  MDKLRVEEVAINLELMKRSRFYHIFNPNGTKIFNCNAYRLLLFLYGSIVNCIVVFSTLGFFVEMDDTMSFTDLFVAIFVLINFFLCYWRICVFMYNVNAIYDVLSVSRFDLLKSKHCCKNVNVLNDYRDRTIKITNYFFLFSSTVMSQWIIYPLVVIAFTMPEDEYGRFQNIMNLRYPVSTYTYNQYYFIFYLMEVMVAIFTMYAMIFPDILLMSVCWAIIAQQEVLTQAFKNIGHE  >ApisOr35  MGNLKVEEVTINLKLWMLYRFYHMLKTNNYIKIFNCNVYRLILFIYGAIVNCMVAYSIIGFFVEMDDIISNVDVFLVVFVMINFFFCSWRMCIILSKSNTICDVLTVSQLNFLTSKQCVKHSNVLYDYRDRTIKITNYFFVFSVIVLIQWIIFPIMLITFTESDIENIRLPNIMNLRFPVFTYTYNQYYFIFYLMEVTIATFPIYVIIVTDTLIMSFSLAIISQQEVLNRAFRSVGYEENSQSEYYEDLKSILEDQIQLNLKIKSYYSIVRPVILANVAMSSTCFIIVTYVFIVVCFSKEPNQILTIIKLGSSAIFICGQFFLYCYLLDSMNLKREYVNFALYACDWSKMDIKFKKLLLLTMRMNDANNFIIRASPSKVVNLQMFANVISMSYNIISVMLKSMDSNNQISEZ  >ApisOr36  MLYRFYHMLKANNYTKIFNCNVYCLILFIYGAIVNCMVVYSSIGFFIEMDDIISDVDVFLVVFVMINFFFCSWRICIILSKSHIICDVLNVAKFNFLTSKQCFKHLNALYDYRDKTIKITNYFFVFSVIVLIQWIIFPIIVITFMESDVENSRSPNIMNLRFPVSTQTYNQYYFIFYLMEVAIAAFPIYVIMVTDTLILSFSLVTISQQEVINRAFKSIGYEENSQSKYYEDFKSILGDQIQLNLKIKSYYSIVKPIILANVAMSSTSFIIVTYVLIVVSFSKESNQILTIIKLGSSAIFICGQFFLYCYLLDSMNLKKESVNFALYSCDWTKMDIKFKKLLLLTMRMNDANNFMIRASPRKVVNLQMFANVISMSYNIISVMLKSMNSNNQSTEZ  >ApisOr37  MKWLQDHEVAINLALFKRYQFYQIFNPNGSKLLNYDTYKLTNVMFIVAVTTYNIFSAMCFFTDTVDTIDSVDLLLMIFIYSIIIISLLKISVLLFNADQIWELFDLTRFDFLTSRQCRKNVGILCKYRDRSITITNLYQNYSTMVFIIWMITPLVLNTFVVVGGPNQRYHNIFNMQYPVSANIYNQYYYLFYLMEIAMGIFVLNYSMIVDNFLISLCWVIIAQYEVITTAFEKIGNDCELTTLQNEKNNNSFEAYEDLKSILMDQNKLYIKLKSFYRVVWIIVIFLIIIDSVLLIILTYSFVMICSSAESFSIFNILKISTAFFVFVIQLYLYCYLFDVLNDKKESVNFGLYCCDWTKMDLRFKKLLLLATKFNNANTLKIKSTPNKIVNLQLFSSVMTTAFNIVTVMLKTMNGKNZ  >ApisOr38  MSSLKSNEVAINLKLFKVFRFYHIFDPNSGKLCKFNVYHLAWYIINCVIGCILIYGLLGYFTEMEDVIDSIFHIQIMFCYLLYSLSLLKIITFLYKANNIWDLLRVTRINFLTSTQCQAHIGILHKHRNKSIKITNLISGFAIVTTLEWILFPLVLRLLSKTDASHSNKRFENIFNFRFPVTVCEYNNYYFIFYIMESFIAIFMLYAYVVTDVFFISVCYVIIAQYEIIKRAYEIVNCEQTSENNNENKNHNNIIVNDCCDDLISIVMDQQNHYAKLRLFYSTYKLIIVSTVVINSGSIIILTYASVVIFTSPETIPILSIVKLISAFTYMFFVLFFLCYLMECINNKIESVQLGMYSCNWTAMNIKSKKLLLFSMRMHNANKLMIKTTPNNIINLQLFNSVMMTSYNIVSAMVNTRSKZ  >ApisOr39  MFSCDFINRTVNMNSENLFNGGSVAFNLSTYKQLGYYQLLDPKGPHIYGYHLYRTILKIFLLIVQFITIFGVMGFFIEMEDTDPGKSNSFELIIILTNCSLSSLKIYTLISNSKIIWDLFDLTRIDFLRCSRHSKLITKNFVKRCKKSTTITKWIARSFLVGLILWLMGPFIANEEHTEPNTVHRHKNIINIKFPVTMKTYNNYYFVFYLMEVAVGFCIVYGSVLIDAYLMSFCWIISAQYQSVTKAFATFGYNKQGSPKDIYKDFKSIIIDHQNIYLKMKSFYAVVRPITLIHVFAYSCSLIMYAYVIVTIFNSKELFIIAEIMKIVMTVSNVTMEVFIFCYLFELIDNKKEDVNFGLYSCNWTGMDIKFKQLLLMSMKMNNANRFKLKASPDVTINRPFFANVIHTCFKIVSVLIQTQSIDLLNZ  >ApisOr40  MNTTDKKYAFNLTLFKTIGYYQMVDPNSKKIFGFNIYNVINITLVIFTSIMTVIGLSGFFYKTDNITSEENNFKDLQMLFYLSCIGLGNLKIAITVYNADAIWKLFNVAHESFLSNKYCKHDKYKLNNCGKQFARIFPWYFFMFIMTAFAWSVVPIVVNNHAASNETQNNENAYMTNIANMRYPITVKTYNTFYKGFYALEFIMVWYSAYGLVVFDLFIVALLQLLATHYEIISSAYENFKYKAENEDGKLRKEEIQKELVSIIFDCQTIYRKLETLYGFSRPIVLVYMVGDAIGMITMPFLIVMSYVQSGSSIFNTNVMAFSWTLFVVGIQSYMYCSLLQNLNERKEDVNFGLYGCDWTSLDIEIKKLILLAMRMNSSNNLKMKVTSTKFIDLPMFASIVRSSYSVTSVLINSNIDKITKZ  >ApisOr41  MNKQKIYDSNFTLFKLIGVYQMVDPHSQKIFGFNVFHFVSMVFIIFTTSMTILGLSGFFYKVHNTNYNNSDVDTIFIMFYTVCITIGNLKVMIIIFKARQLRNMLEITDESFLSNTFYKRNYYKIVKCGGQLSKFFNLYFSFLLITLTSYAIVPIVLNAHFIDGTTQNTETIQKINIVNLKYPFTVETYNAFFKIFYASECIMLFYIGFGVFALNLFSMTILVIISAQYKLLASAFEVLEYRVNDEDDSLLSDEKLLETFISIVSDNQIIHKKIKMLYDIIRPVGLIQLMADALGMICMPYLIVVYFLEYGSLFNPETMKFVFTFGFAGVQSYMYCSLFQRVTDRREEVNFGLYCCDWTGMDIHMKKLILFTMQMNSSNKLKMNLTTNKCINLPLLSTIIRLSYRISSVMINYNINKZ  >ApisOr42  MPNSSEECVMSSSMAKCTGLHYIIDPEGPTVGGHNVFHVTVMVMIGFTVVCLSMCPFGLYYWANDVTQCIFLLITIVNFSFGCFKAFTLVRHSDDICRCLDVTRFDFSSGAIMSDPDSARFFRKCRDASSTFTGWFAASSHFVLLVWTLLPFVVVGKGVEINNRDGSTSYYHFNPYNMYFLVSSETYNRLHLVFHLVEWAFGLCFVLIMVAFDTFMVTLCVAITCQMRGIGNAYSKLGHDRCATASNVCSDGGIESNKSNNEYLRDLKLIIKDHQAVLGKMNDFYKIVGPVILPQLIVASFTIIFVSFIITRNYFNGMLLTSTQSLKMCCFPIFFYQIYYTCHAFGNLSHQKNVMNFALYSSDWTQMEIKFKKLLLLAMQMHDANKLDMKLTDKLVINLELFTRVINMCYSIFSVLVNSQLKIADKQZ  >ApisOr43  MDSKQEKQYIFNMKLARIMGLYQILFPNSTSFFGYNIYHVVTVFFVSFTFAISMLFPIGLLYLRNDIIAIMYYMGCISNFLLSCFKMVNILYHSKDIWKCIDVTSFNYILYKHYDRNVFKNWQTRSIRITYIYIVIALFAFFCWIFSPCIMNKSVIAIRNIDGSYSKYRMNIFNLYLIASNETYNKNFYIFYVIEIIISICYVYFTIVFDVLMLLVCFAISYQLETISNTIKSLGHEIYTRDNIRSGNSIKLKEKHGILYNDLITIMTDHQNVLKKLNDFYNIFRSITLTQIFIASSSHVFIWFIAAMSIDEGDNADSILSFKLFIVLPLINFQLFMTCSLFGTINEKKDSIIFALYSSNWTNMDLKSKKMILFNLTINNASQLKMKFTNTKIVNLEMFSHTMRFCYSIFSMLINYNKNKMKZ  >ApisOr44  MRFRYIFLQVTKRYITSTLIIMNIRGSNSDSIFNLKLAKIFGFYQILDTETVTFLGRHNVYYGIFVFLIVYQWLLSAIVFLNGLYYPVNNSNIIQDMFYFGFTVNMLYGNYKMYIILNRSKVIWDCLSITKFDFTSYGVQGRHTLNVWRNLSIKYTNIYVMFYITVSILCVGFPVVFSNSFIIIKNHDGLSSAYRLGLVNLFLFISEETYNTHFYVFYIVESLCLIINTLFIIIFDTIVNTLAIALTGQLQMISNAFESVGHKSLHFPNDNVDNKIKLPNENIKYMDHYKDLKTLIIDHQNILKKYDEFLSIFRPTMLLQVFVVSCSIIFLWFIFLTSFIEDDFTQYMALTSMEAIFGIPFCTFQMYMSCFVFNTLNIKKDSITFALYSSNWTEMDMKFKKLILLTMRMNDAHQQKLQYTKTKIINMEIFYHTMRVCYTIVNVMINCKKEKMVQQZ  >ApisOr45  MNIHGSNSDSIFNQKLAKIFGFYQILDTKTVTFLGRHNVYYRIFVFLIVYECLLSAIVILNGLYYPVNNNNIVQAMFYFGFVVNMLYGSYKMYIILSRSKVIWDCLSITKFDFTSYGVQGRHTLNVWRNLSIKYTNIYAIFYLTISILCVASPVVFSNSFIIIKNHDGLSNAYRLGLINLYLFVSEETYNAYFYVFHIVESLGLVINTLFIIIFDTIVNTLAFALIGQLQTISTAFESVGHKSLHFPNNNIDNKNKLPNENIKYMDHYKDLKTLIIDHQNILKKYDEFLSIFRPTMLLQVFVVSSSIIFLWFIFLTSFMEDDFTQYMALTFMEVAIFGIPFCTFQMYMSCFVFNTLNIKKDSITFALYSSNWTEMDMKFKKLILLTMRMNNAHHQKLQYTRTRIINMEIFFQTMRVCYTIVNVMISCKKEKWFQQZ  >ApisOr46P  MQYMYTFLQVTKIYITXTLIIMNIHGNSDRIFNLVLAKIFGFYQILDTETVTFLGRHNVYYKFFVFLIVYDCLISVMVFLNGLYYCINNIVEAMFYMGIVGNAMYANYKMYLILNRSKVIWDCMSVTKFDFTSYGVQGIHTLNKWRNISIKYTNIYMMLFLTLLFFYVLAPVVFSNTFTTMKNHDGSSSAYRLNVINLYLFISEEAYNTYYYVFSIIESFSIVIIVWFVFIFDTIVNTLAIALSGQLQMISSAFESVGHKYLHSPNINIDDKIKLPNENIKYKALNNDLKTLIIDHQSILKKYDEFLSIFRPTMLLQVFVLSYSIIFLWFIFIMCFIEEDVTQYMVMTSVKAAFGIPFCTFQMYMTCNIFNTLEAKKDSITFGLYSSNWTEMDIKLKKTILLTMRMSNAHQKRLQFTRTRIINMELFYETMRVCYTIVNVMLNCKKGKLVZ  >ApisOr47  MDIQNEKHHVFNIRLANLIGLYQTLDPETVKFRGRNVYQIFVAFVALYLLVISLGLFAGCLHLWTYNTATSLLDLLITTNSFYASYKMWIVVYRSNEIWDCLSITRYGFTSLNNRKWNGHDILDRWRARSVRYTSLLAGAYFLTIVFYVGCPLVFGAAVIPIKNQDGSIGSYRLNVINLYLFVSDETYNEYYNTFFFIEALFIVGLIITCLLFDTLLLTLCLGICCQIQMICSAFESVNHNSPSDPHSSAIDNNDEKQIISNEHDLIHDELITIIINHQAVIKKFELFLTIFDRVMLSHIFVSSISLIILWFNLIMSFFNDGTFAISGDTTLKTIVAIPSFLFQIFMVCYLFEDIHNQKDSIVYALYSSNWTEMDMKCKRLILLTMQLNNANQKKLRFTRTKIVNLEMFFKTTGHCYTVVSVLMNYINAKNVZ  >ApisOr48  MDIQNEKHHVFNIRLANLIGLYQTLDPETVKFRGRNVYQIFVAFVALYLLVISLGLFAGCLHLWTYNTATSLLDLLITTNSFYASYKMWIVVYRSNEIWDCLSITRYGFTSLNNRKWNGHDILDRWRARSVRYTSLLAGAYFLTMVFYVGCPLVFGAAVIPIKNQDGSIGSYRLNVINLYLFVSDETYNEYYNTFFFIEALFIAGLVITYLLFDTLLLTLCLGICCQIQMICSAFESVNHNSPSDPHSSAIDNKDEKQIISNEHDLIHDELITIIINHQAVIKKFELFLTIFDRVMLSHIFVSSISLIILWFNLIMSFINDGTFAISGDTTLKTIVAIPPFLFQIFMACYLFEDIHNQKDSIVYALYSSNWTEMDMKCKRLILLTMQLNNANQKKLRFTRTKIVNLEMFFKTMGHCYTVVSVLMNYINAKNVZ  >ApisOr49C  MDKQNEKHHVFNIRLANLTGLYQVLDPGALKCRGRNVYQIFVAFIALYLLVISMVLFVDCLHLSTYNMSTSLLDFLVTTNSFYACYKMWIVIYRSNEIWDCLSITRYGFTSLGNRKLTGHNILDHWRACSVWYTSLLAVAYFLTMVMYVGCPLAFSDTIIPIKNYDGSIGNYRRNVLNLYFFASDKTYNEYYNTFFVIEALFIAGLVIIYLLFDILLVTLCLGICCQIQMICSAFESIYHSSPSDLHSFEIDNNDEKQIISNERDLIYDQLITIIINHQAVIKKFELFLTIFER  >ApisOr50C  MDIQNEKHHVFNIRLANLTGLYQILDPGALKCRGRNVYQIFVAFIALYILVVSMALFVDCLHLWTNNTSMSLLNFFVATNSFYACYKMWIVIYRSNDIWDCLSITRYDFTSLSNRKRIGHGILDRWRARSVWYTSLLAGLYFSTMVIYMGSSLAFCNVLIPIKNHDSSFGNYRLNILNLYFFTSDETYNEYYNTFFVVEAWLSVAVTIFYILFDILFVSLCLAICCHMQMIFTAFSSVNHKSLSDFHSSSIDNTVEKHIISNEHELIYDELITIIIDHQAVIKKFELFLNIFERVMLSHIFVSSISLIILWFNLIMRFFNDSTFAISGDTTIKTIVAIPSFLFQIFMACYLFENVHNQKDSIRYALYSSNWTEMDMKCKKLILLTMQMNNANQKTLRFTRTRIVNLEMFFK  >ApisOr51  MDIRNEKNHVFNIRLAKLTGLYQMLDPGTTKFLGRNVYQMFVALFLLYLLVSAVALMVGCLHLWTYDTSMSILDFFLAINSFYACYKMCIIFYRSDDIWECLSITRYGFTSSSLRKRNGHGDVLDRWRARSVWYTSSMAGAYCFSFVFYIRCHLIFGDAIIPVKNLDGLIGNYRWNVLNLSFLTSDETYNEHYNTFFVIEALFIVVITIFYLIFDILFLTLCMAICCQMQMICDAFKSVNHKSLGDPHSSAIDNTDEKQIITSERDLIHDELITNIINHQAVIKKFELFLTIFERVMLSQIFVSSISLIILGFNLIMSFFNDGTFSISGDTTVKTIVAIPSFLFQIFMACYLFENIHDQKDSIKFALYSSNWTEMDMKCKQLILLTMQLNNANQKKLRFSRTKIVNMEMFFKTMGHCYSVLSVLINYMNAKNDZ  >ApisOr52  MDIWNENNHVFNIRLAKLIGLFQILNPGSIKFLGRNVYHIVVAINMLFVCIVAMVFFASGVYYWSDGVLVGVDYGWKGITALFLTYKMWKVVYHSNDIWDCLTITRYDFTSQNLRDRQILDRWRERSVWITNTMAIAYLMSLVILLSGSLMFRHDTLTVKNHDGSVGNYRQNIMNLYFIVTDETYNAHYKTFYFIEMLFTVGGGTLFTAFDVLLVTLCLAISCQFQVVNAKFESVGYKSLCDSRTKISDNKDEKQNISNEHDLIYDELISIIKDHQEVIKKYYELLTIFKRLMLLHVFYSSISLIVIWFIFIMSFTTEDRFFAWEVTTMKIICLIPSFSFHLYMTCSLFDNLHKQKDSIIFALYSSNWTEMNMKCKKLILLTMKMNNANQKKLKFTRTKIVNLELFYNIIRSCYNVVWFLINYIKVKYELZ  >ApisOr53  MDVWDKNNHVFNIRLAKLTGLFQVLSPESIKFLGQNVYHIVVTVILLYMGIISMILIVSGLYYWADNILLSVDYGWKGITALFSTYKMWNVVYHSNGIWDCLAITRYDFTSHGLRNRHILDLWRERSVWITNTLTIIYVSTTVLFAGSSLMFHDNISTVKNHDGSVGNYHQNLFNLYLIVTDETYNAHYETFYFIETLFAVGLATLFIAFDLLLVTLCLTVSCQMQVVNVAFESVGNKPLNDPHTPSIDNADEKKNISNEHDVIYDELISIIMDHQAVMKKYNDLLRRFKRPMLLQVFYSSTSLIVIWFCFLMSFSTEDRFAASEVTTIKIICSIPSISFQLFLVCSLFDNLHKQKDAIIFALYSSNWTEMNMKCKKLILLTMKMNNANQKKLKFTRTKIVNLELFCIIIHNCYSVVSVLIKCIKVKYEZ  >ApisOr54  MDIRDDQNHVFNVTLAKLIGVYQTLDPKTTKYRGINVHRIVMAFIILYIGVTAVILTLSGAYYWTNNMPLSVDCYWKGIVSYTMCYSMWLIVHYSNDIWNCLSITCYGLTSNSLRDRHILDGWRELSVLITTILTFVYFMSAIIFYISSLALSNDLLPVKNHEGLVRNYRYNLFNLYLFVSEETYNVHYNIFYMVEALGVVSLLISFFVFDILLVTFCLAITCQMQMICAAFESVGHKSLANDLSSIDSRDEKKEITNKHDLIYDELKTIIMDHQEVMKKYDMFLTLFKRVLLIQMVVFSVAFIITWFCFIMSFSNEERFKSPTIFIIKIFCGIPPNVFKLFATCYLFEKLHNQKNSIVFALYSSNWTEMDMKCKKLILLTIKMNNANYKKLKFTTTKIVSLEMFFKTMRDSYSVLSVLINYIKNKDESIZ  >ApisOr55P  MDIRDDQNHVSNMVLAECTGLYQMLDPQTIVRCRGLNVYHIFLLSTTLFMCVVGVIMTISGVYYWPNNMPLCVDYYWKGIIPLYMCYSMWVIVHYSNDMWNCLSITCYCFTLHSIRLXLTTILTVTYIITSVIIYFVSSLAVSNDILQVKNRDGSVSNYRYNVINLYLFVSDETYNXHYNMFYIGEALFIVFITIALFVFDFLMVTLCLAIHCQMQMXCTAFESVGQDDIPLVDCQDENKKSPNEHDLIYDELKTITMDHQAVMKKYDAFLTIFERVLLLQMLFYXIAFIIIWFCFIMNFSNDERFKISRIFTVKICCVIPTNLFWNLLLKDSIIFALYSNNWTGYDMDMKCKKLILLTMNITNANYKELKFTITRIVNLEMFFKTMRDSYSILSVLINYIKNMDKZ  >ApisOr56P  MDIRDDKNHVFNTTLEKCTGPYQMLDLSTLQFRGKNVHHMVLIFITLFMCVISVILSVSVVYYWTENIPLSIDYIWKSFFSLYMCYSMWVIVHCSNDIWNCLSITCYGFTSHSLRDRHILDRWREQSVLLTTILTVTYVTAVIIYVVGSLALSNDIQSVKNHVGSVGSYRHNLINLYLFVSDGMYNAHYNIFYMVEALCTISILIAFFVFDFLLVTLCLAVCCQMEMIXSVFESVGHKSLGDNLSLVDYRDEIKETPNEQDLMYDKLKTIIMDHQAVMRIYDEFISIFERVLLTLVVVLSIMFIVLWFCFIMSFSGDGRFRSSGIFIIKMFCAIPPYLFKLFAVCYLFGNLHDKKDSIIFALYSSNWTEMDMKFKKLTLLTMKMINANHKKLKFTRTKIVNLEMFFKAMGHCYAIISVLVNYIQSNVEZ  >ApisOr57P_C  MDIWNDQNHIFNITLAKLIDLYQTLDPETTKYRGKNVYHIVVAFIRYTLYWCNCGDIAVILNITSVYYWTDNMPLSIDNFWKGIISICICXPMCVIVHYSNGVCRSRGVTSHSLQDRHNILDRWRELSVLSTTILAVAYLTTMIIYFFSSLELSNDMLQVKNRDGSVSNYSFNVINLYLFVSNETYNAHYNMFYMVEALFCACIVIALFVFDFFLVTLCLAIRCQMQMVCTAFESVGHKSFGDDLS  >ApisOr58P_C  MDIREDQNHVFNITLAKCWISRPCNFVKNVYHMVLIFIMLFMCVISVILSVSLVYYXTNNMPLSIDYNIWKSFLSLYTCTYVCCSMWVIVHYPNDILWNCLLITCYGFISHSLXDRHIILDRWRELSVLLMTILTVAYLTKMIIYFVSSLELSSNILQVKNRDGSVSSYRYNLLNLYLFVTSGDMYNAHYNMSYMVEALFCVCIVIALFVFYFLLVTLCLVIHCQXMICTAFESDGHKSFGDNLSLV  >ApisOr59P_NC  LDPSEMDIRNEMNHVFNIKLAKFIGIYQVLDPGSLKYRGRNVYHIVVACLMLFMCLNSVLLDVSDLYYXDNISISVDCFWKADALLYLVYKMWIVFHWNDIWVCLYYDYVIMTYYYVXRYDITSFSFRNRHILNHXRERSVRLTTMFALMYLCFPAFYLGSTLAFRNDILPVKNHDGSVGFYRQNVMNFYLIVSDQTYNANYNTFYIVEALYLAMMVMVFLIFDIVLVTLCIALCSQMQMIFSAFESVGHKSLFDSHSPIGEYDYKVHLKVYCIKKSIHIKFIVFLLINLFNSXTLDRYSHVQHFLMTILFFYXKKDSIIFALYSSNWTKMDMKCKXLILLTMKMNNANQKKLKFTKTKIVNLEMFYKVSNSIITI  >ApisOr60C  MDFRNEKNHLINIKLAKITGLHQLLDPETVKYRGQNIYHVVMSCVSLYMCFISMILLLSGLYYWTGNIPISMNYFFKSVTTFYLIYKMWFLVRHSNYIWNCLSITCFDFTSFSNRHRHILDHWRDRLAWFTTTYATIYFTTTVSYLAITLAFSENKSPVKSHDGSIGYYRQNVLNLYLIVSDESYNAHYYMFYFVEALFGTFIGLFFFIFDFLLVTLCFSMCCQMQIICSAFESVGHKSLRVQHSPIWKGSGLEFFTWAPYYQVTPLYNAQLYM  >ApisOr61C  MDFRNEKNHFFNIKLAKMTGLYQMLDPKTVTYRGRNIYHIGMACVLLYMCLFLMIFILSNLYYWTVNIPISMDYFWKAETTLYVIYKMWFVIHHSNDIWNCLSITRFDFTSFTNRKRHILECWRERLAWFITIYATMYFTATVSYCAITLAFNENKSPGKSYDGSIGHYRQNVMNLYLIVSDETYNAHYYMFYFIEALFGIILGLLIFIFDFLLATLCFSMCCQMQIICSMFESVGHKSLHDHHSPI  >ApisOr62C  MDIRNEKNDFFNIKLAKIVGLYQMLDPKTVKYRGRNIYHIGMACVLLYMCLFLMIYILSCLYYWTVNIPISMDYFWKAEITLYVIYKIWFVVQHSNDIWNCLFITRHDFTSFGNQNRDILDYWRDRLAWLTIVYATMYFMAMFSYLAITLVFSDEKSLVKNHGGSIGYYRQNAMNLYLIVSDQTYNAHYYIFYFVEASFGIFIALLFFIFDFLLVTLCFSMCCQMQIICSAFESVGHKSLHDHHSLI  >ApisOr63P_C  MDIRNVKNHVFNIKLAKLIGLYQLLDPETVKYRGRNIYHIAMVCVLLCMCLISLILILSGLYYWTNNISISMDYFWKSEMTLYVSYKMWFVVCHSNDIWNCLSIARYDFTPINDQNRHILDRWRQRLVWLTTIYVVMYSTATAXYLFITLVFSQDKSPVKSHDGSIGYYRQNAMNFYLIVSDETFNTHYYMFYFVEALFVAFMGLFFLIFDCLLVTLCFCMCCQMQIICSAFESVGHKSLRDLDSQIGNYKSIKMTPNEHDLIYNELKTIIVYHQRLM  >ApisOr64  MDIRNETNHVFNIKLAKLIGLYQMLDPGAAKCRGRNIYHIGMACVLLYMCLVLMILVISGLYYWTVNVPISMDYFWKSESTLYVIYKMWFVVHHSDDIWNCLSITRYDFTSFSNRNRHVLDRWRERVSWSTTIYAIIYFTTCVSYLAITLAFSEVKSPVKNHDGSIGYYRQNAMNLYLIVSDDTYNAHFYTFYFVEALFGNLIGLFFFIFDFLLVTLCFSMCCQMQIVCSAFESVGHISLRDHHPPIDYTDENIKISPDEHELIYNELKTIIKDHQAVMEKYKDFLSLFRRVMLLHIFVSSLLVIAIWNTFIMSFSDDDRFQTSDVIVKKMFCVIPSILFQIYMVCYLFGNIHNQKDSIIFALYSSNWTEMDMKCKKLILLTMKMNNANQKKLKFTRTKIVNLEMFYKTMGDCYTVISVLVNYIZ  >ApisOr65P  MDIRNEKNDFFNIKLAKMTGLYQMLDPKTVKYRGRNIYHIGVACVLLYMCLFLMIFILSNLYYWTVNIPISMDYFWKAESTMFIMYKAWFVVHYSDDIWNCLSITRYDFTSFSNRNRHVLDHWRERVSWSTIIYGIVYFTTCVSYLIMTLAFSQIKSPVKNHDGSIGSYRQNSMNLYLIVSDETYNTHYYMFYFVEALFTIFLGFFPFIFDYLLATLCFSMCCQMQIICSMFELVGHKSLHDRHSPIDYTDENIMISPNEHKLIYDELITIIRDHQAVMKKYEDILTLYRRVMLSHIFTSSLLVILLWFTFIMSFSNDERFNTSDVIVKKLFCVIPSALFQIYMLCYLFGNIHDQMNIYSHCTYSSNRTELEMXKCKKLILLTMKLNNANLKKLNFTRTNIVNLEMFFKTMGYCYTVTSVLVYYIQTKYEYILFZ  >ApisOr66P  MDIRNEKNDFFNIKLAKMTGLYQMLDPKTVTYRGRNIYHIGMACVLLYVCFFLMIFILSDLYYWTVNIPISMDYCWKAETTLFVSYKMWFIVQHSNDIWNCLSITRYDFTSGTNRNKHILEHWRERLALLTTNYAIMYSTTVVSYLVFTLAFSEISIVKNHDGSIEYYRQNIMNLYLIVSDETYNTHYYMFYFVEALFSSFLGLFNFIFDFLLATLCFSMCCQMQIICSMFELVGHKSLHDRHSLIDGNIKISPCEHELIYDELITIIMDHQAVMKKYEDILTLYRXVILSHIFTSSILVILLCFTFIMSFSNDERFKTSDIIVQMFCLIPSILFQLYMLCYLFGNIHDQKDEIIFALYSSNWTEMDMKCKKLILLTMKLNNANQIKLKFTRTRIVNLEMFFKTMGNCYLVISVLVNYIKTZ  >ApisOr67  MDIRNQKNDFFNIKLAKITGLYQMLDPKTVKYRGLNIYRIVMACVLVYMCFISMILLLSGLYYWTGNIHISIDYFFKSVTTFYLIYKIWFVVQHSNDIWNCLSITRHDFTLFGNQNIDMLDHWRDRLAWLTIIYATMYFMGMFSYLAITYVFSDEKSLVKNHDGSFGNYRQNVMNLYLIVSDQTYNAHYYIFYFVEASFGTFIALLFFIFDFLLVTLCFSMCCQMQIICSAFESVGHKSIRDCYSPIDVNLIISPNEHDFIYDKLITIIMDHQAVLKKYEDFLTLFRRVILSHIFVSSFLVIALWFAFIMSFSNDERFKTSDIIVKKMFCAIPSILFQIYMVCYLFGNIHNQKDSIIFALYSSNWTEMDMKCKKLILLTMKLNNCNQKKLKFTRTKIVNLEMFFKTMGNCYSVISVLVNYILKQDEZ  >ApisOr68N  DQTYNAHYYMFYFVEASFGILIALLIFIFDFLVVTLCFSMCCQMKIICSAFESVGHKSLGDHHSPIDGNIMISPYEHELIYDELITIIMDHQLVMKKYEDFLTLFRRVMLSHIFVSSFLVIAVWFTFIMSFSNDERFKTSDIIVKKMFCAIPSILFQIYMVCYLFGNIHNQKDSIIFALYSSNWTEMDMKCKKLILLTMKLNNSNQKKLKFTRTKIVNLEMFFKTMGNCYTVISVLVNZ  >ApisOr69  MDFRNEKNHFFNIKIAKLTALYQMLDPETIKFRGRNIYHIVTACVLVYMCLISMILLLSGVYYWTGNIPISMDYFWKSVSAFYIIYKTWIIIRNSNDIWNCLSITRYDFTSFSDRNRHILDRWRDRLTWFTTIYATMYFTAAVTYLAITLAFGENKSSVKSHDGSIGYYRQNVMNLYLIVSDETYNAHYYIFYFIEALFAAFIGLFFFIFDFLLVTLCFSMCCQMQIICSAFESVGHKSLRDQYSPIVENIKISPKEHDLIYDELKKIIMDHQLVMKKYEDFLKLFRRVMLLHIFVSSLSVILLCFTLIMSFSNDERFKTSEIILKKLFCLIPSILFQIYMVCYLFGNIHDQKDEIIFALYSSNWTKMDMKCKKLILLTMKLNNANHKKLKFTRTKIVNLEMFFKTMGHCYTVISVLVNYISTKDEZ  >ApisOr70  MIVDRLLKDILCCYLFLVFQQYIDIITIDIRNETNHVFNIKLAKLLGLYQILDPGALKCRGRNIYHIVTSCLLLYACLISTILIISGLYYCTNIPVSIDYFWKSVTTIYVIYKTWIIIHYSNDVWNCLSITRYDLTSLTDRNRHILDRWRERLAWLTNIYVIMYCMTLVLYLVITLAFSEVKSTVKNRDGSVGYYRQNALNLYLIATDDTYNVHYYTFYFIEASFVAFITLYFLIFDVLMVTLCFGMCCQMEIICSAFKSVGHKFVTDPHSPIDDIKNQTSNEHDLIYDELKTIIMDHQVVMKKYEDFLTIFRRVMLLHIFVSSFTVILLWFTFIMSFSNDDRFKTSDVIIIRMICEIPSILFQIYMMCYLFGNINDQKDEIIFALYSSNWTEMDMKCKKLILLTMQLNNANQIKLKFTRTKIVNLEMFFKTMGHCYTVISVLVNHIQTKNEZ  >ApisOr71P  MDIRNGTNHFKIKLAKLLGLYQMLDPGAVKFRGRNIYHIVTSCLLLYACLESTILILSGLYYCTNITVIMDYFWKAVSTIYIIYKMRINIHHSNDVWYCLSITRYDLTSLTDRKKHILDRWRERLVWLTNIYVIMYFMTLVIYLVITLTFGEDSIENQTSNEHNLIYDELKTIIMDHQVVMKKYEDFSTLFRRVMLFFVSSFTVILLWFTFIMSFSNDDRFNTSDVIIIKMICEIPSILFQIYMMCYLFCNINDQKDEIIFALYSSNWTKMDMKCKKILLTMQLNNANQMKTKFTRTKIVNLEIIFKAMGHIGYTVILVLVNYIQTKNEZ  >ApisOr72  MDIRNETNHVFNIKLAKLLGLYQILDPVALKFRGRNIYHIVTSCLLLYACLISTILILSGLYYCTNIPVIMDYFWKSVSTIYTIYKMWIIIHYLNDVWNCLSITRYDLTSLTDRNRHILDRWRERLAWLTNIYATTYFTTLVIYFVITLAFSEGKLTVKNRDGSVGYYRQNIMNLYLIASDDTYNAHYYTFYIIEASFIVFMTFYFLIFDILLLTLCFGMCCQMEIICSAFKSVGHKSLCDHHSPINDIKNQTSNVHDLIYDELKTIIMDHQVVMKKYEDFLTLFRRVMLLQIFVSSFSVILLWFTFIMSFFNDDRFKTSEVMVTRMFCLIPSTLFQIYIMCYLFGNLYDQKDEIIFALYSSNWTEMDMKCKKLILLTMQLNNANQIKLKFTRTKIVNLEMFFKTMGHCYTVISVLVNYIKTKNEZ  >ApisOr73P_C  MDIQYETNNVFNIKLAKLLELYQMLDPGAVKCRGRNIYQIVTWCLLLYACLISTSLILSSLFYCTNIPVSMDYFWKTITTXIHYSNDVWNCSSITRYDFTSLTNRKRHILDRWRECLSWLTNIIYAIMYFTSIVIYFVITLAFSECKSTVKDRDGSSGYYRQNPLNLXSIATDDTYNAHYYTFYFIEASFVTFMILYFLIFNILLVTLCFGMCSQMQIICSAFESVGQKSFRGQHSPIIDATDDIKNETSNVHDSIYDELKTIIMDHQVVMK  >ApisOr74P  MDIQNETNNVFNIKLAKLLGLYQMLDPGAVKCRGRNIYQIVTSCLLLYACLISTILILSGLYYCTNIPVSMDYFWRAVTVIYIIYKTWIIIHYSNDVWNCLSITRYDLTSLTDRNRHILDRWRERLSWLTNIYAIMYFTSIVIYFVITLAFSECKSTVENRDGSGGYYRQNVMNLYLIASDDTYNAHYYMFYFIEASFIAFMTLYFLIFDILLVTLCFGMCCQMQIICSAFESVGHKSFRDPHTPIIDSTDDIKNETSNAHDLIYDELKTIIMDHQVVMKKYEDFLTLFXRVMLLHIFVSSFSVIFLWFTFIMSFSNDDRFTTSDVIVTKLFCLIPSILFQIYMMCYLFGNINDQKDEIIFALYSSNWTEMDMKCKKLILLTMQLNNANQIKLKFTRTKIVNLEMFFKTMGHCYTVISVLVNYIKTKNGZ  >ApisOr75P_C  MDVWNETNHVFNIKLAKLLGLYQMLDPGALICREQNIYHIVTSCLLLYARLISTMLILSGLYYCTGITVSMDYFWKSAMTIYVIYKTQIIIHYSNDVWNCLSITRYDFTSLTDRNRHIPDRWRERLAWLTNIYDIPYFTTIVIYFVITLEFSEGKSIVKNRDGSVXYYHEKAVNLYLIVSDETYNTNYYMFYFSGSSSIAFMXIYFLIFDILLVTLCFGMCCQMEIICSAFKSVGHKSLCDHHSPIGE  >ApisOr76C  MDIRNVKNHIFNIKLAKLTGLYQMLDPETTKCWGQNIYHVVMSCILLFMCLIPMTLMLSGLYYWTVNIPISMDFFWKSVSIFYIIYKTWVIIRNSNDIWNCLSITSYDFTSFSNRNRHILDDWRDRLARFTTIYAILYFTGTVSYFASTLALSEGKTPVKNIDGSIGHYRQNVMNFYLIASADTYNSHYYMFYFVEALFLALFATCFLIFDILLVTLCFGMCCQMEIICSAFELVGHKSLRDPHSPIIDENENTAFNEHDLIYDEEIKTIIMDHQVVMKKYEDFLRLFQPMMLLHIFISSFSVISLCFTFIMSFSDDDRFRTSDVIVKKMFCSIPPALFQIYMVCYLFGNIHDQKDSIIFALYSSNWTEMDMKCKKLILLTMKLNNANHKKLKFTRTKIVNLEMFFK  >ApisOr77  MDIQNMKNHIFNVKLARLTGLYQMLDPDTIKCRGRNIYHVVMSCVLLYMCLISMILMISGLYYWMVNVPISIDYFWKSVSTFYIIYKTWIIIRHSNDIWNCLSITRHDFTALTDQNRHILERWRKRLAWLTTIYAIMYTMSVVSYLVFTLAFNEGKTPVKNHDGSIGYYRQNVMNLYLIVSDETYNAHYYTFYFIEALFLGLIGLFYLIFDILLVTLCFGMCCQMQIICSAFESVGHKSVRDPHFPIDYTNGNTNITPNEHDLIWDELRTIIMDHQAVMENFKYNFFYFFFSININKMYKLFSCLNCIKFSTLIANSFSNDYRFKTSEVIVKKMFCSIPPILFQIFMVCYLFGNLHEQKDSIIFALYSSNWTEMDMKCKKLILLTMKLNNANQKKLKFTRTKIVNLEMFFKTMGDCYTVISVLVNYIQTKSEZ  >ApisOr78  MDIQNMKNHIFNIKLARLTGLYQMLDPDTIKCRGRNIYHVVMSCVLLYMCLISMILMISGVYYWTVNIPISIDYFWKSVSTFYTIYKTSIIIRHSNDIWNCLSITRLDFTTFSNRNRQVLDRWRERLSWLTTIYAIIYTMSVVSYLVFTLVFNEGKTPVKNHDGSIGYYRQNVMNFYLMVSDETYNAHYYKFYFIEALFAAFMGFFFFIFDFLLVTLCFSMCCQMRIVCSAFESVGHKSVRDPQSPIDEHDLIWDELRTIIMDHQAIMEKYKDFLSLFRRVMLAHIFISSLSVIALWFTFIMSFSNNDRFKTSELIVRKMFCAIPTILFQIFMVCYMFGKLHEQKDSIIFALYSSNWTEMDMKCKKLLLLTMKLNNANQKKLKFTRTRIVNMEMFFKTMGNCYTVISVLVNQIIKQNEZ  >ApisOr79F  MDIQNMNKHIFNIKLARLTGLYQMLDPNTIKCRVRNIYYVVMSCVLLYMCLISMILMFSGLYYWTVNIPISIDYFWKSVSTFYTIYKTWIIIRHSNDIWNCLSITRHDFTTFSNRNRHVLDRWRERVSWSTTIYAIIYTMSMVGYLVFTLAFDEGKTPVKNHDGSIGYYRQNVMNLYLIVSDETYNAHYYTFYIIEALFVGFLGLFFFIFDFLLVTLCFSMCCQMQIVCSAFESVGHKSVCDPHSPIDYTNGNTKITPNEHDLIWDELRTIIMDHQAVMEKYKDFLSLFRRVMLVHIFISSITVIALWFTFIMSFSNDYRFKTSEVIVIKTFCSTPPVMFQIFMVCYLFGKLHEQKDSIIFALYSSNWTEMDMKCKKLILLTMKLNNANQKKLKFTRTRIVNLEMFFKTMGNCYTVISVLVNQIIKQNEZ  >AgosOrco1  MGYKKDGLIKDLWPNIRLIQLSGLFISEYYDDYSGLAVLLRKIYSWITTIIIYSQFIFIVIFMVTKSNDSDQLAAGVVTTLFFTHSMIKFMYFSTGTKSFYRTLSCWNNTSPHPLFTESHSRFHAKSLSRMRQLLIIVSIVTIFTTISWTTITFFGESVWKVPDPETFNQTMYIPVPRLMLHSWYPWDSSHGLGYIVAFALQFYWIFITLSHSNLLELLFSSFLVHACEQLQHLKEILNPLIELSATLDSAVHNPAEIFRANSAKNQPINGVDYNGSYVNEITEYGTKGETELNRKGPNNLTSNQEVLVRSAIKYWVERHKHVVKYENLIRDCYGSALLFHMLVSTVILTILAYQATKINGVNVFAFSTIGYLMYSFAQIFMFCIHGNELIEEVTVMEAAYGCQWYDGSEEAKTFVQIVCQQCQKPLIVSGAKFFNVSLDLFASVLGAVVTYFMVLVQLK  >AgosOr2  MITSVLTMTSVLIADNKKALESFTYFVICVFLLAIITFAIRTKRFNRAMLLMVVDEFPGYERPMPDDLKRKISAIRKSYGEFTMKVMVSYLTLVLFEIPATAMVPLTAARLTDVKLGSQSTQMVVLWFPGDTTQFLIVMIVKFIITGIMCSFSFFVSQMISEFQILSAYVEHAVEIVEYDLSTGKTTDQKLLDHTTDQKLLDHVKSCVMLHHRLIDFKDQLNESYGYIILLELMFSTLYFCLSAFNMIFVGNRFVIAKGLLTLSNYLAELFIFCMYGSMVEEAHMGLLRASYSAAWYSQPVRFRRSLMMVMSRTQTPLQLTIGKVFIANLPLFLSVLKVSYSGVNALRAANAK  >AgosOr3  MKNTAVNEMKFYQILIAIAFSVNTGHPYNYIFQYIIYLGIFNYILSLIIAPLYIIWEGQTMSMIMELILYIICGILHSSHLIVIALNKQSIVATYSFIQTHFFNWSVKRGMDPNGAYKKNIKKIKYFVTIWSFLGCTIMFSPLLSTIADLGELPLDHRSHWNTHWPIFFSINNLYTYGFIYLLQTILAVFLITATGAMKCGFIVLLSELNYQFEHLLHGLEHAFNHRMEQKFKIIFFDCVRHHQYVDNFKSYFKWITLINIFMLQGIISTSLYCIIKIDAPIGYKMKQCGIIITHIAEFLFHCYLGEIISRMHNHLEEKVYNMTWYDMPNPHKKFLMIMLQRTQKDLVPNAAIFSSHSLSRSLMTKFVKQIYTLLNVLLKT  >AgosOr4  MTLIENKNYHRKFYTILMTVAFFLNTSQYDCIPKFLMHFYIFDWMMFVTLAAGYIFFYEKPGMSLGMELIQYMIVGILYTLIFLVFILKNEAIMSNYNFIQTKFIHWSNKKSLHPNAVYKKNIETVKSLAIPLATLSLSIAFGPLVSTINDIGKLPLDNRAHFVLFWPKIVDTNKISMYGIIYAVQVLFTVILYISSLSFNLGFMVFLNELTNQFEILLDGINDAFKYKRDKQFQPLFIDCIRHHQIIIKFLDDLKSYFKWVILIEIIVVQVVLAILIYNLTKVNASIGYKVKVAGSLLFNLLPICFHCHVGEVILSLHTRLSNHIYNMVWYDMPNKNKQLLVIMFQRTQRDLTLSSALFSNERASRSLISKVIKQVYTILNVLLKT  >AgosOr5  MPRIDAINVFLQMTGCTDSKRMLYLTYFEFLITLYYFIASYVSIIYYEQSVSIQLFTLLCMLIESYILLNITFRIYHKNQFREMDQYSKQLGIPDDYQSKINIITMYHLIASNMFVIFPVYFNTMKIVFTKPILQSMSFNAIYFGLTTTLVIQAIRGYINQTIVSICIASGIAAIINITIYTFYGSVLLDLHDEILRVLFDNSFFYVNKSFKRSILIMMLSYTIIKMILSSEAIKPHKMS  >AgosOr6  MDVREENKHVFNIWLAKRVGLYQMFDPGTARYRGKNVYHIALTFIVLYLGVIATMMNVSGVYYWKDNMPISIDYFWKAETWLFVFFKMWIVVYRSTDIWDCLSITRYGFTSFGYRNTRTLDRWRERSVRFTTAVTVIYLTSLVFYIAGSLAFREDVILVKNHDGSVGYYHQNVMNFYFVVSDSTYNAHYNTFFFVEAATAVLLTMLFLIFDILLITMCFATCCQMQLVGCEFESFGHDKPLGDDPRRSPIGEHEFSDYTDERKNVFKERVSMYYDELKTIVLDHQAKYENLLSLFELAMLLQIFVSSITLIILWFIFIMSFSNDDRFIVSDIIVKKMIFLIPSLSYQIYMECYLFGLLHNQKDSVIFALYSSNWTEMCMRCKKLILLTMEMNNANHIKLKFTRTKIVNLEMFFKTMSDCYTITSVLINHIKTKNK  >AgosOr7  MDGFNEQNILIDFNLFKELQFYQIFYSSGIKIFGLNIHQLFYISYALVALCIESYGISTLFSTNCKFLSYIDYFIIFYVVNQMYLSFWKLFKCLNDRNRLLDLFKIAQLNFLTSEECTKYSKVLYKHRDKNSKFVNYFLIFSFVVILQWFMFPIVINQILYFENSNVRAQNIINLCFGVATLTYNKFVLIFYLLEITVTSLTVYILIMMDTLIIFLCSAIIYHQEVLIYAFKNIGYEDNPKISKIIFFYSKYKKLIRINVYFFRKIKLFYSIMKSTILLTVGIDSFYLIFFTYLFILICLTPGSDTLLPLIKISMSVVYITGRLFIYCYLFDSIYIQRESVNFNIYSCNWTKMNLKFKKLLLLTMQMNDANRMGIKASPKKIINLQLFAGIMSMSFNMVPVLLKITNSENNKP  >AgosOr8  MVFIFPLVINEIIHFENSNVRAENIVNFCFGVSTSTFNEYYLIFYLLEITVTSLTVYILIMMDILIIFLCSAIIYHQEVLIYAFKNIGYEDNLTISKTMKIKLFYSIMKSTILLTVGIDSFYLIFFTYLFILVFLTLIKIGSTVLYITARLFIYCYLFDSINKKRELVNYSIYCCNWTKMDLKFKKLLLLTLQMNDANRMGIKASPKKIINLQLFAGVIIKFX  >AgosOr9  MSIAVMVHYMAEMFFKKAICSDDDLHRREAMRMVFFTYGELAITLFFAVSTYLSIVHSTEDLSVHLYGVLCLIIQLLVFAFLSFRSYHRSHFRDMYQRSRGMEISENSNRKIAAVIKHHLIMPNVVHIGDPFTFPFMDVLPIETTSVAVYVCKYVVYALPVYLTQIEVCFLNVTYIVIFRLLEKQVEEAMVNKDEHKLKIAIKHHQELLKFFKEMKTVYEKPIFLIIVSCGLYIGLTHDRILNALFQHQLLYSQNKSFKQLILIMMTRATIPLEFKAGSIFTVNMNLLVRILKFAYTVFNVLITSINHQLIKTAV  >AgosOr10  MEHIVDMFLKKTGCNDDRSYDTMCQVFFIYFELAITLFFAVSSCLSIVNSTEGLSVRLYGLLCFLIESHIFFFIAVRLYYLPQFRDMYQRTLKMGIPENFRQRIAMVIKHHFIISNVFVSIFMLYTISMDWVQMGDPFTFPFIDVLPIKTTNLTIYVCKYILYTLPVYIAHFETCFLNVTFMYSTGVMKRYFQILDGQVEEAMVNKDEQQLKIAIKHHQEVLRFFNDMKTAYEKPILMTIEFCGLYVGLTSYFSILVIQGYIHQIILGLCIVSSIASLITIIIYCIYASNMYDLHDGILSALFEHRSVYSRDNSLKRLISIMMTRATIPLEFKAASIFTIHLNLLIKILKCVYTVFNVLLTSISRKLKETAV  >AgosOr11  MFIYATFFVIIKISFLIIKSDILWNFISFTSINFLSYSGHQKYFLMNARIISLIISNVFAILWVAFIALWIFSPIVINDSYLNIKSKNSTYMQYRYNTLNLLFPVSTQFYNDNFTIFYLFETIILIIYGYSMIVFDCLIISFCLTIAFQLRTIASSYSTLGYNHTNNQIKSFINNIIHIVNTINSNNGISLTSTESIKLLSAEIVNTGHLFSACYLFSLIDIYNDTINFALYNCNWTEMNINFKKLLLFTMQMNNANNFKLNISTNIIVNLKLFTNVIHFTYKIISILKSVVN  >AgosOr12  MDSKALKILRLTGLYQILDPNTAKIDDCNIYHIVIVFFASFTLVVSMLFPMGFIYLRNDIIALMFYVGCISNFMLCCFKVLNILYYSKDIWKFIDVTKYYLTLYKHYNTNVLKNWQTRSTRIMYLYIIIMFIGFCFWFFSPHILNESTVTIRNIDGSYGKYRLNIFNLYLIVSDETYNMYFYVFYVIEIIMQICFLYFTIVCDIIMILISFEIISRMEIICIACGSLNYNIICSKEGSNPIKSEKKFDVIYDDLKIIITDHKTVIRKLKEYYTIFRPVTIIQIFITSSSHIIIWFVVAMNFGEGDKGNSIITIKLFAVHPLLSFQLYMMCYLFGSMNEKKDSIIFALYSSNWTEMDIKCKKMILLAMEMNNANHLKMKFTNTKIVNKEMFTQVNITSHIYKVNYKMNVFYYL  >AgosOr13  MDSKHEKQYIFNMKLAKIMGLYQILTPDSTSIFGYNIYHIVIVFFGSFMFIISMLFPIGLLYLRNDMIALMYYMGCISNFILSSYKMGNILYYSKDIWKCIDVTNFYFISYKHYDRNLFKNWQTRSIRITYIYIVIALFAFFCWIFSPCVMNKSIITIRNIDGSYSRYRMNIFNIYLIASHETYNTNFYIFYIIEIIVSVCYVYFTIIFDVLMLLICFAISYQLETISNTIKSLGYNLSIQDNIGTKCVIFTGTSNSIQLKEKCDIIYNDLITIITDHQHVIKKLNDFYNMFRLVTLTQIFIASSSHVFIWSIGAMSIDEGDNADSILSFKLFIVLPLINFQLFMTCSLFGTINEK  >AgosOr14  MVYASSQMYQSYWKIFKCLKDRNRLLDLFKLAQLDFLKSEECAKYSKVLYKHHDKNLKFSNYFLIISFVVIIQWFVFPLVINEILNFENSNVRAQNIINFCFGVSTQTYNKYIPIFYLLETTGAALTVYFLTMIDTLIISVCSAIIYQQNVLIYAFKNIGYENKSTINYYKTFISILQDQLRLNLKIKLFYSIMKSTMLLNIVIDSFFSMIFTYLFILV  >AgosOr15  MTPDNTLKYIINLKLMKLTGLYQLLNPDNPKSFGCNIFKLGGTLAVVYLILVIIMCNLSIYYSLNDFTEVVKYIMLIIAALFASTKMCFVILYSNELWKFISFTSIDYLSYKGHKKYMHNKARKLSKSISNIFTLAWIAVISVWILSPIIIKDNFMNVKSKDDTYNQYRYNMLNLIFPVSAQFYNNNFKVFYFFESIALIVYGYSMMVFDCLVISMCITITYHKPLDERSNDLNNLILIIQDHQKCIKNLNKGLSLTSPESIKLILSALTNIIHLFSTCYIFSIINTHKDSINFALYDCNWTNKNIMFKKLLLLSMKINNSEKLKLKASSQIIVNLQLFTNVIHTTYKIISVLVNQYR  >AgosOr16  MDSFNEQNILINFNLFKQLQFYIFYSDGTKIFGWNIHQLYIVFGLVGLCIQCIGLSTSFFNNCKNISKIDYFLMVYASSQMYQSYWKIFKCLKDRNRLLDLFKLAQLDFLKSEECAKYSKVLYKHHDKNLKFSNYFLIISFVVIIQWFVFPLVINEILNFENSNVRAQNIINFCFGVSTQTYNKYIPIFYLLETTGAALTVYFLTMIDTLIISVCSAIIYQQNVLIYAFKNIGYKIKLFYSIMKSTMLLNIVIDSFFSMIFTYLFILVC  >AgosOr17  MATASPSEEESTIVDNRLFKAICLHQILNPTHGSNRYIRSQEPFSKHQSAIMATASPSEEESTIVDNRLFKAICLHQILNPTHGSNRYYRIAILACIWMSIVVQITQLVGLYYAVNDLQRFAFTTTVVVNSFLSLAKAYVLMANVDRLRDGLEAARYEFTSCGSRDQRTVRRARAALSTLVRTFTVFSYVTCFFWMLNPLSAIGEFLPLTNADGTVSHYRVTIYNYWLPVSATVYNTTTVWALTYAIEMTVCFFNVNTWLLFDSYVLTMCFTFKAHFRTLSASYATIGHLDTFRSLTPHASGFLNWKKGLNKTQNKFKFKHVLINIL  >AgosOr19  MGVLVVNGLMCLLKGYMVVANADRMCSTLDATRYAFTGCGGRDPSELRRCRATLSTILRTFVALSFGTLVVWVIIPGKYELFFRIVRPVVLLQIANGSYSIITLIFLTSIAYLNGDSIVSPAIFKLVCALISLTIELYIYCYGFNHIEDGRSTVNFGLYSSNWTDKDLQFKKTLLLAMTINSAHKLKMKVSPNSIVNLEMFTRVMNMSYTIVSTLLS  >AgosOr20  MAHSSSTTVVDVSLFKTIGLHQLLCPVNRGGYSVRSRRALMAHSSSTTVVDVSLFKTIGLHQLLCPVNRGGYSVRSRRALMTALGLSFALHAFQVPWLYCALNDLQRFAYMAAVIIYGMMCAFKGYVLVTNSDRLWWVLDAAGYGYTGCGGRDPSALRRCRVTLSALLRSFVALSYATLIVWIVLPFFVDEYTPITNLDGTVTRYRTTIHNMQFPVPLSVYNSRPVWTLIYVTEVSVCIVNVFIWSIFDCYLVTMCFVLNAQFRTMSTGYVTLGRRRVKPLPQDTPVKGVRIKFNDVKSNHYDDLIGHIEDNRKLIKAFDVFFDVVRPVVLVQIGNGSYSVISLIFLTSLMYLMGVPVLSAPFLKFICGVISLTLELFIFCYGFNHIETAKSNINFGLYSSNWTEMDLKFKKTLLLAMKMNSSHKRVMKVSPKSSVGLEMFARVMNMSYSIVSVLLNSRS  >AgosOr21  MYQLLRPVECGLDVGRCRSAALAVVFMTLGLQSMQVARLYLARHDFQMFANMGVLVVNGLMCLLKGYMVVANADRMCSTLDATRYAFTGCGGRDPSVLRRCRATLSTILRTFVALSFGVWAVVYVVESTILTVNVFCWTSFDCMDQWYTKHKLRLFRTMSTGYETLGRSRSGDVKLSARQQFDTVAAGAAISKTSITDDNLDDLKSHIIDNKNIIEQYDAFFDVVRPMVLIQIADGSYSIITLIFLTSLVYLKGYSIVSAPILKFVCGLASLTIELYIYCYGFNHIEDGKSTVNFGLYSSNWTEMGF  >AgosOr22  MYQLLYPAECGLNDGGHGYRTAVLAAMGLVLGLQSMQVCRLYLARHDIQMFANMGMLVVYGFMCLLKGHTTATNASRICVTLDAARYAFTGCGGRDPSVMRRCRATLSTILRTFVYDDMPTVWAVVYVVESIIFTVNVFLWTSFDCYLVTMCFVFDMCFVFDALFRTMSAGYEKLG  >AgosOr23  MNPNDENYIINLKLMKITGFYQLINPHTSKYLGFNVYKVGAGLEVMFGIISMLLLFLSSYYYLDNTNELMSHFMLIVAIFFSTFKISWVSKKSEMIWNNLDMTSINFLSYTGHKQEILQTARAKSISTTIIFVILWSSVTVAWSISPFFIKDVYLNVKFNDEIRRFRYNSLNYVYPITEESYNENFLYFYVVEMLQVIFWGHGTVAYDTFVISICISIAFQLKTIAVSYTSLNDIKGDIKNLKHNDLEAILNLKLVIQDQQKMFKKIKEIYKIFQPVTFVQLAAQSMLIILQAYMIFIFLLLSVPIIKLIVTVAPNIIHLFITCYLYSNINDQVLNLIIRHIIIIMTIIYPQNILIILSIILFQKDSMNFALYSSDWTAMSIKYKNMLLFAMRMNDAEKLKLKISLRKIVNLEMFASVKPKIKFYEKNNN  >AgosOr24  MELQNEHSITNLQFMKITGSYQLLMPSHGLTFFNINIYKIAFIVQILFLTIAAIMGVFSIYSCRNNVNQIIHYIIVIFAIYFAIYKYYFIIKNSKIIWDCMHMMSTNFLSYNDHTKEIFKIARTRSFNVDIDFPYLSAILIHSSYLKIKHEHGIYKYRSNALGLVFPVTDTFYNKYFIVFYTTESIFLLFWGQMMWVFDILMISICISIEYQLKTIADSYSLLGLKDKHLTRELSNSKYIRFFVFIQLAAESFQIILHACMILKLYFDGSMSPTIFLKLLFPEITYLCHLFLTCYLFSIVNEQESMNFALYSSNWTDMNIKFKKLLLFTMRVNNAENLKMKISINRIVNMEMFADVMHITYSIVSVMMKSYSK  >AgosOr25  MATTTKMVYKNEDNLMINTRLMKITGLYQLLDSRTSKIFGQNMLKCMSLFQLSIMFITVVIFLANTYYFSDDINAVMQYSILFVCDVLSILKLYVTIVKSDTIWNCIQMTSIDDLSYKYHNRSILRNGQLKSKSYSILIMFMWMNLIILWALAPLFVTNYFLEVEVKKKIYRYRFNIMNFVFPATDQFYNDNFVIYYCIEFACLIIWCHCTMNFDVLLLSTNITFKYQLKTIANSFSAFNITHYIKNNFTKNIKHYKESELIFDFKSIIYDQQRVIENMRNIYRIFQPVVLTQLAFESIIIILLSCIIMMNYFNGISLLSAMNLRLFAAVLTFTFHIYVICYLFDNVNQDRKSVV  >AgosOr26  MNNAENLRLQVTRKRIVNFKMFTDVRTIFLFFFILVPTTDIIFGHNVFKCLSVVQMSSLILLTVIFLSNIYYFLDDINAVMHYSTLLTSDMISIFKLYVTIVNSDTIWNCIQMTSIDDLNSDTIWNCIQMTSIDDLSYKYHDRRILINGQSKSKSYSILIMFMWMNLIISWSLAPLFVTNFFLELEVENEIYRYRLSIMNFVFPVTDQFYNDNYVIYYCIEFTGLILWCHCTMNFDILLLSMNITLKYQLKTIANSFIIYLYNYVLYAFNNLTKNVEHHKKSELLMFDFKSIIYDQQRVIENMRNIYRIFQPVVLTQLASESIIIILLSCILMMWHFFFFIQNYFNGISLISVMNLRLFLAVLTFTFHIYVICYLFDDVNQQKDSINFALYSHDWTQSNAQHKLLLLHAMRMNNAENLRVHVTQNRIVNFKMFTGVRIIFFFYFNAYLQICLKRYNFIFYI  >AgosOr27  MQYRYNTLNLLFPVSTQFYNDNFTIFYLFETIILIIYGYSMIVFDCLIISFCLTIAFQLRTIASSYSTLGYNHTNNQIKKNQLINEKPMDLSNLIVIIQDHQKLTKKIHDIFDEMRPIILFQLLSESILMSLIPLVLFLNSNNGISLTSTESIKLLSAEIVNTGHLFSACYLFSLIDIYNDTINFALYNCNWTEMNINFKKLLLFTMQMNNANNFKLNISTNIIVNLKLFTNVIHFTYKIISILKSVVN  >AgosOr28  MDNFNGKNILINFKLCKQFQFYQMICSSRMKIFGWNIHQLVLYKQRDKNSKFVNYFLIFSFVIILKWFIFPIVINQILYFENSNVRAQNIINLCFGFVLIFYLLEITVTSLTVYILIMMDTLIIFLCSAIIYHQEVLIYAFKNIGTVLILIKIGSKVIYLTARLFIYCYMFDSINIKRELVNYSIYFCIWTKMDFKFKKLLFLTMQMNDANRMIMSMSFNMVLVLLKITNSENHKSQLT  >AgosOr29  MATRTKMATKNEDNLMINTRLMKLTGLYHLLDSRSSKSFGHNVLKYLSLVEMSILFTMMVIFTANLYYFSDDINAVMQYSMLFACGVISIHKLYVTLVKSDTLWNGIQMTSIEDLSYKYHDRSILSKGQLKSKTYSILIMFSWINLIISWSLAPLFVTNYFFETRVENKIYRYRFNIMNFAYPATDQFYNDNYMIYYYTEFVFLMLWCHCTMNFDILFLSMNITFKYQESELMFDFKSMIYDQQRVIENMRNIYRIFQPVVLTQLASDGTFFFIQNYFNGISLISAMNLRLFAAVLTVTFHIYIICYIFDDVNQQVSYIKDSINFALYSSDWTQSNPQHKHLLLHAMRMNNAENLRLQVTRKRIVNFKMFTDVHSINFALYSHDWTQSNAQHKLLLLHAMRMNNAENLRVHVTQNRIVNFKMFTGIMRKAYSILSVLGKMCAKKT  >AgosOr30  MVTTTKMTTKNEDNLMINTGLMKITGLYHVLDSRSLKIFGHNVFKCLSVVQMSNLILLTIIFPANIYYFSNDINVVMQYLMLLTSDMISILKLYVTIVNSDTIWNCIQMTSIDNLSYKYHDRRMLRDGQLKSKSYSILIMFMWMNLMLSWGLAPLFVTNYFLETQVENKIYRYRFNILSFVFPVTDQFYNDNFMIYYYIEFVYLILWCHCTMNFDILLLSMNITFKYQLKTIANSFSTFNITHYVKNNLTKNVKHRKESELMSDFKSMIYDQQRIIENMRNIYRIFQPVSTYSTSFRVYNNNSSVLYHNDELF  >AgosOr31  MIDILLLSIGWAITVQYEVLAVAFKNLGHDVNFQKGETTLFKKIYKKVKLYFSIVKPIVLMHVAISSGLFIMLSNSFIMIILSKESFTILIVNLFKIGVGIFYICLQLFLYCHLFDNINLKREFVNLGVYSCNWTKMNLIFKKLLLLTMQMNNANQITMKATTNKIVNLQLFSNVNIKFFVLNKSYNIISVMVKAISKX  >AgosOr32  MATFNEHTAAIGLKVLKQFGFYQMFESNTKKIFGWNVYQFSYIILLMINQCLIVFGNSGFLFELDDTINNINLLLIIFSNSFNYLTVYKVIILILNKSKIQQVLDVTDLKFLKSKQCRDNKVKLYFSMMKPIVLMHVSINAGLIMMLSTSFCMVLLSTESFTQAFVNLFKIGIGIVYLSLQLFLYCHLFDNIHLNIQSVNLGIYSCNWTNMDLKFKKLLLLTMQMNKANEIMMKASMKKIINLQLFASVLTTSYNIVSVMVKTIGK  >AgosOr33  MSSFEVSNVAINIKLYKLLRFYHLFDPNNIFGYHFYRFTGIFITVFIQLFVLFGLLGCFMEMEDTINDIEQFIFIFVNLSNFLSVMKLCVFTYKAKNTWDLFDVTCIHFLKSEKCCKYRNEILEKVRNKSIKLTNFIFYRKLKLYYSIIWYVILTYVVLSSCSIITLTYSFIMVITCISSTKSLPVLSIIKIIAPFSIVSFQIFLHCYFFGLINFKKASVSYGMYSCNWTSMDLKFKKLLLLSMQMNDADKLMIKATPTRIINLELFVKVDNNLLLSEAIYK  >AgosOr34  MDGFNEQNILINFKLFKQLQFYQIFNSYGMKIIGWNIYHLFYIIYALVGQFIGCYGMFTSFSINCKYLSDTDYFIFVYTAIQMYLSIWKIFKCLKDSKKFLDLFKIAQLNFLTSEECTEYSKVLYKQRDKNLKFARYFLIFSFTVSIQWFIFPLVINQIINFENSDVRAQNIINFCFGVSTSTFNEYYLIFYLIEIIVASLTMYILIIIDALIISLYSAIIYQQDVLIYAFKNIGYEDKPTICKIIFLVYIFKMNVYFFRKIKLFYSLMKSTILLSVAIDSLYLIFLMYFFILVCFLFKLIQL  >AgosOr35  MKRSRFYHIFNPNGSKIFNYNAYRLLLIILLVLVNGIVVYSSLGFFVKMEDTLSYIDSSVIIFVMINIFLCNWRFSVFLYNAKIIYDVFNVSRFDFFKSKHCCKNINVLCGHRDRTIKITNYFFVFSSTVMSQWVLFPLVLIAFTAPEDENIRQQNIMNLRYPVSTHTYNQYYYLFYLMEVIVAIFTMYSMIIPDILLMSWCWAIIAQQEILIQTFKYFGHEDSSQTVHYEDFKSILVDQIQLNLKIKSFYSVVRPVVLTYVAIISTCFIIVTYVLIVVCLSKESNSVLNIIKLGSSALYMCLDLFLYCYLFDSMNIKLESVNTSIYSCNWTKMDVKFKKLLFLTMQMNNANNLMIKASPKKIVNQQLFANIISMSYNIVSVMLKTTS  >AgosOr36  MDSFNGQNILINFKLFKQLQFYQIFHSSGLKIFGWSIHQLFYIVFGLVGLCIQCYGLSTSFFNNCKNISEIDSFLIVFASTYIYLSLWKLFKCLKDRKIFLDLFKIAQLDFLTTKECTKYSKLLYKQREKNLKFANNFLIFSFVICLQWFIFPLVINEIINFENLNIRAQNIMNLCFGVSTQTYNKYYFYILLTRNNTNIDISFHYNIYGHINNFNLFSYNLSTRFTNLCVQKYWI  >AgosOr37  MKWLQDHEVAINLALFKRYQFYHIFNPNSSKILNYDSYKFTNVLFIVVVTSYNIFSAMCFFTDTVDTIDSIDLLLMIFIYSIIIISLLKICVLLFNADQIWDLFNLTRFDFLSSRRCRKNVGILYKYRDRSITITNLYQNYSTIVFIIWMIVPLVLNTFAMTEGPNQRYHNIFNMQYPVSASIYNHYYYIFYLMEIAMGIFVLNYSMIIDNFLISLCWAIIAQYEVITTAFENIGNDCKLENLQKEKKNNSFEAYEDLKSIIMDQKKIYTLICSSAESFSIFNILKISTAFFVFVIQLYLYCYLFDVLNDKKESVNLGIYSCDWTRMDLKLKKILLIAMKFNNANQLKIKATPNKIVNLQLFSSVMTTTFNIVMVMLKTINEKN  >AgosOr38  MEDVFNIINQIQLMFCILIYYSSLLKIITFLYKANNIWDLLLVSKINFLTSTQCKTHIDILHKYRNKSIKITNMISTIGVVTTLEWIMYPLLLQLLQKEDANKSNQRFENIFNFRFPVTINYYNNNYAIFYFMESFITMYMLYIYVAVDVFFISACYIMIAHYEMIKRAYENINIELISENNNKNKNYCNDCIDDLVSIMMDQQKHFAKLKLFYSTYKFIILSTVIINSGSIIILTYASVVIFMSSETISILSVIKLISAFGYVFIVLFFLCYLIDRINNKMESVHFGMYSCNWTAMNLRSKKMLLLSMQLNNANKLMIKITPKKIINLQFYNSVILKFIYIYFFII  >AgosOr39  MNSENIFNGRPVALNLSTYKQLGYYQLLDPKGPYLYGCHFYRTILKIFLLVIEDSDANNGKSNSFELIIILTNCTLSSLKIYTLISNTKIIWDLFDLTCIDFLKCSRHSELITENFVTRCKKSTEITKWIARSFLIGLILWVMGPFIANEEYTAPNASHRYQNIINIKFPVTVKTYNNYYLVFYLMEVAVGFCIVYGSILVDAFLMSFCWIISAQYQSVTKAFATFGHKNELSSPEEIYKDFKSIIIDHQNVYLKMKSFYAVVRPITLIHVFAYSCSLIMYAYVIVTIFHSKESFIIAEIMKIVMTVSNVTIEVFIFCYLFELIDNKKEDVNFGLYSCNWTGMDIKFKRLLLMSMKMNNANRLKLKATPDVTINRPFFANV  >AgosOr40  MVGDSIGMITMPFLIVMFYTQDKSIFNSNVIAFSWTLLVVGIQLYMYCSLLQNVNERRENINFGLYSCDWTRLDIEIKKLILLAMRMNNSNNLKINVTFTKFIDLPMFASIIRSSYSVTSVLINSNIHKINK  >AgosOr41  MTFDKSILESNEVSINLKLFKFIRFFHLFDPNIKKICNLNVYHLAWYIINCVIGCIVIYGLLGYFTEKEDILNIVNQIQLMFCGLLYYSSLLKIIIFLYKANSIWDLLRVSHMNFLTSSQCKTHIGILHTHRNQSIKITNFISGFGIVIALEWIMFPLLLQLLQKEDVNKLNQRFENIFNFPFPVTINYYNNNYVIFYIMESFIAMYMLYIYVVVDVFFISVCYVMIAHYEMIKRAYENVNTELVSENNNKNKNYCNDCIYDLVSIMKDQQKHFALLKLFYSTYKFIILSTVTINSGSIIILTYASVVIFTSSESIPILSVMKLISAFGYMFVVLFFLCYLMERINNKIESVHLGMYSCNWTAMNLRSKKMLLISMQLNNANKLMIKITPKKIINLQLYNSVIITCYNVLSAMLNTRSE  >AgosOr42  MYFLVSNETYNEWHLIFHLLEWMFGLCFVMFMVLFDTFMVSMCIAITYQMRCVAAAYRRLGHNRRTTMKVYFDGAIESDNTNNECLKDLKIVIKHHQEVLGKMNDFYKIVRPVILPQLIIASFTIIFVSFIITRNYFNGMLLTSTQSLKMCSFPIFFFQIYYTCLVFENLNNQKTAMNFALYSSNWTQMEIQFKKLLLLAMQMHDANKLNIKLTAELIINLELFTKVMNLCYSIFSVLVNSQLKITDKLX  >AgosOr43  MDSKHEKQYIFNMKLAKIMGLYQILTPDSTSIFGYNIYHIVIVFFGSFMFIISMLFPIGLLYLRNDMIALMYYMGCISNFILSSYKMGNILYYSKDIWKCIDVTNFYFISYKHYDRNLFKNWQTRSIRITYIYIVIALFAFFCWIFSPCVMNKSIITIRNIDGSYSRYRMNIFNIYLIASHETYNTNFYIFYIIEIIVSVCYVYFTIIFDVLMLLICFAISYQLETISNTIKSLGYNLSIQDNIGTKCVIFTGTSNSIQLKEKCDIIYNDLITIITDHQHLNDFYNMFRLVTLTQIFIASSSHSIDEGDNADSILSFKLFIVLPLINFQLFMTCSLFGTINEKKDSIIFALYSSNWTDMDLKSKTIILFGLTMNNANQLKMKFTNTKIVNLEMFSHTMRFCYSIFSMLVNYNNKKKNN  >AgosOr44  MMLLNGLYYSKNNITESILYTGFVINMFYASYKMYIVLTRSKDIWDCLSITQYDFTLYDHRDRRIILDLWRNRSIWLTSTFMIFSFVMVTFYVTCPLAFNNTFIVMKNRDGSTSTYRMNVLNLYLFIPEEAYNNTYFNVFYIIEASGTYVLVLFVIIFDTIVMTLCLALSCQLHMNFAAFESVGHTSVVILKTNYFCLDNNDNIDNKIKLPNEYINGIAMYNNLKTIITDHQNVLKKYDEFLSIFKPIMLLEIFVLSYAIIVLWIIFLTSFIVGEFTESMGVTSMQTGFAIPFCVIQLFMSCFVFDILHNKKDSMTFSLYSCNWTELFDMKCKKLVFLTMGMNDAHHQKLQYTRTRIINLEMFYQVCFTLSSX  >AgosOr45  MKIQDNDKQIFNLALAKFIGFYQVVDTEKVTFLGRHNVRYKIFVFLIVYECLIAAILLLNCLYYSENNPTEFIRYTGFVVNMFYASYKMYIVLKRTKDIWDCLSITQFDFTSYDHRDRRIILDLWRNRSIWFTNTFVVFSLIMLIFYTACPLAFNDTFIVMKSRDGLSSNYRLNVVNLYLFVPEEVYNAYFNIFHIIETFGICIFVLFIVVFDTIVMTSCLALSCQLHMNSAAFESVGHTSVVDSPNNNIDNKMKLPYENINGIAMYNNLKTIIIDHQNVLKKYDEFLSIFKPIMLLEIFVLSYSIIILWLIFLTNFIVGEFTDSKGVASMQTCFAIPFCIMQMFMSCYVFDILHNKHNNNMTIDYRLGSNK  >RpadOrco1  MGYKKDGLIKDLWPNIRLIQMSGLFISEYYDDYSGLAVLFRKIYSWITTIIIYSQFIFIVIFMVTKSNDSDQLAAGVVTTLFFTHSMIKFMYFSTGTKSFYRTLSCWNNTSPHLLFTESHSRFHAKSLSRMRQLLIIVSIVTIFTTISWTTITFFGESVWKVPNPETFNQTMYIPVPRLMLHSWYPWDASRGLGYIVAFVLQFYWIFITLSHSNLLELLFSSFLVHACEQLQHLKEILNPLIELSATLDSAVHNPAEIFRANSAKNQPINGVDYNGSYVNEITEYGTKGDTEPNRKGPNNLTSNQEVLVRSAIKYWVERHKHVVKYVSLITECYGSALLFHMLVSTVILTILAYQATKINGVNVFAFSTIGYLMYSFAQIFMFCIHGNELIEESSSVMEAAYGCQWYDGSEEAKTFVQIVCQQCQKPLIVSGAKFFNVSLDLFASVLGAVVTYFMVLVQLK  >RpadOr3  VMTCLCASSVLMANGFSAFHIKMMSLLIIVVSQFFFYCLIGEQFSTMNKQLGDCVYFKLVKCKDPKLARVGLLMILRTQKPLQLTTMGITKYTASLFTFTVTMRSAYAGFNVLYNS  >RpadOr5  MPRIDAVNVFLRMTGCTDSKRMLYLTYFEFFITFYYFITAYVSIIYHEQSVSIKLFTLLCMLIECVILLNITFRIYHQNQFREMDQYSKRSGIPDDYQSKINIITMYHLIASNMFVIFPATYAILSDSVRVGDPFTFPFLDVLPIKTD  >RpadOr16  MYLMGIPVLSAPFLKFICGVISLTIELFIFCYGFNHIETAKSVINFGLYSSNWTEMDLKFKKSLLLAMKMNSSHKRMMKISPNSAVGLEMFARVMNMSYSIVSVLLNSRS  >RpadOr17  MAVFNDHNAAIYLKTLKQYGFYQIFFPNGENIFGWNMKNIYRLSFIALTVIIQCLIGFGNCGFLIDLEDTINKTDLFIIIFSNIFNYHILCKVIILMFYRNKILDLLDVTSLNFLKSEQCLYNIKILYKHRNRVIQRTKLYYKFCMSVIITWVLYPIVINLFVANKNDNRRLENIINRRYPMDVNTYNQYYVLFYIIEMIIAIKSLYLVLMIDILLLSIGWAIIVQYEVLAVTFKNIGHIVNFRKDHDYNNVVDEDNYKKFKSIIFDQQQLDLKVKLYFSIMNPIILMHVAINSGLIIMLSNAFIMVIFSTESFSITIVNLFKIGIGIFYLGLMLFLYCYLFDNMNVKRESVNIGIYSCNWTKMNLIFKKLLLLTMQVNDANRITMKVSEKKIVNLQLFANVLTMSYNIISVMVKTIGK  >RpadOr18  MKNMYQILTPVVFIQLAAESIQIILHACMILKLYFDGSMAPTIFLKLLFPEITYLFHLYLTCYLFSIVNYQKESMNFALYSSNWTDMNIKFKKLLLFTMRVNNAENLKMKISINRIVNMEMFADVMHITYSIVS  >RpadOr31  MNMTFDISILEPKEVCINLKLLKLFHFFHLFDPKIKTICNFNVYHIALYIINCVIGCIIMFGMVGYVTEKENAFNIANDIQIMFVCILYYRCLIIIFTLIYNANNILILLNVIKMHFLTSTQCQKHIGILQKYRKKSIKITNYISFLTIISSVLWFLYPLVLMLFQKEDAYKSNQRFENIFNFRFPVTNIYYNNNFVIFYIIEISIGLPLAYMHLVSEVFFISLCCVMIAQYEMIQIAYKNVNSELNYENSNKHKNDVNINDCYDDLLSIMKDQQKLFEKLKLFHSTYKLVILSNVVVYSWSIIILIYSSTLILFTSSESIPMLTIIKLLTTFGFVFTLLLFLCYLLERINNIKESVHFGMYSCNWTSMNLRS  >RpadOr43  NISNGNSMQLEKKCDIIYNDLITILADHQNVIKKLKDFYNMFRLVTLTQIFIASSSHVFIWSIAAMSTDGGDNADSILSFKLFIVLPLINFQLFMTCSLFGTINEKKDS |
| **GRs** |
| >ApisGr1  MFNDDNNNTGPVYLKHRNFAESAYRQQQPQPISAVQFADSAFNSKANKGNNIKLEIQDDLGMPVNYVRIKALEDDIRMNIISDKNSLHRAITPVLILAQMCALLPVQGIRGKNTSYLVFNWYSWPVIYVFIVIAASLLILSFSLIKIYMTGLTYYSTGEIMFFGSSLVIYILFIHLAREWPKVMEKWELMEREMRQFGYPSKTAFKFKILTSIIMVLAIIEHSASVLTGIMKAIPCSTGGLDIFRAYFSMSFRQVFALIDYSLVIAIPLGFLNLMLACAWNYMDLFIIILACALSDKFKQLNQKLASIKGKVLPSTYWRKSRETYNLLASLTQDFDEFLSPVILLSFANNLYFICLQLLNSLKPMHNVWEAIYFVFSFTYLVGRTCAVSLYAASINDQSKKPKAILFSVPTESYGVEVARFLTQVTSDELALTGCNFFSVTRTLMLTVAGTIVTYEIVLIQFNSVNSDGNGQNHTIVYCP  >ApisGr2  MPNENTFQPVYLKERNFSEAGYDRQSKQSSLTDSAVRSNYNALSLKENKGSIALEIQDDLGMPVNYVRIKVLEDSVRKNIISNKNSLHRAITPLLILMQMSGLLPVQGIRGQNTSYLIFNWISWIMAYDIIVVGSSIVMVSFIICNMYINGLSYEGIGDLVFYGGTLINYMVFIHLAREWPKVMEKWELMEREMKQYGYPPNMAFKIKMLTCIIVLFSIIEHLASILTGVLKAIHCSKDGLDIFRLYFMTWLKSVFTIIDYSFVVAIILKFFDCLYSFAWNFMDLFIIILACVLTEKFKQFNQKFDSVRGKVLPSMYWRKSRETYNILASLTEDLDEFLSPVILLSFGHNLYFICLQLLNSLKPMHSSWEVLCFVILFTYLVGRTCVVSLYVASINDQSKKPKAVLFSVPAECYGVEIKRFLMQVTSDELSFTGCNFFTVTRTFMLTVAGTIVTYEIVLIQLNNVASGVPDQNNTINNYCSKLS  >ApisGr3  MSDENTFQPVYLKDRNFTESGYERQLKQSSLANVAVRSNYNALSLKENKGNIALEIQDDLGMPVNYVRIKALEDSIRKDIISDKNSLHRAITPLLILVQICALLPVQGIRGQNTSYLVFNWFSWIMSYSIILVCSSLLMILLIVFNLYRNGLTYEITGDLVFYGGTLINYMVFIHLAREWPKVMEKWELMEREMKQYGYPPNMAFKIKMLTCIIMLLSIIEHLASILTGVLKAIHCSPDGLDIFRLYILTSLETVFTFIDYSFVVAIILKFFNCLYSFAWNFMDLLIIVLACALTDKFKQLNQKLDSVRGKVLPSMYWRKSRETYNVLASLTHDFDEFLSPLILLSFGHNLYFICLQLLNSLKPMHSSWEALCFAFLFTYLVGRTCVVSLYVASINDQSKKPKTVLFSVPAECYGVEVERFLMQVTADELSFTGCNFFSVTRTFMLTVAGTIVTYEIVLIQFNNVASEIPDQNNTFANYCPKLL  >ApisGr4  MPDENTFRPVYLKDRNFTESGYGRPRQLKQSSITDSVVHSNYNALTSKENKRNIALEIQDKPVNYVRIKALEESIRKGIISDKNSLHRAITPLLILMQMSGLLPVQGIRRQNTSYLVFNWFSWILAYSIILVGSSVVMVSLAVYNLYSNGLSYEVTGDLLFHGGTLIVYTIFIHLAREWPKVMEKWELMEREMKQYGYPPNMEFRIKMFSCIILILSTIEHLASILTGVLKAIYCSPDRTDIFQHYIFIWLKTDFTYINFSFAVAIILKFFDCLYSFAWNFIDLLIIILSCALTDKFKQFNQKLAMVRGKVLPSMYWRKSRETYNILASVTHDFDEFLSPVILLSFGHNLFFICYQLLNSLKPMLSSWEALCFAFLFIYLVGRTCVVSLYVASINDQSKKPKTVLFSVPAECYGVEVERFLMQVTSDELSFTGCNFFSVTRTFMLTVAGTIVTYEIVLIQFNNVASGVHYQNNTLANYCPELL  >ApisGr5N  GIKKATSGSIFYGNACCALWMFIKLAKKWPKLMQDWRAVEISMRRFGAPRLGWKSTTLAVVLLVFAFTEHGLHNWLNTRPGGKDDIASMSLIDNDDNGSLILLDQSVCTYRYLERFSLKTHWYLYDDYDSYNPVKGFLVMWLSLTATFLWNFTDLFIMLVSSALAAQFKKLTKSMHSVRGQMLTMSQWQEYRETYTSLTHLVKKIDDHINVIVALSIGSNIYFICAQLITEIDSIKHSYFRTLFYMYSFMFLVFRTTAVVMQASAINDESKKIVPEIFMCPTHSYSIETQRFLQEVTSDYVALTGLKMFSITRNFLLGVAGAVLTYEIVLIQLQNTN  >ApisGr6  MLKNVIKVKLTNNFKRFKINPHKTVDIESSSCYFLDVPKIPSDSFRSATNFPTILGQYLCLIPISQDRFRIYSLRTALSVVALVCQIIMTFLSFCWLKETGANIFKGGVVLFFGGSAVTMALLINLSTNWCNLLDKWEQVEGYFGHQKNLRLKFTLISILCIIFSMVEHLSYLLTGIITTERENSHEVFEIYVLKMFPQVFTVITYNTWIAVLVLVINSVSTLTLCFSDQIIIMGSLALGNYFQIFNDRMKTHTGKTLTSDQWKTLRVDYTRLCNLTRLLNDCLCHLLCVSLIIHLYIICVELHQGVVRSNENYSLSHHVHAILSFLVSSLRGCLLCLCSVKIYESSKLPKRSLYDIPQQSFCNEASSVYNGSLVVEFFLLQIQKDHIGLTGSHMFLMTRRFLLTMFGTIVSYEILLVQMKGYSSRKNT  >ApisGr7  MKNKNLTRELLKTCTTTQTTRYFLLQFRDKIILFLRCVFKLHVSKILPNPDNLYRCPRGYLSQGGSSKRHRYAFDWRVVPVWLAYAALDAFIMYYNYLEASGYVGTSFRKCFEESYCIAVIELANRNLGPFSALVCTMFYGRRHKAAALAGTERVLVFIRDTDRDAATKYHSAALLSPVAKYYSAALMAAYAACIWLYTAAVPGDRHLSVLRQRAGPRRPIYNLIAFQYNVLGNGYARIRRLLEVAADASGHRARKSLAARVARLARASDDFGRQVGHLNRAYAMILLLKWPYNVVRIIMVVFRIIELSATVHEGRPFARVATVLIVEHVGEMVLFLVQLSYFCYTGARLSRQARANRTVASLQKLKLHHGSHLDLDVKKTVENFWIQMNARKINSTIGGFVIVNMNFMKAMCGIIATYFLVLIQFKSQKDKSKPLYLVWN  >ApisGr8  MFSCEMQIISDCYGYMRLTHVLTVCWGMPITRRRTDGVKSHRDSADVHGGYRYKFDPRVVPVWLAYVALDAFLIYYNYLEASGYVGESLRKCFEDSYCFAVIELANRNVGPFSALACIMFYGRRHKAAALAGTERTLAFIQDTDPDVADGGHVRPPDSRYYSAAVMAAYTACIWLYVAAVPAYVILAIWLNALGQTTVYGLATFQYHVLCRGYARVNKLLEMAADAVGHCGRPAPDCRSLATLVARLADTYEDFGQQVGHLNQAYVPGLLLRWPYSMVRLTMVVFRIIELSAVVKDDRPFAHVAAVLIVEHVGEILLFLVQLSSFCYAGACLSYQARAQNTTASLQKLKLCRGQSLYLNSDVKKKINIFLIRVNARKVNATIGGFVIINMNLMKAICGIIVTYFLVLVQFKSQKGKSKPLHFIWN  >ApisGr9  MKSNDDCYAYMRLTHLISVCWGFPITRRKIKSGYKYDGDTDVQGHRYSFDLRVTPIWLAYATLDAYVLYYNYLTASGYVGESFKKCFEESYCFAVIELANRNLGPFTALACTMFYGRRHKAAALAGTERVLAFIRDTDLDATALLSPAAKYYSAAVIVAYWACMWLYTVVQPAAVMAMFCANALCQTALYNLAMFQYYVLARGYARVNWLLEAAAQRPRAAGPVSAGRVARLADTCDELGRLVDHVNHAYALGLLLRWPYTIVRIIMLVFRIIEYAAIASPGNGHPKSRIAIALIVEHVGEILMFLQQLTIFCMTGTHLSDEANRTLASLQKLKLSHGSQLDLDMKKNIRIFWIQASARKVNATIGGFIFINMDLMKAVCGIIATYFLVLIQFKFQKSISKPLHISG  >ApisGr10  MKSNNSESYKYLRLTHVIAICFGLPYTRRKMKEDFNPDGNGGHLAGQYRYSFDKRLIPVWTLYACLNVYVVHYNYLEGVRYVAGSFRTCFEESYCFALIELLNRNLGPSSNVMCSMFYGRWHKIAGLAGAERTLQFLDDTRDAVPLANRRGLSRAVWYAAPASLAAYASILQFYMILVPPIVVAAYWFNGMGQMVVYILVFVQYYVLARGFNRVNDMVEALDVARDPVSAGRLLARLTSVYDELCTLAGHVNRAYAPEMLLQWAYNIIRVIMVVFRLMEFASSLDGPMAWATPYLLVQHLGELFIFVVHTSCVCTVGDRLSSESKRILASLEKLRLRNRVNMNIEIKKAVDIFYVRATTRNVYASIGGFIIFNMSLIRCVCSVLATYVLVLIQFRDQKGNSKPLYYNWN  >ApisGr11  MYHFSKVLLKVWQCMFIAPVTLRRKSAVDGSVQPQSYYAFSVWWYAFNLAVLVACVTGGVWAVMEDARASATGRSLRIQNTSSAIVTALQVTLQCLVCGLAITCSAGRHVMLQDIERHLNYADAVLRVSTSQPVARYTLALVTFHAALFAVDGYLWNTLSPATWLYGVCYVYMFIDLATMLMYAQIAWNIGRRFEDINAAIECKLAGFQAGAAATFDHSHRGRGYRPPQQVWTFSGQTTVVVSNNPVAMADRQYGTNMKNISIAKLQDLHWSLCNSIKIVNDKFGWQLFLQLFCNCVQLIVTPYFIIINLFYPVIYGSTDIKFILIQVLWVLTHLSHLLLIVLPTSYATTKLEIFVLQLQKYAIDFSACGIVNLHRSTITTIIGTALTYLVILIQFQNSD  >ApisGr12  MVWQSSSRVRKHRLHMWATLVYWMSKVAGVLVLAETAVGRREFTAAAASAGRWRWARARAWNVVCLAAVVHRIAVETWPVTVQAITHLPQASCYDTVCGGVWCVYACVYYAVAWLDRVSEICALAAAVTWRQLSTVECLNSASAGCLDDDDDDGDRAGKPSAFNIPWHAYLIVAFPVAQFAMAANVISLTHNGVWRAIMAAVAAAASCAPVAAHGTAAALLVMANNSMRSINCQITHLLPVGRLRRVRSSGDNLDADDTNIDENCRQRDIIAQLARRHWRITGLVTGGVCGAYSVDLMTAVLLAAVRVTYVAISVFHRLTDDSDARTNMSLAVVVQLIAWFGQFAYLAYTCDDLAAQIIGVTLTYFIVLLQFESSHSSSAHNDTYT  >ApisGr13  MPNGHTVLLFTIIAVQLYLAFDEVRSMWVEDDGTNHLHTLSTNMIGAISILLTALSRVRLRHIQHSLSCVEQSLHVKDNHSLGQRDNVVAVGHRTIEYRTLGWLALAAVVFGYLKYGLLMEYGRKSAYASAIVALLSTISLAGNYYVTLMFVDHVFFAKRVLYKLNVQIMDALSSVSASFLSGFSVPTARLAYLDICDVCTTLTRAFQTQLFINIVGNVFGITFMMLSAFNTLMSDGANTIRITLYFLYESLIRTLQMYFIIDACHTTVEQDILGRETRCFAQLQSIWYLVYYLVQLFILEMLDHKIEFVIYGFICLDFEMFVSIIGTIATYFLILVQLGSSPDVLVKSLNITTTTTTITRTTTPGAFSTKSNYTTLT  >ApisGr14  MISVVCFSSLLFGNWPLEWNEKNQRFEISYYFIFTSICMIICCCYSLFTLLDNIIEDDSIVSNPQYLIISMVYLNLGVLGLNRLMNAKHSARFAEELKRDSGRTVESGRHLPEFWLRTVVSAVFLCMLHYKYRKFTDMATTAIVVQTTLPLGCCVYASSTMESQYTAACSALAQEFASVNSRARGLHRDLQPDIRRNPRDQWAATERLARLSDAHCRLVRLVRLFNDEYGAPIFFTTAGLLLSQIYALNDIVSVVFVSDTFEATYERYAYVLDAVTWTFAWFRLWWICYRADDLTEQIHLTAKIMLENDISSYTENTRKMALDFLDQLLHRHVRITAWGFYDVNKELLFMVLGYQFGLCLICTQFTTSSIQLVNNLTTVQKNSTSSTENZ  >ApisGr15J  MNLRPFKWSPLAMIAPVYYACKLFGILPVSYANCGKIPYRNTIHFIGRRHENGEIIKTTESSYQNNDGVFTFLKLIYIYTLFSEKPPVLRIPRVMLVLYEIDYLYSLYSPNSKSLRDGCFHVSNSGLVLWATQLALQTWAFIRMCFYLGHTLMQRKSLNCSRDNYCSVFGMLLSHTVISPIVVSLVLIRLPRSVDVLNMTARLLLRSRYPRRRLSPNAYVVVLFSAAIVFKLVTTVMSIPQQYPDLYYPYFTAYMVPIVFINLVSILCVVAQQSYEDINRELEELCEVSSKTERAVRLNALMNDHWFVEDYMETMSDTFGPELIFTIMDIYVQLLLFMYVMIWDTVVRRVFKDNAYPYISGIIESSIIIGKFVYLCYRCDSSIKELRLFTLRVNSRAVQVTASGFFDINLSLLFATAGVVLIYFLVLVQFQMEGYKHILGSNSSDAVVAVVCHSWPCLERDDQD  >ApisGr16  MWLIRAFWRLCGVFTIDLSYNKFQNAYSALLIASCVYNFVNASQVICKVDHWCSTFSSTLTAVYDRVLTSAVFLSRIAVVYACKPNMSRYRATIRAFEAYSPPSPTELRRHRAFSLAVVAACLAVIVPTNSICMYYLCRYEPNSDASLFVYQLFMYVQNLSMCCIETQFVVQCFKVYTKFHGINGDLKRLKDENLNRSEYPFMSSAGSPPPPTSRAAVVYDKDFYRPRFMSHPTANTVELLRIKHWLIRLSIDALNNLFGVHMGLSVFYLWLMALFDIYYEMFHKSRSGLLVYCWLLQYTLRLLLIILMAHFTTKQALEAKSLIADTNNGTMDSSTKEELQLFINQIYSSTTEFNAYDFFTLNTQVIKSAIAAGATCLVILVQFHSERN  >ApisGr17  MTMPLQWLRCFPCNKKNIFQYHFSDKCQIIFSIVIIITCVFNLISAPLVVQCMVDDWSDVLSTSLTVLESRVVAIASFISRGIVLYNAYFNKYQKYRTTLESFSIYSPMTAAIRSQYKLYSIVTVSLCLTFMLPTNVVKLYSMYYKHPDGSLLVAHFFFFYLQNFSMYLIENEFANRCFVVYATFRDINDDLTRLKTEHIDRGRFPFLGKAADDSWSNAAPSPTSCVVVYDKDFYCPRDKENPLTNIVEILKIRHWLTREAVVDINYLFGNHLGLSILSLSVLVLLDVYTGVFHNSFANDRVDKKIFHSTLLFFTCVLQYSYRFCVITILSNVTTNQAVNAKTLITDINNRYLDTSTQEELQLFYTQISSRYIEFTACDLFTLNTRLITSAIAAGATYLVILVQFHSGKN  >ApisGr18  MWLVQSFWKLCGVFNTDLTYNKFQNVYSVLLIATCVYNFITASNTLCKLDQWCNVFSTAMIGMYTRVLASTTLLSRIAIMVQSKQSLLKYKETIKAFEMYSPTSSAQHKNYKIFSFAVVFVCLSIILPINISRLYYLYQNEAHDISLFIYFLFIYVQNLSMCCIETQFVTQCFIIYTRFRGINDDMKKLRNENVNYLKYPFIMGTSATIWKDYKKSFQCVRYDKDFYRPRFISHPMANAVEILRIKHWLTRQAVDILNNLLGIQMGLSVFLLWVMALFDIYYEIFHNSPSKLLVYGWLFQYTLRLFMIILVAHYTTKQAIKSKSIILDTNNQMLDNSTKEELLLFVNQIRHQSIEFTAYDFFTLNTQLIKSAIAAGTTYLVILVQFHS  >ApisGr19  MVINNNKMSTYWPITLFWKAFGVFPASVQGGRRNSNTSLPILSTVLLLASSLYALFAGTVVVCRIGEKCTSEAIRILKQIYPGIANVTSMLSRIALSYSVMVGFDKYAETMECYETYSPTTVAEANRYKMFTATAVCACLLLVIPVNAMRLWILWTNNADPTLAIIHFTFIYVQNLTMCCSETQFAEQCFVLYDKLKTVNDDVAVLGGPAGLARFSRHFRATAAAIPEAPSTAAALVPPGAADGAAAAVAVRAAQDPLDSVVAAAVTVETLRIRHWLLREAISCLNRLFGVQLGMSVCALGVMSFFDIYYETFHVMGVYTMSDLVIYSWMLHYAVRYVGIILMSHYTTKEALYTKTLIANLKSNCLDCRINQELHLFLDQLSHSSVEFTACDIFTLNIRLIISTFAAGLTYLVILIQYQTNMKV  >ApisGr20  MATTTNVRGGALMWPTACFWGACGVFTLDMAYNQFDRWFSASVLASGVLNIAITPWSVCVLDGWCDDHLSTTYRRLYTRFVAFTCLLSRATIVYKVRGHLADFRGREEAYERSWPSSGAHRRRYRAYASSVVAAYLALVVPINLTRLYLLYRYEEYGDSMLLLFFFNMYLQNWSMCCLETHFALLCFGVYLKFRAINDELSAVRSDVMVSNRYPVALRSSLPTLVTTATSILQDPCGRPMEAAVEELRLRHGLTRESVEQLNNMFGGQLALSLITLCIMILFDIYNEAFHVGGGISRSKFIFGWLLQYLFRFFVIVITAHSTTQEGYKTKVLVTEINNRHLNNNTKYELQLFLKQMNHQSIDITACDCFTLNGRLVASAIAVGTTYLFVLIQFHSEITQFTDNKKS  >ApisGr21  MTLLTFFTFILAIVLLITCVYNFVMSPLVLCLLDGDCAGVSFTSVQGIYPRMIAVACFLSFSTLLCKYCTIMPVYEKNIEAYEVYSPTTDIEHRNRSLFHLFLTVLYLSVILPFNILRLFILYKSDSGTMVLIFFVFMYLENIGMSMTETQFIIMCRTLDNKFFKINRDLEHLGREITHTSMSIVEKTVAVGHRVIYDGDFYGSNDHSIANAIEIIRIRHRLIRDAVYVLINLFAIPMGLSLFTLCVMTLFDIYYQVFSVMGADSRSLIFIYMWVLQYSIRFYIIVVSAHNTTKQALKSKILITDINNRYLDNNTKEELQLFLNQISSCTIEFTACDFFTLNTHLITSAIAAGTTYLVILLQFNSANN  >ApisGr22C  MFKFNVFVWRRRHVFDALCTFMLIIACLFNIALSPYCMCVMESCHEHELTVLVRVKTIMPQTVAVVCLVIRAKTMLHSANGEFREYENKAREYDACFPDRVGVAFVAFIVSAYAVTILPVNAYRLYLIHRDVRDHTVTAFFVLMYVQNMCTCSTEIHYVARCSRLYRRFCRINEDVSALKLATVAANRYPSVLLQSADGRDDDGRCRGTTSGVGPTAGDVELLRMRHQSVRDAVGDLNDLYGVQLLLSLCILCLMTVIDTYGEMFRKYTLSRSQAFLYVWLTHYSFRLCAIVLTTHFTMKQAYRTKMLVSDINNRILDISTKEELYLFSNQILRRSLEFTTCDFFTLNAHFITS  >ApisGr23P_N  AIACFVSTISNMIKSLTDFPKYNHNIKEYELYFPTNVSQKSTRNTFIIFIGFAYISIILPLNIVRLYLIHYNKHRIEILFFFTTMYVQNWSICLIEIGFISRCFGLYQKFQIINEDLIVLKTDTISKNKYPVVLRNEVQSDIINNPRFHTLANSIELLRMKHQLVRGALRDLNKLYGTQLGLSLVFLFILVLFDIHGEVITEGAKTRSKIFIYGWILQYTFRGFSIIITSHFTKKQZPNLGSLEFTSCNFFTLYPHLITT  >ApisGr24  MFRILPKWPILFWKFFSVIYIDFRYNKWQNVFSAFLIISSVYNFYMTPAVVCALDDRCDGSLSTVIKGLFIRVVSSTCVISRLVIFFKGNDLLIQYKKNIEKFHLFKPMTRFETVVLKKVSTRIVILCLLLTVPVNSFRLWKMSYRSDFTIVIFVCMYVQNISMYCVETHFTVLCYILYQKFVGINKDLMALKIDTVVRNKYPFMLRAREKCGKTINTIDYNRDILQTLAAGHSMANFIEQIKIKHRLAREAVNNLNDLFGIQLGLSMCSLCLFSMFDLYYHIRGIMNPSKSNILIYGWILQYSVRFGSITVLAHLTSKQALKSKILITDISNRYLDKNTKEEIQLFLNQMCSCAIEFTACDFFTLNNHLITSAIAAATTYLVILLQFN  >ApisGr25  MFNFFHNKLHRNIGLLISIFVILSCVYNYIEAPNVTCILNVCKNSTITMMVSMFPRAVAIACMISRITVMYKNVSVMAMYKKKIKEYEVYYPITVDKENSRRFLIIVIVFLYSILIVPINVYRMYLIYYYYKNIKITVYTLLMYIQNVSMSMTEIQFMVYCFGLYAKFQSINEEMSTIKSKTISINRYPFVLKSEERKSVEVYPSVRSIELLKMRHQFVCESVSDLNEIYSIQLGMSISVLFIMLMFDIYEAVTYELDKTKSLLLLYGWLTQYIFRFVVVILMSHITTKQGHRTKLLITDIHNRNLDSRTKEELRLFLNQVCNHSMEFTTFDFLTLNTHLITSAIVAGTTYIVILLQFR  >ApisGr26C  MFKFYLNKLLSSLNLVYFLALAMTCVMNLKNASATVCSLSECKNPVKTEVLAMFSRSVAIACFICRITIMYKSKSDFPNYVKKVEDYELNFPVKISQKRHIRFIAMAIISLYIVIILPINVMRIYLIYTNIGKINTMIFYTMMYVHNWSICSTEIHFIVRCVGLYQKFQSINEEMSTLRLKTIAGNKFPVVGQSERHDHDNTLLIGLETPGGGSPLSVSSNEHQPADHVELLRMKHQFVRGTVVELNDLYGIQLGLSICLLFMMTLVDIYGEVSVESNVTKTHVLFYGWLLQYSFRFCVIVLTSHITTTQAHQPKMIITDINNRYADNSTKKELELFLCQLSSRPVEFTICDLFTLNIRLITS  >ApisGr27  MFVFRNLTMFKFCSSKLRSYLEIMFSITLAMTCVYNCIHSSTYVCTLVTCNGDLITSVNSMFPRIIAIACFVSTISNMIKNLIDFPKYNHKIKEYELYFPTNVSQKSIRNTFIIFIGFAYISIILPLNIVRLYLIHYNKHRIEILFFFTTMYVQNWSICLIEIGFISRCFGLYQKFQIINEDLIVLKTDTISKNKYPVVLRNEVQSDIINNPRFHTLANSIELLRMKHQFVRGALRDLNKLYGTQLGLSLVFLFILVLFDIHGEVITEGAKTRSKIFIYGWILQYTFRGFSIIIISHFTTKQAYNTKMLLADINNRYLDKTTKEELYLFFDQIYQGSLEFTSCNFFTLHPHLITTALATGTTCLVILLRIN  >ApisGr28P_NC  DDRCDGSLSTVIKGLFIRVVSSTCVISRLFIFFKGNDLLIQYKKNIEKFHLIKPMTRFETVVLKKVSTRIVILCLLLPVPVNSFRVWKVSYRSDFKIVIFVCMYVQNISMYCVETHFTVLCYILYQKFVGINKDFMALNIDTVVRNKYPFMLRAREKCGKTINTVDILTSNILIYGWILQYSVRFGSITVLAHLTPKQ  >ApisGr29P_NC  ZVYIVIENVSMSMTEIQFMVYCFGLYAKFQSINEEMSTIKSKTISINRYSFVLKSEERKSVEVYPSVRSFELLKMRHQFVCESVSDLNEIYSIQLGMSISVLFIMLMFDIYEAVTYELDKTKSLLLLYGWLTQYIFRFVVVILMSHITTKQGHRTKLLITDINNRNLDSRTKEELRLFLNQVCNHSMEFTTFDFLTLNTHLITP  >ApisGr30  MFKLYFNKFLSSLKLVYFLALAMTCVMNLKNASATVCSLSECKNPVKTEVLAMFSRSIAIACLLCRITIMYKSKSDFPNYVKKVEDYELYFPVNISQKRQIRFITIAIISSYIVIILPINIMRIYLIYTNFGKINTMIFYTMMYVHNWSICSTEIHFIVRCVGLYQKFQSINEEMATLRLKTIARNKFPVVLRQSERHGHDNTLLIGLETLGGGSPLSVSSNGHQPSDHVELLRMKHQFVRGTVVELNDLYGIQLGLSICLLFIMTLFDIYGEVSVESNVTKTHVLFYGWLLQYSFRFCVIVLTSHVTTTQAHRPKMMITDINNRYADNSTKKELELFLCQLCSRPVEFTICDLFTLNIHLITSAFVAGTTYLILLLQFH  >ApisGr31  MLAIMCYLNTFYDNVGVVVSTMLLLSCVYSCIESSKFMCNLNNCNTSKSTIMISMFPRAVSIVCMLSKLTFMYNDLSATIKYKKNTKMYEMYHPLVDVDKANFRIFVIVMVSLCIIIVLPVNVLRIYLLYDKFRDGAMVLFFSIMYIQNASVCITELQFVAYCFKLYQKYRSINDDLAVLKSRTIITNRYPVVLNTEKYNSIGDSSGFNDDFCQEIRECQLVNSIESLKMRHRFVSNSASDLNDTYNLQLGLSLFILIVMMLFDIYEVVITEFIMIEMYWILYVWLLQYSFRFCMIVLITHLTTKQANKSKTILTDINNRHTDHNTKEELRLFLKELSSRSMDFTIYDLFILNPRLITSAIVVGTTYLVIFLQFS  >ApisGr32  MNNGAWRLFWKRLILVVAAAALMTSCVFNFVTAPLIMCLMDGGCEEVSVTSLRGMYARTIAVACFASASALLCKYGTTMSIYKQTMEAHDVYSPTTEAERRDRALFCVLYVTLCMSVTLPINALRLSLLYRNRSDPIVLVFFVFMYLENVVMCLGETCFVTWCRTLSNKFLVINRDLQLLGEEMATVNDTAAGKSITTALIATTVTTAAAGRRVTYGGDFYGGPREQSIANAVEVIRIRHQLIRDALSVLVDLFGLPVGLSLLTLGVMTLFDIYYQVSDIMGADSRLMIFIYIWLLQYTIRFSTIVLTAHYTTKQALKSKIFITDINNRYLDSNTKEEIFLNQLSNCSIEITAYDFITLNTHLITSAIVAGTTYLVILLQFDSNDD  >ApisGr33  MVLRDNPGTVMSAALLLSCLYNYVWSQEVICSLGDCRTKTTTIIMAMFPRAVAVGCMVSRAAVLYKNVTGAVAVYEGKIAEYEAHYPEDACDTRFRRALVTLIVATCAAIIIPVNAFRIHFLYRQYRSSAIIVFHVLMYVQNASTCVTELQFVVYCFGLCRMFCHINGDMSALRSETIVVNQYPPVLMTCRPLGATAELDRQALVERLKLRHQFISGAVRDLNTLYSFQLVASLSVLFAMSLFDIYDVVSNVVKAAEGHLFFYGWALQYAFRFSMIVLTTHVTTKQALKSKIFITDINTRLTDSNTKDELRLFLDELSGRSVEFTAFDMFIINSRLITSAIVTGTTYLIILFQFQ  >ApisGr34  MRRGWFGRLAGSAAQAVMGALCVLNFVMTPYFECQLDGDSCDENVSIMAGIYARSVSITCLVTVAVAWHKYRGAMVAYREHAELIDAYSVPATAAAASANSLYVDHATFSGVVLCVCMVLILPVNSFRLYRFVMDDRPVTAIVYFVLMYSQNIYVCLYETLFVRLFYALYTRYADLNRDMEAIGERIEDGRCARDEPPPGRDGWIPYDGDDRHRPHYFYYSPATGQPLEDAVERLRIRHRLIREAMDALKSAFAVPIGLSLCNLCVMVLFDVYYHLKNSVGQPAGDLARVYIFMWISQYTFRFFVITMTVDVTVKQALRSKETITDVSRHCLDISTKEELQIFSNQISSTVIEFTMCDLFTLNARLFTSAVGVCITYLVILLQFKIKADNVY  >ApisGr35  MFGLQHFGVISFDSNNDNNIYQIAYTVILFITGISLIIVFPLVNDKFDDWSKAFSMSLTVVTCSSGIITSLISRMIVVYNIKFKYQKFKTTLEGFEIYIPMNSVASKHIKYFSFSVIFFFMSFIIATNSLRLFYIFNNHVNPFLMTTFFGLYYMHNLSMVCTELHFSIQCFLVYTKFRDINEKLIQINDEQKYYNFNVRYSFTGTQVATPRNSDDKSPPCVIMYEKDFYCPKDKSFPLANTIELFRIRHWLSREAINDIKCLFGFQMGMSIIILAVTVLFDIYTELFYSYTNSSFSKSVFRSKILFIGWMLQYSTRLGLIIVTAHTTIQQAVKTKKLITEMNNRHTDSNTKEELQLFYNQLSICSPEFTIFDILTINNSLITSMMSAGATYILILVQFQSEKTTHKNHYLSNRP  >ApisGr36  MLYLKYFGAIFFDSNNDNDMCQIVYSVVLFITGIIMAIMSPYVVLEFDDWSEAFSMSMSLIVCTIVNISSCISRMIVKYNVKFKYQKYKTTLEGFEIYIPTNTVALKHIKYFSFVVISWCMSVIIPINGLKMYYIFNNHVHPILMTTYFFFYYMHNLSMVCTELHFAIQCFVVYTKFRDIHDKLIQINDEQNYYNYNVRYPFTVNRMTPKRNDATSPCDIIYEKDFYCSKDKVSPLANTIELLRIKYWLTQEAVNDLNGLFGFQMGLSIICLTTLILFDIYTEVFYSRAYSAYYIPVFRSKFLFVGWMLQYSSRICIIIFTAHTATKQVVKIKKLIAEMNNRYLDCSTKEELRLFYYQLSICPSEFTIFNTFTINNGIITSAIAAGSTYILILVQIHSKK  >ApisGr37  MNVFITTLKPILLLCKIVGIIKTSYYFESNGLLVKNTNSRYHSLFEFSKTVVFLVLTYYSYRKLEFLETLNIYIYWTAIITSRISETWIIKLINGIIEFDQKLTSLPTRLQAHQRQLNKQYWNRIFICTTLYYIVITLFYMYLWPVKIIDINIVILYFVRVRFIVDFTVIVSSHFYLKNLEHRFELLNDFWKCLPAGLLDVTGECSHHDIAMMVENIRLLHAELSDVLRIFSMGYGHILLGFFVLNYINILVHIFYSICFNFSTSADHNAMMEKIMKESVQYIFYLQSIIFTMSILTAASRVNDKKRQIISYLRLTRISNLSTTLKIQIKMFMNQVSVFESDEMTAYGFFSINLNLVMSIIILVVSGTTTLIQMKEHPYIMKTINVTRNFYRQLNISL  >ApisGr38F  MNVFITTLKPVLMLCKIVGIIKTSYYFESNGLLVKNTISRYHSIFEFSKTFVLLVLTYYSYRKLKFLETLVLYIFWTVIITSRISETWIIKLINGIIEFDQKLTSLPTRLQAHQRQLNKHYWNRIFTWGILYYIAITAMHVYFWPIETIDINIIILYFVRIGLFVDFTVIVSSHFYLQNLEHRFELLNDFWKCLPAGLLDVTGECSHYDITMMVDKIRLLHAELSDVLRIFSMGYGQMLLSYFVFSYISMMICLYYVICFNFSTSSEDNPTIEKIMKQSLLIIIFLQNIIFTMSIVTAASRVNDKKRQMISYLRLTRISKLSTALKIQIKMFMNQVSVYESDEMTAYGFFSINLNLVMSIIILVVSGTATLIQMKEHPYIIQAINATKDFYRQYNITV  >ApisGr39  MNVFITTLKPILMLCKIVGIIKTSYYFESNELLVKNTNSRYHSLFEISKTFVLLILTYYSYRKLEFFETLNLYIFWTVIITSRISETWIIKLINGIIEFDQKLTSLPTRLHAHQRQLNKQYWNRIFTWGTLYYIAVTVIQVYLWPIETIDINIIIIYFVRVGFFVDFTVIVSSHFYLQNLEYRFELLNDFWKCLPAGLLDVTGECSHYDITMMVDNIRLLHAELSDVLRIFSMGYGQMLLGYFVFSYISMMICLYYVICFNFSTSSEDNPTIEKIMKESVSIIYFLQNLVFTMSIVTAASRVNDKKRQMISYLRLTRISKLSTALKIQIKMFMNQVSVFESDEMTAYGFFSINLNLVMSIIILVFSGTATLIQMKEHPYIMQSINATKNFYQQLNISL  >ApisGr40  MNVFITTLKPVLMLCKIVGIIKTSYYFESNGLLVKNTNSRYHSIFEFSKTFVLLVLTYYSYRKLKFLETLVLYIFWTVIITSRISETWIIKLINGIIEFDQKLTSLPTRLHAHQRQLNKQYWNRIFTWGTLYYIAVTVMNVSLWPVKIIDMNIIILYFVRVGFFVDFTVIVSSHFYLQNLEYRFELLNDFWKCLPAGLLDVTGECSHHDITMMVDNIRLLHAELSDVLRIFSMGYGQMLLGYFVFSYISMMICLYYVICFNFSTSSEDHPTIEKIMKESVLIIYFLQNLVFTMSIVTAASRVNDKKRQMISYLRLTRISKLSTALKIQIKMFMNQVSVYESDEMTAYGFFSINLNLVMSIIILVVSGTATLIQMKEHPYIIQTINATKNFYQQYNITV  >ApisGr41  MNAFVITLRPILMLCKIVGIINTSYYFESNGLLVKNTNSKYHLLFEFSKTVIMLIFTYYNLKILSFIDSLILYKFWTVIITSRISETWIIKLINGIIEFDQNLTSLPAYLQSHRRQYNKKCLNTIFICASLYYTAITLLLMYLYPIKKMDINVIFIYFVRISFIVDFTVIVSSYFYLQNLQYRFEILNDLWKCLPAGLLDVPGECSHYDIAMMVDNIRLLHAQLSDILRIFSLGHGQMLLGYFVFSYINMLVFFFFTFYFKYPTSLNYNQNVDMNMILKKTVPVVYNLQNIIFTMSIVTAAARVHDKKRQIISYLRLTRISNLSIPMKIQIKMFMNQVSVFESNEITAYGIFSINLNLVISILILLISGITTLIQMNEHPFIWQTINSTVNFYKKLNISN  >ApisGr42  MTSVYKTTLRTILYLCKFVGIINITYILNSDGLLIQSTDSIYKCLEIARMFMLIISTFSIHMSTYFTQKIFLFKLWSVIIASRISETWVIQLMNGVIEYDQKLTSLPRCLMIRQLLPKKNCWNIIFIFTLIYYIANTFLMVYLWPPKTMDITTIVLYFIRLDFIVDVTVIFSSYFFLQQLEYRFQMLNDSWEYLLPGFLAVPEELTYSITRITLDNIRLLHAELSDLLRIFSEGFGKMLLGFFVFSFINMILSFYYSIIPSSSVLKEFNFDSYLIACIPYLLNLQNVIWTLSIIIAASRVHEKKRKMISYLRLIRISNLSANLKTQVKFFMNQISVFESSELTAFGIFNINLNLVVSILILLVTGFITMIQMKEHPVLLQSKENLKKFIQSLTKE  >ApisGr43  MTTVYKITLRNILYLCKFIGIINISYILESDGLLIQSTDIIYKCLEFTRMIVFIIFTYYIHKNTAFHHTIYLCNVWSVIIASRISEKRVIQLMNGIIEYDQKLTSLPRFLFIPQHSQKKNYWNIVFIFTSIYYIMMTLIAFKIWPPKTIDIFTISLYVVRLEFIVDVAVIFSTCFFLQQLDYRFQTLNDSWQYLLPGFISAPAGELTHSITGMTLDKIRLLHAELSDLLRIFSAGYGQMLLGFFVFSYSNTVLSFYYMIHYNNSKREEFTFTYFLKTFIIYMASLQNVTFILSIIIAASRVHEKKRKMISYLRLIRISNLPTNLKIQVKLFINQISIFESDEITAFGIFNINLNLVVSILVLLVTGLITLIQLKEHPFIKQSVDNLNNYIQNASNGTFK  >ApisGr44  MTNVYKITLRNIVYLCKVIGIINISHILESNGLLIQSPDIIYKFLEFTRMIVLIIYTYYIHKNTAFHQTIYLCNAWSVIIASRISEKRVIQLMNGIIEYDQKLTSLPRFLFIHQHSQKKNYWNIVFIFTSIYYVVMALIAFKVWQPKTIDIFTITFYFVRLEFIVDVALIFSTCFFLQQLNCRFQTLNDSWQYLLPGFISAPAGELTHSITGMTLDKIRLLHAELSDLLRIFSGGYGQMLLGFFVFNYINTVVGFYYMIHYNSTKTEDFTFTYFLKTFIIYMTSLQNVIFILCIIIAASRVHEKKRKMISYLRLIRISNLPANLKIQVKLFMNQISIFESDEITAFGIFNINLNLVVSILVLLITGLITLIQLKEHPFIKQSVNNMVNYVQNKSKGTFK  >ApisGr45  MSTVFQTTQRPVLFLCKVVGIINISYTTGPDGLLIQNTNSKYRSFLELTRMIVLIIITWEVQKRILLPQKISIFKCWAVIISARISEKWIIKMINGIIEYDQKLSSVLTVYFEHRRPIEKKCWSIIYCCTFVYYVAISMILMWILPMKEINIMTISFYFIRWGTIVDIAVVISTYFYLKNLECRFQTLNNFWTQLPDGFTTIPVIVGGWSNPEIVTMVDNVRRLHAELSNLLRIFSMGFGQILLAFFVFNYIDIIFNFFNWIHFIGHNFWTVNVNDVLIKLLMFMTYLQNLIFVMSIIIAASRVKDTTLKFCNRSRHHFLFSLRFGFPGNVRYFMSQLTVFDLDEITAFGIFNINLNLVMSIIILLITGFITVIQMKNQPFTLHFINDTLEFYTNLTKRFKNTTNILS  >ApisGr46  MTTVFHITLRPILFLMKCMGIIDISYTMESTGVLVKNINSTFPAFLEISRMIVLLICTYIYLSQYEPEFYVLQIIKILQFWNVIIAARLSTFWINKFINGIIEFDQKIAPLSSHLLIPQRSWKKIKWDMIIISLFAYFIGFKIVQLYFIPLKAISIEFLVHHILFNIPFLMDYVVAISLCFFLQNIYVRFQTVNDIWKCLPADLVPVSDQWTHIEIVVLMENTRLLHAKLCDFLKMFSLGYGPMLLGFFIFSFINMLFSVYIVLNFGALSSSNSITSIFKNILVMLIYVQCVTFSMSIVTFVSFINEMRLEMVSYLRLYRISNLHLDIKRQIKMFMNQISVCDSDQISAFGFFNINLNLVTSVLVLLISGTTTLIQMKDHPIILKLNNDTYSFFQKLSV  >ApisGr47  MTTVFQITLRPILILCKCIGLIDISYTVKPTGFLVQNRTLTFPVFLEIARMVVLFICTYLYVYQFKQGWFVIQMINIFNFWIIIIAARLSTKWIIRFINGIIEFDRKIIPLTTNLATPQRSWTKKHWNTIFILLFVYFIGFNMFQYYCLPYQIENIIELSTRVISIIPNVMDYAVTVSSCFFLQNLYVRFQTLNDFWKCLPDDLVASPGQWTHFKIVDLMENTRLLHSELCDLLKMFTLGYGLLLLGFYITSYFNLLISIYFIVNSEVFSSFHPTNHNWVKLLPILAHAQIVTFLMSIIVFASSINDKRLKMISYLRSYRISNLHLDKKRQIKMFLNQISACDSDQISAFGFFNINLNLVTSILVLLVSGIITFIQMKNHPMMLKLNNDTKSFFKQLYIKKN  >ApisGr48F  MTTDFHITVRPILFLSKFMGLIDISYTLEPSGLLVQNKNSNVHALLEIARMIVLLVCTYIYFNQYEPEFHILQIISIVQFWIIIIAARLSTIWIIKFINGIVEFDRKITPLSTNLLIPQRSWTKTHWNTILISLFTYFIGFKFLQIYFRSFKIGSIELFIHHVIFTVPYVMDYVVTISSCFFLQNMYARFQTLNDLWKCLPADLVPISDQWTHIEIVVFMENTRLLHAELCDLLKMFTLSYGPMLLGFFIFSFINMLFSVYIIFNHGSLSSSFSINHSLQIVALLVHVQIVTFLMSIVVFVSFINEKRLKMISYLRLYRISNLHFDIKRQIKMFMNQISVCASDQISAFGFFDINLNLVTSILVLLISGIITLIQMKDHPIILKLNNDTYSFFQKIRTRN  >ApisGr49  MTTDFHITVIPILFVSKFMGLIDISYTVGPSGLFVRNKNSNVHALLEIARMIVLLVCTYIYFNQYEPEFHILQIISIVQFWIIIIAARLSTIWIIKFINGIVEFDRKITPLSTNLLIPQRSWTKTHWNTILISLFTYFIGFKFLQIYFRSFKIGSIALFIHHVIFTVPYVMDYVVTISSCFFLQNMYARFQTLNDLWKCLPADLVPISDQWTHIEIVVFMENTRLLHAELCDLLKMFTLSYGPMLLGFFIFSFINMLFSVYIIFNHGSLSSSFSINHSLQIIALLVHVQIVTFLMSIVVFVSFINEKRLKMISYLRLYRISNLHFDIKRQIKMFMNQISVCASDQISAFGFFDINLNLVTSILVLLISGMITLIQMKDHPIILKLNNDTYSFFQKIRTRN  >ApisGr50I  MTTVFQITLRPILILCKCIGLIDISYTLEPTGFLVKNITLTFHVFLEISRMIVLLICTYLYLHQRKQELYVFQMLNIFKFWIIIIAARLSTKWIIKRLKMISCLRLYRISNLHLDKKRQIKMFLNQISVCDSDQISAFGFFNINLNLVTSILVLLVSGIITLVQMKNHPMILKLNNDTKSFIRQLYNINKKN  >ApisGr51  MTTVFQITLRPILILCKCIGLIDISYTVDPTGFLVQSRTVTFPVLLEIARMVVLLICTYLYLHQFKQDWFVLQTIYIFNFWIIIIAARLSTKWIIRFINGIIEFDQKIIPISTNLVTPQRSWTKKHWNTILISVLGYFIGFKILYYYYMPFQIENIVVLLTHTIFGVPFVMAYAVTVSSCFFLQNLYARFQTLNDFWKCLPADLVATSDQWTHIKIVDLMENTRLLHSELCDLLKTFTLGYGPLLLWFYTTNYINLLFSVCLILNIEVLSSSHSIKHNWMQLLPLIVYVQIVTFMMSIIVFVSFINEKRLKMISYLRLYRISNLHLDKKRQIKMFLNQISVCDSDQISAFGFFDINLNLVTSILVLLVTGIITLIQMKDHPMILKLNNDTKSFMRQLYNIKKEN  >ApisGr52J  MTTVFQITLRPILILCKCIGLIDISYTLEPIGFLVQSRTLTFSVFLEIARMVVLLISTYLYVHQFKISWFVIQTLNIFKFWIIIIAARLSTKWIIRFINGIIKFDRKIIPLSTHLVIPQRLWTKKHWNTIFISIFAYFIAFKSFIFYIHPVKIKYFVSWTYGALFTIPFVMDYAVTISSCFFLHNLYVRFQTLNDFWECLPADLVATSDQWTHIKIVDLMENTRLLHSELCDLLKMFTNGYGPLLLGLYTSSYINLLISIYLIVNSEVLASLNSTKNNWEQLLPLLVSVQIITFLMSIIVFVSFINEKRLKMISYLRLYRISNLHLDKKRQIKMFMNQISVCDSDQISAFGFFNINLNLVTSILVLLISGMITLIQMKDHPMILKLNNNTKSFIRQLYNIKKEN  >ApisGr53C  MTTVFHLTLRPILILSKCIGLIDVSYTVEPTGLLHRNRNSKIRIFLEIARMIVFLLFTYAYFYQFHQELHILQIIYVVKFWFIIVAARMSSVWTIKFINGIIEFDRKITPLSTDLMTPNRSWTKKRWQTVFISLWAYFIGFKLIFFYFWPIQIKNIVLLANHAIFTIPYVMDYVVTISSCFFLQNLYVRFQTLNDFWKCLPADLVDVPGQWTHIEIMDLMENTRLLHSELCDLLKMFTLGYGPMLLGFFTSSYISLLLSFYFILNKETFFGPTAKIWDLTFPLMVHVQTITFLMSIIVFVSFINEK  >ApisGr54P_NC  FINGIKEFDQKIIPHSTNLVTPQRSWTKKHWNTILISVLGYFIGFKTKKNLALLFPLILHVQIVTFMMSIIVFVSFINEXXIEIEMISYLRLYRISNLHLDKKRQIKMFLNQMSVCDSDQISASGFFNINLNLITSILVLLVSGIITLIQMKDHPIILKLNQGLKPIEIISVLVSILYTGFEIL  >ApisGr55  MTTVFQITLRPILILCKCIGIIDISYTVEQTGFLVQSRTLTFPVFLEIARMVFLLICTYLYLHQFKQGWFVLQTLHIFKFWVIVIAARLSTKWIIKLINGIIEFDRKIIPLSTNLATPQRSWTKKHWNTIFISLFGYFIGFKILYYNCRPFQMENIAVLTTHAIFTIPFVMDYAVTVSSCFFLQNLYVRFQTINDCWKCLPANLVATSDEWTHTKIVDLMENTRLLHSELCDLLKMFTLGYGPLLLGFYTTNYISLLFSVYLIVNSEEFSSMYSTKNNCVQLLPLMVHVQIVTFLMSIIVFASLINEKRLKMISYLRLYQISNLHLDKKRQIKMFLNQISVCDSDQISAFGFFNINLNLVTSILVLLVSGIIALIQMKDHPMILKLNNDTKSFMRQFYHLKKEN  >ApisGr56  MMTVFQIILRPILILCKCIGLIDISYTVDQTGFLVQKRTSTFNVFLEIGRMVVLLICTYLYVHQFKQDWFVLQTLHIFKFWIIIIAARLSTKWIIRFINGIIEFDQKIIPLSTNLVTPQRSWTQKHWNTIFISLFGYFIGFKILYYYSLPFHLENIVILTTHAIFTIPFVMDYAVTVSSCFFLQNVYARFQTLNDFWKCLPADLVATSDKWTHIKIVDLMENTRLLHSELCGLLKMFTLGYGPLLLGLFTTSYINFLVGVYFIVNNEVFPSFHSTKNNLVRLLHLMVHAQIVTFLMSIIVFVSFINEKILKMISYLRLYRISNLHLDKKRQIKMFMNQISVCDSDQISAFGFFNINLNLVTSILVLLVSGIITLVQMKDHPMILKLNNDTKSFNYIISIILY  >ApisGr57I  MMTVFQITLRPILILCKCIGIIDISYTVEQTGFLVQSRTLTFPVFLEIARMVFLLICTYLYLHQFKQDWFVLQTINIFNFWIIIIAARLSTKWIIRFINGIIEFDRKIIPLSTNLATPQRSWTKKHWNTIFILLFGYFIGFKILYYYCIPFHLENIVVLTTHSIFTIPFVMGYAVTVSSCFFLQNLYARFQTLNDFWKCLPADLVATSDEWTHTKIVDLMENTRLLHSELCDLLKTFTLGYGPLLLWFYTTNYINLLLSVYFIVNREAISHSTKNNWTRLFPIIVHVQIVTFLMSIIVFVSFINEKXFFDQISVCDSDQISAFGFFNINLNLVTSILVLMVSGIITLIQMKDHPVILKLNNNTKFFMKQLYDLKKKN  >ApisGr58NC  FINGIIEFDQKIIPLSTNLMTPQRSWTKKHWNTILISLFGYFVGFKILYYSCRPFPLENIVVLTMHAIYSTPFVMDYAVTVSSCFFLQNLYVRFQTLNDCWKCLPADLVATSDEWTHTKIVDLMENTRLLHSELCDLLKLFTLGYGPLLLGFYTTSYINLLLSVYLIINSDVFYSLNPTKNTCATSLPLIVHVQIVTFLMSIIVFVSSINEKRLKMISYLRLYRISNLHLDKKRQIKMFLNQISVC  >ApisGr59  MTTVFHVTLRHILILSKCIGLIDISYTVEPTGLLDRNSINSTIYIPLEVARMIVLITCTFIYFYQFDQGLNILQYIYVVKFWFIIVAARISTIWIVKFINGIIEFDRKTTQLSTNLMTPNRSWTKRRWETIFISLCAYFIGFKFLQYYFWPVQIENIVLLATHSIFTIPYVMDYVVTISSCFFLQHLYVRFQTLNDIWECLPADLVAIPGQWTHIEIVNLMENTRLLHSELCDLLKMFSLGYGPLLLGFFTSSYIGLLFSVYYIVNKETFFTSYENAWEHILPLTIHLQILVFLMSIIVFVSFINEKRLQMISCLRLYRISNLHLDKKRQIKMFMNQISVCDSDQISAFGFFNINLNLVTTILVLLVSGIITLIQMKDHPMMLKFNNDTKSFFLKIYPKKYD  >ApisGr60  MTTIFHITLRSILIISKCMGLIDISYTVGPTGLFIRNINSTFYVFLEIARMIVLLICTYLYFHQFDPDFHIFQYISIFKFWIVIIAARVSKIWIIKFINGIIEFDRKITPISTNLLITQHSLKKKQWDRILISFIVYLIGFKSLQLYLYPMKKVNISSLVQSLVFSPPYFMDATVTITSCFFLQNLYVRFQTIIDFWKCLPTELVAVPGQWTHTEIVVLMENTRLLHSELCELLKTFTQGYGPLLLGFYTFSYITMLVGVYFIVNNNLLSSANTAEKFRVVIPLVTHLQMFSFMVSIIVFVSFVNEKRIKMISYLRLYPISNLHLDIKRQIKMFMNQIPAYELNRISAFGFFDINLKLVTSIIVLLITGISTMVQMKDHPMILQLNNDTKSFLIKLFKTS  >ApisGr61C  MTTIFHITLRSILIISKCMGLIDISYTVGPTGLFIRNINSTFYVFLEIARMIVLLICTYLYFHQFDPDFHIFQYISIFKFWIVIIAARVSTIWIIKFINGIIEFDRKITPISTNLLITQHSLKKKQWDRIFISFIVYLIGFKSLQLYLYPMKKVNISSLVQSLVFSPPYFMDATVTITSCFFLQNLYVRFQTIIDFWKCLPTELVAVPGQWTHTEIVVLMENTRLLHSELCELLKTFTQGYGPLLLGFYTFSYITMLVGVYFIVNNNLLSSANTAEKFRVVIPLVTHLQMFSFMVSIIVFVSFVNEKRIKMISYLRLYPISNLHLDIKRQIKMFMNQIPAYELNRISAFGFFEINL  >ApisGr62F_N  FINGIIEFDRKTTPLSTKLMTPNRPWTKRRWETIFISLCAYFIGFKFLQYYFWPGHIENILLLATHSIFTIPYVMDYVVMISSCFFLQHLYVRFQTLNDILECLPADLVVIPGQWTHIEIVNLMENTRLLHSKLCDLLKMFSLGYGPLLLGFFTSGYFGLLFSVYYIVNKETFFTSYENAWEHILPLTIHLQILVFLMSIIVFVSFINEKRLQMIPCLRLYRISNLHLDKKQQIKMFMNQISVCDSDQISAFGFFNINLNLVTRILVLLVSGIITLIQMKDHPMMLKFNNDTKSFFLKIYNKKYD  >ApisGr63P  MTTDFHITIRPILFVSKFMGLIDISYTMGPSGLLVQNKNSNVHAFXIARIIVFLVCTYICFNQYEPEFHILQILSIVQFWIMVIVARLSTIWISKFINRIVEVDRKITPLSTNVLIPRRIZTKKHWNTIFISLFTYFIGFNFLQMYFGSFKIESISLSMQHVIFSAPFVMDYVVTIALFFFLQNMYARFQTLNDLWNCLSADFVPISDQWTHIKIVVFMZNTRLLYVELCDLLXMFTLSYGSMLLSFFIFSFINMILSIYIVFNHRVLSSSPSINNFIQILPFMVNVQIVTFLMSIIVFVSFINEKRLKMISYLRLYRISNVHVDIKRQIKMFMNQISVCESDRISAFGFFEINLNLILVLLISGMITLIQMKDHPMIWKLNNDTFSMFTKYAYKKN  >ApisGr64N  VTVSSCFFLQNLYVRFQTLNDFWKCLPADLVATSDEWTHTKIVDLMENTRLLHSELCDLLKTFTLGYGPLLLGLFTTSFINLIVSVYFIVNSEVFTSFHPNRNIWAQLLPLLVHVQIVTFLMSIIVFVSFINEKRLKMISCLRLYRISNLHLDKKRQIKMFLNQISVCDSDQISAFGFFNINLNLVTSILVLLVSGIITLIQMKDHPMILKLNNDTKSFLRQLLVIDRKAN  >ApisGr65P  MTTVFKSTLVPVLALSKIFGLINISYILDPDGLLTLNLHSTYYYPFLEYTRMIVLLIFTYKVFTEDMYYIVHYRLVKFWIAIIAARLSEIWTVKLINGIIQFDRKLALLSPAFPVHQRSISKKKWNIIFTSLFLYFVSYEVFDIYLWPPNTFDINTFLIFFFGMPFVLDYVVIITVCFYLSNIGCRFQTLNDFWTCLPHGMVSVPSEWTDSELVMLMESIRLLHAELTQLFKIFSSSFGTLLLVFFVCCIIDIIYVIYLMIXLENVIKHVPLHMLNIQIVVFLMSVILAASWINEKKMKMVSYLRLTPISKLPVEVKLQIKMFLYQITMLESDGISAFGFFNINLNLVVSMAMLLMTGFSTIVQMKNHPIILALVNNTHMYHSIWVRNLK  >ApisGr66I  MTSVFKSTQLPILILCKVFGLINISYTFESTGLLVQSSTNTTQYALLEITRMCVLIMFTYIVYCRGFYYIVYFRLVKFWIVVIASRISELWIIKLINGIIEFDQKLALLSTAFMVRRRSLSKKRWIIIAASLFLYLIGFEVCALNLWHLKTLDISTIPVLFFGMPHITDFVVIITVSFYLNNLNYRFQTLNDFWKCLPTGLIPNRGEWTHPEIVMLVESIRLLHAKLSEILKIFNLSYGLLLLGFFVCSFIDFMYIFYLMIYHELASPKVSFTQNIIKYLPLHIFTVQIIAFLMSIIVAVSWIKEEKNRIISYLRLCRISNLPVDTKLQILILLITGLASLIQMRNHPIILTMINNTLSFYTIWVIKILNNKNTSNFLKC  >ApisGr67P  MTSVFMETLNLFITASKIVGLMNFCCTMESGLLCRSTKSPYYIFLECIRMFVYLICTYHIVFNMGIVYILIHFNVLKYWSIVTTARISEKWIIKFINGIIEFDNKLFLFNSLFPVKLYSISKILWNIIFAIFVLFYLCCLYFFIGPNGLVSGXVKNLSHYVTVLFYSPEIIIFVVLITSLYDLSNLGYRFCELNRLWKCLPFGLLALPGGWTNSEITMLVERIRLLHAELSELLRLFSLGYGPVLLVYFTFTFANALIETFLITMNKDSLNNNFFPYIFYLQHIFNMVSIIXYVTSWVIEKKKKIISCLRLCRISEMAVHTKLQIKIFMNQISKYEPNEITAFGFFNFDLKLTMSILVLLITAISTMLQMKDHPWMLYLKNAWIANSEFMKANT  >ApisGr68P  MTRVFMETLNLFITASKIVGLMNFCCTMESGLLCRSTKSPYYIFLECIRMFVYLICSYHLIFNMGSVYILFHFNVIKYWAIVITARISEKWIIKFINGIIEFDRKLFLFNSSFPVKLYSISKIFWNILFAIFFLFFLYTLYFYIGPNGFKKVKDISHYLAIVFYSPEIIVFVVLITSLYYLSNLGYRFCELNRLWNZLPFGLLALPGGWTNSEITMLVERIRLLHAELSELLRLFSLGYGPVLLVYFTFTFAHALLETFLITIYKDSLKNIFPYIFYLNHIFNMISIIYVTSWVIEKKRKIISCLRLCRISEMTVHTKLQIKIFMNQISMYEPNEITAFGFFNFDLKLTMSILVFLITAISTMLQMKDHPWMLYLKNAWKAKSDYLENNNL  >ApisGr69  MTSVFMETLNLFITASKIVGLMNFCCTMESGLLYRNTKSPFYIFLECIRMFVYLICTYHIIFNIGSFYILVHFNVIKYWAIVIAARISEKWMIKFINGIIEFDSKLFLFNSSFPVKLYSIRKIFWNIIFAIFFLSFLCCQYFYIGPNGFTIVKDLSHYVTVVFHSPDIIIFIVMITSLYYLTNLGYRFCELNRLWKCLPFGLLALPGGWTNSEITMLVERIRLLHAELSELLRLFSLGYGPVLLVYFTFTFTHAVLETFLFTINMDSLKNNIFPYIFYLQHIFNMISIIYVTSWVIEKKKKIISCLRLVRISEMSVHTKLQIKIFMHQISMYEPNEITAFGFFNFDIKLTMSILVLLITAISTMLQMKDHPWMLYLKNAWTVNSKAWNRN  >ApisGr70P  MTSVFMETLNLFITASKIVGLMNFCCTMESGLLYRNTKSPLKMFLECIRMFVYLICSYHIIFNMGSFYILVHFNVIKYWFIVITARISEKWIIKFINGFIEFDSKLFSFNSSIPVQLYSKRKTFWNVIFASFILYFLCCQYFLIICFGTTIKDLSDYVTVVFHSPDIIIFIVLITSLYYLTNLGYRFCELNRLWKCLPIGLRALPGGWTNSEITMLVESIRLLHAELSELLRLFSLGYGPVLLVYFTFTFAHALIETLLFTIYKDSLRNNILPYIFYLQHIFNMISIIYVTSWVIEKKKXIISCLRLVRISEMSVHTKLQIKIFMNQISMYEPNEITAFGFFNFDLKLTMSILVLLITAISTMLQMKDHPWMLYLKNAWIVNVYNKQTNKD  >ApisGr71  MTSIFMETLNLFITASKIVGLMNFCCTMESGLLYRNTKSPFYIFLECIRMFVYLICSYHIFFNIGSFYILVHFNVIKYWAIVIAARISEKWMIKFINGIIEFDSKLFLFNSSFPVKLYSIRKISWNIIFAIFVLFFLYCQYFLFGPDGRIINDLSHYVTVVFQSPEIIIFVVLITSLYYLSNLGYRFCELNRLWKCLPFGLLALPGGWTNSEITMLVERIRLLHAELSELLRLFSLGYGPVLLVYFTFTFAHALLETFLFTIYEDSLKNNIFPYIFYLQHIFNMISIIYVTSWVIEKKKKIISCLRLIRISEMTAHTKLQIKIFMNQVSMYEPNEITAFGFFNLDVKLTMSILVLLITAISTMIQMKDHPWMLYLKNALMANNLI  >ApisGr72JP_  MTSAFMETLNLFITASKIVGLMNFCCTMESGLLYRNTKSPYYIFLECIRTFVYLICSYHLIFNMGHIYLIFHFNFIKYWAIVITARISEKWIIKFINGIIEFDSKLILFNTLFPVKLYSISKILWNIIFAIFVLFYLCCLYFFIGPNGLVTVKDLSHYVTVLFYSPDIIIFVVMITSHYYLSNLGYRFCELNRLWKCLPFGLLALPGGWTNSEITMLVERIRLLHAELSELLRLFSLGYGPVLLVYFTFTFANTLLETFLFTILEDILENNILPYIFYLQNIFYMISIIYVTSWVIEKKKNIISCLRZVRISEMTVHTKLQIKIFMNQISLYEPNEITAFGFFNFDLKLTMSILVLLITGISTMLQMKDHPWMLYLKNALALSRQHMNARY  >ApisGr73  MSTTFKQTIKFFIIIWKIIGLLHFCCTMETESGLIHRDMSSTFNLFLEMIRIFVYLICSYHMVYNMGENYLIINFNILKYWCIIIPARMSETRIIKLINGIIEFDRKLFLFNSLLVVELPTLSKTFWIAFVAIFSFYFIGCQLYLILNGIILNLENVHFASLLFHSPDIIIFMVMATSYFHLINFGRRFCTLNRLWKLLPSGIVALPGGWSSSELTVVMECIRLLHAELSELLRLFSLGYGPVILIYFTFTFIHALVDIFLIIVFDNSSVKLGILSFVFYIQYIMSTLSILCITSWVIKKKKKIISYLRLTRISELPTETKLQVKIFMSQISVYEPNELTAFGFFNLNLNLFMSILVLMITGIATLIQMKEYSFMIRLNNIMTPQFPKLE  >ApisGr74  MTSVFKETLNLFISASKLVGLMNYCCTMESGLFYRNTKLTYYLFLELIRMFVYLICSYRIISNLRNVNILIPFNVIKYWAIVITARISEKWMIKFINGVIEFDFKLFLFNSSFPVELYSISKKIWNIIFALFVLFFICTQYYFILLHHIEIKELSQFATTLFHTPDIIIFIVLVTSLYYLSNLGYRFCELNRLWKCLPFGLTALPGGLSHSEITILVERIRLLHAELSELLRLFSLGYGPVLLVYFTFTFSNALLETFLFTIYNSSKEYSILPYIFYLQHIFNMISIIYVTSWVIEKKKKIISYLRLIRISELTVHAKLQIKMFMQQISGYEPNEITAFGFFNFDLKLIMSILVLLITGISTMLQMKDHPMMLYLKNALKISNDHVHRIT  >ApisGr75P  MTCVFIETLNLFITASKIVGLVSFCCTMESGLFYRNTKSPYYIFLECIRMFVYLICSYHLIFNMGSAYIILHFNVLKYWSIVITARISEKWIIKFINGIIEFDNKLFLFNSSIPVKLYSISKIFWYIIFAIFVSFYLYCLYFFIGPGGLLKVKELSHYLIVVFFSPEIIIFVVLITSLYYLSNLGYRFSELNRLWKCLPFGLLAFPGGWTNTEITMLVESIRLLHAELSELLRLFSLGYGPVLLVYFTFTFAHALIETFLITIYKDSLNNILPYIFYLQYIFNMISIIYVTSWVIEKKKKIISCFLZLCRISEMTVHTKLQIKIFMNZISMYEPNEITAFGFFNFDLKLTMSILVLLITAISTMMQMKNHPWMLYLKNTFKANNDFIEDDI  >ApisGr76C  MTSVFMETLNLFITASKIVGLMNFCCTMESGLLCRSTKSPYYIFLECIRMFVYLICSYHLIFNMGSVYILFHFNVIKYWAIVITARISEKWIIKFINGIIEFDRKLFLFNSSFPVKLYSISKIFWNILFAIFFLFFLYTLYFYIGPNGFKKVKDISHYLAIVFYSPEIIVFVVLITSLYYLSNLGYRFCELNRLWNCLPFGLLALPGGWTNSEITMLVERIRLLHAELSELLRLFSLGYGPVLLVYFTFTFAHALLETFLITIYKDSLKNIFPYIFYLNHIFNMISIIYVTSWVIEKKRKIISCLRLCRISEMTVHTKLQILVLLITAISTMLQMKDHPWMLYLKNTFIASFEHMEANF  >ApisGr77I  MTSVFMETLNLFITASKVVGLMNFCCTMESGLLCRSTKSPYYIFLECIRMFVYLICTYHIVFNMGIVYILIHFNVLKYWSIVITARISEKLIIKCLYFFIGPNGLVTVKDLSHYVTVLFYSPEIIIFVVLITSLYYLSNLGYRFCELNRIWKCLPFGLLALPGGWTNSEITMLVERIRLLHAELSELLRLFSLGYGPVLLVYFTFTFAHALIETFLITMKKDSLNNNFFPYIFYLQHIFNMISILYVTSWVIEKKKKIISCLRLCRISEMAVHTKLQIKIFMNQISMYEPNEITAFGFFNFDLKLTMSILVLLITAISTMLQMKDHPWMLYLKNAWIANCEFMKANT  >DmelGr2a  MEFGMDTLRALEPLHRACQVCNLWPWRLAPPPDSEGILLRRSRWLELYGWTVLIAATSFTVYGLFQESSVEEKQDSESTISSIGHTVDFIQLVGMRVAHLAALLEALWQRQAQRGFFAELGEIDRLLSKALRVDVEAMRINMRRQTSRRAVWILWGYAVSQLLILGAKLLSRGDRFPIYWISYLLPLLVCGLRYFQIFNATQLVRQRLDVLLVALQQLQLHQKGPAVDTVLEEQEDLEEAAMDRLIAVRLVYQRVWALVALLNRCYGLSMLMQVGNDFLAITSNCYWMFLNFRQSAASPFDILQIVASGVWSAPHLGNVLVLSLLCDRTAQCASRLALCLHQVSVDLRNESHNALITQFSLQLLHQRLHFSAAGFFNVDCTLLYTIVGATTTYLIILIQFHMSESTIGSDSNGQ  >DmelGr5a  MRQLKGRNRCNRAVRHLKVQGKMWLKNLKSGLEQIRESQVRGTRKNFLHDGSFHEAVAPVLAVAQCFCLMPVCGISAPTYRGLSFNRRSWRFWYSSLYLCSTSVDLAFSIRRVAHSVLDVRSVEPIVFHVSILIASWQFLNLAQLWPGLMRHWAAVERRLPGYTCCLQRARPARRLKLVAFVLLVVSLMEHLLSIISVVYYDFCPRRSDPVESYLLGASAQLFEVFPYSNWLAWLGKIQNVLLTFGWSYMDIFLMMLGMGLSEMLARLNRSLEQQVRQPMPEAYWTWSRTLYRSIVELIREVDDAVSGIMLISFGSNLYFICLQLLKSINTMPSSAHAVYFYFSLLFLLSRSTAVLLFVSAINDQAREPLRLLRLVPLKGYHPEVFRFAAELASDQVALTGLKFFNVTRKLFLAMAGTVATYELVLIQFHEDKKTWDCSPFNLD  >DmelGr8a  MSGHLGRVLQFHLRLYQVLGFHGLPLPGDGNPARTRRRLMAWSLFLLISLSALVLACLFSGEEFLYRGDMFGCANDALKYVFAELGVLAIYLETLSSQRHLANFWWLHFKLGGQKTGLVSLRSEFQQFCRYLIFLYAMMAAEVAIHLGLWQFQALTQHMLLFWSTYEPLVWLTYLRNLQFVLHLELLREQLTGLEREMGLLAEYSRFASETGRSFPGFESFLRRRLVQKQRIYSHVYDMLKCFQGAFNFSILAVLLTINIRIAVDCYFMYYSIYNNVINNDYYLIVPALLEIPAFIYASQSCMVVVPRIAHQLHNIVTDSGCCSCPDLSLQIQNFSLQLLHQPIRIDCLGLTILDCSLLTRMACSVGTYMIYSIQFIPKFSNTYM  >DmelGr9a  MSLWLEHFLTGYFQLCGLVCGWSGSRLGRLLSSTFLVLILIELVGEIETYFTEENPDNESVPAYFAKVIMGVNMAYKMIHAWIALSALFECRRFRYLLEELPPVKATSFIYRHLILEIILFACNAFLVLSEYTIRGIYLENLRYAYSLQAVRARYLQMMVLVDRLDGKLEQLHHRVISGSSDYKTLRLDYAHLAKVTRSLSHLFGLSLLLLNVLCLGDWIIVCNVYFMVAYLQVLPATLFLFGQVMFVVCPTLIKIWSICAASHRCVSKSKHLQQQLKDLPGQTPVERSQIEGFALQIMQDPIQIDVCGIYHLNLQTLAGMFFFILEALVIFLQFVSLVRT  >DmelGr10a  MTSPDERKSFWERHEFKFYRYGHVYALIYGQVVIDYVPQRALKRGVKVLLIAYGHLFSMLLIVVLPGYFCYHFRTLTDTLDRRLQLLFYVSFTNTAIKYATVIVTYVANTVHFEAINQRCTMQRTHLEFEFKNAPQEPKRPFEFFMYFKFCLINLMMMIQVCGIFAQYGEVGKGSVSQVRVHFAIYAFVLWNYTENMADYCYFINGSVLKYYRQFNLQLGSLRDEMDGLRPGGMLLHHCCELSDRLEELRRRCREIHDLQRESFRMHQFQLIGLMLSTLINNLTNFYTLFHMLAKQSLEEVSYPVVVGSVYATGFYIDTYIVALINEHIKLELEAVALTMRRFAEPREMDERLTREIEHLSLELLNYQPPMLCGLLHLDRRLVYLIAVTAFSYFITLVQFDLYLRKKS  >DmelGr10b  MRVGKLCRLALRFWMGLILVLGFSSHYYNPTRRRLVYSRILQTYDWLLMVINLGAFYLYYRYAMTYFLEGMFRRQGFVNQVSTCNVFQQLLMAVTGTWLHFLFERHVCQTYNELSRILKHDLKLKEHSRFYCLAFLAKVYNFFHNFNFALSAIMHWGLRPFNVWDLLANLYFVYNSLARDAILVAYVLLLLNLSEALRLNGQQEHDTYSDLMKQLRRRERLLRIGRRVHRMFAWLVAIALIYLVFFNTATIYLGYTMFIQKHDALGLRGRGLKMLLTVVSFLVILWDVVLLQVICEKLLAEENKICDCPEDVASSRTTYRQWEMSALRRAITRSSPENNVLGMFRMDMRCAFALISCSLSYGIIIIQIGYIPG  >DmelGr21a  MSFWAVSRGLTPPSKVVPMLNPNQRQFLEDEVRYREKLKLMARGDAMEEVYVRKQETVDDPLELDKHDSFYQTTKSLLVLFQIMGVMPIHRNPPEKNLPRTGYSWGSKQVMWAIFIYSCQTTIVVLVLRERVKKFVTSPDKRFDEAIYNVIFISLLFTNFLLPVASWRHGPQVAIFKNMWTNYQYKFFKTTGSPIVFPNLYPLTWSLCVFSWLLSIAINLSQYFLQPDFRLWYTFAYYPIIAMLNCFCSLWYINCNAFGTASRALSDALQTTIRGEKPAQKLTEYRHLWVDLSHMMQQLGRAYSNMYGMYCLVIFFTTIIATYGSISEIIDHGATYKEVGLFVIVFYCMGLLYIICNEAHYASRKVGLDFQTKLLNINLTAVDAATQKEVEMLLVAINKNPPIMNLDGYANINRELITTNISFMATYLVVLLQFKITEQRRIGQQQA  >DmelGr22a  MSQPKRIHRICKGLARFTIRATLYGSWVLGLFPFTFDSRKRRLNRSKWLLAYGLVLNLTLLVLSMLPSTDDHNSVKVEVFQRNPLVKQVEELVEVISLITTLVTHLRTFSRSSELVEILNELLVLDKNHFSKLMLSECHTFNRYVIEKGLVIILEIGSSLVLYFGIPNSKIVVYEAVCIYIVQLEVLMVVMHFHLAVIYIYRYLWIINGQLLDMASRLRRGDSVDPDRIQLLLWLYSRLLDLNHRLTAIYDIQVTLFMATLFSVNIIVGHVLVICWINITRFSLLVIFLLFPQALIINFWDLWQGIAFCDLAESTGKKTSMILKLFNDMENMDQETERRVAEFTLFCSHRRLKVCHLGLLDINYEMGFRMIITNILYVVFLVQFDYMNLKFKTD  >DmelGr22b  MFGSSREIRPYLARQMLKTTLYGSWLLGIFPFTLDSGKRIRQLRRSRCLTLYGLVLNYFLIFTLIRLAFEYRKHKLEAFKRNPVLEMINVVIGIINVLSALIVHFMNFWGSRKVGEICNELLILEYQDFEGLNGRNCPNFNCFVIQKCLTILGQLLSFFTLNFALPGLEFHICLVLLSCLMEFSLNLNIMHYHVGVLLIYRYVWLINEQLKDLVSQLKLNPETDFSRIHQFLSLYKRLLELNRKLVIAYEYQMTLFIIAQLSGNIVVIYFLIVYGLSMRTYSIFLVAFPNSLLINIWDFWLCIAACDLTEKAGDETAIILKIFSDLEHRDDKLEMSVNEFAWLCSHRKFRFQLCGLFSMNCRMGFKMIITTFLYLVYLVQFDYMNL  >DmelGr22c  MFASRSDLQSRLCWIILKATLYSSWFLGVFPYRFDSRNGQLKRSRFLLFYGLILNFFLLLKMVCSGGQKLGIPEAFARNSVLENTHYTTGMLAVFSCVVIHFLNFWGSTRVQDLANELLVLEYQQFASLNETKCPKFNSFVIQKWLSVIGLLLSYLSIAYGLPGNNFSVEMVLINSLVQFSFNCNIMHYYIGVLLIYRYLWLINGQLLEMVTNLKLDCSVDSSRIRKYLSLYRRLLELKGYMVATYEYHMTLVLTTGLASNFLAIYSWIVLDISMNINFIYLLIFPLFLLVNVWNLWLSIAASDLAENAGKSTQTVLKLFADLEVKDIELERSVNEFALLCGHCQFNFHVCGLFTINYKMGFQMIITSFLYLIYMIQFDFMNL  >DmelGr22d  MFRPRCGLRQKFVYVILKSILYSSWLLGIFPFKYEPKKRRLRRSMWLIPFGVVISSSLLILMVKQSAEDREHGIMLDVFQRNALLYQISSLMGVVGVVSICTVHLRTLWRSKHLEEIYNGLMLLEAKYFCSNAVECPAFDGYVIQKGVVIVVGLLAPWMVHFGMPDSKLPVLNVLVVSMVKLGTLLLALHYHLGVVIIYRFVWLINRELLSLVCSLRGNHKGSSSRVRFLLKLYNKLVNLYSKLADCYDCQTVLMMAIFLAANIIVCFYMIVYRISLSKMSFFVMLIMFPLAIANNFMDFWLSMKVCDLLQKTGRQTSMILKLFNDIENMDKDLEISISDFALYCSHRRFKFLHCGLFHVNREMGFKMFVASVLYLLYLVQFDYMNL  >DmelGr22e  MFRPSGSGYRQKWTGLTLKGALYGSWILGVFPFAYDSWTRTLRRSKWLIAYGFVLNAAFILLVVTNDTESETPLRMEVFHRNALAEQINGIHDIQSLSMVSIMLLRSFWKSGDIERTLNELEDLQHRYFRNYSLEECISFDRFVLYKGFSVVLELVSMLVLELGMSPNYSAQFFIGLGSLCLMLLAVLLGASHFHLAVVFVYRYVWIVNRELLKLVNKMAIGETVESERMDLLLYLYHRLLDLGQRLASIYDYQMVMVMVSFLIANVLGIYFFIIYSISLNKSLDFKILVFVQALVINMLDFWLNVEICELAERTGRQTSTILKLFNDIENIDEKLERSITDFALFCSHRRLRFHHCGLFYVNYEMGFRMAITSFLYLLFLIQFDYWNL  >DmelGr22f  MKMFQPRRGFSCHLAWFMLQTTLYASWLLGLFPFTFDSRRKQLKRSRWLLLYGFVLHSLAMCLAMSSHLASKQRRKYNAFERNPLLEKIYMQFQVTTFFTISVLLLMNVWKSNTVRKIANELLTLEGQVKDLLTLKNCPNFNCFVIKKHVAAIGQFVISIYFCLCQENSYPKILKILCCLPSVGLQLIIMHFHTEIILVYRYVWLVNETLEDSHHLSSSRIHALASLYDRLLKLSELVVACNDLQLILMLIIYLIGNTVQIFFLIVLGVSMNKRYIYLVASPQLIINFWDFWLNIVVCDLAGKCGDQTSKVLKLFTDLEHDDEELERSLNEFAWLCTHRKFRFQLCGLFSINHNMGFQMIITSFLYLVYLLQFDFMNL  >DmelGr23aA  MKTLECLTRRFLEVIFSVLALVPLPPISQLGWLFLSLAIRCCWIVYFIYLLDVAISFSWVAIENVGNAVGTMLFVGNSVLGFALLLESVLKQKTHSQLEDLRVQTELQLQRLGMFGRSRHAAYLLPLIGVQFTCDLVRLATNFGETVSPVFCISLPLMWLLRYRYVQLVQHVMDLNQRSIHLRRSLLSMASGNDLWQPYGVQECLQLQTLRTTYERIFECYETFSDCYGWGMLGLHLLTSFQFVTNAYWMIMGIYDGGNVRSLIFNGATGIDFGTPIATLFWHGDSGAENGRQIGCLISKLVKPQGSKLYNDLVSEFSLQTLHQRFVVTAKDFFSLNLHLLSSMFAAVVTYLVILIQFMFAERSSTRGSG  >DmelGr23aB  MFPPTRVQASSRVVLKIFHFILVAFSLRSRRLSRLVLWLQFLGWLTWFISMWTQSVIYAQTIDCTLDCSLRHILTFFQTVSHAFIVVTSFLDGFRIKQDQLDEPIAFEDSDPWLAFTVLAMLVPTLGVEYLVCSNAPEYAFRIRIYHLKTLPSFLALQVQIISFILEVMKVNIRVRQTKLQLLILARELSCRWPQRKQKPQFSDQQAHRVKDLKRRYNDLHYLFVRINGYFGGSLLTIIIVHFAIFVSNSYWLFVDIRTRPWRIYAILLNLGFIFNVALQMAAACWHCQQSYNLGRQIGCLISKLVKPQGSKLYNDLVSEFSLQTLHQRFVVTAKDFFSLNLHLLSSMFAAVVTYLVILIQFMFAERSSTRGSG  >DmelGr28a  MAFKLWERFSQADNVFQALRPLTFISLLGLAPFRLNLNPRKEVQTSKFSFFAGIVHFLFFVLCFGISVKEGDSIIGYFFQTNITRFSDGTLRLTGILAMSTIFGFAMFKRQRLVSIIQNNIVVDEIFVRLGMKLDYRRILLSSFLISLGMLLFNVIYLCVSYSLLVSATISPSFVTFTTFALPHINISLMVFKFLCTTDLARSRFSMLNEILQDILDAHIEQLSALELSPMHSVVNHRRYSHRLRNLISTPMKRYSVTSVIRLNPEYAIKQVSNIHNLLCDICQTIEEYFTYPLLGIIAISFLFILFDDFYILEAILNPKRLDVFEADEFFAFFLMQLIWYIVIIVLIVEGSSRTILHSSYTAAIVHKILNITDDPELRDRLFRLSLQLSHRKVLFTAAGLFRLDRTLIFTITGAATCYLIILIQFRFTHHMDDTSSNSTNNLHSIHLGD  >DmelGr28bA  MIRCGLDIFRGCRGRFRYWLSARDCYDSISLMVAIAFALGITPFLVRRNALGENSLEQSWYGFLNAIFRWLLLAYCYSYINLRNESLIGYFMRNHVSQISTRVHDVGGIIAAVFTFILPLLLRKYFLKSVKNMVQVDTQLERLRSPVNFNTVVGQVVLVILAVVLLDTVLLTTGLVCLAKMEVYASWQLTFIFVYELLAISITICMFCLMTRTVQRRITCLHKVLKNLAHQWDTRSLKAVNQKQRSLQCLDSFSMYTIVTKDPAEIIQESMEIHHLICEAAATANKYFTYQLLTIISIAFLIIVFDAYYVLETLLGKSKRESKFKTVEFVTFFSCQMILYLIAIISIVEGSNRAIKKSEKTGGIVHSLLNKTKSAEVKEKLQQFSMQLMHLKINFTAAGLFNIDRTLYFTISGALTTYLIILLQFTSNSPNNGYGNGSSCCETFNNMTNHTL  >DmelGr28bB  MSALRRVRKYFISSQVYEALRPLFFLTFLYGLTPFHVVRRKMGESYLKMSCFGVFNIFIYICLCGFCYISSLRQGESIVGYFFRTEISTIGDRLQIFNGLIAGAVIYTSAILKRCKLLGTLTILHSLDTNFSNIGVRVKYSRIFRYSLLVLIFKLLILGVYFVGVFRLLVSLDVTPSFCVCMTFFLQHSVVSIAICLFCVIAFSFERRLSIINQVLKNLAHQWDTRSLKAVNQKQRSLQCLDSFSMYTIVTKDPAEIIQESMEIHHLICEAAATANKYFTYQLLTIISIAFLIIVFDAYYVLETLLGKSKRESKFK  TVEFVTFFSCQMILYLIAIISIVEGSNRAIKKSEKTGGIVHSLLNKTKSAEVKEKLQQFSMQLMHLKINFTAAGLFNIDRTLYFTISGALTTYLIILLQFTSNSPNNGYGNGSSCCETFNNMTNHTL  >DmelGr28bC  MDIEMAKEPVNPTDTPDIEVTPGLCQPLRRRFRRFVTAKQLYECLRPVFHVTYIHGLTSFYISCDTKTGKKAIKKTIFGYINGIMHIAMFVFAYSLTIYNNCESVASYFFRSRITYFGDLMQIVSGFIGVTVIYLTAFVPNHRLERCLQKFHTMDVQLQTVGVKIMYSKVLRFSYMVLISMFLVNVLFTGGTFSVLYSSEVAPTMALHFTFLIQHTVIAIAIALFSCFTYLVEMRLVMVNKVLKNLAHQWDTRSLKAVNQKQRSLQCLDSFSMYTIVTKDPAEIIQESMEIHHLICEAAATANKYFTYQLLTIISIAFLIIVFDAYYVLETLLGKSKRESKFKTVEFVTFFSCQMILYLIAIISIVEGSNRAIKKSEKTGGIVHSLLNKTKSAEVKEKLQQFSMQLMHLKINFTAAGLFNIDRTLYFTISGALTTYLIILLQFTSNSPNNGYGNGSSCCETFNNMTNHTL  >DmelGr28bD  MSFYFCEIFKPRDAFGAEQTLLLYTYLLGLTPFRLRGQAGERQFHLSKIGYLNAFLQLSFFSYCFLAALIEQQSIVGYFFKSEISQMGDSLQKFIGMTGMSILFLCSSIRVRLLIHIWDRISYIDDRFLNLGVCFNYPAIMRLRLLQIFLINGVQLGYLISSNWMLLGNDVRPIYTAIVAFYVPQIFLLSIVMLFNATLHRLWQHFTVLNQVLKNLAHQWDTRSLKAVNQKQRSLQCLDSFSMYTIVTKDPAEIIQESMEIHHLICEAAATANKYFTYQLLTIISIAFLIIVFDAYYVLETLLGKSKRESKFKTVEFVTFFSCQMILYLIAIISIVEGSNRAIKKSEKTGGIVHSLLNKTKSAEVKEKLQQFSMQLMHLKINFTAAGLFNIDRTLYFTISGALTTYLIILLQFTSNSPNNGYGNGSSCCETFNNMTNHTL  >DmelGr28bE  MWLLRRSVGKSGNRPHDVYTCYRLTIFMALCLGIVPYYVSISSEGRGKLTSSYIGYINIIIRMAIYMVNSFYGAVNRDTLMSNFFLTDISNVIDALQKINGMLGIFAILLISLLNRKELLKLLATFDRLETEAFPRVGVAMHQVAANKKMNRLVIILVGSMVAYITCSFLMISLRDTTTFSISAVISFFSPHFIVCAVSFLAGNVMIKLRIYLSALNEVLKNLAHQWDTRSLKAVNQKQRSLQCLDSFSMYTIVTKDPAEIIQESMEIHHLICEAAATANKYFTYQLLTIISIAFLIIVFDAYYVLETLLGKSKRESKFKTVEFVTFFSCQMILYLIAIISIVEGSNRAIKKSEKTGGIVHSLLNKTKSAEVKEKLQQFSMQLMHLKINFTAAGLFNIDRTLYFTISGALTTYLIILLQFTSNSPNNGYGNGSSCCETFNNMTNHTL  >DmelGr32a  MSPNTWVIEMPTQKTRSHPYPRRISPYRPPVLNRDAFSRDAPPMPARNHDHPVFEDIRTILSVLKASGLMPIYEQVSDYEVGPPTKTNEFYSFFVRGVVHALTIFNVYSLFTPISAQLFFSYRETDNVNQWIELLLCILTYTLTVFVCAHNTTSMLRIMNEILQLDEEVRRQFGANLSQNFGFLVKFLVGITACQAYIIVLKIYAVQGEITPTSYILLAFYGIQNGLTATYIVFASALLRIVYIRFHFINQLLNGYTYGQQHRRKEGGARARRQRGDVNPNVNPALMEHFPEDSLFIYRMHNKLLRIYKGINDCCNLILVSFLGYSFYTVTTNCYNLFVQITGKGMVSPNILQWCFAWLCLHVSLLALLSRSCGLTTTEANATSQILARVYAKSKEYQNIIDKFLTKSIKQEVQFTAYGFFAIDNSTLFKIFSAVTTYLVILIQFKQLEDSKVEDPVPEQT  >DmelGr33a  MIQIMNWFSMVIGLIPLNRQQSETNFILDYAMMCIVPIFYVACYLLINLSHIIGLCLLDSCNSVCKLSSHLFMHLGAFLYLTITLLSLYRRKEFFQQFDARLNDIDAVIQKCQRVAEMDKVKVTAVKHSVAYHFTWLFLFCVFTFALYYDVRSLYLTFGNLAFIPFMVSSFPYLAGSIIQGEFIYHVSVISQRFEQINMLLEKINQEARHRHAPLTVFDIESEGKKERKTVTPITVMDGRTTTGFGNENKFAGEMKRQEGQQKNDDDDLDTSNDEDEDDFDYDNATIAENTGNTSEANLPDLFKLHDKILALSVITNGEFGPQCVPYMAACFVVSIFGIFLETKVNFIVGGKSRLLDYMTYLYVIWSFTTMMVAYIVLRLCCNANNHSKQSAMIVHEIMQKKPAFMLSNDLFYNKMKSFTLQFLHWEGFFQFNGVGLFALDYTFIFSTVSAATSYLIVLLQFDMTAILRNEGLM  S  >DmelGr36a  MFDWVGLLLKVLYYYGQIIGLINFEIDWQRGRVVAAQRGILFAIAINVLICMVLLLQISKKFNLDVYFGRANQLHQYVIIVMVSLRMASGISAILNRWRQRAQLMRLVECVLRLFLKKPHVKQMSRWAILVKFSVGVVSNFLQMAISMESLDRLGFNEFVGMASDFWMSAIINMAISQHYLVILFVRAYYHLLKTEVRQAIHESQMLSEIYPRRAAFMTKCCYLADRIDNIAKLQNQLQSIVTQLNQVFGIQGIMVYGGYYIFSVATTYITYSLAINGIEELHLSVRAAALVFSWFLFYYTSAILNLFVMLKLFDDHKEMERILEERTLFTSALDVRLEQSFESIQLQLIRNPLKIEVLDIFTITRSSSAAMIGSIITNSIFLIQYDMEYF  >DmelGr36b  MVDWVVLLLKAVHIYCYLIGLSNFEFDCRTGRVFKSRRCTIYAFMANIFILITIIYNFTAHGDTNLLFQSANKLHEYVIIIMSGLKIVAGLITVLNRWLQRGQMMQLVKDVIRLYMINPQLKSMIRWGILLKAFISFAIELLQVTLSVDALDRQGTAEMMGLLVKLCVSFIMNLAISQHFLVILLIRAQYRIMNAKLRMVIEESRRLSFLQLRNGAFMTRCCYLSDQLEDIGEVQSQLQSMVGQLDEVFGMQGLMAYSEYYLSIVGTSYMSYSIYKYGPHNLKLSAKTSIIVCILITLFYLDALVNCNNMLRVLDHHKDFLGLLEERTVFASSLDIRLEESFESLQLQLARNPLKINVMGMFPITRGSTAAMCASVIVNSIFLIQFDMEFF  >DmelGr36c  MDLESFLLGAVYYYGLFIGLSNFEFDWNTGRVFTKKWSTLYAIALDSCIFALYIYHWTGNTNIVNAIFGRANMLHEYVVAILTGLRIVTGLFTLILRWYQRCKMMDLASKVVRMYVARPQVRRMSRWGILTKFIFGSITDGLQMAMVLSAMGSVDSQFYLGLGLQYWMFVILNMAMMQQHMIMLFVRTQFQLINTELRQVIDEAKDLLLSPRHQGVFMTKCCSLADQIENIARIQSQLQTIMNQMEEVFGIQGAMTYGGYYLSSVGTCYLAYSILKHGYENLSMTLSTVILAYSWCFFYYLDGMLNLSVMLHVQDDYWEMLQILGKRTIFVGLDVRLEEAFENLNLQLIRNPLKITVVKLYDVTRSNTMAMFGNLITHSIFLIQYDIEHF  >DmelGr39aA  MGTRNRKLLFFLHYQRYLGLTNLDFSKSLHIYWLHGTWSSTAIQIVVVGVFMAALLGALAESLYYMETKSQTGNTFDNAVILTTSVTQLLANLWLRSQQKSQVNLLQRLSQVVELLQFEPYAVPQFRWLYRIWLLVCLIYGAMVTHFGINWLTTMQISRVLTLIGFVYRCVLANFQFTCYTGMVVILKKLLQVQVKQLEHLVSTTTISMAGVAGCLRTHDEILLLGQRELIAVYGGVILFLFIYQVMQCILIFYISNLEGFHSSNDLVLIFCWLAPMLFYLILPLVVNDIHNQANKTAKMLTKVPRTGTGLDRMIEKFLLKNLRQKPILTAYGFFALDKSTLFKLFTAIFTYMVILVQFKEMENSTKSINKF  >DmelGr39aB  MDFQPGELCAYYRLCRYLGIFCIDYNPTKKKFRLRRSVLCYIVHFALQAYLVGCISVMVTYWRRCFKSELTTTGNHFDRLVMVIALGILVVQNAWLIWLQAPHLRIVRQIEFYRRNHLANVRLLLPKRLLWLIIATNVVYMANFIKTCIFEWLTDASRLFVITSLGFPLRYLVTSFTMGTYFCMVHIVRLVLDWNQSQINAIIDESADLKMTSPNRLRLRVCLEMHDRLMLLCNDEISLVYGFIAWLSWMFASLDVTGVIYLTMVIQTKKSIVLKLITNVVWLSPTFMTCAASFMSNRVTIQANKTAKMLTKVPRTGTGLDRMIEKFLLKNLRQKPILTAYGFFALDKSTLFKLFTAIFTYMVILVQFKEMENSTKSINKF  >DmelGr39aC  MKRNAFEELRVQLRTLKWLGVLRFTIDFNKCLVRENASEERSAWLYLIGVVGITCSLIVYSTYFPSHFIMGKHNTTGNCYALINIRSCSIVTMLIYTQLYIQRFRFVALLQSILRFNQISGSHREEGRFAFYYYTHLSLLIICMLNYAYGYWTAGVRLTTIPIYLLQYGFSYLFLGQVVVLFACIQQILLSILKYYNQVVLKNIKSSKESREFYYNFCKYNQVIWLSYTEINHCFGLLLLLVTGLILLITPSGPFYLVSTIFEGRFRQNWQFSLMSFTAILWSLPWIVLLVLAMGRNDVQKEANKTAKMLTKVPRTGTGLDRMIEKFLLKNLRQKPILTAYGFFALDKSTLFKLFTAIFTYMVILVQFKEMENSTKSINKF  >DmelGr39aD  MSKVCRDLRIYLRLLHIMGMMCWHFDSDHCQLVATSGSERYAVVYAGCILVSTTAGFIFALLHPSRFHIAIYNQTGNFYEAVIFRSTCVVLFLVYVILYAWRHRYRDLVQHILRLNRRCASSCTNQQFLHNIILYGMLTILCFGNYLHGYTRAGLATLPLALCMLVYIFAFLVLCLLLMFFVSLKQVMTAGLIHYNQQLCQGDLISGLRGRQQILKLCGGELNECFGLLMLPIVALVLLMAPSGPFFLISTVLEGKFRPDECLIMLLTSSTWDTPWMIMLVLMLRTNGISEEANKTAKMLTKVPRTGTGLDRMIEKFLLKNLRQKPILTAYGFFALDKSTLFKLFTAIFTYMVILVQFKEMENSTKSINKF  >DmelGr39b  MLYSFHPYLKYFALLGLVPWSESCAQSKFVQKVYSAILIILNAVHFGISIYFPQSAELFLSLMVNVIVFVARIVCVTVIILQVMVHYDDYFRFCREMKYLGLRLQCELKIHVGRLKWQSYAKILALGIGFLVTVLPSIYVALSGSLLYFWSSLLSILIIRMQFVLVLLNVELLGHHVSLLGIRLQNVLECHLMGANCTLDGNANRLCSLEFLLALKQSHMQLHYLFTHFNDLFGWSILGTYVVLFSDSTVNIYWTQQVLVEVYEYKYLYATFSVFVPSFFNILVFCRCGEFCQRQSVLIGSYLRNLSCHPSIGRETSYKDLLMEFILQVEQNVLAINAEGFMSTDNSLLMSILAAKVTYLIVLMQFSSV  >DmelGr43a  MEISQPSIGIFYISKVLALAPYATVRNSKGRVEIGRSWLFTVYSATLTVVMVFLTYRGLLFDANSEIPVRMKSATSKVVTALDVSVVVMAIVSGVYCGLFSLNDTLELNDRLNKIDNTLNAYNNFRRDRWRALGMAAVSLLAISILVGLDVGTWMRIAQDMNIAQSDTELNVHWYIPFYSLYFILTGLQVNIANTAYGLGRRFGRLNRMLSSSFLAENNATSAIKPQKVSTVKNVSVNRPAMPSALHASLTKLNGETLPSEAAAKNKGLLLKSLADSHESLGKCVHLLSNSFGIAVLFILVSCLLHLVATAYFLFLELLSKRDNGYLWVQMLWICFHFLRLLMVVEPCHLAARESRKTIQIVCEIERKVHEPILAEAVKKFWQQLLVVDADFSACGLCRVNRTILTSFASAIATYLVILIQFQRTNG  >DmelGr47a  MAFTSSQLCSLLTKFTALNGLNTYYFDTKTNAFRVSSKLKIYCAIHHALCVLALAHMSYSTASNLRVSVTVLTIGGTMACCVKSCWEKAQGIRNLARGLVTMEQKYFAGRPSGLLLKCRYYIKITFGSITLLRIHLIQPIYMRRLLPSQFYLNVGAYWLLYNMLLAAVLGFYFLLWEMCRIQKLINDQMTLILARSGQRNRLKKMQHCLRLYSKLLLLCDQFNSQLGHVAIWVLACKSWCQITFGYEIFQMVAAPKSIDLTMSMRVFVIFTYIFDAMNLFLGTDISELFSTFRADSQRILRETSRLDRLLSMFALKLALHPKRVVLLNVFTFDRKLTLTLLAKSTLYTICCLQNDYNKLKA  >DmelGr47b  MQRDDGFVYCYGNLYSLLLYWGLVTIRVRSPDRGGAFSNRWTVCYALFTRSFMVICFMATVMTKLRDPEMSAAMFGHLSPLVKAIFTWECLSCSVTYIEYCLSLDLQKDRHLKLVARMQEFDRSVLMVFPHVQWNYRRARLKYWYGTVIVGFCFFSFSISLIFDTTRCTCGIPSTLLMAFTYTLLTSSVGLLGFVHIGIMDFIRVRLRLVQQLLHQLYQADDSSEVHERIAYLFEMSKRCSFLLAELNGVFGFAAAAGIFYDFTIMTCFVYVICQKLLEREPWDPEYVYMLLHVAIHTYKVVITSTYGYLLLREKRNCMHLLSQYSRYFSGQDVARRKTEDFQHWRMHNRQAAMVGSTTLLSVSTIYLVYNGMANYVIILVQLLFQQQQIKDHQLTSGKDVDIVGPMGPITHMD  >DmelGr57a  MAVLYFFREPETVFDCAAFICILQFLMGCNGFGIRRSTFRISWASRIYSMSVAIAAFCCLFGSLSVLLAEEDIRERLAKADNLVLSISALELLMSTLVFGVTVISLQVFARRHLGIYQRLAALDARLMSDFGANLNYRKMLRKNIAVLGIVTTIYLMAINSAAVQVASGHRALFLLFALCYTIVTGGPHFTGYVHMTLAEMLGIRFRLLQQLLQPEFLNWRFPQLHVQELRIRQVVSMIQELHYLIQEINRVYALSLWAAMAHDLAMSTSELYILFGQSVGIGQQNEEENGSCYRMLGYLALVMIPPLYKLLIAPFYCDRTIYEARRCLRLVEKLDDWFPQKSSLRPLVESLMSWRIQAKIQFTSGLDVVLSRKVIGLFTSILVNYLLILIQFAMTQKMGEQIEQQKIALQEWIGF  >DmelGr58a  MLLKFMYIYGIGCGLMPAPLKKGQFLLGYKQRWYLIYTACLHGGLLTVLPFTFPHYMYDDSYMSSNPVLKWTFNLTNITRIMAMFSGVLLMWFRRKRILNLGENLILHCLKCKTLDNRSKKYSKLRKRVRNVLFQMLLVANLSILLGALILFRIHSVQRISKTAMIVAHITQFIYVVFMMTGICVILLVLHWQSERLQIALKDLCSFLNHEERNSLTLSENKANRSLGKLAKLFKLFAENQRLVREVFRTFDLPIALLLLKMFVTNVNLVYHGVQFGNDTIETSSYTRIVGQWVVISHYWSAVLLMNVVDDVTRRSDLKMGDLLREFSHLELVKRDFHLQLELFSDHLRCHPSTYKVCGLFIFNKQTSLAYFFYVLVQVLVLVQFDLKNKVEKRN  >DmelGr58b  MLHPKLGRVMNVVYYHSVVFALMSTTLRIRSCRKCLRLEKVSRTYTIYSFFVGIFLFLNLYFMVPRIMEDGYMKYNIVLQWNFFVMLFLRAIAVVSCYGTLWLKRHKIIQLYKYSLIYWKRFGHITRAIVDKKELLDLQESLARIMIRKIILLYSAFLCSTVLQYQLLSVINPQIFLAFCARLTHFLHFLCVKMGFFGVLVLLNHQFLVIHLAINALHGRKARKKWKALRSVAAMHLKTLRLARRIFDMFDIANATVFINMFMTAINILYHAVQYSNSSIKSNGWGILFGNGLIVFNFWGTMALMEMLDSVVTSCNNTGQQLRQLSDLPKVGPKMQRELDVFTMQLRQNRLVYKICGIVELDKPACLSYIGSILSNVIILMQFDLRRQRQPINDRQYLIHLMKNKTKV  >DmelGr58c  MNQYFLLHTYFQVSRLIGLCNLHYDSSNHRFILNHVPTVVYCVILNVVYLLVLPFALFVLTGNIYHCPDAGMFGVVYNVVALTKLLTMLFLMSSVWIQRRRLYKLGNDLMKMLHKFRFNLGNDCRNRCLCKGLLTSSRFVLLTQQLLTRDSVVNCESNSSLRQAMVPYQSAAIVYALIMILLMSYVDMTVYMVEVAGNWLLVNMTQGVREMVQDLEVLPERNGIPREMGLMQILAAWRKLWRRCRRLDALLKQFVDIFQWQVLFNLLTTYIFSIAVLFRLWIYLEFDKNFHLWKGILYAIIFLTHHVEIVMQFSIFEINRCKWLGLLEDVGNLWDINYSGRQCIKSSGTILSRKLEFSLLYMNRKLQLNPKRVRRLHIVGLFDLSNLTVHNMTRSIITNVLVLCQIAYKKYG  >DmelGr59a  MKRIGQAYNVYAVFIGMTSYETMGGKFRQSRITRIYCLLINAIFLTLLPSAFWKSAKLLSTADWMPSYMRVTPYIMCTINYAAIAYTLISRCYRDAMLMDLQRIVLEVNREMLRTGKKMNSLLRRMFFLKTFTLTYSCLSYILAVFIYQWKAQNWSNLCNGLLVNISLTILFVNTFFYFTSLWHIARGYDFVNQQLNEIVACQSMDLERKSKELRGLWALHRNLSYTARRINKHYGPQMLAMRFDYFIFSIINACIGTIYSTTDQEPSLEKIFGSLIYWVRSFDFFLNDYICDLVSEYQMQPKFFAPESSMSNELSSYLIYESSTRLDLLVCGLYRVNKRKWLQMVGSIVVHSSMLFQFHLVMRGGL  >DmelGr59b  MVYWMIKLYFRYSLAIGITSQQFSNRKFFSTLFSRTYALIANIVTLIMLPIVMWQVQLVFQQKKTFPKLILITNNVREAVSFLVILYTVLSRGFRDTAFKEMQPLLLTLFREEKRCGFKGIGGVRRSLRILLFVKFFTLSWLCVTDVLFLLYSTDALIWVNVLRFFFKCNTNNILEMVPMGYFLALWHIARGFDCVNRRLDQIVKSKSTRKHRELQHLWLLHACLTKTALNINKIYAPQMLASRFDNFVNGVIQAYWGAVFTFDLSTPFFWVVYGSVQYHVRCLDYYLIDNMCDVAVEYHDSAKHSWSEVRWTKEISSYVIYANSTKLQLWSCGLFQANRSMWFAMISSVLYYILVLLQFHLVMRK  >DmelGr59c  MVDLVKTILLIAYWYGLAVGVSNFEVDWLTGEAIATRRTTIYAAVHNASLITLLILFNLGNNSLKSEFISARYLHEYFFMLMTAVRISAVLLSLITRWYQRSRFIRIWNQILALVRDRPQVVRGRWYRRSIILKFVFCVLSDSLHTISDVSAQRKRITADLIVKLSLLATLTTIFNMIVCQYYLAMVQVIGLYKILLQDLRCLVRQAECICSIRNRRGGVYSIQCCSLADQLDLIAERHYFLKDRLDEMSDLFQIQSLSMSLVYFFSTMGSIYFSVCSILYSSTGFGSTYWGLLLIVLSTASFYMDNWLSVNIGFHIRDQQDELFRVLADRTLFYRELDNRLEAAFENFQLQLASNRHEFYVMGLFKMERGRLIAMLSSVITHTMVLVQWEIQNDES  >DmelGr59d  MADLLKLCLRIAYAYGRLTGVINFKIDLKTGQALVTRGATLISVSTHLLIFALLLYQTMRKSVVNVMWKYANSLHEYVFLVIAGFRVVCVFLELVSRWSQRRTFVRLFNSFRRLYQRNPDIIQYCRRSIVSKFFCVTMTETLHIIVTLAMMRNRLSIALALRIWAVLSLTAIINVIITQYYVATACVRGRYALLNKDLQAIVTESQSLVPNGGGVFVTKCCYLADRLERIAKSQSDLQELVENLSTAYEGEVVCLVITYYLNMLGTSYLLFSISKYGNFGNNLLVIITLCGIVYFVFYVVDCWINAFNVFYLLDAHDKMVKLLNKRTLFQPGLDHRLEMVFENFALNLVRNPLKLHMYGLFEFGRGTSFAVFNSLLTHSLLLIQYDVQNF  >DmelGr59e  MDSSYWENLLLTINRFLGVYPSGRVGVLRWLHTLWSLFLLMYIWTGSIVKCLEFTVEIPTIEKLLYLMEFPGNMATIAILVYYAVLNRPLAHGAELQIERIITGLKGKAKRLVYKRHGQRTLHLMATTLVFHGLCVLVDVVNYDFEFWTTWSSNSVYNLPGLMMSLGVLQYAQPVHFLWLVMDQMRMCLKELKLLQRPPQGSTKLDACYESAFAVLVDAGGGSALMIEEMRYTCNLIEQVHSQFLLRFGLYLVLNLLNSLVSICVELYLIFNFFETPLWEESVLLVYRLLWLAMHGGRIWFILSVNEQILEQKCNLCQLLNELEVCSSRLQRTINRFLLQLQRSIDQPLEACGIVTLDTRSLGGFIGVLMAIVIFLIQIGLGNKSLMGVALNRSN  WVYV  >DmelGr59f  MRSSATKGAKLKNSPRERLSSFNPQYAERYKELYRTLFWLLLISVLANTAPITILPGCPNRFYRLVHLSWMILWYGLFVLGSYWEFVLVTTQRVSLDRYLNAIESAIYVVHIFSIMLLTWQCRNWAPKLMTNIVTSDLNRAYTIDCNRTKRFIRLQLFLVGIFACLAIFFNIWTHKFVVYRSILSINSYVMPNIISSISFAQYYLLLQGIAWRQRRLTEGLERELTHLHSPRISEVQKIRMHHANLIDFTKAVNRTFQYSILLLFVGCFLNFNLVLFLVYQGIENPSMADFTKWVCMLLWLAMHVGKVCSILHFNQSIQNEHSTCLTLLSRVSYARKDIQDTITHFIIQMRTNVRQHVVCGVINLDLKFLTTLLVASADFFIFLLQYDVTYEALSKSVQGNVTRYK  >DmelGr61a  MSRTSDDIRKHLKVRRQKQRAILAMRWRCAQGGLEFEQLDTFYGAIRPYLCVAQFFGIMPLSNIRSRDPQDVKFKVRSIGLAVTGLFLLLGGMKTLVGANILFTEGLNAKNIVGLVFLIVGMVNWLNFVGFARSWSHIMLPWSSVDILMLFPPYKRGKRSLRSKVNVLALSVVVLAVGDHMLYYASGYCSYSMHILQCHTNHSRITFGLYLEKEFSDIMFIMPFNIFSMCYGFWLNGAFTFLWNFMDIFIVMTSIGLAQRFQQFAARVGALEGRHVPEALWYDIRRDHIRLCELASLVEASMSNIVFVSCANNVYVICNQALAIFTKLRHPINYVYFWYSLIFLLARTSLVFMTASKIHDASLLPLRSLYLVPSDGWTQEVQRFADQLTSEFVGLSGYRLFCLTRKSLFGMLATLVTYELMLLQIDAKSHKGLRCA  >DmelGr63a  MRPSGEKVVKGHGQGNSGHSLSGMANYYRRKKGDAVFLNAKPLNSANAQAYLYGVRKYSIGLAERLDADYEAPPLDRKKSSDSTASNNPEFKPSVFYRNIDPINWFLRIIGVLPIVRHGPARAKFEMNSASFIYSVVFFVLLACYVGYVANNRIHIVRSLSGPFEEAVIAYLFLVNILPIMIIPILWYEARKIAKLFNDWDDFEVLYYQISGHSLPLKLRQKAVYIAIVLPILSVLSVVITHVTMSDLNINQVVPYCILDNLTAMLGAWWFLICEAMSITAHLLAERFQKALKHIGPAAMVADYRVLWLRLSKLTRDTGNALCYTFVFMSLYLFFIITLSIYGLMSQLSEGFGIKDIGLTITALWNIGLLFYICDEAHYASVNVRTNFQKKLLMVELNWMNSDAQTEINMFLRATEMNPSTINCGGFFDVNRTLFKGLLTTMVTYLVVLLQFQISIPTDKGDSEGANNITVVDFVMDSLDNDMSLMGASTLSTTTVGTTLPPPIMKLKGRKG  >DmelGr64a  MKGPNLNFRKTPSKDNGVKQVESLARPETPPPKFVEDSNLEFNVLASEKLPNYTNLDLFHRAVFPFMFLAQCVAIMPLVGIRESNPRRVRFAYKSIPMFVTLIFMIATSILFLSMFTHLLKIGITAKNFVGLVFFGCVLSAYVVFIRLAKKWPAVVRIWTRTEIPFTKPPYEIPKRNLSRRVQLAALAIIGLSLGEHALYQVSAILSYTRRIQMCANITTVPSFNNYMQTNYDYVFQLLPYSPIIAVLILLINGACTFVWNYMDLFIMMISKGLSYRFEQITTRIRKLEHEEVCESVFIQIREHYVKMCELLEFVDSAMSSLILLSCVNNLYFVCYQLLNVFNKLRWPINYIYFWYSLLYLIGRTAFVFLTAADINEESKRGLGVLRRVSSRSWCVEVERLIFQMTTQTVALSGKKFYFLTRRLLFGMAGTIVTYELVLLQFDEPNRRKGLQPLCA  >DmelGr64b  MPQGETFHRAVSNVLFISQIYGLLPVSNVRALDVADIRFRWCSPRILYSLLIGILNLSEFGAVINYVIKVTINFHTSSTLSLYIVCLLEHLFFWRLAIQWPRIMRTWHGVEQLFLRVPYRFYGEYRIKRRIYIVFTIVMSSALVEHCLLLGNSFHLSNMERTQCKINVTYFESIYKWERPHLYMILPYHFWMLPILEWVNQTIAYPRSFTDCFIMCIGIGLAARFHQLYRRIAAVHRKVMPAVFWTEVREHYLALKRLVHLLDAAIAPLVLLAFGNNMSFICFQLFNSFKNIGVDFLVMLAFWYSLGFAVVRTLLTIFVASSINDYERKIVTALRDVPSRAWSIEVQRFSEQLGNDTTALSGSGFFYLTRSLVLAMGTTIITYELMISDVINQGSIRQKTQYCREY  >DmelGr64c  MQQSGQKGTRNTLQHAIGPVLVIAQFFGVLPVAGVWPSCRPERVRFRWISLSLLAALILFVFSIVDCALSSKVVFDHGLKIYTIGSLSFSVICIFCFGVFLLLSRRWPYIIRRTAECEQIFLEPEYDCSYGRGYSSRLRLWGVCMLVAALCEHSTYVGSALYNNHLAIVECKLDANFWQNYFQRERQQLFLIMHFTAWWIPFIEWTTLSMTFVWNFVDIFLILICRGMQMRFQQMHWRIRQHVRQQMPNEFWQRIRCDLLDLSDLLGIYDKELSGLIVLSCAHNMYFVCVQIYHSFQSKGNYADELYFWFCLSYVIIRVLNMMFAASSIPQEAKEISYTLYEIPTEFWCVELRRLNEIFLSDHFALSGKGYFLLTRRLIFAMAATLMVYELVLINQMAGSEVQKSFCEGGVGSSKSIFS  >DmelGr64d  MLRSHLSVHGLQMERSVQENTLHYTIGHVLIIARIFGVLPLAGINPNGKPENVRFRWFSPYILFFVVAFTFVIADFMLSTKIVLNDGLQLYTMGSLSFSVICIFCFGSFIKLSRRWPHIIRETALCERIFLKPCYANQEGLNFTRFLRRWALILLVAALCEHLTYVGSAAWSNYVQIRDCNLKVGFVENYFLRERQELFSVFEYRAWMVFFIEWNTMAMTFVWNFGDIFLFLMCRGLKIRFQQLHWRIRQNLGKPMAKEFWQEIRSDFLDLDSLLKLYDKELSGLILVCCAHNMYFICVQVYHSFQVKGAFMDELYFWFCLLYVISRLMNMMLAASSIPQEIKDISNTLYEVRSSPWCDELGRLSEMLRNETFALSGMGYFYVTRRLIFAMAGALMGYELVLFRQMQGAVVQKSICSRGPGSSMSIFFS  >DmelGr64e  MARTTGDPAKRRRCMSRIKFWRRSRVGSEATLGIIKYRVVEKDTKRFKLSLIKAWLLRIRQEDYKYSGSFQEAIKPVLIIAQIFALMPVRKVSSKFAEDLTFTWFSVRSYYALVTILFFGVSSGYMVAFVTSVSFNFDSVETLVFYLSIFLISLSFFQLARKWPEIAQSWQLVEAKLPPLKLPKERRSLAQHINMITIVATTCSLVEHIMSMLSMGYYVNSCPRWPDRPIDSFLYLSFSSVFYFVDYTRFLGIVGKVVNVLSTFAWNFNDIFVMAVSVALAARFRQLNDYMMREARLPTTVDYWMQCRINFRNLCKLCEEVDDAISTITLLCFSNNLYFICGKILKSMQAKPSIWHALYFWFSLVYLLGRTLILSLYSSSINDESKRPLVIFRLVPREYWCDELKRFSEEVQMDNVALTGMKFFRLTRGVVISVAGTIVTYELILLQFNGEEKVPGCFEN  >DmelGr64f  MKILPKLERKLRRLKKRVTRTSLFRKLDLVHERARKKAFQESCETYKNQIENEYEIRNSLPKLSRSDKEAFLSDGSFHQAVGRVLLVAEFFAMMPVKGVTGKHPSDLSFSWRNIRTCFSLLFIASSLANFGLSLFKVLNNPISFNSIKPIIFRGSVLLVLIVALNLARQWPQLMMYWHTVEKDLPQYKTQLTKWKMGHTISMVMLLGMMLSFAEHILSMVSAINYASFCNRTADPIQNYFLRTNDEIFFVTSYSTTLALWGKFQNVFSTFIWNYMDLFVMIVSIGLASKFRQLNDDLRNFKGMNMAPSYWSERRIQYRNICILCDKMDDAISLITMVSFSNNLYFICVQLLRSLNTMPSVAHAVYFYFSLIFLIGRTLAVSLYSSSVHDESRLTLRYLRCVPKESWCPEVKRFTEEVISDEVALTGMKFFHLTRKLVLSVAGTIVTYELVLIQFHEDNDLWDCDQSYYS  >DmelGr66a  MDNMAQAEDAVQPLLQQFQQLFFISKIAGILPQDLEKFRSRNLLEKSRNGMIYMLSTLILYVVLYNILIYSFGEEDRSLKASQSTLTFVIGLFLTYIGLIMMVSDQLTALRNQGRIGELYERIRLVDERLYKEGCVMDNSTIGRRIRIMLIMTVIFELSILVSTYVKLVDYSQWMSLLWIVSAIPTFINTLDKIWFAVSLYALKERFEAINATLEELVDTHEKHKLWLRGNQEVPPPLDSSQPPQYDSNLEYLYKELGGMDIGSIGKSSVSGSGKNKVAPVAHSMNSFGEAIDAASRKPPPPPLATNMVHESELGNAAKVEEKLNNLCQVHDEICEIGKALNELWSYPILSLMAYGFLIFTAQLYFLYCATQYQSIPSLFRSAKNPFITVIVLSYTSGKCVYLIYLSWKTSQASKRTGISLHKCGVVADDNLLYEIVNHLSLKLLNHSVDFSACGFFTLDMETLYGVSGGITSYLIILIQFNLAAQQAKEAIQTFNSLNDTAGLVGAATDMDNISSTLRDFVTTTMTPAV  >DmelGr68a  MKIYQDIYPISKPSQIFAILPFYSGDVDDGFRFGGLGRWYGRLVALIILIGSLTLGEDVLFASKEYRLVASAQGDTEEINRTIETLLCIISYTMVVLSSVQNASRHFRTLHDIAKIDEYLLANGFRETYSCRNLTILVTSAAGGVLAVAFYYIHYRSGIGAKRQIILLLIYFLQLLYSTLLALYLRTLMMNLAQRIGFLNQKLDTFNLQDCGHMENWRELSNLIEVLCKFRYITENINCVAGVSLLFYFGFSFYTVTNQSYLAFATLTAGSLSSKTEVADTIGLSCIWVLAETITMIVICSACDGLASEVNGTAQILARIYGKSKQFQNLIDKFLTKSIKQDLQFTAYGFFSIDNSTLFKIFSAVTTYLVILIQFKQLEDSKVEDISQA  >DmelGr77a  MPLPLGDPLALAVSPQLGYIRITAMPRWLQLPGMSALGILYSLTRVFGLMATANWSPRGIKRVRQSLYLRIHGCVMLIFVGCFSPFAFWCIFQRMAFLRQNRILLMIGFNRYVLLLVCAFMTLWIHCFKQAEIIGCLNRLLKCRRRLRRLMHTRKLKDSMDCLATKGHLLEVVVLLSSYLLSMAQPIQILKDDPEVRRNFMYACSLVFVSVCQAILQLSLGMYTMAILFLGHLVRHSNLLLAKILADAEHIFESSQKAGFWPNRQELYKGQQKWLALELWRLLHVHHQLLKLHRSICSLCAVQAVCFLGFVPLECTIHLFFTYFMKYSKFILRKYGRSFPLNYFAIAFLVGLFTNLLLVILPTYYSERRFNCTREIIKGGGLAFPSRITVKQLRHTMHFYGLYLKNVEHVFAVSACGLFKLNNAILFCIVGAILEYLMILIQFDKVLNK  >DmelGr85a  MYSLIEAQLLGGKLVNRVMASLRRIIQRSLGYFCALNGILDFNTDIGTGNLRRYRVLFMYRLLHNFAVISLTLKFLFDFTDHFKYIESSTLITVNFFTYFTLVFFALLSSMGSCYQWQNRILAVLKELKHQRDLSRHMGYRVPRSKQNSIDYLLFALTVLLILRLSIHLATFTLSARMGFNHPCNCFLPECMIFSMNYLLFAILAEITRCWWSLQSGLKMVLLNRQLSTVAFNLWEIERLHTRFQCLIDLTSEVCSIFRYVTLAYMARNLWSGIVAGYLLVRFVIGNGLQDVELVYLVFSFITCIQPLMLSLLVNSMTSTTGSLVEVTRDILKISHKKSVNLERSIEWLSLQLTWQHTHVTIFGVFRINRSLAFRSASLILVHVLYMVQSDYISITN  >DmelGr89a  MLRFPHVCGLCLLLKYWQILALAPFRTSEPMVARCQRWMTLIAVFRWLLLTSMAPFVLWKSAAMYEATNVRHSMVFKTIALATMTGDVCISLALLGNHLWNRRELANLVNDLARLHRRRRLSWWSTLFLWLKLLLSLYDLLCSVPFLKGAGGRLPWSQLVAYGVQLYFQHVASVYGNGIFGGILLMLECYNQLEREEPTNLARLLQKEYSWLRLIQRFVKLFQLGIFLLVLGSFVNIMVNIYAFMSYYVSLHGVPLTISNNCLVLAIQLYAVILAAHLCQVRSAKLRKKCLQLEYVPEGLTQEQAMASTPFPVLTPTGNVKFRILGVFILDNSFWLFLVSYAMNFIVVILQTSFEHINHGEI  >DmelGr92a  MFEFLHQMSAPKLSTSILRYIFRYAQFIGVIFFCLHTRKDDKTVFIRNWLKWLNVTHRIITFTRFFWVYIASISIKTNRVLQVLHGMRLVLSIPNVAVILCYHIFRGPEIIDLINQFLRLFRQVSDLFKTKTPGFGGRRELILILLNLISFAHEQTYLWFTIRKGFSWRFLIDWWCDFYLVSATNIFIHINSIGYLSLGVLYSELNKYVYTNLRIQLQKLNTSGSKQKIRRVQNRLEKCISLYREIYHTSIMFHKLFVPLLFLALIYKVLLIALIGFNVAVEFYLNSFIFWILLGKHVLDLFLVTVSVEGAVNQFLNIGMQFGNVGDLSKFQTTLDTLFLHLRLGHFRVSILGLFDVTQMQYLQFLSALLSGLAFIAQYRMQVGNG  >DmelGr93a  MFSSSSAMTGKRAESWSRLLLLWLYRCARGLLVLSSSLDRDKLQLKATKQGSRNRFLHILWRCIVVMIYAGLWPMLTSAVIGKRLESYADVLALAQSMSVSILAVISFVIQARGENQFREVLNRYLALYQRICLTTRLRHLFPTKFVVFFLLKLFFTLCGCFHEIIPLFENSHFDDISQMVGTGFGIYMWLGTLCVLDACFLGFLVSGILYEHMANNIIAMLKRMEPIESQDERYRMTKYRRMQLLCDFADELDECAAIYSELYHVTNSFRRILQWQILFYIYLNFINICLMLYQYILHFLNDDEVVFVSIVMAFVKLANLVLLMMCADYTVRQSEVPKKLPLDIVCSDMDERWDKSVETFLGQLQTQRLEIKVLGFFHLNNEFILLILSAIISYLFILIQFGITGGFEASEDIKNRFD  >DmelGr93b  MVYGFTMSGLLVMPRILRCLNVSRISAILLRSCFLYGTFFGVITFRIERKDSQLVAINRRGYLWICLVIRLLASCFYGYSYDAWSGQYEDMYLRAFFGFRLIGCLICSVIILVMQFWFGEELINLVNRFLQLFRRMQSLTNSPKNRFGDRAEFLLMFSKVFSLLFVFMAFRLMLSPWFLLTLVCDLYTSVGTGMITHLCFVGYLSIGVLYRDLNNYVDCQLRAQLRSLNGENNSFRNNPQPTRQAISNLDKCLYLYDEIHQVSRSFQQLFDLPLFLSLAQSLLAMSMVSYHAILRRQYSFNLWGLVIKLLIDVVLLTMSVHSAVNGSRLIRRLSFENFYVTDSQSYHQKLELFLGRLQHQELRVFPLGLFEVSNELTLFFLSAMVTYLVFLVQYGMQSQQI  >DmelGr93c  MIERLKKVSLPALSAFILFCSCHYGRILGVICFDIGQRTSDDSLVVRNRHQFKWFCLSCRLISVTAVCCFCAPYVADIEDPYERLLQCFRLSASLICGICIIVVQVCYEKELLRMIISFLRLFRRVRRLSSLKRIGFGGKREFFLLLFKFICLVYELYSEICQLWHLPDSLSLFATLCEIFLEIGSLMIIHIGFVGYLSVAALYSEVNSFARIELRRQLRSLERPVGGPVGRKQLRIVEYRVDECISVYDEIERVGRTFHRLLELPVLIILLGKIFATTILSYEVIIRPELYARKIGMWGLVVKSFADVILLTLAVHEAVSSSRMMRRLSLENFPITDHKAWHMKWEMFLSRLNFFEFRVRPLGLFEVSNEVILLFLSSMITYFTYVVQYGIQTN  RL  >DmelGr93d  MKATKYSVGILRFMSFYARFLSLVCFRLRKQKDNNVWLEEIWSNRSRWKWISVTLRIVPLCIYAFTYAEWISNRMLITEKFLHSCSLVVSIPCYLSIIHLKICHGPEVTKLVNQYLHIFRLGTLDIRRRSQFGGGRELFLLILSVCCQIHEYVFILVIASRLCGFQHIIWWVSYTYVFIICNSIMCFGFIWHLSLGVLYAELNDNLRFESGFQTAFLRKQQRIRVQKSMALFKEISSVVTSLQDIFNVHLFLSALLTLLQVLVVWYKMIIDLGFSDFRIWSFSLKNLIQTLLPVLAIQEAANQFKQTRERALDIFLVGKSKHWMKSVEIFVTHLNLSEFRVNLLGLFNVSNELFLIIVSAMFCYLVFVTQCVIVYRRRYVI  >DmelGr94a  MDFTSDYAHRRMVKFLTIILIGFMTVFGLLANRYRAGRRERFRFSKANLAFASLWAIAFSLVYGRQIYKEYQEGQINLKDATTLYSYMNITVAVINYVSQMIISDHVAKVLSKVPFFDTLKEFRLDSRSLYISIVLALVKTVAFPLTIEVAFILQQRRQHPEMSLIWTLYRLFPLIISNFLNNCYFGAMVVVKEILYALNRRLEAQLQEVNLLQRKDQLKLYTKYYRMQRFCALADELDQLAYRYRLIYVHSGKYLTPMSLSMILSLICHLLGITVGFYSLYYAIADTLIMGKPYDGLGSLINLVFLSISLAEITLLTHLCNHLLVATRRSAVILQEMNLQHADSRYRQAVHGFTLLVTVTKYQIKPLGLYELDMRLISNVFSAVASFLLILVQADLSQRFKMQ  >DmelGr97a  MRFLRRQTRRLRSIWQRSLPVRFRRGKLHTQLVTICLYATVFLNILYGVYLGRFSFRRKKFVFSKGLTIYSLFVATFFALFYIWNIYNEISTGQINLRDTIGIYCYMNVCVCLFNYVTQWEKTLQIIRFQNSVPLFKVLDSLDISAMIVWRAFIYGLLKIVFCPLITYITLILYHRRSISESQWTSVTTTKTMLPLIVSNQINNCFFGGLVLANLIFAAVNRKLHGIVKEANMLQSPVQMNLHKPYYRMRRFCELADLLDELARKYGFTASRSKNYLRFTDWSMVLSMLMNLLGITMGCYNQYLAIADHYINEEPFDLFLAIVLVVFLAVPFLELVMVARISNQTLVETRRTGELLQRFDLQHADARFKQVVNAFWLQVVTINYKLMPLGLLELNTSLVNKVFSSAIGSLLILIQSDLTLRFSLK  >DmelGr98a  MEQMSGELHAASLLYMRRLMKCLGMLPFGQNLFSKGFCYVLLFVSLGFSSYWRFSFDYEFDYDFLNDRFSSTIDLSNFVALVLGHAIIVLELLWGNCSKDVDRQLQAIHSQIKLQLGTSNSTDRVRRYCNWIYGSLIIRWLIFIVVTIYSNRALTINATYSELVFLARFSEFTLYCAVILFIYQELIVGGSNVLDELYRTRYEMWSIRRLSLQKLAKLQAIHNSLWQAIRCLECYFQLSLITLLMKFFIDTSALPYWLYLSRVEHTRVAVQHYVATVECIKLLEIVVPCYLCTRCDAMQRKFLSMFYTVTTDRRSSQLNAALRSLNLQLSQEKYKFSAGGMVDINTEMLGKFFFGMISYIVICIQFSINFRAKKMSNEQMSQNITSTSAPI  >DmelGr98b  MVAQKSRLLARAFPYLDIFSVFALTPPPQSFGHTPHRRLRWYLMTGYVFYATAILATVFIVSYFNIIAIDEEVLEYNVSDFTRVMGNIQKSLYSIMAIANHLNMLINYRRLGGIYKDIADLEMDMDEASQCFGGQRQRFSFRFRMALCVGVWMILMVGSMPRLTMTAMGPFVSTLLKILTEFVMIMQQLKSLEYCVFVLIIYELVLRLRRTLSQLQEEFQDCEQQDMLQALCVALKRNQLLLGRIWRLEGDVGSYFTPTMLLLFLYNGLTILHMVNWAYINKFLYDSCCQYERFLVCSTLLVNLLLPCLLSQRCINAYNCFPRILHKIRCTSADPNFAMLTRGLREYSLQMEHLKLRFTCGGLFDINLKYFGGLLVTIFGYIIILIQFKVQAIAANRYKKVVN  >DmelGr98c  MEMEAKRSRLLTTARPYLQVLSLFGLTPPAEFFTRTLRKRRRFCWMAGYSLYLIAILLMVFYEFHANIVSLHLEIYKFHVEDFSKVMGRTQKFLIVAIATCNQLNILLNYGRLGLIYDEIANLDLGIDKSSKNFCGKSHWWSFRLRLTLSIGLWMVIIIGVIPRLTLGRAGPFFHWVNQVLTQIILIMLQLKGPEYCLFVLLVYELILRTRHVLEQLKDDLEDFDCGARIQELCVTLKQNQLLIGRIWRLVDEIGAYFRWSMTLLFLYNGLTILHVVNWAIIRSIDPNDCCQLNRLGSITFLSFNLLLTCFFSECCVKTYNSISYILHQIGCLPTAEEFQMLKMGLKEYILQMQHLKLLFTCGGLFDINIKLFGGMLVTLCGYVIIIVQFKIQDFALIGYRQNTSDTS  >DmelGr98d  MEANRSRLLAAARPYIQIYSIFGLTPPIQFFTRTLHKRRRGIVILGYACYLISISLMVIYECYANIVALQKDIHKFHAEDSSKVMGNTQKVLVVAMFVWNQLNILLNFRRLARIYDDIADLEIDLNNASSGFVGQRHWWRFRFRLALSVGLWIVLLVGLTPRFTLVALGPYLHWTNKVLTEIILIMLQLKCTEYCVFVLLIYELILRGRHILQQISVELEGNQSRDSVQELCVALKRNQLLAGRIWGLVNEVSLYFTLSLTLLFLYNELTILQIVNWALIKSVNPNECCQYRRVGTCLLLSINIFLSCLYSEFCIQTYNSISRVLHQMYCLSAAEDYLILKMGLREYSLQMEHLKLIFTCGGLFDINLKFFGGMVVTLFGYIIILVQFKIQFFAQSNFMQNINSTELKAYTA  >Aspi51990  GLTGKEKRDSNVGTSKFWSYDILYSIFLGRFSSPFSGKTKIKPKGHDDEDLDTPKIDQWDDVEYSPKDSFRQASSLFIFLGQWMCFIPLNHNEFRWLSFRVIFSVTAVLCNIGMTVASVLWVRNTGASILKGGRF  >Aspi18904  IALRGIRGKMLTMVEWREYRETYTSLTHLVKTVDSNINTIITLSVAGNVYFICAQLITEIDSITHSYFRTLFYLYSFLFLVFRTTAVVMLAAEIHDESSQIVPELFFCPYQSYCLETQRFLQEANSDYVALTGLNMFSVTRNFLLGIVGAILTYEIVLIQLQNPKEKPVVVMGNF  >Aspi18974  RQSYITNLFSKDQYRLCDTIEIYRECHRQLCELLEFLNYMYQFHLLITLAACFLKVLFNIYFTMFGYVIGTASAHSSKEEAEYLRTILWSTYYVMRFLTLIISAYLTVDRATKTRIVVSTISNRFLDSETKEELKLFANQISSRNIEFTACGFFTLNTHLITSAIAAGTTYLVILVQFHSDKE  >Aspi6070  FCINAKLGAAILSYGSLMKTEDRSKRLAQYTKLLISKMSNRFLDANVKEVLNLFWNYIAHKQLEFTVFGLFTLNARLIATAFGAGASYLVILLQFNPRKDLTVFENT  >Aspi53621  FRIFFFYRLAYAYPYVQLMLVYIVVHVSLKAASIASYEYNVHFQKMMDRGQPEDLEHYRLLWIELSNLMQESGNLYHNTNSLNNYVVIMVGLVLHIYRVILVFPKRAMELEVKRFTLEMILILYRHLYFLLVLYDHGDTITRQCSEQIHTRINLNRSAALINRKKYNFTERLFFKSLLLYKPKPSVHGYFKLNRATFMSMMGTVFTYLVVLIQLKRQISPDQLVKEDSNDKIAIVLHG  >Aspi19648  ILFSVEHISSLITGLITVKEDSEEILKHYCEKRFPQVFQYVEFRLWIGFLVLLINMVACHTFAFVDQLVIMFSIGLNDFFEDFNERIGQERKQVSL  >Aspi12848  KQFGACFSASGTVFFFGASAVAMVLFICLSMKWPKLIKRWEILERYHGHRKYLSYKYNILTLSIIVITFGAF  >Aspi14878  ADFASSQAQYTKVLISKINNRFLDADTKEELELFWNHITSSKLEFTACGFFTLNTRLIASAFVAGTTYLVILLQFNPEKPAVIQGA  >RpadGr1  MHDVWEAIYFVFSFTYLVGRTCAVSLYAASINDQSKKPKAILFSVPTESYGVEVARFLMQVTSDELALTGCNFFSVTRTLMLTVAGTIVTYEIVLIQFNSVNSE  >RpadGr2  VLPSMYWRKSRETYNILASLTHDFDEFLSPVILLSFGHNLYFICLQLLNSLKPMHSSWEALCFAFLFTYLVGRTCAVSLYVASINDQSKKPKGVLFSVPAECYGVEVERFLTQVSADELSFTGCNFFSVTRTFMLTVAGTIVTYEIVLIQFNNVASGVADQNNTLINICP  >RpadGr3  VLPSTYWRKSRETYNLLASLTKDFDEFLSPVILLSFANNLYFICLQLLNSLKPMHDVWEAIYFVFSFTYLVGRTCAVSLYAASINDQSKKPKAILFSVPTESYGVEVARFLMQVTSDELALTGCNFFSVTRTLMLTVAGTIVTYEIVLIQFNSVNSE  >RpadGr4  MSNENIFRPVYLKDRNFIESGYERQFKQSSLSDSAARSSYNMFSLKENKGNIALEIQDDLGMPVNYVRIKALEDSIRKDIISDTNSMHKAITPVLILAQMFAILPVQGIRGQNTSYLVFNWFSCTVIYVFIVIAASLLILSFSLIKIYMTGLTYYSTGEIMFFGSSLVIYILFIHLARE  >RpadGr5  VSSALAAQFKKLTKAMHSVRGQMLTMSQWQEYRETYTSLTHLVKKIDDHINVIVALSIGSNIYFICAQLITEIDSIKHS  >RpadGr6  NTWIAVLVLIVNSVSTLTLCFSDQIIIMGSLALGNYFEIFNERMKTHKGKKLTPDQWKTLRVDYTRLCNLARLLNDCLCHLLCVSLLIHVYIICVEMHQGVVRTNENYSL  >RpGr7  YTMRLSCAGEYRYSFDKRLIPVWTLYACLNAYVMHYNYVESVRYVAGSFRKCFEESYCFALIELFNRNVGP  >RpadGr8  SVYDELCTLAGHVNRAYAPEMLLQWAYNIVRVIMVMFRLLELVSSLDGPMASVMPYLLVQHFGELFIFVVHTSCTCTVGDHLSGEVGTYYLY  >RpadGr13  NIVGNVFGVTFMMLSAFNTLMSDGANTIRITLYFLYESLIRTLQMYFIIDACHTTVEQANFINITFQKKMNEYNEVNLTKRKVQLFILEMLDHKIEFVIYGFICLDFEMFVSIIGTIATYFLILVQLGSSPDALVKPLNSTIPIANNTKLN  >RpadGr14  FFFTSIFMIIYFFYSVFALLHNVIKDNSGTVNPQYLIIALAYFILGVMVLNRLINAGHSARFADELKRGCGRTVESGRHLLEFWLRTVGSAALLCMVHFKYRLFTDMPTAAIAVTTTVPLCCCVYCGSTMECQY  >RpadGr15  CSRDNYCSVFGMLLSHTVISPIVVSLVLVRLPRSVDVLNMTARLLLRSRYPRRRLSPNTYVVILFSAVIVFKLVTTVMSIPQKYPDLYYPYFTAYMVPIVFINLVSILCVVAQQSYE  >RpadGr16  ALEAKSLITDTNNGIMDSSTKEELQLFINQICSSSTEFNAYDFFTLNTQVIKSAIAAGATCLVILVQFHSGKN  >RpadGr17  FFACVLQYSYRFCVITILSNLTTKQAVNAKTLITDINNRYLDTSTQEELQLFYSQISSRCIQFTACDLFTLNTRLITSAIAAGATYLVILVQFHSGKN  >RpadGr18  LIRQSIDALNNLFGVHMGLSVFYLWLMALFDIYYEMFYNSRSELLVYCWLLQYTLRLLMIILMAHFTTKQ  >RpadGr35  TIRQVVKTKKLITKMNNRHTDINTKEELQLFYNQLTVCSSEFTICDILTIENSLLTSIWSAGATYILILVQ  >RpadGr37  EQIIPLIIHVQMVTFLMFIIVFVSFINEKKMEMISYLRLYRISNLHLDIKRQIKMFMNQISACDSNQISAFGFFDINLNLVTTILVLLITGIITLIQMKNHPIILKLNNDTKSF  >RpadGr41  IPYVMDYVVTITSCFFLQNLYVRFQSVNDLWKCLPPGLVAVPGLWTNIEIVVLMENTRLMHSELCELLKKFTLGYGPLLLGFYTFSFISMLIGFYFIFNAEPLTNNQYSEEKYVIVI  >RpadGr43  YLLCTQNSFLIMSIIIAASRVNDTKRKMISYLRLIKISSLPVDVKLQVKMFMNQLTVFNMDEISAFGIFNINLNLVMAIIILLITGLTTLLQMKNNSNMIQ  >RpadGr45  IIPLKVVNIITFSFYFFRLSFAIDVTVVISTYFYLQNLECRFQTLNGFWTQLPDGLTTITVVSGGWTHVEIIML |
| **IRs** |
| >DmelIr25a  MPRNAFGQCTLTDVIPSLWIVFINEVDNEPAAKAVEVVLTYLKKNIRYGLSVQLDSIEANKSDAKVLLEAICNKYATSIEKKQTPHLILDTTKSGIASETVKSFTQALGLPTISASYGQQGDLRQWRDLDEAKQKYLLQVMPPADIIPEAIRSIVIHMNITNAAILYDDSFVMDHKYKSLLQNIQTRHVITAIAKDGKREREEQIEKLRNLDINNFFILGTLQSIRMVLESVKPAYFERNFAWHAITQNEGEISSQRDNATIMFMKPMAYTQYRDRLGLLRTTYNLNEEPQLSSAFYFDLALRSFLTIKEMLQSGAWPKDMEYLNCDDFQGGNTPQRNLDLRDYFTKITEPTSYGTFDLVTQSTQPFNGHSFMKFEMDINVLQIRGGSSVNSKSIGKWISGLNSELIVKDEEQMKNLTADTVYRIFTVVQAPFIMRDETAPKGYKGYCIDLINEIAAIVHFDYTIQEVEDGKFGNMDENGQWNGIVKKLMDKQADIGLGSMSVMAEREIVIDFTVPYYDLVGITIMMQRPSSPSSLFKFLTVLETNVWLCILAAYFFTSFLMWIFDRWSPYSYQNNREKYKDDEEKREFNLKECLWFCMTSLTPQGGGEAPKNLSGRLVAATWWLFGFIIIASYTANLAAFLTVSRLDTPVESLDDLAKQYKILYAPLNGSSAMTYFERMSNIEQMFYEIWKDLSLNDSLTAVERSKLAVWDYPVSDKYTKMWQAMQEAKLPATLDEAVARVRNSTAATGFAFLGDATDIRYLQLTNCDLQVVGEEFSRKPYAIAVQQGSHLKDQFNNAILTLLNKRQLEKLKEKWWKNDEALAKCDKPEDQSDGISIQNIGGVFIVIFVGIGMACITLVFEYWWYRYRKNPRIIDVAEANAERSNAADHPGKLVDGVILGHSGEKFEKSKAALRPRFNQYPATFKPRF  >DmelIr40a  MACNELHNGYRAKFLTIVYWIAATYVLADVYSAQLTSQFARPAREPPINTLQRLQAAMIHDGYRLYVEKESSSLEMLENGTELFRQLYALMRQQVINDPQGFFIDSVEAGIKLIAEGGEDKAVLGGRETLFFNVQQYGSNNFQLSQKLYTRYSAVAVQIGCPFLGSLNNVLMQLFESGILDKMTAAEYAKQYQEVEATRIYKGSVQAKNSEAYSRTESYDSTVISPLNLRMLQGAFIALGVGSLAAGVILLLEIVFIKLDQARLWMLCSRLQWIRYDRKV  >DmelIr68a  MRCLWILIVAFISLAMATSIPIPIANPAPLSGYEMQLKILLQKILWVANVKRCFAVITDDLHYPIYDRIFFESVGRRVIPFFVMRTNESDDLQRPSRQVELFVKAIKSSDCELNVITILNGWQVQRFLGYIYDNRSLNMQKKFVLLHDLRLFESDMIHLWSVFIDAIFLKRQLDNKYTISTIAFPGILSGVLVMKNIANWELGKGLNGRILFADKTSNLFGTSLPVAISEHVPMVLWANATKSFQGVEVEIMNALGKALNFKPVYYKPNQTENMDWTELDGGASVAYGSGNPDGYAQNGTHIDSMLVDEVAAHSARFAIGDLHLFQVYLKLVELSAPHNFECLTFLTPESSTDNSWQTFILPFSAGMWVGVLLSLFVVGTVFYAISFLNAIINGNVSSEFFRCLRPNRNVPMDPKIYRRISFRIAISRYRSSKGDRMPRDLFDGYTNCILLTYSMLLYVALPRMPRNWPLRVLTGWYWIYCILLVATYRASFTAILANPAARVTIDTLEDLLRSHIPPSTGATENRQFFLEANDEVARKVGEKMEVFGYSDDLTSRIAKGQCAYYDNEFYLRYLRVADESGSALHIMKECVLYMPVVLAMEKNSALKPRVDASIQHLAEGGLIAKWLKDAIEHLPAEALAQQEALMNIQKFWSSFVALLIGYVISMLTLLAERWHFKHIVMKHPMYDVYNPSLYYNFKRIYPQH  >DmelIr31a  MNLLISMFILILAAGEGEIIPSMEESVVTNFVKSLVKTKQAIVFSCLFKDFKEISLALMRINQFVSVVNLNQSYSLTSILTRENYARTSVMVNARCSGSSELLFEASENRYFNKTYQWFLWGVDLEVQSLFPLNLNYVGPNAQITYVNETADGYAYWDIHSKGRHLKSNLEINLIATLINDTLNIARDIFHLQSIDFRGQFNGLTLRGASVIDKEDIISNEQIESILSRPTKDAGVAAFIKYHYELLGLLRERFNFTVNFRNSRGWAGRLGNTTFRLGLLGIVMRNEADIAASGAFNRINRFAEFDTIHQSWKFETAFLYRYTSDLDTHGKSGNFLSPFSDRVWLFCLLTLGAFSIIWVLFEIIDYKILRIRVNSQKLEHLNQKSSVICIKTTCIERILQTFGACCQQGLDPNPVDRSVRFLVMTLFLFSLVMYNYYTSSVVGGLLSSSDQGPSTVDEITASPLKISFEDIGYYKVLFRESQNRSITRLIEKKLSSSRSLNELPIFSHIEDAVPYLKAGGFAFHCEVVDAYPVISEYFDANEICDLREVSGLMEVEILNWILHKNSQYTEIFKTAMCNAQEKGFVERILRRRQIKKPACQSLYTVYPVSLSGVLPGFVILICKSINKFS  >DmelIr7c  MLHSAVHNVSLVYALVWAIDNYYGMATSTPLAVVQFPTSRESRRLHNDLIDAALGRSSGTGRIQFLLEDDRVEMTETDTDPPPPSGLTGRPIAIWFLDSLRSYFRLEMYLNQLGSPYKRNGFFLVIYTGLEDQPMESLKIMFRRLLNMYVLNVNVFLQRDGTVHLYTYYPYGPHHCQSSLPVYYTAFQDLAAPANGFGLTKPLFPRKLTNMHGCEMVVATFEHRPYVIIEDDPKTPGGRSIHGIEGLIFRSLAERMNFTIKLVEQKDKNRGEILPDGNFTGILKMMVDGEVNLTFVCFMYSKARSDLMLPSTSYTSFPIVLVVPSGGSISPMGRLTRPFRYIIWSCILVSLIFGFVLICLLKITALPGLRNLVLGRRNRLPFMGMWASLLGGLALYNPQRNFARYILVMWLLQTLILRAAYTGQLYLLLQDVEMRSPIKSLSEVLAKDYEFRILPALRTIFKDSMPTTNFHAVLSLEESLYRLRDEDDPGITVALLQPTVNQFDFRSGPNKRHLTVLPDPLMTAPLTFYMRPHSYFKRRIDRLIMAMMSSGIVARYRKMYMDRIKRVSKRRNLEPKPLSIWRLSGIFVCCAGLYLVALIVFILEILTTNHRRLRRAFNVINRYAA  >DmelIr76a  MENLLVESYYFSTVLSFFAQQFFADSHATCIFWHPAFDFRLETVHPMPLIIMDWHRWANRSDQDVYDYKIKEDEFEGKGIPYNDWTLRLTVAIERSHCETFIAFQEQIPEFARYFYHASIYSIWRSLRNRFMFVYTKEFEDKKDSYLSGYIFQDQPNILVITSQYLNSSTFEIKTNRFVGPRNFNKNPEPVEFYILQRFDAKGTKATWETQSAMSSKMRNLKGREVVIGIFDYKPFMLLDYEKPPLYYDRFMNTTDVTIDGTDIQLMLIFCELYNCTIQVDTSEPYDWGDIYLNASGYGLVGMILDRRNDYGVGGMYLWYEAYEYMDMTHFLGRSGVTCLVPAPNRLISWTLLLRPFQFVLWMCVMLCLLLESLALGITRRWEHSSVAAGNSWISSLRFGCISTLKLFVNQSTNYVTSSYALRTVLVASYMIDIILTTVYSGGLAAILTLPTLEEAADSRQRLFDHKLIWTGTSQAWITTIDERSADPVLLGLMEHYRVYDANLISAFSHTEQMGFVVERLQFGHLGNTELIENDALKRLKLMVDDIYFAFTVAFVPRLWPHLNAYNDFILAWHSSGFDKFWEWKIAAEYMNAHRQNRIVASEKTNLDIGPVKLGIDNFIGLILLWCFGMICSLLTFLGELWRGQG  >DmelIr8a  MELPLLVLLLALRFAGSEVLKITFWIEPVQRAEFDTDIAMVLKELDALRLDVKVDDTTLTLTRSEDGLDMQRFCEILSTVGASAVIDLTYSHWEEGYNLVRSLGIGYVRLERIMRPFLDMFGDFMRQKRANNVAMVFMNARDAVEAMQQMLVGYPFRTLIMDASQTDPGQHFLERIRSLRPAPTYIALFARAAAMNGIFEKVQKADLFQRPLEWHFVFLDTRDRVFKYRRQAELCTRFTLNPRAICRSMPMPDLYCGSGFTMQRAMLLNVLRSLINAAQVSPGYPLAIYQDCNATASSSEVSDPLEKDDYNWLDMVHWSNFLAYAPPLPHIQDQFQSPVPGLTFAVNISAGYYSSEHEAKTDLAAWSSVGEMRLLNETISPARRFFRIGTAESIPWSYLRREEGTGELIRDRSGLPIWEGYCIDFIIRLSQKLNFEFEIVAPEVGHMGELNELGEWDGVVGDLVRGETDFAIAALKMYSEREEVIDFLPPYYEQTGISIAIRKPVRRTSLFKFMTVLRLEVWLSIVAALVGTAIMIWFMDKYSPYSSRNNRQAYPYACREFTLRESFWFALTSFTPQGGGEAPKAISGRMLVAAYWLFVVLMLATFTANLAAFLTVERMQTPVQSLEQLARQSRINYTVVKDSDTHQYFVNMKFAEDTLYRMWKELALNASKDFKKFRIWDYPIKEQYGHILLAINSSQPVADAKEGFANVDAHENADYAFIHDSAEIKYEITRNCNLTEVGEVFAEQPYAVAVQQGSHLGDELSYAILELQKDRFFEELKAKYWNQSNLPNCPLSEDQEGITLESLGGVFIATLFGLVLAMMTLGMEVLYYKKKQNALEITQVRPVNDSSGSGGNSSTAPPTATSTTKQAWHIPVLEAEEKPAKVSPPPSFETATFRGKKLPARITLGDGKFKPRHGLYARRNLGASDSHSGYME  >DmelIr75a  MQLVQLANFVLDNLVQSRIGFIVLFHCWQSDESLKFAQQFMKPIHPILVYHQFVQMRGVLNWSHLELSYMGHTQPTLAIYVDIKCDQTQDLLEEASREQIYNQHYHWLLVGNQSKLEFYDLFGLFNISIDADVSYVKEQIQDNNDSVAYAVHDVYNNGKIIGGQLNVTGSHEMSCDPFVCRRTRHLSSLQKRSKYGNREQLTDVVLRVATVVTQRPLTLSDDELIRFLSQENDTHIDSLARFGFHLTLILRDLLHCKMKFIFSDSWSKSDVVGGSVGAVVDQTADLTATPSLATEGRLKYLSAIIETGFFRSVCIFRTPHNAGLRGDVFLQPFSPLVWYLFGGVLSLIGVLLWITFYMECKRMQKRWRLDYLPSLLSTFLISFGAACIQSSSLIPRSAGGRLIYFALFLISFIMYNYYTSVVVSSLLSSPVKSKIKTMRQLAESSLTVGLEPLPFTKSYLNYSRLPEIHLFIKRKIESQTQNPELWLPAEQGVLRVRDNPGYVYVFETSSGYAYVERYFTAQEICDLNEVLFRPEQLFYTHLHRNSTYKELFRLRFLRILETGVYRKQRSYWVHMKLHCVAQNFVITVGMEYVAPLLLMLICADILVVVILLVELAWKRFFTRHLTFHP  >DmelIr60b  MRRSLYLIIAIGLVDVHCVSLRYILNALENELQYRAILLVESASEIESCWEQKYIQGAVPILNFNANQSLYLKDALNTNILALVCLNENVESTMQALYENLEDMRDTPTILFVLSDSKVQDVFLECLRRKMLNVLAFKGLDRGFVYSFRAFPTFRVIERNVMDILQYFEQQLEDLGGHTLTTLPDNIIPRTVVYKSPDGSRQLAGYLYPFLRNYVSTINATLKVCWHLVPEDGMIQLGEVVRLSEIHDVDFPLGMHGIEHGSTSQNVPLEVSSWFLMLPMEPSLSRAQFFIMLGFEKVTPVLLLLTILLSTAHRIEMGLRPSWRCYVLGDRVLQGTLGQAFFLPRRLSVKLMLVYSLILLNGFTFSNYSITSLETWLVHPPSGHPIHSWEQMRTLNLKVLIVPSELDSMTKALGKQFTESNSDLFELSKSGNFQDKRLAMDQSYAYPVTCTLWPLLEHAQIRLPKPEFRRSREMVLIPLLIMAMPLPKNSMFHKSLNRYRALTHQSGLYEFWFKRSFNELVALRKIHYKVNGDHQIYRDFEWQDFSYVWLGFVGGTIASILVLLAEIGYHRWQLNQN  >DmelIr56b  MLLDTDLASGVIRSPYSFDIPHAFIFNETQFVVPKFCGPYMEIVKHFAEVYHYQLFLDSLESLPKKSVVEQDIISGKYNLSLHGVIIRPEETSDFFNATQHSYPLELMTNCVMVPLAPELPKWMYMVWPLGKYIWTCLFLGTFYVALLLRYVHWREPGNATRSYTRNVLHAMALLMFSANMNMSVKLKHASIRVIIFYTLLYIFGFILTNYHLSHMTAFDMKPVFLRPIDTWSDLIHSRLRIVIHDSLLEELRWLPVYQALLASPSRSYAYVVTQDAWLFFNRQQKVLIQPYFHLSKVCFGGLFNALPMASNASFADSLNKFILNVWQAGLWNYWEELAFRYAEQAGYAKVFLDTYPVEPLNLEFFTTAWIVLSAGIPISSLAFCLELFIHRRKQRRPQYERFECYDY  >DmelIr54a  MWTVITGIVLWAPVLVAGSAVDFIFRAAAEHSLSVIMIRIDYCPYNWAKDIFENQTIPVVVLSDSETFINIRMFSRPLHVACLPGHELQKDLALLENFTSSLMDFPSQKKIVYISNNFSDPTRMDYIFETCYHRRIWNIVGLLASDEHRYFYRYHLYPSFRTEYRSLESSTIFDKDFPNMHGHPLTVMPDQWLPRSVLYVDRRTGKQILAGSVGRFFHVLSWKLNATLQLSKKVTTGRFLNATALKELSESFSVDVPASLTIMERVEQLASTSYPMEVTHVCLMVPVARRIPIKDIYFILSSASNMFLAIVIVSSYGLALNLLRNMTHRDVRLVDFVLNDKALRGILGQSFNLPLSRSFSTRLIFLMLGIVGLNVSSIFGAGLDTLMAHPPRQFQARSFAGLRRTKIPLVTTEEDFPTWMKLRVPMLVVNVSEYNHLRNGRNTSNAYFASRLYWNLFSEQQKRFTRELFIYSTDDCLWSLALLSFQWPQNSLFTEPVSQLILEVNANGLYDFWVGMHYYDMTAAGLSGLEDPSLQLKEREHPTSLRIVDFQWMWQAYGTFMVIAILVFLLEVSWHRITSLFVSLVY  >DmelIr85a  MSIQWLKHILLLAILVNLAGTRENHIPLDLKKSSIVMVKMSQILCKARIKVLFVYFENQTSHEHTGQILKEVTKCDISNQNTPLEAVKDDGILMYMVMITTNISQPLELSLIRKKSAAKHRSHVFLLVRDADTVSDAWMRASFRQFWKIWLLNIVILYWRDGRLNAYRYNPFMDNYLIPVDNKPNEVPTLEQLFPKTIPNMQRKPLRMCIYKDDVRAIFWRQGTILGTDGLLAAYVAERLNATMMITRPHSYNNHNLSSDICFLEVAKEYVDVAMNIRFLVPDTFRKQAESTVSHTRDDLCVIVPKAKTAPTFWNIFRSFGSLVWALILVSVLVANVFCYILKSEVGRVPMQLFAGALTMPMTQIPPNHSIRLFLIFWLYFGLLICSAFKGNLTSMMVFQPYLPDINQLGALARSHYHIIIRPRHVKHIQHFLTLGHKHESRIREQMLEVSDTQMYEMMRNNDIRFAYLEKYHIARFQVNSRVHMHLGRPLFHLMNSCLVPFHAVYIVPYGSPYLGFLDSLIRSSHEFGFERYWDRIMNSAFIKSGVKVVNRRRGSGNDEPVVLKLQHFHAVFALWLVGIGMACIVLAWEHLTHNYNLAVTKRRD  >DmelIr67a  MLPILVPVLLLFNETSWINPILTSIYKDRHHETVLLLQHSQHGNASGLERFPWPVFSFNEQMDFYVRGKYNSEMLVLIWQTGNSDWDLDLWQALDRSLLNMRKVRVLLLRKWEKIPTADVAATAEHLLFLHVAVIGQGNRIYRLQPYAPQSWLQVDPIESPIFIKIRNYFGRYIVTLPDQFPPRSIVYRNPKTDEIQMTGYVYKFLLEFIRIYNFTFRWQRPIVQGERMNLILLRNMTLNGTINLAISLCGFETPSELGVFSDVYDMEEWYIMVPRAQEISIADVYVVMVSGNFLIVLIIFYFIFTILDTCFGPLLLKERVDWSNLMLNERMISGIMGQSFNMSARNTISSKVTNATLFLLGLVLSTLYAAHLKTLLTKRPTSQQISNFKQLRDSPVTVFFEEAERFYLKHAWDRpadIRYIKDQLNFRETIEYNALRMGLNRSNAFSALTSEWMIVAKRQELFKQPIFTVQPELRVIQTSVLLSLVMQSNSIYEDHINDLIHRVQSAGIVEYWKHQTLREMITMGMISQKDPFPYVAFREFKVGDLFWIWLLWVSFLFMSFVIFLCELLVDCFISKTLIRNKRPH  >DmelIr21a  MSYYWVALVLFTAQAFSIEGDRSASYQEKCISRRLINHYQLNKEIFGVGMCDGNNENEFRQKRRIVPTFQGNPRPRGELLASKFHVNSYNFEQTNSLVGLVNKIAQEYLNKCPPVIYYDSFVEKSDGLILENLFKTIPITFYHGEINADYEAKNKRFTSHIDCNCKSYILFLSDPLMTRKILGPQTESRVVLVSRSTQWRLRDFLSSELSSNIVNLLVIGESLMADPMRERPYVLYTHKLYADGLGSNTPVVLTSWIKGALSRPHINLFPSKFQFGFAGHRFQISAANQPPFIFRIRTLDSSGMGQLRWDGVEFRLLTMISKRLNFSIDITETPTRSNTRGVVDTIQEQIIERTVDIGMSGIYITQERLMDSAMSVGHSPDCAAFITLASKALPKYRAIMGPFQWPVWVALICVYLGGIFPIVFTDRLTLSHLMGNWGEVENMFWYVFGMFTNAFSFTGKYSWSNTRKNSTRLLIGAYWLFTIIITSCYTGSIIAFVTLPAFPDTVDSVLDLLGLFFRVGTLNNGGWETWFQNSTHIPTSRLYKKMEFVGSVDEGIGNVTQSFFWNYAFLGSKAQLEYLVQSNFSDENISRRSALHLSEECFALFQIGFLFPRESVYKIKIDSMILLAQQSGLIAKINNEVSWVMQRSSSGRLLQASSSNSLREIIQEERQLTTADTEGMFLLMALGYFLGATALVSEIVGGITNKCRQIIKRSRKSAASSWSSASSGSMLRTNAEQLSHDKRKANRREAAEVAQKMSFGMRELNLTRATLREIYGSYGAPETDHGQLDIVHTEFPNSSAKLNNIEDEESREALESLQRLDEFMDQMDNDGNPSSHTFRIDN  >DmelIr60e  MVIKMISFLLVSVLLCLVGASDSESMQVQVLQDLNLALQTELNVFIDFECCATSEILHKLDSPRILLSSNSREARDLRIRGNFTESTLIIVSVMDSDLNPLVASLLPRLLDELHELHIVFLSNEEPGFPKQDLYTYCFKEGFVNVILMSGKGLYSYLPYPSIQPISLSNVSEYFDRARIIRNFQGFPVRILRSTLAPRDFEYSNEQGGLVRAGYLFTAVKELTYRYNATIESVPIPDLPEYDVYLAVAEMLHTKKIDIVCYFKDFSLEVAYTAPLSIIREYFMAPHARPISSYLYYSKPFGWTLWAVVISTVLYGTVMLHLAARGARVEIGKCLLYSLSHILYNCHQKIRVAGWRDVAIHGILTIGGFILTNVYLATLSSILTSGLYDEEYNTLEDLARAPYPSLHDEYYRSQMKAKTFLPERLRRNSLSLNATLLKAYRDGLNQSYIYILYEDRLELILMQQYLLKTPRFNMIRQAVGFTLESYCVSNSLPYLAMTSEFMRRLQEHGISIKMKADTFRELIHQGIYTLMRDDEPPAKAFDLDYYFFAFVLWTVGLISSLLVFFAELVSGHL  >DmelIr41a  MFIDLSWSLVLSAIVGKYLNESTICIFWNDKFEFQLLHKSDYISFVGINIKSFDDNGGHYIIDTGLKKKELQNKHLFLDELVIKIIISIEVTHCETFVVFDKDIDRFVNAFNKASVYSIWRSLHNKFVFAHIANESPESRNHFFEDQPNILFVVRDHSSASSFDIKTNKFVGRKAENPSQMILVDRYLASEQRFQFGKSLFADKLNNLQGREVIIAGFDYPPYTVIKHNMSTNAQDMGVSGESDFKNVYIDGTETRIVLNFCEQFNCTIQIDSSAANDWGKVYPNMSGDGALGMLINRKADICIGAMYSWYEDYTYLDLSMYLVRSGITCLVPAPLRLTSWYLPLEPFKETLWAAILLCLCAEATGLVLAYKSEQALYVLPGYREGWWTCTSFGVCTTFKLFISQSGNSKAYSLTVRVLLFACFLNDLIITSIYGGGLASILTIPSMDEAADTVTRLRFHRLQWAANSEAWVSAIRASDEALVKDILYNFHIYSDDELLRLAQDQHMRIGFTVERLPFGHFAIGNYLGPQAIDQLVIMKDDIYFQYTVAFVPRLWPLLDKLNTLIYSWHSSGFDKYWEYRVVADNLNLKIQQQVQETMTGTKDIGPVPLGMSNFAGFIIVWILGSAIATLTFLLELSLTYILKQSNLK  >DmelIr94c  MSKVFKLLVLPLIYLSLTKGSKNPQLKFLRELINVIEEGREIRTIMVIKHSRDEYCHLDQWNPRGSPILRTNEMGSIRISGYFNDQAVILACMGENSDYGLLKSLANAMDNMRQERIILWSEREPTKMLMDYISQQADRYNFAQIIIVTMNEDVDAVPSLHQLNPYPTPRFRQITNISNIRRTSFFGCGLSFQGKTAILKESVVSNIRFKVWSPSGPIPLSELKDYEIVQFAVKYNLSLKLYDQNESKSDHFDIQLGPLFITKDFPTQMAFVSPNTACSLIVIVPCSPKWRFMDVLHKLGVLKLIGCLLIAYAVFVLIETLILWLTHRISGREVRLTSLNQLLNPRAFRGILGLPFPEFRRSSISLRQLFLVISVFGLVYSNFVSCTLSALLTKPAQNPQVRNFKELRDSGLITIMDKYTHSFIEKHIDPEFFDHVLPHYLILQKKEALRMIWNFNDSYSYVMYTTTWKSLNTVQKSFDERVFCESESLTIAWNLPRMYVLGNNSVLKWMLSRYITYMPQTGIPDSWTEQLPKVLKLLYNVTSPRRIKEGAVPLSIQHLSWIWHLLFIGESIATLVFIVEILLQKSNQHTSNMRERSSEDDDFV  >DmelIr94b  MSLIFNLLFILILSQAVSQETEFLQLKYLNNIVRSMIKLHKMETLVIVKHHLDNNCSLQNWNAHGMGIIRTNDQGKLIMKDTFNSRTLAIICIGQNSHITLLRNVFETFGKVQQKKIILWTQMELKEKFFQEISKKSRDLKLLNLLVLKAVTKDKLLIYRLNPFPSPHFKRIENIWTPNDTLFMDTKFNFHGMTAVVKHDYNWTIQMGNIRKFPISRIEDKEVIEFALKYNLTLQFFNDVERFDIELRKRIILKSNSTQPIDSGIPMVFSSLLIVVPCGNYLSIQDVIKVSGIEKWIFYIILVYVIFVLIEITFLGVTILISRQSRHQMIPNTLVNLCAFRAILGLPFPETRRTSLSLRQLFLAIALFGMIFSIFINCKLSSMLTNPCPRPQVNNFEELKTSGLTVVMDHDAENFIEKEIGVDFFNQYMPRKVTLTFTERAKLLFSLKGNHAFTLFSESFAIIESYQRSKGLRAHCTSEDLIVAERVPRIYILENNSILDRPLRRFIRQMQESGITNHWLKNIPSSLEKNLMQITIPYDRERVHPLSIEHLTWLWCILILGYSISMIVFFVEMSLKRRKKNLENRAPNICIC  >DmelIr93a  MNPGEMRPSACLLLLAGLQLSILVPTEANDFSSFLSANASLAVVVDHEYMTVHGENILAHFEKILSDVIRENLRNGGINVKYFSWNAVRLKKDFLAAITVTDCENTWNFYKNTQETSILLIAITDSDCPRLPLNRALMVPIVENGDEFPQLILDAKVQQILNWKTAVVFVDQTILEENALLVKSIVHESITNHITPISLILYEINDSLRGQQKRVALRQALSQFAPKKHEEMRQQFLVISAFHEDIIEIAETLNMFHVGNQWMIFVLDMVARDFDAGTVTINLDEGANIAFALNETDPNCQDSLNCTISEISLALVNAISKITVEEESIYGEISDEEWEAIRFTKQEKQAEILEYMKEFLKTNAKCSSCARWRVETAITWGKSQENRKFRSTPQRDAKNRNFEFINIGYWTPVLGFVCQELAFPHIEHHFRNITMDILTVHNPPWQILTKNSNGVIVEHKGIVMEIVKELSRALNFSYYLHEASAWKEEDSLSTSAGGNESDELVGSMTFRIPYRVVEMVQGNQFFIAAVAATVEDPDQKPFNYTQPISVQKYSFITRKPDEVSRIYLFTAPFTVETWFCLMGIILLTAPTLYAINRLAPLKEMRIVGLSTVKSCFWYIFGALLQQGGMYLPTADSGRLVVGFWWIVVIVLVTTYCGNLVAFLTFPKFQPGVDYLNQLEDHKDIVQYGLRNGTFFERYVQSTTREDFKHYLERAKIYGSAQEEDIEAVKRGERINIDWRINLQLIVQRHFEREKECHFALGRESFVDEQIAMIVPAQSAYLHLVNRHIKSMFRMGFIERWHQMNLPSAGKCNGKSAQRQVTNHKVNMDDMQGCFLVLLLGFTLALLIVCGEFWYRRFRASRKRRQFTN  >DmelIr92a  MLLQPLVMHLSQLLRIIVGQYFAEFPSILIVYNNSASTTPLQLEYLSALELVLRELSKPIRLQWINVAFLKDLNDLEDQVMGALNSSVTEGFITILSQTHHFIHARYYATRNANVRLKDKRYLFLCEDESPAELLCMDILQFYPHHLMVRPGTETAPTGPTGPHPDPRRGGGASVSTKNKDDGEGGAGNKTTSPYRDINFELWTQKFVGAVGNLDALLLDAFLPNETFANRVELYPNKLLNLQRRSLLVGSITYVPYTITNYVPAGQGDVDPIHPQWPNRSLTFDGAEANVMKTFCQVHNCHLRVEAYGADNWGGIYDNESSDGMLGDIYEQRVEMAIGCIYNWYDGITETSHTIARSSVTILGPAPAPLPSWRTNIMPFNNRAWLVLISTLVICGTFLYFMKYVSYRLRYSGTQVKFHHSRKLEKSMLDIFALFIQQPSAPLSFDRFAPRFFLATILCATITLENIYSGQLKSMLTFPFYSAPVDTIEKWAQSGWKWSAPSIIWVHTVQSSDLETEQILARNFEVHDYSYLSNVSFMPNYGFGIERLSSGSLSVGDYVSTEALENRIVLHDDLYFDYTRAVSIRGWILMPELNKHIRTCQETGLYFHWELEFIDKYMDKKKQEVLMDLANGHKVKGAPQALDVRNIAGALFVLAFGVAFAGCALVAELLIHRMDLSK  >DmelIr87a  MSTPEQRFWLAALLFLLSQHSEVRGFGINLMKVQTEDKGQEACILALLRKYFDSGDGLSGSVLCINRNYQLPNIEEQLLRGVNNYENYPWSLLITNSREGPSPAKFLMNEKPQCYFLIVDNLEDEDLDEVFEHWKGMVNWNPLAQFVVYLASLEETDEEMNDLMVELLLTFINKKIFNVNVIGQSEENQFYYGKTVFPYHPDNNCGNRVISVELLDACDYPSEETDSEDENDEDEGDGAQEEDDGPQEEGDGEQEEEDGPQEQEDGDQAKGDEGQENDDGGLENKVENEFRIGASDDDELENDLSSNSSEPEAIIEEFFRAKFEDKFPRDLSGCPLTASFRPWEPYIFRNSEEQPVDDYYYGLQGDEDDYNDTSPNYGESDDESYADPGEDGDGAIPDTETQSGGKLKLSGIEYEMVQTIAERLHVSIEMQGENSNLYHLFQQLIDGEIEMIVGGIDEDPSISQFVSSSIPYHQDELTWCVARAKRRHGFFNFVATFNADAGFLIGIFVVTCSLVVWLAQRVSGFQLRNLNGYFPTCLRVLGILLNQAIPAQDFPITLRQLFALSFLMGFFFSNTYQSFLISTLTTPRSSYQIHTLQEIYSNKMTVMGTSEHVRHLNKDGEIFKYIREKFQMCYNLVDCLNDAAQNEHIAVAVSRQHSFYNPRIQRDRLYCFDRRESLYVYLVTMLLPKKYHLLHQINPVIQHIIESGHMQKWARDLDMRRMIHEEITRVREDPFKALTFDQFRGAIAFSGGLLLVASCVFAFELCYVKYVYRTEKRERKTKKITKKVHNIKIQHD  >DmelIr84a  MIKLQVKVISWPLIILTAFLRVLQIESINTNFLELAAFEDFLRSEHLSHVLVVRGDDADGDWKIECHQKLLANYRVQFYRPEMSANFEDLMFYGSPRTAVLVLNSEHVLVRRQVFGVASEAGYFNNSLAWFILGSGRESLPVEQLIDQLLSGYRMGIDADITVALRGPDNASMLFYDVYRISRQANTPLIIEKKGLWTHSGGYQKFGNFKNTWVIRRRNFLNVTLIGSTVLTEKPPGFGDMEYLADDKQLQQLDPMQRKTYQLFQLVERMFNLSLAISLTDKWGELLDNGSWSGVMGQVTSREADFAVCPIRFVLDRQPYVQYSAVLHTQNIHFLFRHPRRSHIKNIFFEPLSNQVWWCVLALVTGSTILLLFHVRLERMLSNMENRFSFVWFTMLETYLQQGPANEIFRLFSTRLLISLSCIFSFMLMQFYGAFIVGSLLSESARSIVNLQALYDSNLAIGMENISYNFPIFTNTSNQLVRDVYVKKICKSGEHNIMSLQQGAERIIQGRFAFHTAIDRMYRLLLELQMDEAEFCDLQEVMFNLPYDSGSVMPKGSPWREHLAHALLHFRATGLLQYNDKKWMVRRPDCSLFKTSQAEVDLEHFAPALFALALAMVASALVFLLELFLHWLPDFRRRLGTMST  >DmelIr7b  MKYWLYILSCCSLVASTMESSSDWDLAEALAQVVANSEMGRFKTLYIYTHTNSQSTGGHLEELLDQVLMIVPNNLQARRLLLQQSMEYKPYVHAVLALVDGLPSLSAIYARIRATQDLSHTLIYMSMPTDAYGEEMQATLRFLWRLSVLNVGVVLRPPGDHILMVSYFPFSALHGCQVISANVVNRYQVGTKRWASQDYFPSKLGNFYGCLLTCATWEDMPYLVWRPDGSGSFVGIEGALLQFMAENLNFTVGLYWMNKEEVLATFDESGRIFDEIFGHHADFSLGGFHFKPSAGSEIPYSQSTYYFMSHIMLVTNLQSAYSAYEKLSFPFTPLLWRAIGLVLILACLLLMLLVRWRHHHELPRNPYYELLVLTMGGNLEDRWVPQRFPSRLVLLTWLFATLVLRSGYQSGMYQLLRQDTQRNPPQTISEVLAQHFTIQLAEVNEARILASLPELRPEQLVYLEGSELQSFPALAQQSGSSARVAILTPYEYFGYFRKVHPMSRRLHLVRERIYTQQLAFYVRRHSHLVGVLNKQIQHAHTHGFLEHWTRQYVSAVDEKDESVARIASTSYSTLDGIDGDPSLSESEEDQQVAPVRQNVLSMRELAALFWLILWANLGAVVVFVLELLLPRIKLRKILRKMKSDIKKQISKLVRK  >DmelIr75d  MKVQVAHWLPLIFFLLVSGTPRVAGSWRSEYSRQDPDPKTRWGNQLPDMLVAYYRHHGVHSLMLVVCHTDIADFRLWKLWQHFNLNNFYVQVSTESSLRDLQHVDALDEHKDAPPPKSFHANNSTHWETSFLLPALPYKMGILLLEFSSECALNLLRWSAASEHNYFTTNRFWLLLTEDPGDIDLLEDPEIFIPPDSELRVLHYENVGNFSCSLIDLYKVAAWKPLKRTLVGHNIRNSRHVIHALQHFGSAITYRQDLEGIVFNSAIVIAFPDLFTNIEDLSLRHIDTISKVNHRLMLELANRLNMSYNTYQTVNYGWRQPNGSFDGLMGRFQRYELDLAQLAIFMRLDRIALVDFVAETYRVRAGIMFRQPPLSAVANIFAMPFENDVWVSILMLLIITTVVLVLELFFSPHNHDMSYMDTLNFVWGAMCQQGFYVEVRNRSARIIVFTTFVAALFLFTSFSANIVALLQSPSDAIQSLSDLGQSPLEIGVQDTQYNKIYFTESTDPVTKNLYHKKIASKGENIYMRPLLGMEKMRTGLFAYQVELQAGYQIVSDTFSEPEKCGLMELEPFQLPMLAIPTRKNFPYKELIRRQLRWQREVSLVNREERKWIPQKPKCEGGVGGFVSIGITECRYALGIFGCGAAVSFVLFLFEFIFRHFKQVYRIIKGYREVQR  >DmelIr76b  MATGIELLVAAALCVACPPLNDSPPTNLIQMGENGTLSPVTELPMDVDASEAGFDADAPVETLETINRKKPKLREMLDWIGGKHLRIATLEDFPLSYTEVLENGTRVGHGVSFQIIDFLKKKFNFTYEVVVPQDNIIGSPSDFDRSLIEMVNSSTVDLAAAFIPSLSDQRSFVYYSTTTLDEGEWIMVMQRPRESASGSGLLAPFEFWVWILILVSLLAVGPIIYALIILRNRLTGDGQQTPYSLGHCAWFVYGALMKQGSTLSPIADSTRLLFATWWIFITILTSFYTANLTAFLTLSKFTLPYNTVNDILTKNKHFVSMRGGGVEYAIRTTNESLSMLNRMIQNNYAVFSDETNDTYNLQNYVEKNGYVFVRDRPAINIMLYRDYLYRKTVSFSDEKVHCPFAMAKEPFLKKKRTFAYPIGSNLSQLFDPELLHLVESGIVKHLSKRNLPSAEICPQDLGGTERQLRNGDLMMTYYIMLAGFATALAVFSTELMFRYVNSRQEANKWARHGIGRTPNGQSVAPSRWLRGWRRLNSGHGQLLGASTHGQNVTPPPPYQSIFNGGSHGDPLNRWRRPLANGNALGNGVLLGGDSEGGVRRLINGRDYMVFRNPNGQSQLVPVRSPSAALFQYSYTE  >DmelIr75c  MTSWPLYRLIVFNLLEINLSNLMVFHCWSIKEAFPLVEMLNQNGIFSQYIDVQNPDNLANVHKEYLDSDLVRLGVFLDLGCDKAELVTNQSSRARLYNQNLHWLLYDEAGNFTKLTQLFEGANLSLNADVTYVSREDEERFILHDVYNKGSHLGGKLNITVDQTLQCNRSHCQVKEYLSELHLRPRLQHRMDLSSVTFRLAALVSVLPINSSEEELLEFLNSDRDSHMDSISRIGNRLIMHTQEILGFKLHYIWCGTWSVQDAFGGAIGMLTNESAELCTTPFVPSWNRLHYLHPMTEQAQFRAVCMFRTPHNAGIKAAVFLEPFMPSVWFAFAGLLIFAGVLLWMIFHLERHWMQRCLDFIPSLLSSCLISFGAACIQGSYLMPKSAGGRLAFIAVMLTSFLMYNYYTSIVVSTLLGSPVRSNIRTIQQLADSSLDVGFDTVPFTKTYLVSSPRPDIRSLYKQKVESKRDPNSVWLSPEEGVIRVRDQPGFVYTSEASFMYHFVEKHYLPREISDLNEIILRPESAVYGMVHLNSTYRQLLTQLQVRMLETGITSKQSRFFSKTKLHTFSNSFVIQVGMEYAAPLFISLLVAYFLALLILILEICWARYAKKKFSTIIPQNQ  >DmelIr75b  MLQLHNLILHNLIHMAKLSHVLILHCSLSHLALLAQSKNIFTQFQPLHSDIQLNDDFLNHNILKLGVFLDINCDKSGTVLDMASAKRFFSHRYHWLIYDRSMNFSVLESHFKEAQIFVDADVTYVTHDPFSKNFLLYDVYNKGRQLGGELNITADREIFCNKTNCRVERYLSELYTRSALQHRKSFTGLTMRATAVVTALPLNVSIKEIFDFMNSKYRIQLDTYARLGYQARQPLRDMLDCKFKYIFRDRWSDGNATGGMIGDLILDKADLAIAPFIYSFDRALFLQPITKFSVFREICMFRNPRSVSAGLSATEFLQPFSGGVWLTFALLLLLAGCLLWVTFILERRKQWKPSLLTSCLLSFGAGCIQGAWLTPRSMGGRMAFFALMVTSYLMYNYYTSIVVSKLLGQPIKSNIRTLQQLADSNLDVGIEPTVYTRIYVETSEEPDVRDLYRKKVLGSKRSPDKIWIPTEAGVLSVRDQEGFVYITGVATGYEFVRKHFLAHQICELNEIPLRDASHTHTVLAKRSPYAELIKLSELRMLETGVHFKHERSWMETKLHCYQHNHTVAVGLEYAAPLFIILLGAIILCMGILGLEVIWHRHCTLH  >DmelIr60d  MRLAIYVAFLSSIGNRSGFLSSLLMSLGKELHYKTILLVGGSSTCWSLEPFETGVPILNLRGENNAYPQDTFNSQMLALACLQTESEDAVKLLYRSLKDMRDTPTLLFASSEEHIHDTLFLGCFRENMLNVLALTASSKEFIYSYQAFPTFRVIKRKLVEIHRYFEPQLKDLGGHIVSALPGNIMPRTMCYRNAEGERQLAGYLNTFIRNYVESINGTLRISWGLVPEDDMRHLTISRLSKIQHVDFPLGIIPLYNKTDKQHVYMEISSWFLMLPMETSVPRAHLFVKLGLERLLPIIVVVGAVLGNAHRIEVGLGPSWRCYYLADKVLRGALAQPIVLPRRLSPKLMLIYSLLLLSGFFLSNYYMASLTTWLVHPPASDRILEWDQLRYLHLKVLTIPEEFKYMSLILGTDFMTAYGSIFQLTNSTDFQRRRISMDPSYAYPVTTSLWPFLELSQVRLRRPLFRRSYDMVLQPFQVMSLPLPRNSIFHKSLLRYAALTRETGLYYYWFRRSYYELVALGKISYKEEEGNPYCDLKWNDFRIVWLAFLGGTIISCLALLLEVAHYRWHLGNSSL  >DmelIr94h  MLSNISFSSAPELVDLYGLVLKFLVSSETTLFYFNPTGQKCSWETLPRTILSNHPQIIWFREETYPGLYKRHSSNLFVMACLSSTSYDGQLQLLAESLTRYRSVRVLIEVQDKEGSFLASQILLLCQQHSMLNVVLYFSRWTRTLNVFSYLAFPYFKLLKQRLSGSLRPKIFINQLKDLQGYKIRVQPDLSPPNSFSYRDRHGECQVGGFLWRIVENFSKSLKGDTQVLYPTWAKAKVSAAEYMIQFTRNGSSDIGVTTTMITFKHEERYRDYSYPMYDISWCTMLPVEKPLSVEILFSHVLSPGSALLLILAFILFFLIVPQLIKCLGITFRGRLIGMASRIFALVMLCSSSAQLLSLLMSPPLHTRIKSFDDLLTSGLKIFGIRSELYFLDGGFRAKYASAFHLTENPNELYDNRNYFNTSWAYTITSVKWNVIEAQQRHFAHPVFRYSTDLCFSSETPWGLLIAPESFYREPLQHFTLKINQAGLITQWMTQSFHEMVRAGRMTIKDYSRTNLMKPLRIQDLRKCWVIFAVGLGTSTVVFTIELLLIYTNVFLNSL  >DmelIr94g  MSTAVNSVHSKLVSLISRGQELTSIFFYAPAKEKCHLEDTISSATWGLPLVIWRTDRTVILNGFIGEGLLVLACLPGFHWRALLGSLARSLKYLRQARILIELMQDRDEFLVSEVLQFCLSQDMINVNAIFDDFPETENLSSFEAYPSFEVVNQTFTPDTQVSDLYPNKMLNLRGGVIRTMPDYSEPNTILYQDKEGNKEILGYLWDLLEAYAHKHNAQLQVVNKYADDRPLNFIELLDAAQSGIIDVGASIQPMSMGSLSRMHEMSYPVNQASWCTMLPVERQLHVSELLTRVIPYPTLALLLLLWIFYEVLRGRWRRHSRLQSIGWLVLATLVSSNYVGKLLNLFTDPPSLPPVNSLAALMESPVRIISIRSEYSAIEFTQRTKYSAAFHLALHASILIGLRNAFNTSYGYTITSEKWKIYEEQQKRSSKPVFRYSKDLCFYEMIPFGLVIPENSPHRAPLHSYTLLLRQAGLHDFWVNRGFSYMVKAGKINFTAVGERYEAKTLTITDLRNVFIIYVSVLLISLILFTCELFVSWVNYWLGF  >DmelIr7a  MFHHLWLLMGLRSLAMGALHPPQPEAMTPLVAAALEILAEQVSPSQSTLAVMDLTQDAEHRDERQEQLMTIILRSVGSEMALRTFQKPPAEVPASFVVFLVNSAQAFNTLGFHFTDIHSTREFNFLILLTHRMSSRAERLQVLRDISRTCVRFHTSNVILLTEKRDGVVLVYAYRLLNMDCDLSVNLELIDIYKNGLFRHGHEARSFNRVLSLSGCPLQVSWYPLPPFVSFIGNSSDPEERAQIWRLTGIDGELIKLLASIFDFRILLEEPCNKCLSPDIKDDCSGCFDQVIISNSSILIGAMSGSHQHRSHFSFTSSYHQSSLVFIMHMSSQFGAVAQLAVPFTVIVWLALVVSSLLLVLVLWMRNRLVCGRSDLASHALQVLTTLMGNPLEARSLPRSSRLRILYAGWLLLVLVLRVVYQGKLFDSFRLPYHKPLPTEISELIRSNYTLINQEYLDYYPRELTVLTRNGSKDRFDYIQGLGKEGKFTTTSLIATMEYYNMMHWSTSRLTHIKEHIFLYQMVIYLRRHSLLKFAFDRKIKQLLSAGIIGYFVREFDACQYRKPFEEDYEVTPIPLDSFCGLYYISLIWLSAAVVAFILELLSQRIVWLRRIFE  >DmelIr7d  MDIRCVVALLLGLCKVQAVVWPHQHLLEEQLASQISATLQKIFINGLAVYNFGVFISTSYEEMDRDRVILVHQVLNRNLYPPNFPVAVVLASKMNRKITAQVFTQLLFVQNAEQAIAIAEGVNRNGLCVIVLLTSQPERPIMTKIFTYFMQERYNINVVILVPRLHGVQAFNVRPYTPTSCSSLEPVEIDIKDGDLWDVFPRRLKNLHGCPLSVIVWDIPPYMRINWKSSDPMDGLDGLDGLLLRIVARKMNFTLKLIPNEPNGLIGGSSFMNGTFTGAYKMLRERRANITIGCAACTPERSTFLEATSPYSQMSYIIVLQARGGYSIYEVMLFPFEKYTWLLLSTILGLHWIVGSRWRMPSPILAGWMLWIFVIRASYEASVFNFIQNSPVKPSPRTLDQALSGGFRFITDHASYRMTLKIPSFQGKTLISAGQPVDVFDALLKAPWKTGAFTSRAFLADHLVRHRKHRNQLVILAEKIVDNMLCMYFPHGSYFAWEINKLLFNMRSFGIFQHHSQILAWDNLPTTTDTDTPGKRIHSSTESVATGFAESMSFVVAALNCLMGALCISIVVFGLELLSRRRHWTGLEWLFERV  >DmelIr7e  MNHINEFVARAVLHVVHHYILSVTPSLVLTLCCRSNHTCNFYNKMMSTLFREWGLAPLQIVNVLRGVPWHPVPGRRHFNVIFTDSFAAFEEIRMEYYSREYNYNEHYFIFLQARDRLLQGEMRLIFDYCWRYRLIHCSIQVQKSNGDILFYSYYPFGEHGCSDMEPQLINRYNGSMLVEPDLFPRKLRNFFGCPLRCALWDVPPFLTLDEDQEEVLRVNGGYEGRLLLALAEKMNFTIAVRKVHVNMRDEALEMLRRDEVDLTLGGIRQTVARGMVATSSHNYHQTREVFGVLASSYELSSFDILFYPYRLQIWMGILGVVALSALIQLIVGRMLRERMGSRFWLNLELVFVGMPLLECPRSHTARLYCVMLMMYTLIIRTIYQGLLYHLIRTHQLNRWPQTIESLVQKNFTVVLTPIVQEVLDEIPSVQHMRFRLLEANSELDPLYFLEANHQLRQHVTASALDIFIHFNRLSADKVHQRGEQGSGAHFEIVPEDIISMQLTMYLAKHSFLIDQLNEEIMWMRSVGLLSVWSRWELSESYLRNEQSFQVLGTMELYAIFLMVLVGLIVGLLVFILELVSMRSIYLRKLFT  >DmelIr7f  MQGEDANLYVARALRLVIENVLAQLSTTLVVTISTRHLGTAHWFEYMMNILMDSWRMVAVQLLRIRPDLVVNPVPGRKRVSLLMVDSYQGLLDTNITASNANFDDPDYYFIFLQARDHLIPKELQLILDHCLAHFWLHCNVMIQTAQVEVLVYTYYPYTADACQKAYPIPVNTFDGRKWKASQMFPDKLSQMHGCPLTVLTWHQPPFVELVWDPKHNRSRGSGFEIQLVEHLARRMNFSLELVNIALLRPNAYRLAEGSSEGPIEKLLQRNVNISMGYFRKTARRNQLLTTPMSYYSANLVAVLQLERYRIGSLALLVFPFELSVWMLLLLALLIHLGIHLPSARRGNEEDGGGGLQVVALLLGAALARLPRSWRHRFIAAHWLWASIPLRISYQSLLFHLIRLQLYNTPSFSLDQLLAEGFQGICTANTQRLLLEMPQLARDPDSIQSVDTPFDWDVLNVLTRNRNRKIFAVANQDVTLSFLHSSAHPNAFHVVKQPVNVEYAGMYMPKHSFLYEKMDDDIRRLDASGFIHAWRRASFASVHRKEQVHMTSRRYINHAKLSGIYMVMAGLYLLAGLLFAGEVLLRQRN  >DmelIr7g  MNVTSLLNFESMKYIGAQTQAASINHHVAQALRVFIEDFYQRIAPAFIVVLSCRRPSPMNFYRNIMQLLYESVDTMIVQLVLVELGRPRRIAGPRTHNLLLVDSLDALLDIEIHTYTAQSDTSEYYFIFLQQRDALIPHDMQGVFAYCWRHQLINCNVMTQSSGGQVLLHTYFPYAPGQCNDSQPTRINMFLGESWKHRDYFPSKLHNLNGCPLIVLARKVSPFLDLDEGQRELRGLEGRLLQELSRRMNFSIQFSGLQDQLKNRTTWTEKQLLQKLVQERIAHLAIGYVRKRIQYATNLTPVFPHYSNRVVGCLLLNAHNLTSLEIWSFPFQALTWICLVAGDRLALVLAVYAASLGLPIDPPERPSLQLLFASWLIFGLIVRSMYSALLFFILRYHLHQRLPGNLQDLTHGDYAAVMGRTTLQDLREVPSLQDLLGLKSVIVTSEREEEVLRTLDRCTLREGAGSHPLFFGLISQDALLHLTQRGHRAGAYHIIPQDVLEQQLAIYLQKHSHLASHLDHLVMSIRSVGLVHHWAGQMASERYFRSRFLYREKRIRQPDLWAVYILTAGLYLLSLVVFICELLASRRAGL  >DmelNmdar2  MMPSRVKLKRGTDGPTPTPTPMPTTMRKHTPIATLNTASCQHNSTTSRRKRILTPPSGPISLLLLTVLTLLILDTRSCQGLRLTNGGGSLSKGAAANKEQLNIGLIAPHTNFGKREYLRSINNAVTGLTKTRGAKLTFLKDYSFEQKNIHFDMMSLTPSPTAILSTLCKEFLRVNVSAILYMMNNEQFGHSTASAQYFLQLAGYLGIPVISWNADNSGLERRASQSTLQLQLAPSIEHQSAAMLSILERYKWHQFSVVTSQIAGHDDFVQAVRERVAEMQEHFKFTILNSIVVTRTSDLMELVNSEARVMLLYATQTEAITILRAAEEMKLTGENYVWVVSQSVIEKKDAHSQFPVGMLGVHFDTSSAALMNEISNAIKIYSYGVEAYLTDPANRDRRLTTQSLSCEDEGRGRWDNGEIFFKYLRNVSIEGDLNKPNIEFTADGDLRSAELKIMNLRPSANNKNLVWEEIGVWKSWETQKLDIRDIAWPGNSHAPPQGVPEKFHLKITFLEEAPYINLSPADPVSGKCLMDRGVLCRVAADHEMAADIDVGQAHRNESFYQCCSGFCIDLLEKFAEELGFTYELVRVEDGKWGTLENGKWNGLIADLVNRKTDMVLTSLMINTEREAVVDFSEPFMETGIAIVVAKRTGIISPTAFLEPFDTASWMLVGIVAIQAATFMIFLFEWLSPSGYDMKLYLQNTNVTPYRFSLFRTYWLVWAVLFQAAVHVDSPRGFTSRFMTNVWALFAVVFLAIYTANLAAFMITREEFHEFSGLNDSRLVHPFSHKPSFKFGTIPYSHTDSTIHKYFNVMHNYMRQYNKTSVADGVAAVLNGNLDSFIYDGTVLDYLVAQDEDCRLMTVGSWYAMTGYGLAFSRNSKYVQMFNKRLLEFRANGDLERLRRYWMTGTCRPGKQEHKSSDPLALEQFLSAFLLLMAGILLAALLLLLEHVYFKYIRKRLAKKDGGHCCALISLSMGKSLTFRGAVFEATEILKKHRCNDPICDTHLWKVKHELDMSRLRVRQLEKVMDKHGIKAPQLRLASSSDLLNHHHLKERPPLLGNLSLAASAQDLYRWSYKTEIAEMETVL  >DmelNmdar1  MAMAEFVFCRPLFGLAIVLLVAPIDAAQRHTASDNPSTYNIGGVLSNSDSEEHFSTTIKHLNFDQQYVPRKVTYYDKTIRMDKNPIKTVFNVCDKLIENRVYAVVVSHEQTSGDLSPAAVSYTSGFYSIPVIGISSRDAAFSDKNIHVSFLRTVPPYYHQADVWLEMLSHFAYTKVIIIHSSDTDGRAILGRFQTTSQTYYDDVDVRATVELIVEFEPKLESFTEHLIDMKTAQSRVYLMYASTEDAQVIFRDAGEYNMTGEGHVWIVTEQALFSNNTPDGVLGLQLEHAHSDKGHIRDSVYVLASAIKEMISNETIAEAPKDCGDSAVNWESGKRLFQYLKSRNITGETGQVAFDDNGDRIYAGYDVINIREQQKKHVVGKFSYDSMRAKMRMRINDSEIIWPGKQRRKPEGIMIPTHLRLLTIEEKPFVYVRRMGDDEFRCEPDERPCPLFNNSDATANEFCCRGYCIDLLIELSKRINFTYDLALSPDGQFGHYILRNNTGAMTLRKEWTGLIGELVNERADMIVAPLTINPERAEYIEFSKPFKYQGITILEKKPSRSSTLVSFLQPFSNTLWILVMVSVHVVALVLYLLDRFSPFGRFKLSHSDSNEEKALNLSSAVWFAWGVLLNSGIGEGTPRSFSARVLGMVWAGFAMIIVASYTANLAAFLVLERPKTKLSGINDARLRNTMENLTCATVKGSSVDMYFRRQVELSNMYRTMEANNYATAEQAIQDVKKGKLMAFIWDSSRLEYEASKDCELVTAGELFGRSGYGIGLQKGSPWTDAVTLAILEFHESGFMEKLDKQWIFHGHVQQNCELFEKTPNTLGLKNMAGVFILVGVGIAGGVGLIIIEVIYKKHQVKKQKRLDIARHAADKWRGTIEKRKTIRASLAMQRQYNVGLNSTHAPGTISLAVDKRRYPRLGQRLGPERAWPGDAADVLRIRRPYELGNPGQSPKVMAANQPGMPMPMLGKTRPQQSVLPPRYSPGYTSDVSHLVV  >AgosIr1-C  MENLLSAIRRSNLMSRNVVYVFLWLRSPVSRTFKTDILEAMRVCVITSPRPGFYQIYYSQASARPGYGSTLKMVNWWSAMDGLVRFPLLPPPKRVYKNFEGRYFNVPVLHKPPWTFVEYLNDSFRVEGGRDDKLINLLADKLHFHYQIYYSQASARPGYGSTLKMVNWWSAMDGLVRFPLLPPPKRVYKNFEGRYFNVPVLHKPPWTFVEYLNDSFRVEGGRDDKLINLLADKLHFQFKYIDPPDRTQGSGLDQGSSMQGVLGLIWQREADWFVGDLSITYERNLVVDFSFLTLVDNEAFLTHAPGRLNEAFSLIRPFHWSVWPLLLITVIFSGPILYILVDTTDGHPQGKSMLYWKCVWWSVTVFLQQGNVLH  >AgosIr2-N  MNLLIASAGPALQEKNKNEVADIKLYTHEMYIDGLGSSVQIILTTWKLNRFTRPEVNLYPVKLVNGFWGHRFIVSAIEKPPLVFRSIDKILTQEQSILWDGIEIRLIQLLAGILNFTLEIHDATLSKSRDESDDRIIGDLVTSKAELGISGLYMTNARYSMVDFSPVIMQDCGTFMSLGSFALSKYRAIFGPFHWSIWVMVVITYMAAIFPIAFTNNRNVKTLCKSPKQLESMCCYMFGTYTNLFTFKDVKSWTNTKMGSTRLFIGTYWIFTIIITTAYTSSIIAFITLPAQPVIVDSSYQLVDQRYRVMTLDKGGWQYYLNITNDTMSKRLISNIKLMNNLEDAIDYIVRTRFILDYAFLGSKISLTYLYQNNYIQKYKNKKIFLHVASECYVPFNIGVAYKKHFLFRNIFNNFILRAQQSGLITKIIKDIEWEIIQKSGVRNPNLIIAPEDRQLALDDVQGMFVLLGGGILLATFTLIIEYVKRKREKRKITQIKIKNHQKKKKRSKSLTVAENTIAESIRPLTTF  >AgosIr3-C  MFCIQFSRGVYSMLGSVNPDSFDTLHSYSNTFQMPFVTPWFPEQVLSPSSGMLDYAVSLRPDYHRAILDTVRYYGWTNIIYMYDSHDGLLRLQQLYEALDLGPNSLKVDMVKRIQNVSDALNFIHQVERVNRWGNKRIVLDCPTLMAKQIVVSHVKDIQLGKRTYHYLLSGLIMDDHWETEVIEYGAINITGFRLLDMNQWSVKHFLSEWKNLDPNTSPGAGKDTISAQAALMHDAVLVLVETFDKLLWKKPDMFKMNAKRTLSNGNSSMSGISSSQILGLDCNNGRTSGNQWEHGEKISRFLRKVVMEGLTGHIMFNDDGKRYNYTLHVVQMTIDSTITKIAEWSDTDGFKTVVSKPERVHTSGHSHKGNSTLIVATVMEEPFIMFKKPKYGESLAGNDRFEGYCKDLTTLLADKIGVK  >AgosIR4-F  MALTNTLLLALCANWPPLNITETTLLPPWKWISEHKAKYINSSEYQCDLKNNFQIQYEILRGKRLKIATFPNSKPLSWVTKEINGSLIGHGIAFEIVETLRQRYGFTYDVVVPTRETLLNENGSIIYMLVNGEVDMAAAFIPVLPGLDDIVKWGIELTQFQYVVLMKRPKESATGSGLLAPFEMEVWFLILMSLIAVGPIIYGIMMLRHKLCGHESGVYPIPTCIWFVYGALMKQGSSLNPDTDSARLIFATWWIFIMILTSFYTANLTAFLTLSKFTLPIKRIEDIASNEYRWISSEGSAVEYIVKVDNDLKPLRQSMFEGNGQFMVINIDEIDTILKYISSGTLLLHDKNWLNFLMLHIERSITDDKERCRYVLTTDPYLTRSMSFVYPKHSILPPLFNPIRMLSYMESGIVRHLQTKDLPEAVICPLNLGSKERQLRNSDLFTTYAVVVCGFSMAAAVFTLELLSRRTGWFATTDLSSRPHDPHSGVVRCEIAVFGKPSKTMYPFSHRHNVMNAASATHFVFHGSSSSSPSRSFVNARSKIASDSSSSSSSPFLWLEGKSSLRRFHY  >AgosIR5-F  MHQNYKPALLLNEFHVKLFNPNRYILPHAYLVAANNTTDLVDHLDTINVTDASWQPEVNFIIILEKFTELSQHILEPVFQRLWIKNMFKSVLLIPAIDGNAIEAFYWYPFHKRCGEYHTPTLEDLCTRGDNNNSQWTVFDVFEKIIPNTFYNCTVNIVGFNWAPMTLLSNQTPRTMLHGMDVEVVKLMSRIGKVQLEFHEVENNQRWGVKLENGTWNGGFGKLSGHRGDFLIGGGILTAERKEMFDSAPARQVIRFPIYTPLPRKLPYWQNMLNVFSGNFWLTLFVVFFLTSGLLWLSGIHLPSEKRAFSNCGYCLVISWAILCSVASGQQPTSVSSKMIFLSWVIYMLHISAVYTSMQLIYIYKPKYEPPMRTVNDVKESGLTICSVPTFIPIAHSMDKDNFNLTEYIPCMDNHFEALISGSIKKVNKVEEVVIVYNIGIFMQKGNPYKNILSKAQIIAYETGLHDKWRQDASPSRPIKKKGNIKVKKLSVDELQGAFIILICGLGMSSVIFVFEWLFGSK  >AgosIR6-F  MDYINVVLLFLVTYTISTSQLDSNFDAQYKLMDQFLTIKNLDTAHLYTCWSQSDRIKAMKKFNEKKIAVTYRNKEDFYGNFNLNDLFTITVYSPLLGIIVDWSCNDDPLFKFSASWLWSASYHWLMMMSEKSNNIQSFLTSKNVNLTISSEILLAYPIMMDGVITNWHIFDIYRTAFEPRGKLMIDVVNKTLGMWGNRLWKFDKRSNLENLTLNVVTVVRTVMEPTKLSNRLIVLLMFVFSLVTYQFYSSSIVSGLLRPTVMNIDSVQKLEESGLDVGVEDFNVITKVIEVYGEMNPFLKKIIDSKIKPKSEYLLAKDGVKKIKKGHYAFFTDPATSYWLINDIFTEKEKCDLSELALHRPETTGYLVQKNSPYRKLINYANCILWETGLMQRELSIWHAEKPKCTAGKTTTEESLVSVEIKDVASLIAFLIIGFIGSFAILVLEIIIHRHKTHTTAILVKNN  >AgosIr7-F  MNCSYGEIKWNSEFENLAVDITYKWKDTATCLNLILDHYHDGILDKGFYTAIAGIPLFKTLVDDSEDLMSPNFQTWQILNNVRKQGCNMNIIFILNADQTMRLLKFSDKKYRLKEMYELSTVPYPAPIKGTLVTLRLDIWNKRNFQKKTDLYIDKVSDLQGNLLKVVTFNYIPSAIKNPLMNENDENSGYKKGLEIEVLRTLGSAMNFIPVIYEPLNWRTEQWGKKQINGTISGLLGEVWSARADLALGNLHYTPYHLNILDLSIPYNTECLTFLTFESKTDNSWKTLILPFKLNMWVGVLITLLVGGFLFYAFATAHKHIEDNENLIKMIQCDIQKTKILENKPELLTENKTIIKNIDIIKHTVKYPKIIKEQKCINEQTKNNQFNNTDVTGLYLFENIENSILYTYGMLVAVSLPKVPSGWAIRILTGWWWIYCLLVVVAYKASMTAILANPDTRVTIDTLDALAESNINCGGWGEQSKEFFMTSLDKTGQKVGQKFQEVYEVDKAIDLVSKGQFAYYDNIHFLRYVKVMQNTKTYEQNVQLINGTRNDTSNGDFTLHIMSTCIINMPISLGLQKNSPLKPAVDRFLRRVIEAGLVKKWLNDVMSDTVILEEPQQIEEVKALMDLKKLYGALVVLVAGYILSILVLLIEIGYWYGVVKKDPLYDEYSLNCYYSQK  >AgosIr8-F  MRTYAGVLTQNENDTNFIQLKSILTNYDNISIEPILNDQPNNITDKFCNVASNNTLAIIDLLNPSCTTCWKISNANAMAYFRTDFSYIQPAIQLIESYVTWLNITKEITFVFTNQEDADQAVTYLTSGKSSLRAIVLNKLTSNEIDQLKNTKIGIRHVALIGNNLDSYVKTINQEKLIKLDESWIIVTNDTSKYKLEAAVTLMKFTSWENGHTNKTTRARTLFNFIFYFLGRVSRDRLKLNCNMNSDMLVLGKRKETEDILNSYEYKNEFHYDIDTKLMTYNEQATILKFSPDGVPNQLGTWTINGGLEMKYDALTVVSGRRFFRVGTAKSIPWTFMEDEWKGYCIDLIEKLSIEMNFKYELVIKDQFGELDPVTNKWNGLIGGLVDGELDIVIAALTMTSEREEVIDFIAPYFEQTGISIVIRKPSRKTSLFKFMTVLKPEVWLSIVAALALTAFMIWILDKYSPYSAQNNKPKYEQFRHFTLVESFWFALTSFTPQGGGETPKAISGRVLVAAYWVFVVLMLATFTANLAAFLTVERMQTPVQSLQQLARQSRINYSVIDGSDAHQFFRNMKMAEDILYKQCWVLEVSEKLKMPSSVWKEIALNQTNNRKDFRVWDYPIKEEYGQILAAIERTGTVPNRTVGYQMVTVWKEIALNQTNNRKDFRVWDYPIKEEYGQILAAIERTKSDIEYEVYNNCNLTEVGEIFAERPYSIAVQQGSLIQEEISRKILDLQKDRFFELLNAKYWNASKTSTCPNSDDSEGITLESLGGVFIATLVGLLIALITLAFEVVYFKHKRAKIAEVSVVNNTIHKDKLLYGHELFMTLGRNSNSDDQTRWANKIKLDSTTGRLNNALFFRRNKFQN  >AgosIr9-F  MKRSAGAVVAVVALLQAAAAVGGPTTRRRLRDLALDDDAHRIRLSRLAGSVNKIIDEYLGTRSVVMFADEVDVSELGQRLLVDMGHPRLLVQSSAATDARLANGLVVYLEPEARAQRRDGRSADYDAVLGRLPSSDHSRHMVLWEGSQRPGDRVDLHRIQMLFEAFWHHQLVDVAVLVPVYTGSIRVYSFNPYTGSRCNGAGPPIMVNVWSSSTDAFVRPDRVFGLDNKLKDLHKCPLKCLGIHRPPVSTVVQTEKGFKLSGSGLRIINFIQEYMNFTGIVTMAVGHSGVHFVSETALSDNDSSPIGVKVKHKKVDLAFGRFTRIFDSESDVEFIKEDQMECFTWGLPSGIGHDPTLWINYVAEFSLVTWILIIMSIILAFGVVVTLSQLTSMLKQPAATISWSPLFILFYTYGTFIGAPIKVTPKSCALQVFLSNWLLYSLVVTSAYQAYLGSLITIPQTIPEINDQHTLLKTDLNLVGRQDMYYLINSSAGSSNDFKELVDRYQILPPEDFSHFIQRILLKRDTAVLASKRELIFYAQRYKTIFNDSRHLHVLPTCTIESYSSTFMLRRGSPFRHRISTIMSRLSETGIMQQWDRENLREEQVHNIVFTEDTILSMSQSFGAFVVLFFGLFLGFCAFIGEIIVYLINKFCIRHTKQIMFIN  >AgosIR10-F  MRNSVAFWFYLVASSFKTSAEYDGLINALTRVFIQKHVLTVTAITCWPKDANSRLLLALSSADISVNFRPSMIPVHETWYRYGILVDVSCVQVTTTLFQKISEPNKFKGFYPFADTDLDIFAQMHYDLNMILQDQLNFKIDLGIVDSFGWNLGNGSFSGLTGLLQREEIDFGGIGSFIRNDRMNAIDYTVGTFYRQPAALFKQPPLSSVHNICILPFKFEVWMVTLFTFIGFTILIAFLSRTTRRFKKDEKESLNVLDSVTIVHGAICQQGYTMNLNAGSIRVAIFVLFLTAVFLFTSYSASIVALLQSPSNSIKTINDLVESSMTFSAQVTPYSRVYFDETDDPLLRKLYDKKMKSHEKDMYTEASTGIARIRTEFHGFLIEVVSAYKLISQLWREEEKCGISEIQLFKLPILALAVVKRSGYKDILKQKEESDFGAAGSLMRLDRMTAVDFTVGTVSLESNILFKQPMLSSVTNIHIEPFKYEVWQVTLVMLMGFVLILLFLNKFKANRRTSLNVLEIIGLVHGAICQQGSTIVLVLNSIKIVVFVLFSTTFFLFNAYSASIVSLLQSTSSSINNVKDLLHEKSMTMSIQIATYAKPYFNVEKTSAYQIINRKWREEEKCGLYEIQLFKLPVLAIPVVKKSGYKDVFKQKLIQQHEVGIKKRLIQRWTPKKPTCDSAKKNRNYVSVSIKEILPTIILFGYGLLISLTVLVLELAYYYGMHYFMKRFKKIKK  >AgosIr12-F  MDKFVIFIIFMVFKVANSADYNEMLNVLKIFFVRRHVQTVTAAITCWPFDVNKKLLDDLSTADISVSFNSHTLQYYSTWYRCAFIIDLSCENSTEILQQISNDRLFNTQNDWILFDQNSFANETNSAQFALRTFQLYLGNAYVLPDASVFLFLKTYDGVWEIWSGFRASKSDAIRVFEIGTASLNRMTIREMHDEKRNFRGVTLKSTSVIVEKDSFFGFDKKISSDLDVFAHMHYEMIVTLTNQLNFKTEITIVNDYGWYLGNGSFGGLIGLLQNEAIDFSATGVLIRTDRMSVIDFAVGIVELRTAAMFRQPSLSSVHNILLLPFTTDVWIGFNSTLTLISIRVVIAVLFFTSVFFYTSYSASIAALVQSNSNSIKSIKDIVESSMTFSTQISPYGKRYFEETEDSDLKKLYKTKMVPYGNKAFVKTAEGMERIRTEFHGFMVEIMSAYQTISKEWREEEKCALGEIQLFKLPLLSIALVKKSGHKDIFKQKLIQQMEIGLSKRISNQWIPPKPSCGSSSRTKQYVSVSVKETFLTLAVFGFGICISLASNRRLFSLQNDWVLFNRHGNPNKTDALEIALHSFKTHMNQAYVMPDSRVYLLIEVNRNLWEIWDGFRLSVAKEVQVSRVGVMTPERVEIKLSDRTNFKRVVLSASTVVSPM  >AgosIr13-F  MYYGFRRFDEHGYTRAWKKVGTIGPSSTGGQLVKLDSVVWAGGKMVPASDLGRRAVYRVVTAIGPPFVMHAPLQQDRQCLRGIQCYQMTTTNKDNITMILKDVKLDSKKNATLPNTYCCFGLSIDLLEKMSKDLEFDFHLYLVADGTFGSQKIHWNGVVGELVSGTAHMAFCPLSVTSTRSKWVDFSTPYFYSGVSMMVAPKRKTNVPLLAFLLPLSPSLWIAIFVSLHVTTVAVALYEWFSPFGLNPSGRQRSKNFGMPSALWAMWGLLCGALVNFKAPKSWPNKFLINMLLMKVGSAKSSSAEVYLKDKNPPLWQHVQKYSVPDTASGMRMLRNGSLDIFIGDKPILDYYSGTDHDCKLQTHGDPLYDDVYAVGMTKNFILKEKVSAAVSTYINNGFMDILQNKWFSDLPCVNRQLETSDIGQPTPLGVDAFLGVFLMLGFGILGGAIILCLEHAFYRYALPILRQKPPDSMWRNPNIMFVSQKLHRFINNVELISPQHTAKELVHTLRQGQIASLFQKSVRRVSLPFMHPQHAQKEFERRKRKGQFFEVIEEIRRIQREERDNKKQNVRLTLSNSPTAGQSPKRTTSKFLSPARLSLGRRYSKQRSRSSGNLSIRRYSTDVASYSETVGRRLSQGASNSPPDFNTRKQLIRRSSGNPSPADSSRMSIFSASELIGAKLSNNNLYVENDLPRSPNLLSPGVFFRSSFSSDTSSSRPDLGIQSRKSSYSQGPPRVVINGEQSTRRKSDDEPPATLPRIMEVKRARRSSDGHRPTKNRKTVEVFRWLRQAPKTELEALSKMPEDEIKHCIIQALKDKDPT  >AgosIr14-F  MVSIFIVSYCEDIWDLYYKAEFEGLNGLLYISITDTNCPRLPTDEAITIPLTTHDSELSQMILDLRMSNAFSWRSAILMHDNSIGDSIVQHIVTSLTKHYPSNILSPSVAIFEIYTQGSEWKRRKLFMEELEHFLKMSEINSNFICIVSIHYVPLILDVAKSLNLMTAENSWLIIIPDIESSRSNVSSFTSLLSEGENISFIYNSTKTDSKCVFRLNESGVCETCPMWHIDSGVTWGQEYFGHGCYILPVGYWNTKTGLKLTEPLFLAFGECYNVIVISNSDQTNATRTLFTQNNILDEHDAVVSKPIWDKMIDLIRSEKTWLCIAIVVGLMGPILNVFHVLSPYYEYHNITRKGGLNSPLNCFWYVYGALLQQGGAHLPDADSGRLVVGTWWLFVLVIVTTYSGNLVAYLTFPQMDTMVSNVADLMARKPQGYSWGIPKSSNLHSLLTSLPDDTMVKELIKNAEHHEGLSRPVIERVRSGKHAFIHRRTNLMYIMKNDFFKTNRCDFAIGNEDFAEEKLAMMLAKESPYLSRINKEIEKMHKVGLINKWLVDTLPKKDQCWTNTQLEVTNHKVNLDDMQGSFIVLLLGVLSSLVSFLFEYMLDKYINRRQIVITPFIN  >AgosIR25-F  MYKQWRNLDGEQQKYLIQISPPADLIPEIVRSIVVAQNITNAGIMFDDTFVMDHKYKSLLQNIPTRHIIAAIDDTTSIKLHLTRFRDVDIVNFFVLGKLSIIKSVLDHANSNKLFGRKYAWHVITQDKGSLKCGCSNATILFVKPEPDAGSREKLSNLRTTYGLTSTPELKAAFYFDFYYRSLLAIRSMMNSGEWPTNVTYTTCDEYNEENPLPRRNVDLRRYLKDMTEPPSYAPFLIDTNGHSYEEFTMRLEKVTVLNSQSVSAENVGSWKASLNSPIVVKDAANMTHFSAVTVYRVVTVLQNPFMIQVDDEDGKGVKFKGYCIDLIEEIRKLIGFEYEIYIAPDNNFGNMDENGQWNGMVKELVEKRADIALGSLSVMAERENVVDFTVPYYDLVGITILMKKPQTPTSLFKFLTVLENDVWMCILGAYFFTSFLMWIFDRWSPYSYQNNRNKYKDDEEKREFNLKECLWFCMTSLTPQGGGEAPKNLSGRLVAATWWLFGFIIIASYTANLAAFLTVSRLDTPVESLDDLSKQYKIQYAPLNGSSAMTYFQRMADIETRFYELSINIFLNINRIWKDMSLNDSLSEVERAKLAVWDYPVSDKYTKMWQAMKEAKLPNTLEEAIERVQSSKSSSEGFAYLGDATNDIRYQVMIDCHLQMVGDEFSRKPYAIAVQQGSPLKDQFNNAILLLLNKRKLEKLKETWWNLNPERIQCEKQDNQSDGISGVFIVIFVGIGLACFTLAFEYWWYKYKKSSKVANTMNPKQMAMNKGGEFTYPVVPTFNTTSGMRSRSIIQGFRRSVGQSSPKQK  >RpadIR7c  MLYSQRDSDGAGGRPSDVQDVPARTIALVASGNISVFCSAYVVVGRHLETIDKYFEYFKSDAAERMLVVVVQDRKYIKPFIKYSPSYFNYKDVILINVYPQKESVYQMSIYFKKFVPISKQHDQWFRHDLQGKQKVKNFHNTRIKLLAYNNSFFSSVIKENGTGKISLSYGVECSIFKEIATRLNVTWDTYTSDDKDKWGTVWSNNTITGGALKWLYTKKVDVSFCSLWTDHIKSKFVDMSKFWTLTCLKFLVPKPRPLREKWDLLFKPFPLSLWLLVLFSATLTTFTVWILACVQKKIGYENINVFTSISATIFWIIGMMLLTNNPRTYRMGPIRHLINWWCIFIFIMSTSFSSILYSFITSFEYTDIVLEVEDMVKANYHWGLTYPPPYKYILKMQNPVHVEFSKRFIPERSVDDRIKRLKRGRYAILSKCLYKKHFMELEDVPVSMLNNLRTGRTCMNQFYIGFGFAKQSPYVKPANLVIQ  >RpadIR8a  MTVLKPEVWLSIVAALAMTAFMIWILDKYSPYSAQNNKPKYEQFRHFTLVESFWFALTSFTPQGGGETPKAISGRVLVAAYWVFVVLMLATFTANLAAFLTVERMQTPVQSLQQLARQSRINYSVVDGSDAHQFFRNMKMAEDILYNVWKEIALNQTNNRKDFRVWDYPIKEEYGQILAAIERTGTVPNRTVGYQMVLDNEQGEFALIHDSSDIEYEVYNNCNLTEVGEIFAERPYSIAVQQGSLIQEEISRKILDLQKDRFFELLNAKYWNASKISACPNSDDSEGITLESLGGVFIATLVGLLIALITLAFEVVYFKHKRAKVAEVSVVNNTVHKDKLIYGHELFMTLGRSSNSDDQTRWANKIKLDSTTGRLNNALFFRRNKFQN  >RpadIR25a  MRLEKVTVLNSQSVSAENVGSWKASLNSPIVVKDAANMTHFSAVTVYRVVTVLQNPFMIQVDDEDGKGVKFKGYCIDLIEEIRKLIGFEYEIYIAPDNNFGNMDENGQWNGMVKELVEKRADIALGSLSVMAERENVVDFTVPYYDLVGITILMKKPQTPTSLFKFLTVLENDVWMCILGAYFFTSFLMWIFDRWSPYSYQNNRNKYKDDEEKREFNLKECLWFCMTSLTPQGGGEAPKNLSGRLVAATWWLFGFIIIASYTANLAAFLTVSRLDTPVESLDDLSKQYKIQYAPLNGSSAMTYFQRMADIETRFYEIWKDMSLNDSLSEVERAKLAVWDYPVSDKYTKMWQAMKEAKLPNTLEEAIERVQSSKSSSEGFAYLGDATDIRYQVMIDCHLQMVGDEFSRKPYAIAVQQGSPLKDQFNNAILLLLNKRKLEKLKETWWNLNPERIQCEKQDNQSDGISIHNIGGVFIVIFVGIGLACFTLAFEYWWYKYKKSSKVANTLNPKQMAMNRGGEFTYPVVPTLNTTSGMRSRNIIQGFRRSVGQSSPKQK  >RpadIR31a  MNAIVDLLVLLVLILAGGKRCAGDTTITLGILYNEENSMLETAFKSSVDVAKAKMMDSGVQLDVVSKIVPLYDSFETQHHVCEMLVDGVSGMFGPSAGDTAPIVQSICDYKEIPHIQTRWDINQKRGSCQINLYPHPNTLAEALIDIIVAANWESFTIIYENNDSLMQITNILKSPPTNYPIRIRQLSSGPNYRKELREIKDSGETKILLDCSFSILTDVLLQAQQVGLMGSEHNFIIASLDMHTLDLDPFRYSGTKITGMRLVKPLELEFQNIVSQWTDNLSPLEPDDKIVLPETIQLESALIYDAVQLFTTSIYNLSKEFEISETSTPCNSSLSWKHGFTLINYMKMAKDFKGLTGKIKFDQEGFRTDIELELVDLTQDGLRVTGTWNTKTGINVSATPKPEIVPSGKDSDLRNMSFVVITALTQPYGMLKLSSNTLKGNDRYEGFGIDLIKELSEMSGFNYTFIIQEDSNSGYKDDKTKKWSGMIGEVINGQADLAIADISITRQREHDVDFTSPFMNLGISILYKKSTKSSPSLFSFLAPFSSFVWLWVITAYCGVSVLLFIMARISPYEWTNPYPCIEEPEYLENQFSLSNAFWFTIGSLMQQGSDIAPIAVSTRLVAGIWWFFTLIMVSSYTANLAAFLTVESVSEPFKDVEDLVNNQNIISFGLKKKGSTEEYFKESTNPTYKKLFDILQKNPAWYTANNDEGVEKVLKENYAFFMESTSIEYMVERNCKLAQIGGLLDNKGYGIVMKKNASFRNVLSANILSLQEKGKLTALKNKWWKEKRGGGACQVGTTVSTGVKKILTISHIINKLFACINRTESLLARKPTFRQTGRVVMQFYRRLGWGLFSFKNEKKLCFRSYNKCL  >RpadIR40a  MQLVRDNKNYAVIGGRETFYYDIKRFGAQHFHLSEKLNTRYSAIAFQRACPYRDNFDDVLMRLFEGGILSKITEEEYQKLNDKLMGSEKFDAASVVIEPVLEGSEPQQEDDDKQLTIAMSMKTLQGAFYVLAIGSILAGFLLLIEMRSHDKWKNDKRIKRIKAPFVYKQKAPIKFQNRLYDLKE  >RpadIR41a  MLYSQRDSDGAGGRPSDVQDVPARTIALVASGNISVFCSAYVVVGRHLETIDKYFEYFKSDAAERMLVVVVQDRKYIKPFIKYSPSYFNYKDVILINVYPQKESVYQMSIYFKKFVPISKQHDQWFRHDLQGKQKVKNFHNTRIKLLAYNNSFFSSVIKENGTGKISLSYGVECSIFKEIATRLNVTWDTYTSDDKDKWGTVWSNNTITGGALKWLYTKKVDVSFCSLWTDHIKSKFVDMSKFWTLTCLKFLVPKPRPLREKWDLLFKPFPLSLWLLVLFSATLTTFTVWILACVQKKIGYENINVFTSISATIFWIIGMMLLTNNPRTYRMGPIRHLINWWCIFIFIMSTSFSSILYSFITSFEYTDIVLEVEDMVKANYHWGLTYPPPYKYILKMQVSL  >RpadIR68a  TLHIMSTCIINMPISLGLQKNSPLKPAVDRFLRRVIEAGLVKKWLNDVMSDTVILEEPQQIEEVKALMDLKKLYGAFVVLVAGYILSILVLLFEIGYWYGVVKKDPLYDEYSLNCYYTQK  >RpadIR75b  MQSATIVVLFLLSGFAVCTAAAEDMERITIVGLFPSENSIEQMAFELAIHKVNLDPTLSEVKLEGRVEIVDINDGYQTSKKVCESFESGVGAIFGPTGYESSAIVQSICDSMEIPHIETHWKMNPKQQPNYYMNVYPDPMTLSRGYTAIVRDMDWTSFTLLYQRDEGLLRLQHLIQDYSGLTKLSDTELSAITIIKLPENNDFRPMLKEVKKSLESHIVLDCDTDIILTVLEQAEDVGLMDDYHSFIITSLDAHTINYGHLQFKRTNITAVKLIDPSSPTVTNIMADLEFVQQRMNLDMEVFRADTITVNALLMFDAVNVYAKALRGIGGTKVIKAEPNSCANRTSTGWSSGFSLINFMKVVEVDGLTGKLRFNQNSGYRSYFTLEMVELTNTGFKKIGVWDPQNEMSYTRTRNQMLDDLVNANMNKTFIVASKITEPYMMLKTDHKDRIGNDKYEGYVVDLIRMISEEINITYEFKLRNDGNGKKDKKTGKWDGLIGEVHELRADLAICDLTITHDRRTAVDFTTPFMNLGISILFSKPKEPETNLFSFTQPLSFHVWIYTATAYLGLSIILYILARITPNEWQNPHPCATEPEELENSLSLINCLWFSLGSILCQGSEVLPRAFSTRVCASMWWFFALIVTQSYTANWTAFLTSSRKESAIKRVEDLDKQSAIKYGCVRGQSTASFFENSDVNLYQKMFSVMETYGDTVMMFDNKQGVDRVKKEREAYAFFMESSTIEYEVQRNCDLTEVGSWLDNKAYGIAMPFNAPHRTAVSMALLKLSESGKLMELKDKWWSVSEEKMCPVPKKDSAELDVNEVGGMFVILILGCMLGLIFSLLEFLWNIRKVAVAEKLSPWDAFKLELKFVLKCHYSTKPVRHTTYSDEPSSEG  >RpadIR75c  MNKKSGRISVLSPDTIKTKTALMYDAVNFFATSLHGLVSTQTMGPSRIVCNDIKPWVHGYSLINYMRVMELNGLTGKMRFDAETGYRNYFKIDVVRVKESKKHRLGSWDPVEGMTLTRSASEMNLEFVQSITNKTFIVVGKLVQPYLMRCNATEKGMDKDEECFEGFAYDLVEEMAKYNGFKFKFTTNQDYGTMQKTGKWSGMIGELQSMRADLAICDLTITFDRRNAVDFTTPFMTLGISILYAKPEKKKPQLFWFLNPLSFSVWMYTATAYLGVSLFLFMLARITPNEWHDPQPWKDGNNELETRLNVANLIWFSCGTMLQQGSDISPQAVSTRLVAGMWWFFALIMTSSYTANLTASITSGRLDTPIKNVDDLSKDSNIQYGCYEEGSTAGFFQKSNLSLYQRMWSVMEASNPTVFTKSNQEGVDRVLKGKGRYAFLMESSSIEYQTERNCNLMEIGNTLDSKGYGIAMPMNSPYRTLISESVLRLQESGFMRELKDKWWKVQGGNKCEEEDESDELGFTKIGGVFVVLVLGCLIAFMFSILEFLWNIRKVAIEEEITPKEALILEWKFAMKCDGGVKPLRRRHHVNADTNSDTS  >RpadIR75d  MKLEIVVWIIHFASHVNTLPDTIRIGGLFHPNDINQENVFKHAIHDVNANRLILSRSNLSGQVEKVSPQDSFHASKRVCSLLRLGVAAVFGPQSAQISSHVQSICDTMEIPHLETRWDYKLRRESCLVNLYPHPTVLSKAYLDLVKKWGWKSFTIIYESNEGLVRLQELLKARNGAFSAYPITIRQLGSSRDHRPLLKQIKNSAESHIVLDCSTEKIYDVLKQAQQIGMMSDYHSYLITSLDLHTIDLDEFKYGGTNITGFRLVNPDTPVVQKVLKQWGENFTVMSTETALIYDAVHLFARALHDLDSSQKIDIKPLSCDASDTWSHGYSLINYMKIVEISGLTGVIKFDNQGFRTDFELDVVEVNKEGLSKIGTWNSSQGINFTRSFVEAYSSIVDNLHNKTLVVTLILSSPYTMRRESSLKLVGNDQFEGYAIDLIYEISKLLGFNYTLKLVPDGRYGSYNEDTKEWDGMMGELLQQRADLVVADLTITYDREQAVDFTMPFMNLGISILYRKPIKQPPNLFSFLSPLSLDVWIYMATAYLGVSVLLYILARFTPYEWYNPHPCNPDPDNLENQFSLMNCMWFAFGSLMQQGCDILPKAVSTRIVAGMWWFFTLIMISSYTANLAAFLTVERMDSPIESAEDLAKQTKIKYGALRGGSTTAFFRDSNFITYQRMWSFMESSRPSVFMSSNNEGVERVVKGKGNYAFLMESTSIEYVIERNCELTQVGGLLDSKGYGIAMPPNSPYRTAISGAVLKLQEIGKLHKLKTKWWKEKRGGGACRDDTSKSNSAANELGLANVGGVFVVLMGGMGVACVVAVFEFVWKSRKIAVEERIIKYHTKYRK  >RpadIR75d.1  SIAALVQSNSNSIKSIKNIAESSMTFSAQISPYGKLYFEETEDSDLKKLYKTKMVPRGNEAFVRAPEGMERIRTEFHGFLVEVMSAYKIIGKEWREEEK  >RpadIR76b  MNAIVDLLVLLVLILAGGKRCAGDTTITLGILYNEENSMLETAFKSSVDVAKAKMMDSGVQLDVVSKIVPLYDSFETQHHVCEMLVDGVSGMFGPSAGDTAPIVQSICDYKEIPHIQTRWDINQKRGSCQINLYPHPNTLAEALIDIIVAANWESFTIIYENNDSLMQITNILKSPPTNYPIRIRQLSSGPNYRKELREIKDSGETKILLDCSFSILTDVLLQAQQVGLMGSEHNFIIASLDMHTLDLDPFRYSGTKITGMRLVKPLELEFQNIVSQWTDNLSPLEPDDKIVLPETIQLESALIYDAVQLFTTSIYNLSKEFEISETSTPCNSSLSWKHGFTLINYMKMAKDFKGLTGKIKFDQEGFRTDIELELVDLTQDGLRVTGTWNTKTGINVSATPKPEIVPSGKDSDLRNMSFVVITALTQPYGMLKLSSNTLKGNDRYEGFGIDLIKELSEMSGFNYTFIIQEDSNSGYKDDKTKKWSGMIGEVINGQADLAIADISITRQREHDVDFTSPFMNLGISILYKKSTKSSPSLFSFLAPFSSFVWLWVITAYCGVSVLLFIMARISPYEWTNPYPCIEEPEYLENQFSLSNAFWFTIGSLMQQGSDIAPIAVSTRLVAGIWWFFTLIMVSSYTANLAAFLTVESVSEPFKDVEDLVNNQNIISFGLKKKGSTEEYFKESTNPTYKKLFDILQKNPAWYTANNDEGVEKVLKENYAFFMESTSIEYMVERNCKLAQIGGLLDNKGYGIVMKKNASFRNVLSANILSLQEKGKLTALKNKWWKEKRGGGACQDTDNNEASELSMKNVGGVFIVLCSGVAVAAVLAALEMFWTLWKTTSKEKVSFKSEFKDELKFIAKCRGSTKPARKNQNSSADNSNPTGSNQYMNSDRHYDYES  >RpadIR84a  MFLSYAFIVTVCLCHVVGQENENVPLSVVGFFDKENSIEQQMFELAIQKVNIDPKFANVFLKPEIKIIDSTDLYMTGLKVCETMESGIGAVFGPSNLESSNIVQSLCETMEVPRIETSWEDTPVNNFNFYFNPYPEPTLLAKGYTAIVHDMDWKSFTLLYQRPESLKRLQDLIQDYSGKTKPNDKQVAAISIIQLPEGNNFRPILKDIKKSLEGHIVLDCDADLLLTVFKQAKEVNLLDDYHSFIITSLDAHTVDFSSIVQNLRTNITTVRLIDPLSPFVENIVRDLNFVQQRMNLNMEPLKVNKLTVNAILIYDALNVFAKALKSLGMTNKIITEPLQCMNSPFIPWSNGFKLINFMRVIETEGLTGLLRFDNKTGHRSYFTLEMVELVDTGFKKIGLWDPERGMTYTRTSLEMLRDLYAGSKNKTFIVSSKITEPYLMLKEGHNKLEGNDKYEGYAVDLIQMIAEEINITYEFRLRNDGNGKRDKKTNKWNGIIGEVQEMRADLGVCDLTITHERRNAVDFTMPFMNLGISILFSKPEEPQTNLFSFTQPLSFQVWIFTATAYLGLSLVLFFLARITPNEWQNPHPCNPHPEELENSLSLLNCLWFSMGSILCQGSDILPRAFPTRLCAAMWWFFALIMTQSYTANWTAFLTSNRMETTIKNVEDLDKKGGTGTESIKYGCVTEQSTASFFQNSDVNLYQKMWSGMELNGDSVMVSDNKQGVDRVKKERNHYAFFMESSSIEYEVQRNCDLTEVGYWLDNKAYGIAMPFNAPHRTLVNMAVLKLSESGALMDLKNKWWSVTDDKRCKDLKKDTAELDVNEVGGMFVILILGCFIAFLFSILEFLWNIRKVAVEEKLTLWEAFMVELKFVLKCHGTSKPVRHVEDSSSDITTK  >RpNmdar1  MYKIFKVQFSMMKPVFSVFLLFLFFIFDCKSENLEKENTDNNPYEFHIGGVLSSNASEDYFKQTIEHLNFNTPFMKPGNTFYAHSIKMDPNPIKTALNVCKQLIVRRVYAVIVSHPQIGDLSPAAVSYTSGFYHIPVIGISSRDSAFSDKNIHVSFLRTVPPYSHQAEVWVELLKYFNYLKVIFIHSSDTDGRAFVGRFQTTSQNQGEDIEKKVQVEAVIEFEPGLFHFNNQLNEMKNAQARVYLMYASKIDAEIIFRDAANRNMTEAGYVWIVTEQALDANNVPEGTIGLKLVNASNELAHIYDSIYILASAITDMNRTKTITPPPADCDNSGAIWDTGKTLFEYVKKQVYKDGHTGKVAFDNNGDRIYAEYDIVNVKEVANKDAIGKYYFNNELNKMKLRLNESNIIWPGRLKKKPEGFMIPTHLKVLTIEEKPFVYVRPLKKDDGNSCKTDEIMCPLYNTSSKEPIVYCCKGYCIDLLVELSQTINFTYSLSLSPDGQFGNYEIRNNSASGKKEWNGLIGEIVYERADMILAPLTINPERAEFIEFSKPFKYQGITILEKKPSRSSTLVSFLQPFSHTLWVLVMGSVHVVALVLYLLDRFSPFARFKLINADGTEEDALNLSSATWFAWGVLLNSGIGEGTPRSFSARVLGMVWAGFAMIIVASYTANLAAFLVLERPKTKLSGINDARLRSTMENLTCATVKGSAVDMYFRRQVELSNMYRTMEANNYETAEDAIRDVKNDKLMAFIWDSSRLEFEAAQDCQLVTAGELFGRSGYGVGLQKGSPWSEAVTLSILDFHESGFMESLDDKWIFQGRVEQCEDQEKTPNTLGLKNMAGVFILVAVGIVVGMVLIVIEIGYKKHHVRKQNRLQLARNYGQTWRAIVQKRKMMRMQGRTSGVGALSLSVEALPRSVLNIQSPIRSIETLPRTCRNSPSPTRAWSGRQMVHRKSDDIPLRSPIGGPPQLHFHNNLM  >RpadIR93a  RKGGLNSPLNCFWYVYGALLQQGGAHLPDADSGRLVVGTWWLFVLVIVTTYSGNLVAYLTFPQMDTMVSNVADLMARKPQGYSWGIPKTSNLHSLLNLLPDDTMVKELIKNAEHHEGLEGLSRPVIERVRSGKHAFIHRRTNLMYIMKNDFLKTNRCDFAIGHEDFAEEKLAMMLAKESPYLSRINREIEKMHKVGLINKWLVDTLPKKDQCWTNTQLEVTNHKVNLDDMQGSFIVLLLGVLSSMVSFVFEYLLDKYINRRQIVITPFIN  >RpadNmdar2  MDLNVGLLMPKTSFGVRGYLRAIHDAIQVINKAYKKNRTTNFSKIYDFEEKNVRSQMMSLTPSPTAILDSLCKEFLNMNVSAILYLMNYEKYGRSTASAQYFLQLAGYLGIPVIAWNADNSGLERRASRSSLQLQLAPSLEHQTAAMLSILERYKWHQFSVVTSQIAGHDDFIQAVRERVADMQDKFKFTILNSVLVTKNSDLQQLVASESRVMLLYCTREEALRILQAARNFKITGENYVWVVTQSVMENLQAPQLFPVGMLGVHFDTSSESLVKEITTAIRVFAHGVEDFVNDPDNINETLTTQLSCEGEGESRWVTGEKFYKYLRNVSVEHTEPAKPKVEFTADGVLKSAELKIMNLRPGVGNSLVWEEIGVWKSWEREGLYIKDIVWPGDSHTPPQGVPEKFNLRITFLEEAPYITLAPPDPITGKCSMNRGVICRIAKGKDTEGLDEMEVKRNSSYYQCCSGFCIDLLEKFAEDLGFTYELVRVEDGKWGTLENGKWNGLISDLVNRKTDMVMTSLMINSDRESVVDFSLPFMETGISIAVTKRTGIISPTAFLEPFDTASWMLVGVVAIQAATFSIFAFEWMSPSGFDMKSVSQAPNHRFSLFRTYWLIWAVLFQAAVQVDIPKGITSRFMTNIWAMFAVVFLAIYTANLAAFMITREEYYDFIGIDDYRLSKPHSHKPMFKFGTVPHSHIDSTIHKYFPEMFSYMKSFNRSTVQEGLSSLLTAELDAFIYDGTVLDYLVTQDDDCRLLTVGSWYAKTGYAIAFQRNSKYVQMFNKRLLDFRENGDLERLQRFWMTGTCKPDDHDQNATSDPLALEQFLSAFLLLMGGTLLAALILLLECLYFNFFRKKLAKTDQGGCCALISLSMGKSLTFRGAVYEAQDFLKFRRCKDAVCDSQYQLLRNQLMSTYSKMKQLEKELELRKLKSEKRYQFSPASPLRQRDIVRPSIISTEYTNRYDHEPVRTEIAEMETVL  >Aspi54060  ELKEWMKYISIREDSTIMSTLSSAENYQLNVRGPFSFDETLNTWQGFVLNFNCENNKKIMRKVPLHLFKSTRYKWIIWSYEKTFLDALRLEDLDISFESDVKLISIDQNVSEIYEVYKFDPAWSLNVSSLGFWSEETGLQIRVTQPKSEERKNMHGKVITAGLALPELGTVSEEVYTNHHFLQSKDYYGRRDYDLWMLIAEMHNITSSIYVTDNYGTVKSATVIQYDGFIGKLQRKELDVGFSVIRNIERMRAVDYCRADVTLPPVQSINRAAFVFFEPVTLVRWRALYDSFSTSTWLVVGIISVLSACFFRFGLDLERNIRPSVCKYILFVIAAVSQQGLEIEPNKTPGRVVALTFMMFSFLVYNYYNTALIISLLSPPQYRINNLRDLVDSDLNLVSEDIPYNRAFFKSYNVSILNEAYEKKMKDKDAFTSLEDGLQKVRNGSFAFEADIRHLFLEMETLFTDSEKCNIRVIDLFPFKVSRAFVMQKDSHYNEFISSGFLLLLERGLTSHVEAVWDIKKPACFAREYAAAELDELLFAFFILAIGIILSGIAFGVEILTHREK  >Aspi14132.0  MAVARSMMRRIQGYVDVFFLHANCVLFAYDDTVADSIILQTTKRLGKPIILWNELQNLGTEKFCDGLVILAQETEAVSTFMQKAVLANWSFKVLVLHPTTSLQLGDRFIFTQEEMIRWRSYDMVLIEFGNHRFKVRQYLIHQTSFQHTTLPILESEMNPKKLMNFNQREMNISAFSCPLFVVFKRKDENGKPLHLKDIDGIEYRIFLEFARILNFRWNLQVPKGKDKWGMPRFKGEAPFGMIQMLIERKIELAICSWWLEVAKKKYIDLSTYWNSCCMTFLIPKPAPYSIQWSCFIRPFSLDLWLLLIITFCFITCVYWMNFDVRTRTIHPVIPFDRSLLQIYGMLLMNDGYDPQGRGNRKFNRFLLTLWGIFTLNIVSAYSGSLAARLTLPEFEPMIHSPAELSQKNYVFSAYYL  >Aspi8053.0  KHFNSMLNCFWYVYGALLQQSGTHLPEADSGRIVIGTWWLVVLVIVTTYSGNLVAFLTFPQMEDVVTNLDELLAKKDTLSWGIEEGSTLIPILKQADDGSRLHELYRNAKFHPQLNDRVMDKVREGRHVIIQRRTNLLFYMRKDYLRYDRCDFLLGRDEFIEESIAMIIAEGSPYLGIINERIRRMHQMGLIHKWITDALPKKDRCWSKSAANGVTNHSVNLDDMQGCFFLLLLGFLLAAVFVIGEIVKKKLGSKVGSRAIYPFTQ  >Aspi17580  FFKGIRLPSTDCRYRLVISVFILCAVYVIGDMYSANLTSILAHPSKEQPISTLSQLVDAMQFKDYQLLVERQSSSHALLENGTGTYSVMWKRMKKQRLYLIDSMEKGMKYVKERRNYALLGGRETFTFDTRRFGTQHFHISEKLNTKYSAIAVQLGCPFLELFNAQLMFLYEAGILNKITEEEYQKLGELRVEETEVTEGDSPKTGTTKQKQEDETMALSMKMLQGSFYVLAVGHLIATVVLGTEMLCHAYCGTFLAYSKKIVSLTRKKHINKSELLERHYRYLN  >Aspi4915  VGVCFNKIQNVTEIIKGDVPVLDLVQEIATLYLHDCMVILLYDDTFFQDAYLQMVVNRLLETFPFPLMHGAIDTTGTLRVNQGMLNPTELKCSHYIAFTKQLELMGDLLAERRDSKIVLVTTTTKWMVREYLSSTSAKRVPNLLIVSDPQLQEILYQNEDEDDDDDELPEEINLYTHKFYADSLGTSIPLILTTWRNGTFTRPHVNLFPPKFKHGFNGHRLAIAVADQPPFIFFRSGAMGKGNANYDSSGGWDGLEVRIIKLIAQMLNFSAEFKVMSDSNTADGPAGQVIEELKSRRVDVGLAGLYITADRINDIIFSPPHTQDCASFVTVTSTSVDRYRAVFGPFTGGVWIAVFFVYVFGIIPLSFSDSHTLKHLKSIKEWENMFWFVFGTFTNCFTFKGVSSWTSSKKLANRTFIAAYWVFTIIITAAYTGSIIAFITLPALPHMIESSKELLRGGYKISLLNEGGWQRWFTDLEDPVGKKLLKSPIYVPDIHSGLRNVTKTNYLRRYSFLGSFNLIEWYLRTNSTERQNLKATFHLSSECFVPFMVSMAYSKQAMFVYEMNEALLKAGQAGYIAKFERDLEWEYYRLAGTRLLMEAATKLVPEDRSLTIEDVSGVFILMVFGYVLGQIVLLAECLHLFKAKRDCEKRVDPSSGTVDLSVTDISQTPSIVISDYDIKAKNYNSDPSDLQTRISASVAALRSHLMDTVNVRSQSC  >Aspi2243  MKDLGITWQPKEVDCDAPSKGDSVSSEFQNQVSKLVSNSSVLKFEEDSKRIHYQTFFEFKSISRSSPSGATPFATSDSAEIQVHNESNIQPLRRFFRVGTTLAIPWSYPSVDPVTGNVLKDEMGNEIWEGYCIELVKKLSDDMKFDYQLVIKDSFGNRFPNGSWDGLIGELATGQIDFISTALTMTSEREEVIDFVPPYFDQTGITIVIRKGIRKTTIFKFMTVLKLEVWLSIVAALILTAVLLWLLDKYSPYSAQNNPKLYPYECRKFTLKESFWFALTSFTPQGGGETPKSFSARVLVSAYWLFVVLMLATFTANLAAFLTVERMQSPVQSLEQLAKQSRIAYTVVNGSTTHQYFKNMKFAEDTLYRVWKEITLNASSDQSQYRVWDYPIKEQYGHILYAIEQGEPVPNVSVGFQKVIDSENGEFALIHDSAEIRYEVSRNCNLTEVGEIFAEQPYALAVQQGSRLQEEISRNILDLQKDRFFEILTAKYWNSSYRGQCSDADDNEGITLESLGGVFIATICGLILAMITLSFEVYFHKRKKRAQTIPELSGGSENSFPELNDPKAPTKLMYAKEFENAVMRNRREFSDNWRREANKIKFITVQPRSHLY  >Aspi52928  IMNVRYSGFCVFLQLISSVLSIPEVINLGGLFHSTDEIPKIAFKYATQLVQKDPHLLPNTTIITQMLDVPRYDSFQVQLKVCELMGKGISAVFGPQTGSTSSIVQSLCDNKEIPHIQSRWDYYQRRGSCLVNLYPHPSILLKALFDIVEAWNWKSFTIIYENNDSILKVSELLKIHDTKGFPVVLRQLNADDNHRPVLREVKDSGETNIVLDCSDEKLFQVLKQAQQVGLMGSEHSFIITSLNLHAMDLEPFQYSGTNITGMRLFDPEDPQVQNVVKYWVDAELSEGRILDMTPEKLPVEAALIHDAVLLFAKAFEHLNMSEELANQPLQCEDTLSWDQGYSVINYMKVSELKGLTGHIVFDHEGFRTNIHLHLIELTAEGLNLLGTWNTTEGLRTITAEDEEDPSVALQSDDLRNMTFIVITALTPPYGMLKESSQKLLGNDRFEGYGIDLIHELSLMSGFNYTFRIQADKSSGNPDPKTGRWSGMIGEVLEGRADLSIADMTITRERESAVDFTLPFMNLGISILYKSPTKLAPSLFSFLSPFSNEVWIYMIAAYVGVSVLLFIMARISPYEWNNPYPCIKEPEELENQFSMLNSFWFTIGSLMQQGSEIAPIAPSTRMVAGIWWFFTLIMVSSYTANLAAFLTVEDLSLPFRNVEELANQNTIKYGAKAGGSTANFFRDSNHTTYQRMWQYMQANPEVMTSSNQEGVDRVVNEDYAFLMESTSIEYEVERKCELSQVGGLLDNKGYGIVMRKNSPYRNVLSTNVLSLQEKGVLSQLKKKWWKEKRGGGACQGEGGEEEVTDEGLDLDNVGGVFVVLAAGVVVGIFLAFAEMTWHLWRVSVNEHVSFKEEVIAELKFVARFHGSTKPVRRAVRGSSRATSVDQTNFDNSNAYPHHFAIDSDSKQPLD  >Aspi3371.0  DWFINVVREVTTIAKMTGSLVQMFLMTMCTNYPVMNVTTPEPPPWQWIPERKTPRSEGFYCMLKQIPQLKKELLRGKKLRVVTIDHWPVSWIKNVNGTMIGQGIAFEILNTLMEKFGFTYEIRLPSSMSLLDEKGTITDMLKTRQADMVAAFVPNLPDLEHVLEWGVELCQYEYVVLTKRPRESATGSGLLAPFAKEVWILILVALICTGPVIFIIMKIRAWLCRPSQVYSLTACVWFVYGALMKQGSTLSPIHDSTRVLFATWWIFIMILTAFYTANLTAFLTLSKFTLSIHTVNDIARSSSPWMAEKGAAFEYGVQMDEDLTVLKRSLERGYGSYVNLTEVDQDHIAELVMNGALFIQDRFWLNFFMLNVEKQVIDDDMHCQYVLTTEKYLTSSLSFVYPKNSILPRIFNPVMLSYVESGIIKHLLTKDLPDATICPLDLGSKERQLRNADLATTYYVILGGFTSACLVFLCEVIVRHYHPEEDATLWPNNDKFGFIEVKPKMNPFSHFMNLKVQPERLPGMPTTINGREYVVYNNREGQKKLIPMRTPSALLFYSKWQQ  >Aspi14487  MFLFLLCASFLDGYQKENITFVKTETALMYDAAHLFAKALHDLDSSQSIDIKPLSCDAVNTWPHGYSLINYMKIVELTGLTGIIKFDNQGFRSNFMLDVVELTKEGLTKIGTWNSTEGVNFTKSYGEAYTQIVESLHNKTFVVTVILSSPYVMLKDSSEKLVGNDQFEGYGIDLIADIARILGFNYTVKLVPDGRYGSLNKQTGEWDGMMKELLDQRADLAIADLTITYDREQAVDFTMPFMNLGISILYRKPIKQPPNLFSFLSPLSLDVWIYMATAYLGVSVLLFILARFSPYEWDNPHPCRDEPDVLENQFSLLNSLWFTIGSLMQQGSDIAPKAVSTRMVAGMWWFFTLIMISSYTANLAAFLTVERMDSPIESAEDLAKQTKIKYGALRGGSTAAFFRDSNFTTYQRMWSFMESQRPSVFTASNVEGVERVVRGKGSYAFLMESTSIEYVIERNCDLTQVGGLLDSKGYGIAMPPNSPYRTAISGTVLKLQEEGRLHILKTKWWKEKRGGGSCRDDTSKSSSAANELGLANVGGVFVVLLGGMGVACVIAVCEFVWKSRKIAIDERVRTMYT  >Aspi50436  ERRVWGDNSESVNSRGVWRRGARRDEMWCSPTWFSLHFLFILSKFYSCHGRNSEEETTREIGIAGLFGAEDEVEERSFRMAIQRINLNKNLLPNVTLVPKIGVISEIDSFNTSRTVCNLAAEGVAAVFGPNTDEVTGIVHSVCDTLDLPHIKTHWDISNEPLRGINIYPNADLLAQALIDTIEDMDWRTFTIVYETTEGLIRLQEVLKKHERVINVHGHAPVTIKQLPPNGDYRPLFKEILQSSENHVVLDCRTELILDILRQAKEVKMMGDYQSYVLTSLDAHTLDYSEFRSTRTNITSLRLVNPNSTVVQYALQDWIHEEQRAGRRSHLTPDSITVEAALIHDAVYLFAEALHMILGTEKDLTTQSLQCGTLSGDDQWSGEKWLHGFKLSNFMKLLDIEGMTGPMRFNSTSGSRTYFELEISEILPHLKKIGSWDPIDKIKYTRTRSEMYEDIVQAISNKTFIVSARLGEPYLMLKEEEGLEGNDKYRGYSIDLIDAISKDLGFKYRFELVPDGNYGSLNKQTKKWNGLIRELQDMKADLAICDLTITYERRTAVDFTMPFMTLGISILYSKPEKQEPDLFSFLSPLSLEVWIFMATAYLGVSILLFILARCTPNEWESSHPCESEPEELENALNLNNCLWFSLGSVLAQGCDILPKAVSTRLVAGMWWFFTLIMISSYTANLAAFLTKTGMETTISSVEDLAKQSKVKYGCLLGGSTGAFFSSSNDSLYQKMWSVMESTRPSVFVKTNAEGVDRVMKGKGSYAFFMESTSIEYQVERNCENLMQVGGLLDSKGYGIAMPFNSPYRTAISGSVLKLQESGGLRELKNKWWKVQGQEACVEGVQEDSQELGIANVGGVFVVLILGCSFAFCFALLEFLWNVRKVAVEEKLTLLEAFMVEVKFVLSCSENKPVRRRPESELSNNMDTSFIHQLNSVQ  >Aspi4133  MFQIAKLRFGGAARFRSFIYCLWLLSSCQHTSTLPPVIRIGAIFTQDQKDSSSELAFKYAVYKINKDKNLLPDTTLVYDVQHVPRDDSFHASKKACRQMEHGIQALFGPSDPLLGAHIQSICEALDVPHIEARMDFEPSFKEFSINLHPSQEHMNHAFKDLISFLNWTKVAIVYEEDYGLFKLQDLVKAPPATRTEMYIRQANPSTYRQVLREIRQKEIFKLIVDTNSRHMYKFFRAILQLQMNDHRYHYMFTTFDIETFDLEDFKYNSVNITAFRVVDVENTKVAETLDQMEKFQPIGHAILNKSGVIQAEPAFMYDSVHIFAKGLSALNELAPVNLSCDLETPWKNGLSLYNYINSAGLNGLTGRIEFKEGKRSNFKLDLLKLKKEELRKVGHWTPLEGINITDPTAFYESSATNITLVVMTREERPYVMIREDKNLTGNARYEGFCIDLLKTIATQVGFHYAIRLVPDNMYGVYDNEKKEWNGIVRELIEKRADLAVASMTINYARESVIDFTKPFMNLGIGILFKVPTSQPTRLFSFMNPLAVEIWLYVLTAYILVSFTLFVMARFSPYEWNNPHPCSIESDVVENQFSVSNSFWFITGTFLRQGSGLNPKATSTRIVGAIWWFFTLIIISSYTANLAAFLTVERMITPIENAADLSEQTEISYGTLAGGSTMTFFRDSKIGIYQKMWRFMESKKPSVFVASYEEGVRRVLGGNYAFLMESTMLDYAVQRDCNLTQIGGLLDSKGYGIATPKGSPWRDKISLAILELQEKGTIQIMYDKWWKNTGDVCNRDDKSKDSKANALGVENIGGVFVVLLCGLALAILVAILEFCWNSKKNAQTDRQSLCSEMAEELRFAVRCHGSRQRPALKRSCTRCSPATTYVPAALELPLHLNGETGIMPMVEMKKGSLSYEPDI  >Aspi605  MLTHNENVTGDGPSGRKFELQAYVDVINTADAYKLSRLICNQFSRGVFAMLGAVSSDSFDTLHSYSNTFEMPFVTPWFPEKVLSPSSGSIDYAISMRPDYHQAIIDVIKHYGWRHIAYLYDSHDGLLRLQQIYQGLQPGSELFRVDVVRRISNVSQAIDFIHALEVVDRWSHKYIVLDCSIAMAKEIVVAHVRNITLGKRIYHYLLSGLVMDDQWENEVKEYGAINITGFRIVDPSRKYVQDFLRGWHQLEPTKSPGSSHESISAQAALMHDAVFVLVEVFNKILRKKPDTFKGNLRRGQMINNGTRGIDCNTSKGWVTPWEHGEKISSQLRKVQVEGLTGEIRFNENGKRKNYTLHVMEMTMHSAMIKVAEWSDVSGLTVLAPKSEKSRPPTEIEKNRTYVVTTIDEEPYIIIKKPEPHEQLVGNDRFEGYCKDLADLIAKKLDINYELRIVKDGKYGSENPDKKGGWDGMIGELVRREADMAIAAMTITAEREKVVDLSKPFMSLGISIMIKKPVKQKPGVFSFLSPLSKEIWVCVLFSYVAVSIVLFIVSRFSPYEWRLISYTEEVHHNQGIAPAVLTNDFSILNSLWFALGAFMQQGSDISPRSISGRIVGSVWWFFTLIIISSYTANLAAFLTVERMVAPINSAEDLAAQTDVQYGTLKGGSTWDFFKKSQITLYQKMWEFMSARPHLLVQTYDEGIRRVRSSKGKYALLIESPKNEYINEREPCDTMKVGRNLDSKGFGIATQLGSPLSNPINLAVLSLTENGELAKLMNRWWYDRTECKLDDQDPSRSELSLSNVAGIFYVLIGGLILAMAVALLEFCYKSHSEASRAKIPLTDAIKAKARLTIGSREYENNRKRETLYQYYASGNQMNQLDSDQVLHSNTHTQV  >Aspi33013  MMRAIVPALHLLLCIGCLHYVSTDARNPTTFYIGGVLSNNESLSHFKNIISVLNFDSTFVNRGVTFYDAAILIDANPIRTALNVCKDLIGSKGVYAVIVSHPLIGDLSPAAVSYTSGFYHIPVIGISSRDCAFSDKNIHVSFLRTVPPYSHQADVWVELLKHFNYMKVIFIHSSDTDGRALLGRFQTTSQNQEDDVEIKVQSGSENLNDSHLTTRDSLQVESVIEFEPGLGTFHEQLKEMKNAQARVYLMYASKKDAEVIFRDAEALNMTDAGYVWIVTEQVLNASNAPEGIIGLRLMNATSEEAHIKDSVHVLASALREMNKTVTITEAPQDCDNSGSIWETGKMLFEYIRKQVLLNGATGKVAFDDSGDRIFAEYDVVNLQEGRRIVPVGQYYFNAEQDKMKLRIDDGNITWPGRQQTKPEGFMIPTHLKVLTIEEKPFVYVREISEAAEAAGEGCGPEEIPCPHFNSSDEDSRSFCCRGFCIDLLRQLSQTINFTYNLALSPDGQFGTYLIKNTSVGGKKEWTGLIGELVAERADMIVAPLTINPERAEFIEFSKPFKYQGITILEKKPSRSSTLVSFLQPFSNTLWILVMVSVHVVALVLYLLDRFSPFGRFKLSNSDSTEEDALNLSSAIWFAWGVLLNSGIGEGTPRSFSARVLGMVWAGFAMIIVASYTANLAAFLVLERPKTKLTGINDARLRNTMENLTCATVKGSAVDMYFRRQVELSNMYRTMEANNYDTAEDAIHDVKIGKLMAFIWDSSRLDFEAAQDCELVTAGELFGRSGYGIGLQKGSPWSDFVTLAILDFHESGFMESLDNKWILQGNQPQCEQYEKTPNTLGLKNMAGVFILVAAGIVGGVGLIVIEMVYKKHQIRKQKRMELARHVADKWRGAIEKRKTLRATIAAQRRLKSNGVNETGEMVPRAVLQTVSPIRSVESLPRNVIDAPSPIRVWPGSNIRQRRTDEIHSPPAYDAQPSDLIV  >Aspi11154.0  DTWCNVSISVNANVRNCWQSRIKQLNSSLSYNFLNFFYSNLKTRTHSALRGSKDKSMLGDRNSMAARFQILNLVPEQPARSLVDSNDTGETKKWRAVGEIVGESDVRLDSLVWPGGQLVPDSTGKGARSVFRIVTAIAPPFVMEGELDEDGQCLRGLECHRILTSDKDNLTLVFNEMERIEEQEEQVDEKEKERLEKMQDIWGIRPNTMFSNSRNKYVTNCCYGLTMDLLENVAHELEFDFHLYLVADGSYGTKIMKDGNSAWNGIVGDLVSGAAHMSFSALSVSSARADVIDFTAPYFFSGVSLLAAPKQRNDIPLLAFLLPFSPELWIAIFTSLHVTAVAVAIYEWLSPFGLNPWGRQRSKNFSMASALWVMWGLLCGHLVQFKAPKSWPNKFLINVWGGFSVIFVASYTANIAALIAGLFFQVAVGDYHDRNLLLQRVGAPWASAADYYVRRNNPQLWEHMQRYAVKTVAEGVHHLKNGSLDILIADTPILDYYRATDHGCKLQKIGDTINEDTYAVGMTKGFPLKDSVSAVIAKYSSNGYMDILTEKWYGGLPCFKLASDMAQPRPLGIAAVAGVFLLLGLGMAVGCVILFIEHLFYKHTLPILRQKPKGTIWRSRNIMFFSQKLYRFINCVELVSPHHAARELVHTLRQGQITSLFQKSVKRKEHEQRTRRKSKGQFFEMIQEIRRVQQEERDYDSPENKGSPKKKHKKLNNNNRSPQLLLSPPDLMKQQRRLSPSRLDIARRLSKDFFRSKSSGNLSVRRLSSDISVAAGKFFDQNSQAIGRRLSHGCEENSPPDLNSRRSSQRMGESPVTTSPAGSGTFAPEASDPEPPRSPNLLSPGFNFGSKMPPEPPRSPREVGPVAARRFTYTDISSPNFTKKFPLKPCTEPTPKVVINCDNSDKVTEDVPGAEPDTVFREEDEVGMSPLDRNNWSNVSDRSTEHMIKIFIPASEVIDDKIVEEDETSHAIRPKKRKSPTGDDSTKPKIVSYVPKKVTRSTETEVAPSGSAASGSLERLSKEELSALCQLSDSELKSKLYQALKSKDPT |

**TABLE S2 |** Primers used in this work.

| Gene names | Primers | Gene names | Primers |
| --- | --- | --- | --- |
| qAspiRPS28-F | TCCAGTGGTTCTGAAGCAACA | qAspiOR2-F | TTGCTGTACGGTTGTAGTTGTT |
| qAspiRPS28-R | TTTCGGAGTAAAGTGGCAAAGG | qAspiOR2-R | GTGGCTCGGTCTTCCTCAT |
| qAspiOBP1-F | GCCTGCTCACGATGTAACTG | qAspiOR3-F | CCTCCTCCTCCAATACCATCT |
| qAspiOBP1-R | GAACCATCCAAGCTCCCAAG | qAspiOR3-R | ACGAAGGTTGTCAGTAATGTCT |
| qAspiOBP2-F | GCAGGGCTGAGACATCAAC | qAspiOR5-F | CTCGACATATTGGCGGAGAATT |
| qAspiOBP2-R | GGACATTCTCCCGATTCTTCTT | qAspiOR5-R | AGCAGTTAAGAAAGGCGTGAAA |
| qAspiOBP3-F | GCTGACGGGCACTATGTTG | qAspiGR4-F | ATCACTCTCGCTGCTTGCT |
| qAspiOBP3-R | ATCGGTGGCTGATGTGATTATC | qAspiGR4-R | ATATTCCGCCTCCTCCTTGG |
| qAspiCSP12-F | TTGTTCCTCACCTGTCTTCG | qAspiGR6-F | CAGGTCTTATCACTGTGAAGGA |
| qAspiCSP12-R | GATTGCCCGTCCCTCAGAT | qAspiGR6-R | CCAATCCACAGCCTGAACTC |
| qAspiORco-F | AGTGCCTGACGAGACCTATC | qAspiGR8-F | TTCGGAGCATCGGCAGTT |
| qAspiORco-R | GCTCCTTCTTGCTTGCTTAGT | qAspiGR8-R | TCTGTGTCCATGATATCTCTCA |
| qAspiGR1-F | GCATACGCATATCCTTATGTTC | qAspiIR7-F | ACTGTTCGCTACATGGTGGAT |
| qAspiGR1-R | AAGTGGACGTTGTATTCATAGC | qAspiIR7-R | ATCGTTGACCGTGTGAATAGAC |
| qAspiGR3-F | AGATGTTGACAATGGTGGAGTG | qAspiIR6-F | ATGACCTGGCATCTATGGAGAG |
| qAspiGR3-R | CGGATAGAGTGATGATGGTGTT | qAspiIR6-R | GCGGACTGGCTTAGTGGAA |
| qAspiNmdar-F | GCAAGACACTACGAGCAACAA | qAspiIR8-F | CGAGATGAGCCAGATGTCCTA |
| qAspiNmdar-R | AGCGATTCTACCGACCTGATAG | qAspiIR8-R | CACCACATACCTGCCACCAT |
| qAspiIR2-F | AGCAGAAGGTAGTCCGTACTTG | qAspiIR9-F | TGCGAGCCGTAGATTACTGT |
| qAspiIR2-R | AATGATTGGTGACTCCGTTAGC | qAspiIR9-R | CGACTACTAACCACGTTGATGT |
| qAspiIR3-F | TTCGTTCCGCCATACTTCG | qAspiIR11-F | TGTTGGTGCTATCTGGTGGTT |
| qAspiIR3-R | GACAATACTTAGCCACACCTCT | qAspiIR11-R | ATCTCCGTCTGCTCCGAAAG |
| qAspiIR4-F | ACTCAGGTCTCCGCAATGTAA | qAspiIR13-F | GCGGTCATCTTGTCCAGTTC |
| qAspiIR4-R | AATTCTGCCGTTCTGTGCTATT | qAspiIR13-R | AGCGATTAAGGCGGCGATA |
| qAspiIR5-F | AGACTTCCATCTACCGACTGTA |  |  |
| qAspiIR5-R | AGTGTGCTGATAGGTTGTTCTT |  |  |

**TABLE S3 |** Summary of *A. spiniferus* transcriptome annotation.

| Database | Number of Unigenes | Percentage (%) |
| --- | --- | --- |
| Annotated in NR | 18576 | 24.66 |
| Annotated in NT | 7082 | 9.4 |
| Annotated in KO | 7949 | 10.55 |
| Annotated in Swiss Prot | 12986 | 17.24 |
| Annotated in PFAM | 18836 | 25.01 |
| Annotated in GO | 18830 | 25 |
| Annotated in KOG | 7614 | 10.11 |
| Annotated in all Databases | 2778 | 3.68 |
| Annotated in at least one Database | 25706 | 34.13 |
| Total Unigenes | 75298 | 100 |

**TABLE S4 |** Transmembrane domains in ORs, GRs and IRs.

| Chemosensory receptor | TMD | Chemosensory receptor | TMD |
| --- | --- | --- | --- |
| *AspiORco* | 7 | *AspiIR1* | 2 |
| *AspiOR2* | 6 | *AspiIR2* | 2 |
| *AspiOR3* | 6 | *AspiIR3* | 3 |
| *AspiOR4* | 3 | *AspiIR4* | 4 |
| *AspiOR5* | 2 | *AspiIR5* | 2 |
| *AspiOR6* | 2 | *AspiIR6* | 3 |
| *AspiGR1* | 4 | *AspiIR7* | 4 |
| *AspiGR2* | 1 | *AspiIR8* | 3 |
| *AspiGR3* | 2 | *AspiIR9* | 4 |
| *AspiGR4* | 3 | *AspiIR10* | 3 |
| *AspiGR5* | 1 | *AspiIR11* | 3 |
|  |  | *AspiIR12* | 3 |
|  |  | *AspiIR13* | 4 |
|  |  | *AspiNmdar1* | 3 |
