# Supplementary material for: Characterization of Antennal Chemosensilla and Associated Chemosensory Genes in the Orange Spiny Whitefly, Aleurocanthus spiniferus (Quaintanca)
Source: Front Physiol. 2022 Feb 28;13:847895. doi: 10.3389/fphys.2022.847895 (PMC8920487; doi:10.3389/fphys.2022.847895)
Supplement: Supplementary Table S2 — Primers used in this work. [file Table_2.docx]

**TABLE S2 |** Primers used in this work.

| Gene names | Primers | Gene names | Primers |
| --- | --- | --- | --- |
| qAspiRPS28-F | TCCAGTGGTTCTGAAGCAACA | qAspiOR2-F | TTGCTGTACGGTTGTAGTTGTT |
| qAspiRPS28-R | TTTCGGAGTAAAGTGGCAAAGG | qAspiOR2-R | GTGGCTCGGTCTTCCTCAT |
| qAspiOBP1-F | GCCTGCTCACGATGTAACTG | qAspiOR3-F | CCTCCTCCTCCAATACCATCT |
| qAspiOBP1-R | GAACCATCCAAGCTCCCAAG | qAspiOR3-R | ACGAAGGTTGTCAGTAATGTCT |
| qAspiOBP2-F | GCAGGGCTGAGACATCAAC | qAspiOR5-F | CTCGACATATTGGCGGAGAATT |
| qAspiOBP2-R | GGACATTCTCCCGATTCTTCTT | qAspiOR5-R | AGCAGTTAAGAAAGGCGTGAAA |
| qAspiOBP3-F | GCTGACGGGCACTATGTTG | qAspiGR4-F | ATCACTCTCGCTGCTTGCT |
| qAspiOBP3-R | ATCGGTGGCTGATGTGATTATC | qAspiGR4-R | ATATTCCGCCTCCTCCTTGG |
| qAspiCSP12-F | TTGTTCCTCACCTGTCTTCG | qAspiGR6-F | CAGGTCTTATCACTGTGAAGGA |
| qAspiCSP12-R | GATTGCCCGTCCCTCAGAT | qAspiGR6-R | CCAATCCACAGCCTGAACTC |
| qAspiORco-F | AGTGCCTGACGAGACCTATC | qAspiGR8-F | TTCGGAGCATCGGCAGTT |
| qAspiORco-R | GCTCCTTCTTGCTTGCTTAGT | qAspiGR8-R | TCTGTGTCCATGATATCTCTCA |
| qAspiGR1-F | GCATACGCATATCCTTATGTTC | qAspiIR7-F | ACTGTTCGCTACATGGTGGAT |
| qAspiGR1-R | AAGTGGACGTTGTATTCATAGC | qAspiIR7-R | ATCGTTGACCGTGTGAATAGAC |
| qAspiGR3-F | AGATGTTGACAATGGTGGAGTG | qAspiIR6-F | ATGACCTGGCATCTATGGAGAG |
| qAspiGR3-R | CGGATAGAGTGATGATGGTGTT | qAspiIR6-R | GCGGACTGGCTTAGTGGAA |
| qAspiNmdar-F | GCAAGACACTACGAGCAACAA | qAspiIR8-F | CGAGATGAGCCAGATGTCCTA |
| qAspiNmdar-R | AGCGATTCTACCGACCTGATAG | qAspiIR8-R | CACCACATACCTGCCACCAT |
| qAspiIR2-F | AGCAGAAGGTAGTCCGTACTTG | qAspiIR9-F | TGCGAGCCGTAGATTACTGT |
| qAspiIR2-R | AATGATTGGTGACTCCGTTAGC | qAspiIR9-R | CGACTACTAACCACGTTGATGT |
| qAspiIR3-F | TTCGTTCCGCCATACTTCG | qAspiIR11-F | TGTTGGTGCTATCTGGTGGTT |
| qAspiIR3-R | GACAATACTTAGCCACACCTCT | qAspiIR11-R | ATCTCCGTCTGCTCCGAAAG |
| qAspiIR4-F | ACTCAGGTCTCCGCAATGTAA | qAspiIR13-F | GCGGTCATCTTGTCCAGTTC |
| qAspiIR4-R | AATTCTGCCGTTCTGTGCTATT | qAspiIR13-R | AGCGATTAAGGCGGCGATA |
| qAspiIR5-F | AGACTTCCATCTACCGACTGTA |  |  |
| qAspiIR5-R | AGTGTGCTGATAGGTTGTTCTT |  |  |
