# Supplementary material for: Characterization of Antennal Chemosensilla and Associated Chemosensory Genes in the Orange Spiny Whitefly, Aleurocanthus spiniferus (Quaintanca)
Source: Front Physiol. 2022 Feb 28;13:847895. doi: 10.3389/fphys.2022.847895 (PMC8920487; doi:10.3389/fphys.2022.847895)
Supplement: Supplementary Table S3 — Summary of A. spiniferus transcriptome annotation. [file Table_3.docx]

**TABLE S3 |** Summary of *A. spiniferus* transcriptome annotation.

| Database | Number of Unigenes | Percentage (%) |
| --- | --- | --- |
| Annotated in NR | 18576 | 24.66 |
| Annotated in NT | 7082 | 9.4 |
| Annotated in KO | 7949 | 10.55 |
| Annotated in Swiss Prot | 12986 | 17.24 |
| Annotated in PFAM | 18836 | 25.01 |
| Annotated in GO | 18830 | 25 |
| Annotated in KOG | 7614 | 10.11 |
| Annotated in all Databases | 2778 | 3.68 |
| Annotated in at least one Database | 25706 | 34.13 |
| Total Unigenes | 75298 | 100 |
