# Supplementary material for: Characterization of Antennal Chemosensilla and Associated Chemosensory Genes in the Orange Spiny Whitefly, Aleurocanthus spiniferus (Quaintanca)
Source: Front Physiol. 2022 Feb 28;13:847895. doi: 10.3389/fphys.2022.847895 (PMC8920487; doi:10.3389/fphys.2022.847895)
Supplement: Supplementary Table S4 — Transmembrane domains in ORs, GRs and IRs. [file Table_4.docx]

**TABLE S4 |** Transmembrane domains in ORs, GRs and IRs.

| Chemosensory receptor | TMD | Chemosensory receptor | TMD |
| --- | --- | --- | --- |
| *AspiORco* | 7 | *AspiIR1* | 2 |
| *AspiOR2* | 6 | *AspiIR2* | 2 |
| *AspiOR3* | 6 | *AspiIR3* | 3 |
| *AspiOR4* | 3 | *AspiIR4* | 4 |
| *AspiOR5* | 2 | *AspiIR5* | 2 |
| *AspiOR6* | 2 | *AspiIR6* | 3 |
| *AspiGR1* | 4 | *AspiIR7* | 4 |
| *AspiGR2* | 1 | *AspiIR8* | 3 |
| *AspiGR3* | 2 | *AspiIR9* | 4 |
| *AspiGR4* | 3 | *AspiIR10* | 3 |
| *AspiGR5* | 1 | *AspiIR11* | 3 |
|  |  | *AspiIR12* | 3 |
|  |  | *AspiIR13* | 4 |
|  |  | *AspiNmdar1* | 3 |
